# A reference catalog of DNA palindromes in the human genome and their variations in 1000 Genomes

**DOI:** 10.1038/s41439-020-00127-5

**Published:** 2020-11-20

**Authors:** Madhavi K. Ganapathiraju, Sandeep Subramanian, Srilakshmi Chaparala, Kalyani B. Karunakaran

**Affiliations:** 1 Department of Biomedical Informatics, School of Medicine, University of Pittsburgh, 5607 Baum Blvd, Suite 401, Pittsburgh, PA 15206 USA; 2 Intelligent Systems Program, School of Computing and Information, University of Pittsburgh, Pittsburgh, PA 15213 USA; 3 Language Technologies Institute, Carnegie Mellon University, Pittsburgh, PA 15213 USA; 4 Supercomputer Education and Research Centre, Indian Institute of Science, Bangalore, 560012 India

**Keywords:** Genome informatics, Sequence annotation, Data mining, Predictive markers

## Abstract

A palindrome in DNA is like a palindrome in language, but when read backwards, it is a complement of the forward sequence; effectively, the two halves of a sequence complement each other from its midpoint like in a double strand of DNA. Palindromes are distributed throughout the human genome and play significant roles in gene expression and regulation. Palindromic mutations are linked to many human diseases, such as neuronal disorders, mental retardation, and various cancers. In this work, we computed and analyzed the palindromic sequences in the human genome and studied their conservation in personal genomes using 1000 Genomes data. We found that ~30% of the palindromes exhibit variation, some of which are caused by rare variants. The analysis of disease/trait-associated single-nucleotide polymorphisms in palindromic regions showed that disease-associated risk variants are 14 times more likely to be present in palindromic regions than in other regions. The catalog of palindromes in the reference genome and 1000 Genomes is being made available here with details on their variations in each individual genome to serve as a resource for future and retrospective whole-genome studies identifying statistically significant palindrome variations associated with diseases or traits and their roles in disease mechanisms.

## DNA palindromes: Reference catalog contributes to disease research

A toolkit to detect DNA patterns has pinpointed millions of palindromes within the human genome, which could further the understanding of their association with disease. Madhavi K. Ganapathiraju of the University of Pittsburgh and colleagues used their Biological Language Modelling Toolkit to mine the human reference genome and the genomes of 2504 individuals from the 1000 Genomes project for palindromes: DNA sequences with two complementary halves, where folding at the midpoint leads to complementary base pairs lining up with each other. They identified their locations and lengths and looked for known disease-associated genetic variants, called single nucleotide polymorphisms, within them. They found links to diseases such as diabetes, obesity, Alzheimer’s disease, Crohn’s disease, and some cancers. The data was compiled into the Catalog of Palindromic Sequences to serve as a resource for investigating palindromic variations associated with disease.

## Introduction

**Fig. 1:**
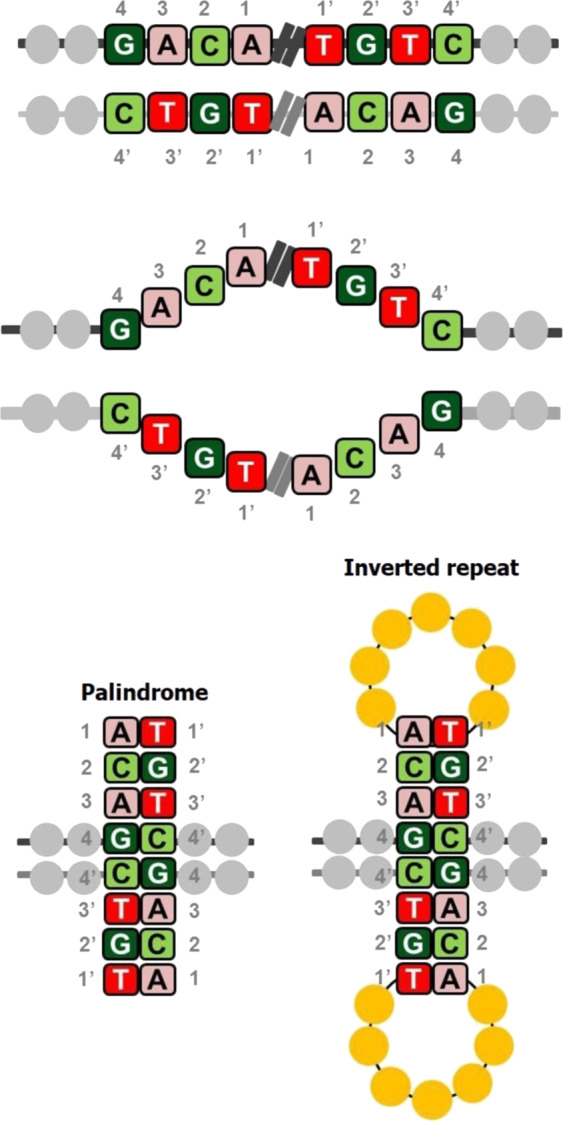
Palindromes and inverted repeats. A sequence (GACA) and its complement in reverse order (TGTC) appearing on the same strand of DNA. When the two halves are separated from each other by a gap sequence, they are simply inverted repeats, and when there is no gap sequence, they are referred to as palindromes. The two halves complement each other when folded, thus leading to a hairpin or cruciform structure.

In DNA, palindromes are defined as a sequence of nucleotides that are followed by its complement sequence appearing in reverse order^1^. For example, as shown in Fig. 1, the sequence on the positive strand 5′-GACA|TGTC-3′ is a palindrome since GACA is followed by its complement CTGT, but appearing in reverse order as TGTC^2^. When a palindromic sequence is folded at its midpoint, the base pairs (bp) on the two halves are complementary. If the complementary portions are separated by a gap sequence, the sequence is referred to as inverse repeat^3^. Sequences that are palindromic except for a few mismatches in base pairing are called *near-palindromes*. Palindromic DNA sequences are prevalent in the genomes of a wide variety of organisms, including humans; the functions and implications of such sequences are only beginning to be understood. Measuring the abundance, frequency, and location of palindromes across the human genome is critical to understanding their functional roles. Palindromes affect various cellular processes, including gene expression, regulation, and gene replication^4^. They stimulate deletions during DNA replication and interchromosomal recombination between homologous sequences, leading to the loss of intervening sequences; in fact, palindromes and near-palindromes account for 83% of deletions and small insertions^4,5^. Short palindromes, under 50 bp, prevent DNA degradation, while palindromes longer than 50 bp result in mutations and DNA instability^6^. A long palindrome has the unique ability to fold back onto itself to form a secondary structure called a cruciform or hairpin (Fig. 1)^7^. The sites of cruciform formation are hot spots for DNA breakage and chromosomal translocation^8^. They can alter the DNA replication process and inhibit gene expression by inhibiting ribosomal translocation along the mRNA transcript^9,10^. Palindromic AT-rich repeats (PATRRs) can cause DNA to denature more readily due to weak A–T bonds, thereby increasing the propensity of the sequence to fold into a secondary structure^11^. Because of this tendency, they are often responsible for chromosomal translocations, recombinations, and deletions and are associated with the inheritance of many genetic diseases. Palindromes and near-palindromes also serve as binding sites for restriction enzymes and transcription factors (TFs). Some examples are TATA box, a core promoter sequence and binding site for transcription-initiating factors^12^; TGACGTCA, the cAMP-responsive element that binds B-ZIP proteins; and binding sites of cancer-associated TFs such as SMAD3/4^13,14^.

### Palindromic variations in disease-associated loci

Palindromic sequences in the genome are essentially “unstable,” rendering them vulnerable to insertions and deletions. The implications of this genomic instability are twofold: one, these regions may act as major hot spots of innovation (“gene nurseries”) and may be retained in the gene pool due to their capacity to drive species evolution through gene creation; two, genomic aberrations, such as insertions and deletions, in these regions may act as causal or modulatory factors of disease^15^. In this work, our focus is on delineating palindromic variations possibly underlying disease-causing mechanisms. Disease-associated loci are either “causal” or “contributory” in nature. Primary disease loci are causal in nature and may be necessary for the manifestation of a disease, whereas susceptibility loci modulate the risk of a person developing a disease while not being the sole factor for disease manifestation^16^. Studies have shown that palindromic and near-palindromic variations underlie both types of disease-associated loci.

### Palindromic variations in causal disease loci

Near-palindromes are linked to many genetic disorders. Slip mispairing in the repeat regions of the dystrophin gene at site Xp21 may cause *Duchenne muscular dystrophy*^17^. The absence of serum cholinesterase activity (*silent serum cholinesterase*), which is essential for the degradation of paralyzing agents during anesthetization, may be caused by the conversion of a near-palindrome to a perfect palindrome by an intrastrand switch mechanism^18^. DNA palindromes are associated with gene amplification. Thus, palindromes are found frequently in cancers such as medulloblastoma, breast cancer, and colorectal adenocarcinoma^19–22^. In HER2-positive breast tumors, the oncogene ERBB2 was shown to be susceptible to a mechanism called palindromic gene amplification, in which genomic segments undergo inverted or palindromic duplication^23^. Short inverted repeats (≤30 bp) are capable of inducing genomic instability, a major driver of carcinogenesis^24^. Both constitutive activation of oncogenes and inactivation of tumor suppressor genes may arise from genomic instability in “fragile” genomic regions that are more susceptible to chromosomal breakage^25^. In human cancers, palindromic sequences may mediate chromosomal translocations, as they can form hairpin structures that undergo double-strand breaks, making them genetically unstable^26,27^.

### Palindromic variations in disease susceptibility loci

Single-nucleotide polymorphisms (SNPs), insertions, and deletions in palindromes contribute to disease susceptibility by causing abnormal gene expression or genomic instability^17^. An SNP in the *PIK3C3* gene, which is strongly associated with schizophrenia and bipolar disorder, converted a 6-base palindrome into an 8-base palindrome that could be recognized and bound by TFs expressed in the brain^28^. Palindromic sequences flanking the multiple sclerosis (MS) susceptibility locus in chr17 undergo segmental duplications and contribute to MS pathogenesis. Moreover, the frequency of palindromes is much higher in the MS susceptibility loci, 17q22 and 17q24.3, compared with other regions in chr17^29^. Molecular findings from the analysis of mitochondrial DNA from the fibroblasts of a child with sideroblastic anemia and proximal tubulopathy showed a single 3.3 kb deletion in 50% of the genome^30^. Palindromic sequences flanking this mutation at deletion breakpoints may contribute to the disease^30^.

Thus, it is important to investigate the mechanistic properties of the genome, such as palindromes and inverted repeats, and their variations in disease versus control populations. High-throughput techniques such as genome-wide analysis of palindrome formation are used to identify these palindromes^22^ but require expertise, intensive effort, and time. These limitations can be circumvented by employing computational methods to identify palindromic sequences from sequenced genomes. A linear-time algorithm to identify palindromic sequences is available, but it can identify only exact palindromes^2^. A prior study on the distribution of palindromes in the human genome was limited and provided insufficient details about the locations of all palindromes. The database associated with the study (HPALDB) is currently inactive^8^.

With the advent of high-throughput technologies for sequencing personal genomes, the computational question can be revisited not only to find the locations of palindromes in the reference genome but also to study the variations exhibited across individuals. We have developed a suite of tools called the Biological Language Modeling Toolkit (BLMT, version 2) for pattern mining in a genome sequence^31^. The tools in BLMT preprocess the genome sequence into a suffix array that is augmented with other data arrays for mining genomic patterns, including palindromes.

Here, we expanded the BLMT suite of tools to carry out a comparative analysis of genomic patterns in a population-wide study. Specifically, we developed these tools for the study of palindromes in the human population using 1000 Genomes (1000G) data^32^. Further, we analyzed disease-associated SNPs in the GWAS (genome-wide association study) Catalog that create, alter, or disrupt palindromes, which will enable the study of the association of palindromes with various diseases. The palindromes and near-palindromes in the human reference genomes presented here may be studied either as genomic annotations along with other annotations, such as SNPs and expression quantitative trait loci (eQTLs), or may be used in disease-specific studies as control data for comparing palindromic variations in patient genomes with palindromes in 1000G data^32,33^.

## Materials and methods

### Data

Human reference genome and whole-genome sequences of 2504 individuals in the 1000G project were analyzed in this work. We collected the phase 3 data of 1000G given in variant call format (VCF), which contain haplotype variant information based on the reference genome. VCF files with integrated information about variants from 2504 individuals were downloaded for all the chromosomes (chr1–22, X, Y) from ftp://ftp-trace.ncbi.nih.gov. We used the release dated 2013-05-02 that uses ShapeIt (https://mathgen.stats.ox.ac.uk/genetics_software/shapeit/shapeit.html) for estimating haplotypes and MVNcall (https://mathgen.stats.ox.ac.uk/genetics_software/mvncall/mvncall.html) for genotype calling and phasing. The human genome build GRCh37/hg19 was used as the reference against which the variants were assembled in 1000G, as indicated in the header of the variant files.

The gene regions, including coding and non-coding regions in each chromosome, were downloaded from the UCSC genome browser using the Ensemble genes track and ensGene table. Upstream regions that are 3000 bp upstream of transcription start sites and downstream regions that are 3000 bp downstream of transcription end sites were downloaded in a similar fashion. In addition, TF-binding sites (TFBS) in the ENCODE chromatin immunoprecipitation-sequencing data (ChIP-Seq) (track: Txn factor ChIP); CpG islands; and regions of non-coding RNA (ncRNA), such as long intergenic ncRNA (lincRNA), small nucleolar RNA (snoRNA) and microRNA (miRNA), were also downloaded from the UCSC genome browser.

### Methods

#### Palindrome computation with BLMT

We employed the BLMT (version 2)^31^ to identify palindromes and near-palindromes in the individual human genomes. BLMT preprocesses each whole-genome sequence into a suffix array and then computes the longest common prefix array and rank array, thus making pattern searches very efficient. BLMT computes palindromes that are perfectly palindromic in the central 8 bases and expands it on both arms until it remains palindromic, but allowing for a user-specified number of mismatches. We set this mismatch tolerance to be four pairs of mismatched bases. The extension is constrained to be of same length on either side (i.e., insertions of unmatched base on only one side is not allowed). The edges of the extended palindromes must be complementary; mismatched bases at the end are deleted. We first analyzed the lengths and locations of the palindromic data in various genomic regions of the reference genome (GRCh37/hg19 build). To account for palindromes that span different types of overlapping regions, we consider intronic regions that do not overlap with exons, upstream regions that may also be intronic regions, upstream regions that may also be exonic regions, and exclusively upstream regions.

Then, the whole genomes corresponding to each of the individuals in 1000G are assembled by incorporating the corresponding variants into the reference genome using a software program that we developed, and the palindromes are computed for that individual by applying BLMT. In this study, we did not address structural variants, and incorporated only SNPs, insertions, and deletions.

#### Aligning palindromes in personal genomes to the reference genome

To understand how palindromes vary due to the presence of variants in individuals, we computationally aligned and mapped the positions of palindromes in an individual’s genome to their corresponding position in the reference genome by computing the cumulative offset of any given position of the individual genome based on the changes in length to DNA caused by insertions and deletions. For example, the insertion of a 2 bp sequence would introduce an offset of −2 (i.e., the corresponding position in the reference genome would be two positions to the left), while the deletion of a 10 bp sequence would introduce an offset of +10. The offset at a particular genomic location is the sum of offsets introduced by every variant until that location. By mapping the midpoint of a palindrome in an individual back to its corresponding location in the reference genome and checking whether this location is the midpoint of a reference genome palindrome, we can determine if a palindrome has remained intact. However, its length may have been altered to be shorter or longer. If the mapped location is not the midpoint of any reference genome palindrome, then it is a new palindrome. Similarly, it is possible to map all reference genome palindromes to the palindromes of an individual to determine whether any palindromes have disappeared. Some of the disappearances in personal genomes may be attributed to our definition of a palindrome; that is, a sequence is considered perfectly palindromic in the central 8 bases; a palindrome may have disappeared or a new one formed due the presence of an SNP within these central 8 bases. To address this, we introduced the idea of a “near-palindrome,” which is a palindrome that tolerates 1 mismatch in the central 8 bases and 4 mismatches outside of the central 8. Before concluding that a palindrome has either disappeared or appeared, we first determine if it is a near-palindrome in the reference or individual genome.

It is thus possible to catalog the nature of every palindromic change that occurs within each individual in 1000G.

#### Cataloging palindromic changes across individuals

**Fig. 2:**
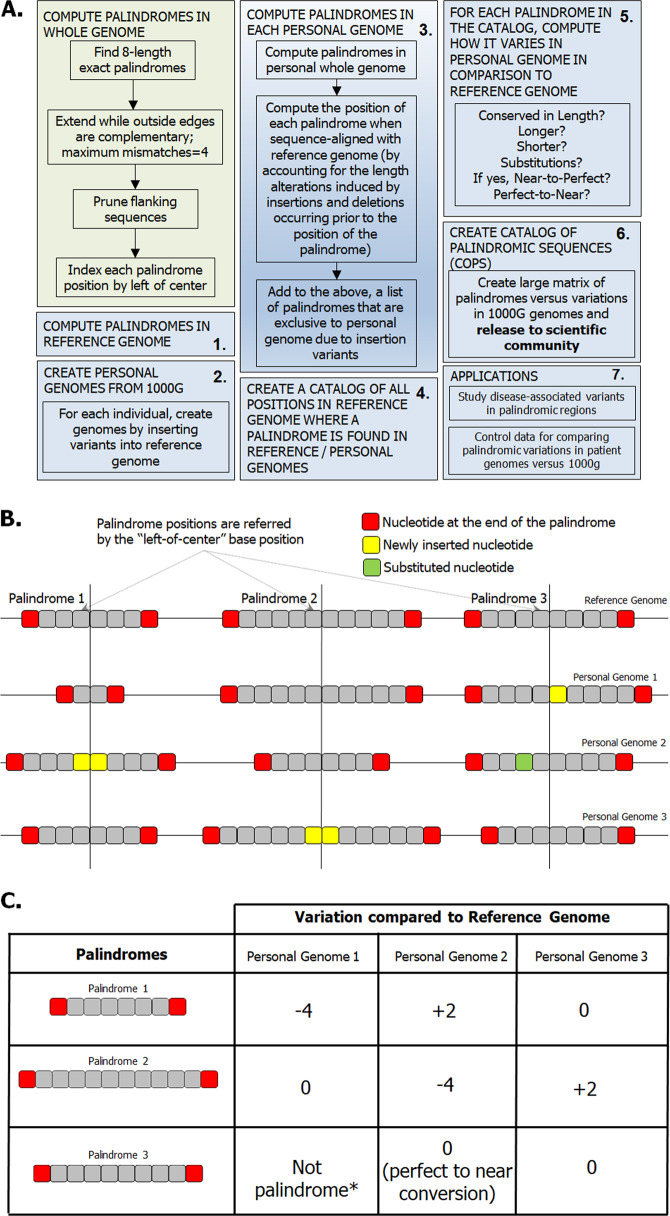
Methodology to identify and catalog palindromic variations in personal genomes. **a** The steps involved in computing palindromes in personal genomes, identifying and cataloging palindromic variations with respect to the reference genome, and applications of the methodology are shown in the schematic diagram. Further, the specific methods that were followed to **b** align palindromes in the personal genomes to those in the reference genome and **c** catalog palindromic changes across individuals are shown. *The newly inserted nucleotide creates a gap between the complementary halves and fails to create a palindrome that fits our definition.

We computed a matrix where each row (*i*) corresponds to a palindrome that occurs in any of the 1000G individual genomes, each column (*j*) corresponds to an individual and each entry in the matrix corresponds to how a palindrome *i* changed in individual *j* compared to that in the reference genome. This matrix can help understand which palindromes tend to be conserved across the population versus those that exhibit variations. This information will be particularly useful when analyzing palindromes in the context of tumor genomes^33^; here, a palindromic change is interesting if abundant in the tumor population but not in the healthy 1000G population. Figure 2 is a schematic diagram of the methodology and its applications.

### Computational infrastructure

We computed the palindromes on a compute cluster with 16 machines (nodes), each with 16 CPUs (Intel E5-2660 v3 Haswell) with 256 GB of RAM. Jobs were submitted to compute palindromes for each individual using the Slurm Workload Manager (http://slurm.schedmd.com/). Approximately 200 jobs were run in parallel.

## Results

### Palindromes in the reference genome

Palindrome computations were carried out using the Biological Language Modeling Toolkit (version 2)^31^. We computed the number of palindromes in the reference genome (GRCh37 build) across all chromosomes, except mitochondrial chromosomes. BLMT finds all occurrences of perfectly palindromic 8-base-long sequences and then extends the span of each of them, as long as the bases on either side are complementary but allowing up to 4 mismatches during this extension. We found that the number of palindromes is proportional to the length of the chromosomes (Supplementary Fig. 1a), that chromosomes 4 and X showed greater density, and that chromosomes 22 and Y showed lower density, as indicated by the normalized counts per 1000 bp (Supplementary Fig. 1b). When the distribution of the palindromes in the human genome was inspected in terms of both their location and length, we discovered that they are not distributed uniformly. Palindromes 8 and 20 bp in length occur most frequently, and a high concentration of palindromes can be found in intronic and intergenic regions. The genome contains 13,267,316 palindromes up to 40 bp long and 182,437 palindromes longer than 40 bp.

**Fig. 3:**
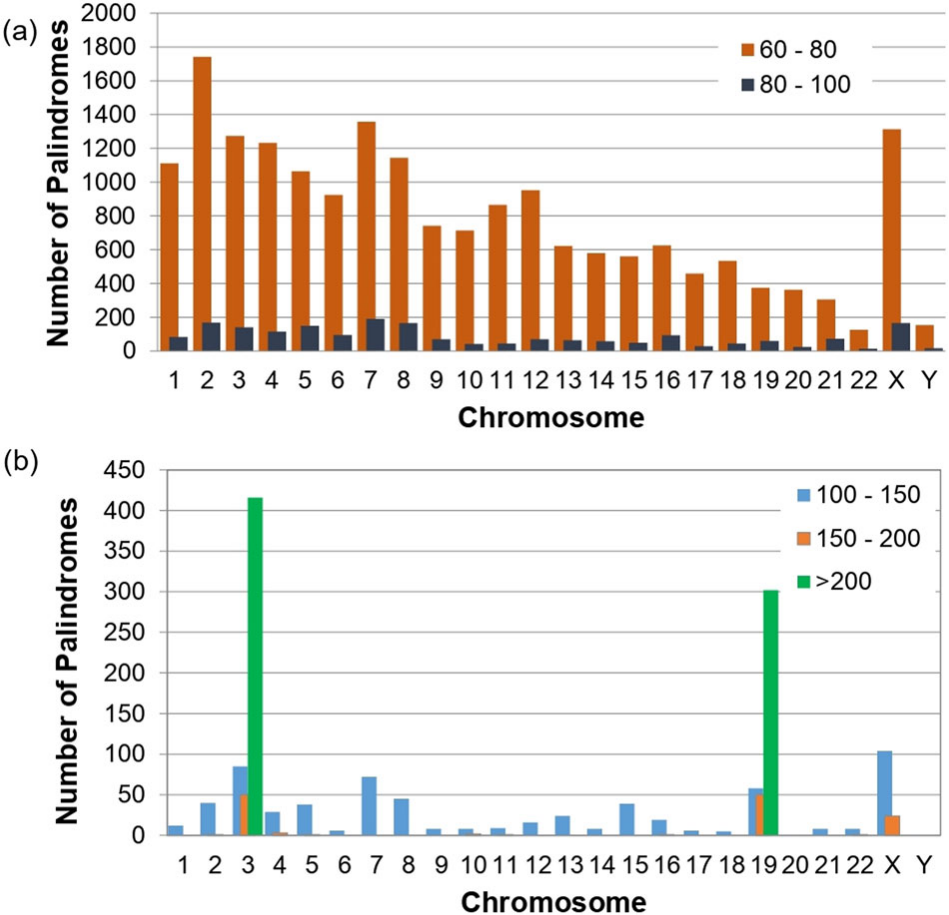
Distribution of palindromes across all chromosomes by length. **a** Frequency of palindromes from 60 to 100 bp in length is shown. On average, 797 palindromes with a length between 60 and 80 bp and 84 palindromes between 80 and 100 bp were found in each chromosome. **b** Frequency of palindromes with lengths from 100 to 200 and >200 bp is shown. While most chromosomes contain palindromes of length 100–150 bp, only chromosomes 3 and 19 have very long palindromes (>200 bp).

There are a total of 69,888 palindromes longer than 50 bp, with 783 that are 100–200 bp long and 718 palindromes longer than 200 bp (Fig. 3 and Supplementary Table 1). Chromosome 3 has the longest palindrome, which spans 618 bp, whereas chromosomes 19 and 3 have 410 and 551 palindromes longer than 100 bp, respectively (Fig. 3). We computed the palindromes in various regions of the reference genome, such as exons, introns, intergenic regions, and upstream regions (3000 bp), and ascertained that 56% of these palindromes lie within gene regions across all chromosomes. Our results show that palindromes tend to be highly concentrated in introns and intergenic regions, and 126,900 palindromes in coding exons (Supplementary Fig. 2). A detailed analysis of the number of palindromes in TFBS, CpG islands, and ncRNA, such as lincRNA and sno/miRNA, revealed that 70.7% of the TFBS in the genome had at least one palindrome. The details of the number of palindromes in various regions of the reference genome are shown in Supplementary Table 2.

### Palindromes in 1000Gs

The 1000G project provides genomic variants for 2504 individuals in relation to the reference genome (GRCh37 build) in VCF. We constructed a personal genome for each individual and then computed the palindromes occurring in that genome. Each palindrome was then indexed in relation to its aligned position in the reference genome. We constructed a catalog of all the palindromes that occur in any of the 1000Gs, as well as how each of these palindromes varied in the 2504 individuals. A sample of this palindrome variants matrix is shown in Fig. 2. The entire matrix is presented in Supplementary Table 3.

**Fig. 4:**
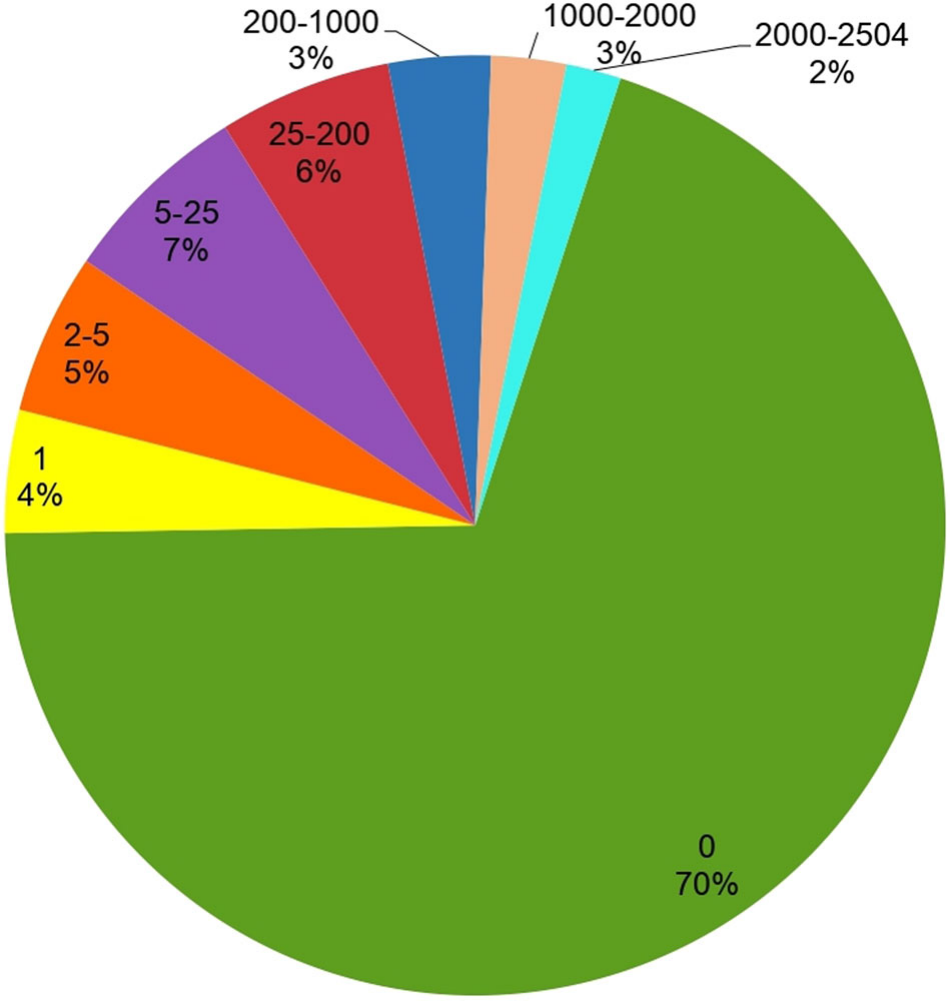
Conservation of palindromes across different individuals using 1000 Genomes. Each slice of the pie chart represents the fraction of the reference genome palindromes with a specific number of variations (approximately). For example, 7% of the palindromes varied in 5–25 individuals (purple slice); 70% of the palindromes did not vary in any individual (green slice).

**Fig. 5:**
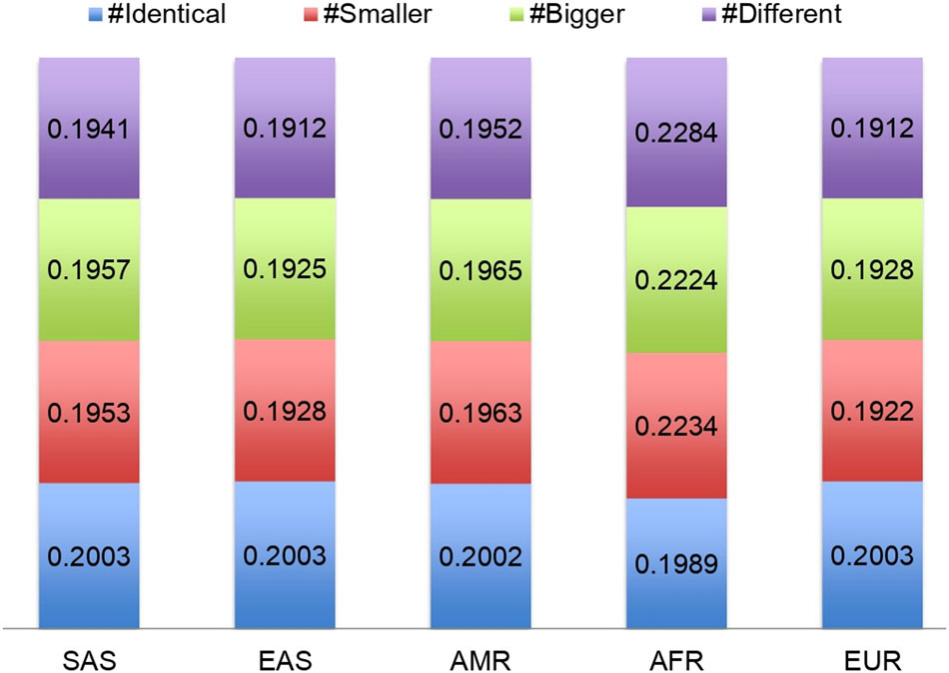
Palindrome variation across different subpopulations in 1000 Genomes. The subpopulations include South Asian (SAS), East Asian (EAS), American (AMR), African (AFR), and European (EUR).

From these data, we analyzed the extent to which the palindromes are conserved across the personal genomes in 1000G and discerned that ~70% of the reference genome palindromes remain completely unchanged across the 2504 individuals. Four percent of the palindromes exhibited changes in only one individual studied, 5% in 2–5 individuals, 7% in 5–25 individuals, 6% in 25–200 individuals, and 3% in 200–2000 individuals (Fig. 4). A summary of the variants within the palindromic regions across all the samples is presented in Supplementary Table 4. Palindrome *conservation* across the 1000G samples is shown in Fig. 4. On an average, 95.8% of an individual’s palindromes were identical to those in the reference genome, 1.9% were shorter, while 0.842% were longer, 1.46% were new palindromes caused by variants, and 0.05% were found in insertions. The major populations represented include South Asian (SAS), East Asian (EAS), American (AMR), African (AFR), and European (EUR). The African population in 1000G had the highest number of altered palindromes, which is expected because of the larger number of variants found in this population^34^. Palindrome *variation* across various populations in 1000G is shown in Fig. 5.

### Palindromes in the personal genomes formed by disease-associated variants

**Fig. 6:**
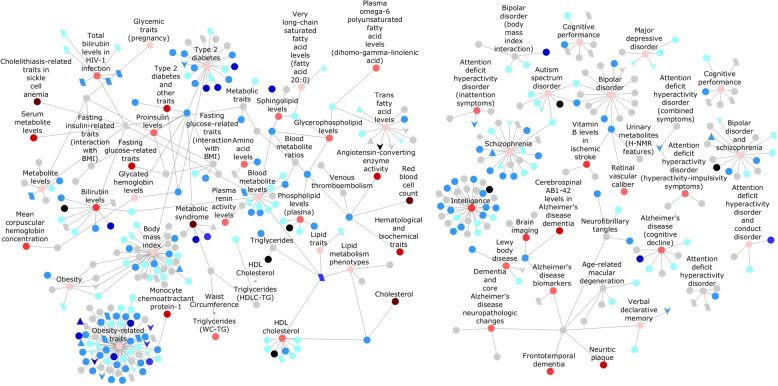
Network of palindrome-associated GWAS SNPs and their connection to various diseases. Diseases that are associated with GWAS SNPs with palindrome changes in 1000 Genomes are shown as round nodes with labels. Their size represents the overall number of SNPs associated with that disease. Their color represents the percentage of their SNPs that are palindrome altering: gray (0–8%), light pink (8–12%), medium pink (12–25%), light red (25–50), medium red (50–75%), and dark red (75–100%). For example, the trait “height” has more SNPs associated with it compared to “multiple sclerosis” (node size); however, a larger percentage of multiple sclerosis SNPs are palindrome-altering (node color). The specific SNPs that alter palindromes are shown as unlabeled nodes, in which SNPs that convert a palindrome to a non-palindrome are shown as diamond-shaped nodes; SNPs that create new palindromes are shown as parallelogram-shaped nodes; SNPs that lengthen the palindromes are shown as triangle-shaped nodes; SNPs that shorten the palindromes are shown as v-shaped nodes; and SNPs that alter palindromes in other ways (non-identical, near-to-perfect or perfect-to-near) are shown as round-shaped nodes. These nodes that represent SNPs are colored by their RegulomeDB scores: black (score = 1), dark blue (2), medium blue (3), light blue (4 and 5), very light blue (6), and gray (0). The network diagram was made using Cytoscape^50^.

The GWAS Catalog is a list of SNPs that have statistically significant associations with specific diseases or traits, as curated from the published literature^35^. To understand the association of palindromes with a disease, we studied whether any of the SNPs in the GWAS Catalog were in 1000G palindromes. The process was made simple as both data sources use reference SNP identifiers to describe variants; <5% of the GWAS Catalog variants were not found among the 1000G variants. To determine whether palindromic regions with variants are more susceptible to disease than other genomic locations, we compared the expected and observed probabilities of variants in palindromic locations. GWAS variants occurred in palindromic regions 14-fold more than expected. A large number of palindrome-altering GWAS SNPs were those that are associated with diabetes (53 SNPs) and obesity-related traits (44 SNPs). Fourteen SNPs were associated with Alzheimer’s disease, 10 SNPs with attention deficit hyperactivity disorder, 14 with schizophrenia, 16 with Crohn’s disease, and several SNPs were associated with various cancers. We also checked whether any of the GWAS SNPs resulted in the formation of new palindromes (i.e., the sequences that were either non-palindromic or near-palindromic in the reference genome, but became a perfect palindrome in the individual genome because of an SNP). Approximately 760 GWAS SNPs led to a gain of palindrome in 1000G. Of these, 41 SNPs that are associated with various diseases, and formed new palindromes, were found in fewer than 100 samples (with two alleles or samples per individual). Some of the disease-associated SNPs, such as those of type 2 diabetes, ovarian cancer, breast cancer, and schizophrenia, have low allele count in 1000G as expected, as these individuals were from healthy populations. Some individual SNPs that caused sequences to be perfectly palindromic were in genes associated with multiple diseases such as PVLR2 and PTPN22. An analysis of a few SNPs with palindrome associations is shown in Fig. 6, and the detailed analysis of the palindromes in 1000G, their associated GWAS SNPs, palindrome changes, allele counts, genes, and disease annotations can be found in Supplementary Fig. 3 and Supplementary Table 5.

We further analyzed the palindromes that are associated with SNPs present in regulatory regions using RegulomeDB, which annotates SNPs with regulatory elements, including in regions with DNase hypersensitivity sites, TFBS, promoter regions, and so on, and ranks the SNPs by their significance score^36^. We found that 46 SNPs in palindromes were likely to affect the binding of proteins and were linked to the expression of gene targets (scores 1a–1f). Of these SNPs, five were associated with obesity and diabetes, and three were associated with mental disorders. Ninety-four SNPs likely to affect the binding of proteins (scores 2a–3b)^36^ also occurred in palindromes, as shown in Fig. 6 and Supplementary Fig. 3.

## Discussion

Recent research has shown that palindromes may be critical to several cellular processes, including transcription, replication, and DNA recombination^37^. Therefore, it is important to study palindrome distribution in the genome to understand their functions and disease associations. Palindromes are abundantly present in the human genome, but their distribution is non-uniform. This distribution can be correlated with their participation in important biological functions. The palindrome lengths also vary greatly in the genome. In general, shorter palindromes are expected to be more abundant than longer palindromes, and both short and long palindromes have been implicated in genomic instability^7^.

The frequency and distribution, from our analysis, of both long and short palindromes of varying lengths in each chromosome are shown in Supplementary Fig. 1 and Supplementary Table 1. We observed that palindromes with lengths between 8 and 20 bp were the most frequent, and chromosomes 3 and 19 had a high number of long palindromes (>200 bp). Long palindromes form secondary structures that act as hot spots for genomic rearrangements and translocations^8^, and are known to participate in as many as 30% of integration events in the human DNA^38^. These palindromes constitute fragile sites, are correlated with breakage and deletion, and are associated with diseases^7^. Our analysis revealed the presence of a large number of palindromes in both the reference genome (GRCh37/hg19 build) and in 1000G. According to our results, the very long palindromes were mostly AT repeats.

### AT richness of palindromes

We defined the AT richness of a palindrome using the following formula:

$${\mathrm{AT}} {\mbox{-}} {\mathrm{richness}}\,{\mathrm{percentage}} = \frac{{\left( A + T \right) \times 100}}{{A + T + C + G}},$$

where *A*/*T*/*C*/*G* represent the number of respective bases present in the palindrome.

AT-rich palindromes are those with an AT-richness percentage of ≥50. Other palindromes are referred to as CG-rich. Eighty percent of the palindromes had an AT content of 80–90%, whereas only 2% of the palindromes had CG content >80%. The analysis of AT- and GC-rich palindromes is shown in Supplementary Table 1. PATRRs are sites frequently associated with double-strand breakage and hairpin or cruciform DNA formation that lead to translocations and recombinations^11^. We found that the longest palindromes were AT-rich.

### Palindromes in functional regions

The distribution of palindromes in various genomic regions, such as exons, introns, and regulatory regions, including TFBS, CpG islands, and ncRNA regions, is shown in Supplementary Fig. 2 and Supplementary Table 1. Regulatory regions contain promoters and enhancers, and palindromic sequences in these regions are known to serve as TFBS for regulating gene expression. For example, palindromic sequences were found in promoter regions that may be binding sites for TFs, such as CREB, USF, and NRF-1, providing further support for their role in gene regulation^12^.

As a preliminary study to understand the association of palindromes with diseases, we analyzed the GWAS Catalog SNPs for their influence on the nature of the palindromes, that is, whether they altered the palindromes to make them longer/shorter, or near/perfect. We found that, overall, disease-associated risk variants (GWAS SNPs) were 14 times more likely to be present in palindromic regions than expected. Many diseases/traits are associated with SNPs that cause palindrome changes. For example, 7% or 62 obesity-related trait SNPs, 15% or 30 Crohn’s disease-associated SNPs, and 50% or 28 intelligence-associated SNPs induced palindrome changes; these variants were also found in 1000G (Supplementary Table 6).

Using the eQTL calculator from GTEx^39^, we tested whether any of the SNPs in palindromic regions associated with six diseases — diabetes, rheumatoid arthritis (RA), schizophrenia, Alzheimer’s disease, breast cancer, coronary heart disease — affected the expression of the genes to which they had been mapped. This association was tested in tissues relevant to the diseases. Of the 15 SNPs that were significant eQTLs in any of these 6 diseases (Supplementary Table 7), 14 were intronic variants, and 1 was a downstream gene variant (rs610932 mapped to MS4A6A, Alzheimer’s disease). Two of these intronic variants (rs3825932, CTSH, diabetes; rs4239702, CD40, RA) overlapped with regions called “retained introns.” These introns are retained during transcription and introduce premature stop codons into mRNA, leading to erroneous gene expression.

When analyzing palindrome-altering GWAS variants with low allele counts (AC < 100) in 1000G (which represents a healthy population), we found one SNP (rs11571833) that is associated with both breast and lung cancers in the BRCA2 gene (with AC = 22). Similarly, an intronic SNP (AC = 15) that is associated with ovarian cancer formed a new palindrome in *BRIP1*, a gene encoding the *BRCA1-interacting protein*, required for BRCA1-mediated DNA repair^40^. In our previous pilot study of palindrome alterations by breast cancer-associated variants in The Cancer Genome Atlas (TCGA), we found that many palindrome changes were associated with oncogenes and breast cancer genes^33^. Of all the palindromes that showed any variation in cancer genomes (matched normal and tumor samples), 38% of what was near breast cancer genes happened to be the most differentiated palindromes in tumor samples. The palindromes that are associated with oncogenes, such as RAD21, NBN and KMT2A, were found to have changed significantly in the tumor samples. In addition, we observed that the palindromes that were associated with oncogene NUP98 were completely absent in tumors. These results further support the possible role of palindromes in various diseases, including cancer.

We also identified the individual SNPs in or near palindromes that are associated with multiple diseases or traits. An SNP (rs6857) that is present in the palindromic region of *NECTIN2* (alias *PVRL2*) gene is associated with many diseases that are related to neuronal functions. Examples include macular degeneration, Alzheimer’s disease, memory, and frontotemporal dementia. Interestingly, this SNP is present in the 3’UTR region, leading to the formation of a perfect palindrome in 553 individuals in 1000G. The 3′-untranslated region (UTR) region is involved in gene regulation and influences polyadenylation, mRNA stability, and translation. It also contains binding sites for transcriptional regulators such as miRNA^41^. miRNAs regulate neurogenesis and brain development. Hence, palindrome changes in UTR regions may affect miRNA binding, leading to disease progression. Further, due to the presence of a missense variant in *PTPN22* gene that is associated with multiple diseases, such as Crohn’s disease, diabetes, and RA, a non-palindromic sequence became perfectly palindromic in 1000G.

**Fig. 7:**
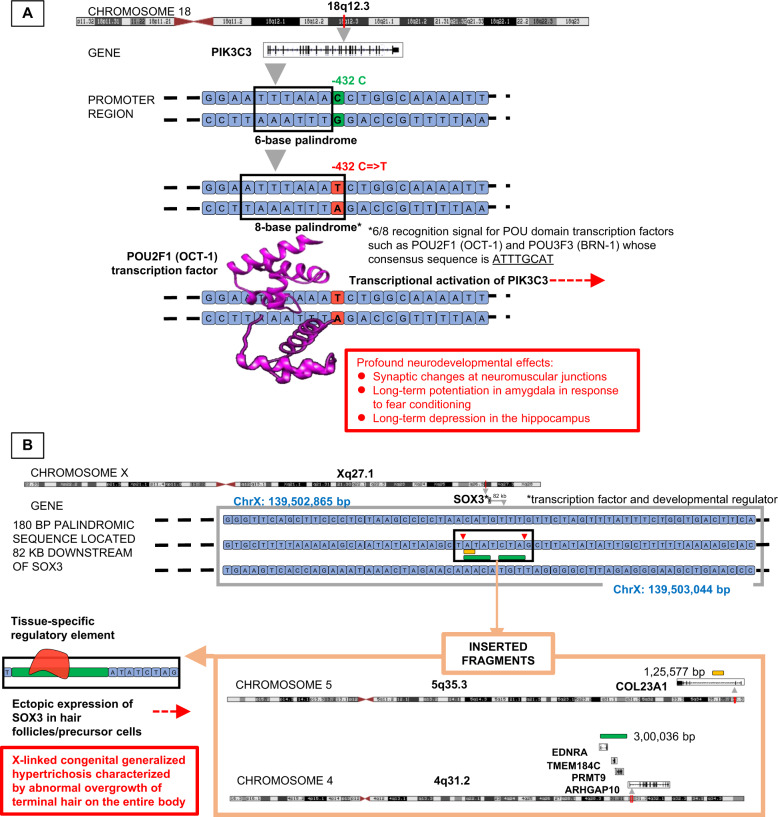
Illustrative examples of palindrome-mediated mechanisms of disease, as indicated in the literature. **a** In certain subsets of bipolar disorder and schizophrenia patients, a mutation in the promoter region of PIK3C3 (−432C -> T) extends a 6-base palindrome (“TTTAAA”) into an 8-base palindrome (“ATTTAAAT”), which also acts as a 6/8 recognition sequence for POU domain transcription factors such as POU2F1 (OCT-1) and POU3F3 (BRN-1), whose consensus sequence is “ATTTGCAT”^51^. These transcription factors are regulators of brain development. Binding of POU domain transcription factors to the palindromic sequence may lead to the transcriptional activation of PIK3C3 and PIK3C3-mediated neurodevelopmental changes. **b** X-linked congenital generalized hypertrichosis is a rare genetic condition characterized by hair overgrowth over the entire body. In families in which this condition is segregated, chromosomal breakpoints are observed in a 180-base palindromic sequence located 82 kb downstream of the *SOX3* gene on Xq27.1^52^. SOX (SRY-related HMG-box) transcription factors are regulators of embryonic development. The 180-base palindromic sequence mediates an interchromosomal insertion of either a 125,577 bp fragment from COL23A1 of 5q35.3 or a 300,036 bp fragment from 4q31.2 (including the genes *PRMT9* and *TMEM184C* and sections of *EDNRA* and *ARHGAP10*) into these breaks. New regulatory elements may be introduced with the insertion of these fragments. It has been conjectured that, as a result of these new elements, *SOX3* may be ectopically expressed in hair follicles or precursor cells during the early stages of hair follicle development. Structures of chromosomes 18 and X, and gene structures of *PIK3C3*, *SOX3*, *COL23A1*, *PRMT9*, *TMEM184C*, *EDNRA*, and *ARHGAP10* were taken from UCSC Genome Browser (https://genome.ucsc.edu/)^53^. The images were produced based on the GRCh38 (hg38) assembly. The protein structure of POU2F1^54^ (PDB ID: 1OCT) was downloaded from RCSB PDB^55^. The image of POU2F1 was created using UCSF Chimera^56^.

We then reviewed the palindrome sequences that have SNPs with significant regulomeDB scores. We learned that one of the SNPs (rs7386474), which is associated with bipolar disorder and schizophrenia, is a binding site for FOXP2 protein, a TF playing a significant role in these mental illnesses^42^. Another palindrome-altering SNP (rs2535629) associated with autism spectrum disorder and other mental disorders^43^ was present in the intron of *ITIH3* gene and is bound by several proteins such as FOS, MYC, CTCF, RAD21, SMC3, and ZNF143. These results further support the role of palindromes in diseases since these SNPs lead to palindrome changes that may affect the binding of TFs, hinting at a possible mechanism for disease pathogenesis. A missense variant (A/G) in rs2476601 on chr1, mapped to the gene *PTPN22*, was associated with the formation of a new palindrome. In a study, this SNP, which is associated with RA, was identified as a “functional SNP” modulating the binding of TFs^44^. The least frequent allele (minor allele) in 1000G in this case was “A” (frequency = 0.027). This allele had a frequency of 0.09 in a particular RA cohort and was linked to RA (*p* value = 9E − 170)^45^. Compared with the controls, marked downregulation of PTPN22 expression was observed in the RA patients carrying this risk allele (*p* value = 7E − 03)^46^. RegulomeDB assigned a score of “2b” to rs2476601, indicating that “protein binding is likely to get affected.” From the ENCODE ChIP-Seq data, the two TFs that bind within this region (chr1:114377420–chr1:114377736) were FOS and STAT3, both of which have been linked to RA. Figure 7 provides illustrative examples of palindrome-mediated mechanisms of disease, as indicated in the literature.

We believe that these results will help researchers to understand palindrome distribution and conservation across various populations. These results will also help to identify individual palindromes that undergo rearrangements due to the presence of variants such as SNPs that could affect various cellular processes leading to gene dysregulation and disease pathogenesis.

### The catalog of palindromic sequences (COPS)

The COPS will serve as a resource to investigate palindromic variations in genomics studies of diseases. Specifically, COPS can serve as control data for the comparison of palindrome variations in patient genomes with the palindromes in 1000G. This was demonstrated in our pilot study on TCGA data in which we compared palindromes in matched tumor and normal pairs of genomes with the 1000G data presented in COPS^33^.

We are making available the location and length of every palindrome that appears in the reference genome or the 1000G genomes and its variation in each of the 2504 individual genomes with respect to the reference genome. In addition to the individual occurrences of palindromes, aggregated results are presented to show the distribution in coding and non-coding regions, palindrome conservation across the genomes, the presence of rare and common variants within the palindromes, and the GWAS SNPs that are associated with palindromic changes for various diseases.

## Limitations

During genome sequencing, DNA that is to be cloned is inserted into bacterial artificial chromosomes (BACs), which are used for transforming *Escherichia coli*, a process by which foreign DNA is introduced into a bacterial cell. Regions that are highly susceptible to genomic rearrangements, namely, palindromes, duplicated segments, and satellite DNA, might be deleted during transformation and cloning in *E. coli*. As a result, these sequences may be underrepresented in the reference genome that was sequenced using this technology^47^. Thus, the palindrome computation in this work is limited by the incomplete nature of the reference genome.

Our work is based on the human reference genome build GRCh37 (hg19). The primary reason for this choice was that the 1000G was assembled with hg19 as the reference. Hg19 was also annotated in more detail than the later build GRCh38 (hg38), at the time of our computations. Although sequence gaps and misassembly have been reported in hg19^48^, ~99% of the SNPs in hg38 can be retrieved from hg19^49^. Users wishing to study palindromes in the context of hg38 may convert the coordinates by uploading BED files into the “UCSC liftOver tool.” This tool is available through a web interface and as a standalone program.

### Future directions

We plan to extend this work to compute and analyze inverted repeats (i.e., palindromic sequences with a gap sequence between the two halves), which are known to be genetically unstable hot spots in cancer genomes. These studies will enhance the knowledge of palindrome functions in the genome and their contribution to human diseases, and highlight the mechanism by which DNA variants play a role in disease.

## Supplementary information

Supplementary Table 1

Supplementary Table 2

Supplementary Table 3

Supplementary Table 4

Supplementary Table 5

Supplementary Table 6

Supplementary Figure 1

Supplementary Figure 2

Supplementary Figure 3

Supplementary Table 7
