# Supplementary material for: A reference catalog of DNA palindromes in the human genome and their variations in 1000 Genomes
Source: Hum Genome Var. 2020 Nov 20;7:40. doi: 10.1038/s41439-020-00127-5 (PMC7680136; doi:10.1038/s41439-020-00127-5)
Supplement: Supplementary file 1 — Supplementary Table 1 [file 41439_2020_127_MOESM1_ESM.pdf]

Palindromes in different Chromosomes

Chromosome	Palindrome Count	Length	Normalized Palindrome Count
1	1,015,470	249,250,621	4.07
2	1,142,820	243,199,373	4.70
3	938,926	198,022,430	4.74
4	971,349	191,154,276	5.08
5	870,183	180,915,260	4.81
6	810,885	171,115,067	4.74
7	733,663	159,138,663	4.61
8	685,065	146,364,022	4.68
9	552,417	141,213,431	3.91
10	602,026	135,534,747	4.44
11	600,275	135,006,516	4.45
12	610,037	133,851,895	4.56
13	495,191	115,169,878	4.30
14	414,580	107,349,540	3.86
15	363,204	102,531,392	3.54
16	332,040	90,354,753	3.67
17	319,484	81,195,210	3.93
18	364,294	78,077,248	4.67
19	213,440	59,128,983	3.61
20	252,059	63,025,520	4.00
21	172,416	48,129,895	3.58
22	136,966	51,304,566	2.67
X	741,731	155,270,560	4.78
Y	111,232	59,373,566	1.87

Ditribution of Palindromes by Length

Chromosome	<20 bp	20 - 40 bp	40 - 60 bp	60 - 80 bp	80 - 100 bp	100 - 150 bp	150 - 200 bp	>200 bp
1	792,701	210,890	10,672	1,112	83	12	0	0
2	884,917	242,689	13,263	1,742	168	40	1	0
3	727,141	198,950	10,871	1,273	140	85	50	416
4	741,765	216,137	12,067	1,232	116	29	3	0
5	671,167	187,740	10,023	1,065	149	38	1	0
6	627,285	173,397	9,177	925	95	6	0	0
7	566,459	156,933	8,652	1,357	190	72	0	0
8	529,953	145,269	8,489	1,144	165	45	0	0
9	430,154	115,408	6,035	742	70	8	0	0
10	468,972	125,658	6,630	714	42	8	2	0
11	467,056	125,060	7,238	866	45	9	1	0
12	472,263	129,641	7,096	952	69	16	0	0
13	378,493	109,654	6,334	623	63	24	0	0
14	321,489	87,483	4,962	580	58	8	0	0
15	283,528	75,134	3,894	560	49	39	0	0
16	258,196	68,919	4,187	625	93	19	1	0
17	249,074	65,829	4,089	458	28	6	0	0
18	281,630	77,924	4,157	534	44	5	0	0
19	165,179	44,738	2,680	374	59	58	50	302
20	198,050	50,749	2,872	363	24	1	0	0
21	133,097	37,151	1,783	305	72	8	0	0
22	108,072	27,317	1,430	125	13	8	1	0
X	565,244	163,267	11,613	1,313	166	104	24	0
Y	85,401	24,093	1,566	154	17	1	0	0

AT - GC richness

Chromosome	AT %	GC %
1	71.22	28.78
2	73.14	26.86
3	73.96	26.04
4	75.49	24.51
5	73.89	26.11
6	73.74	26.26
7	72.84	27.16
8	73.21	26.79
9	71.52	28.48
10	71.38	28.62
11	71.33	28.67
12	72.68	27.32
13	75.28	24.72
14	72.36	27.64
15	70.66	29.34
16	67.60	32.40
17	67.01	32.99
18	73.70	26.30
19	64.56	35.44
20	67.90	32.10
21	72.61	27.39
22	62.43	37.57
X	74.34	25.66
Y	74.14	25.86

Percentage of Palindromes by % AT richness

Chromosome	<20%	20 - 30%	30 - 40%	40 - 50%	50 - 60%	60 - 70%	70 - 80%	80 - 90%	90 - 100%
1	18,144	46,672	52,777	129,715	83,153	115,580	214,694	147,196	207,539
2	14,633	41,738	48,636	131,209	89,993	133,212	255,637	179,699	248,063
3	10,089	30,618	36,113	105,007	75,155	110,993	213,282	151,880	205,789
4	8,459	25,117	29,198	93,430	71,606	112,707	227,481	171,536	231,815
5	9,333	28,050	32,428	95,621	68,420	102,895	198,454	142,503	192,479
6	8,877	26,477	30,980	89,320	63,821	95,577	184,460	132,088	179,285
7	11,467	29,569	33,086	85,191	56,633	83,062	160,272	114,506	159,877
8	8,708	24,832	28,614	79,543	55,172	80,098	152,417	106,753	148,928
9	10,065	24,954	27,259	67,545	44,718	63,513	119,769	82,310	112,284
10	10,058	27,158	31,463	77,046	49,536	68,891	127,258	87,669	122,947
11	10,898	28,056	30,528	75,715	49,690	68,880	128,082	88,054	120,372
12	8,976	23,918	27,034	72,495	48,559	70,167	133,304	93,700	131,884
13	4,634	13,872	16,296	48,360	35,029	56,859	115,405	87,016	117,720
14	6,681	16,992	19,195	48,906	32,893	47,451	90,383	64,350	87,729
15	6,897	18,025	20,485	48,341	30,006	40,932	75,532	50,724	72,262
16	10,626	23,443	24,532	50,051	27,819	35,162	59,518	39,381	61,508
17	11,434	24,889	25,464	48,112	25,589	31,290	55,374	37,382	59,950
18	4,352	12,163	14,134	40,527	28,437	42,735	82,374	59,080	80,492
19	12,148	22,389	21,455	35,399	16,318	17,183	29,961	20,443	38,144
20	6,649	16,408	17,799	38,339	21,621	27,824	47,309	30,836	45,274
21	3,063	7,636	8,151	19,323	12,630	19,165	36,930	27,527	37,991
22	6,662	15,028	14,344	23,513	10,771	12,046	19,856	13,079	21,667
X	6,794	22,495	26,387	82,736	61,533	89,786	167,893	118,563	165,544
Y	748	3,112	3,886	13,356	9,759	13,666	24,764	17,333	24,608
