# Supplementary material for: A reference catalog of DNA palindromes in the human genome and their variations in 1000 Genomes
Source: Hum Genome Var. 2020 Nov 20;7:40. doi: 10.1038/s41439-020-00127-5 (PMC7680136; doi:10.1038/s41439-020-00127-5)
Supplement: Supplementary file 2 — Supplementary Table 2 [file 41439_2020_127_MOESM2_ESM.pdf]

Distribution of Palindromes in Different Regions

Chromosome	Exons	Introns	Upstream	Introns (that are not exons)	Upstream + Exon	Upstream + Intron	Exclusively Upstream	Crossover	Intergenic	Coding Exons	UTR
1	46,461	542,660	106,941	523,284	40,564	95,159	34,825	1,628	409,272	12,522	39,319
2	35,752	601,948	86,391	584,974	30,418	77,957	26,595	1,172	494,363	8,310	31,010
3	29,081	537,186	72,324	523,040	22,196	65,046	19,154	841	366,810	6,773	25,803
4	25,089	464,994	60,312	453,984	22,766	55,447	20,455	1,134	470,687	6,493	21,576
5	25,110	432,497	62,645	420,656	22,817	56,980	20,382	754	403,281	5,557	22,087
6	24,482	374,422	58,203	364,227	25,192	53,068	22,621	783	398,772	5,977	20,678
7	24,634	409,200	61,032	397,897	22,487	54,980	19,698	754	290,680	5,837	21,499
8	20,216	375,264	53,245	365,652	18,505	48,140	16,305	613	282,279	4,342	17,954
9	19,372	247,475	45,288	240,053	19,881	40,877	17,516	700	274,776	5,237	16,128
10	19,329	313,174	47,421	305,394	18,241	43,387	16,228	672	260,403	4,878	16,135
11	27,025	327,418	67,642	313,973	25,421	59,227	21,826	766	236,685	7,392	23,099
12	25,471	342,957	64,836	330,163	20,081	57,302	16,865	719	236,819	6,306	22,240
13	9,610	187,158	25,229	183,771	10,981	23,505	10,179	409	291,222	2,250	8,110
14	16,130	229,886	44,232	221,658	15,834	39,286	13,859	461	162,472	3,956	14,083
15	17,885	237,556	44,912	228,224	13,218	39,732	11,022	520	105,553	4,203	15,763
16	20,703	187,554	48,469	176,671	15,854	40,266	12,249	631	121,786	5,859	18,245
17	24,625	193,888	56,806	180,946	17,192	47,170	13,262	557	100,094	7,025	21,452
18	9,497	177,906	26,285	173,316	8,764	24,019	7,749	274	173,458	1,980	8,449
19	25,701	128,582	52,511	115,461	19,346	42,735	15,107	651	56,520	9,510	20,427
20	10,478	128,166	24,570	124,203	10,580	21,936	9,188	397	107,793	3,178	8,334
21	5,939	84,144	15,806	81,216	6,778	14,478	6,196	170	78,895	1,263	5,256
22	10,759	81,843	23,341	77,191	9,589	20,070	7,963	333	40,720	2,926	9,136
X	17,474	256,683	39,923	250,753	21,737	36,963	19,992	762	452,750	4,863	13,846
Y	2,199	29,049	6,767	28,535	5,074	6,406	4,811	238	75,449	263	2,058

Distribution of Palindromes in non-coding regions

Chromosome	LincRNA	SnoRNA/miRNA	CpG islands	TFBS
1	69,145	37	11,662	136,699
2	95,612	23	7,948	123,485
3	74,725	20	5,651	96,533
4	103,683	8	4,477	88,102
5	76,994	11	5,897	88,253
6	54,534	9	5,592	96,158
7	55,878	9	7,039	82,483
8	37,873	20	5,423	70,538
9	46,416	9	6,013	60,767
10	40,144	15	6,230	71,554
11	26,018	18	6,559	71,007
12	31,398	30	5,299	72,801
13	35,562	3	2,815	42,857
14	23,243	27	3,913	46,952
15	28,216	11	4,229	45,408
16	24,795	27	6,868	49,569
17	15,870	32	7,335	57,775
18	20,767	5	2,916	34,012
19	12,106	53	9,651	43,916
20	16,131	22	4,050	45,472
21	21,455	2	1,611	19,631
22	9,549	0	3,789	28,228
X	31,989	29	3,282	41,530
Y	6,967	No Sno/miRNA	226	790
