# Supplementary material for: A reference catalog of DNA palindromes in the human genome and their variations in 1000 Genomes
Source: Hum Genome Var. 2020 Nov 20;7:40. doi: 10.1038/s41439-020-00127-5 (PMC7680136; doi:10.1038/s41439-020-00127-5)
Supplement: Supplementary file 3 — Supplementary Table 3 [file 41439_2020_127_MOESM3_ESM.pdf]

Supplementary Table 3.

Intentionally left blank.

A large data file of catalog of palindromes will be uploaded at the time of publication.
