# Supplementary material for: A reference catalog of DNA palindromes in the human genome and their variations in 1000 Genomes
Source: Hum Genome Var. 2020 Nov 20;7:40. doi: 10.1038/s41439-020-00127-5 (PMC7680136; doi:10.1038/s41439-020-00127-5)
Supplement: Supplementary file 4 — Supplementary Table 4 [file 41439_2020_127_MOESM4_ESM.pdf]

1000 Genomes Sample ID	No.of Identical palindromes	No.of Smaller palindromes	No.of longer palindromes	No.of Different palindromes
HG03270	14,293,757	276,771	141,278	240,875
NA19676	14,474,629	242,601	126,320	206,619
HG03271	14,415,698	268,027	134,635	230,908
HG03279	14,296,665	274,610	140,813	240,177
NA19679	14,509,763	233,395	118,420	194,256
NA19678	14,384,836	244,544	125,719	205,686
HG01398	14,473,653	243,314	125,461	207,625
HG01390	14,352,161	255,776	131,826	217,639
HG01393	14,375,600	248,428	126,699	210,374
HG01392	14,465,859	247,527	126,518	210,260
HG01395	14,477,802	242,189	124,119	205,578
HG01396	14,380,642	246,811	125,561	208,527
HG00421	14,490,315	237,386	121,896	201,045
HG00422	14,410,460	236,502	119,296	196,951
HG00428	14,412,177	235,611	119,433	196,784
NA19773	14,395,616	241,602	123,505	201,834
HG02477	14,301,064	273,811	139,887	237,984
HG02476	14,297,373	274,693	140,290	238,298
HG02471	14,288,243	277,576	143,260	241,734
HG02470	14,394,112	274,817	139,656	236,447
NA20808	14,406,221	235,708	121,159	198,567
HG02479	14,293,710	276,240	140,827	239,439
HG02277	14,510,052	232,513	118,934	195,082
HG02275	14,426,417	229,396	117,485	191,934
HG02274	14,468,133	249,175	126,496	210,280
HG01260	14,379,174	247,176	126,781	209,126
HG02271	14,499,581	233,131	120,987	197,626
HG01269	14,386,215	242,239	125,832	205,461
HG02278	14,419,350	232,291	118,093	194,805
HG01067	14,394,826	240,884	123,645	203,133
NA19074	14,412,933	234,974	119,981	197,381
NA19077	14,407,351	237,030	121,119	198,146
HG01064	14,381,453	246,348	125,371	207,979
HG01063	14,457,428	251,918	127,076	213,090
NA19070	14,507,751	234,089	118,491	195,628
HG01061	14,389,225	243,855	124,019	203,843
NA19072	14,503,405	234,130	119,977	196,678
NA19079	14,512,947	232,464	117,346	194,411
NA19078	14,409,634	235,437	120,097	197,822
HG01069	14,489,238	241,753	122,078	201,501
HG02512	14,508,842	232,011	118,150	195,469
HG02513	14,409,548	235,701	120,456	198,351
HG02511	14,294,594	276,553	141,309	238,649
HG03018	14,477,471	242,436	125,071	205,353
HG03019	14,402,612	238,561	121,587	199,570
HG03012	14,500,698	237,405	119,570	198,408
HG03015	14,501,051	236,197	119,715	197,907
HG03016	14,395,520	241,905	123,104	202,639
HG00280	14,490,551	237,669	122,808	201,686
HG00281	14,410,970	235,609	120,649	197,475
HG00282	14,404,810	237,952	121,424	198,653
NA11919	14,497,608	236,346	121,426	199,538
HG00284	14,509,082	232,635	119,015	195,271
HG00285	14,413,876	234,913	119,624	196,623
HG00288	14,404,731	236,850	123,101	199,698
HG02604	14,412,797	234,073	121,498	196,771
HG02603	14,490,986	239,373	123,298	200,961
HG02600	14,469,677	244,950	127,764	207,946
HG02601	14,381,163	246,183	127,916	207,044
HG03705	14,499,343	237,746	119,586	197,880
HG03706	14,399,478	240,075	121,918	201,021
HG03702	14,495,676	238,283	121,243	200,704
HG03703	14,396,057	240,780	123,469	202,073
HG03708	14,479,877	241,565	124,744	205,630
HG03709	14,393,080	241,783	124,345	202,798
HG01885	14,414,469	268,850	134,940	230,587
HG01886	14,283,711	280,341	144,859	243,715
HG01880	14,319,554	267,674	137,734	231,163

HG01883	14,297,362	275,887	140,498	238,291
HG01882	14,398,722	272,773	137,838	236,980
HG01080	14,387,966	243,285	125,068	206,120
HG01889	14,242,011	281,193	145,342	282,349
HG03649	14,494,164	238,717	121,200	200,637
HG03640	14,394,712	241,099	123,346	202,856
HG03643	14,389,928	243,654	125,244	204,428
HG03642	14,385,718	244,089	126,354	205,766
HG03645	14,398,457	239,345	122,165	201,361
HG03644	14,466,610	246,222	128,089	208,705
HG03646	14,500,995	236,495	120,052	197,914
HG00268	14,410,663	235,814	119,998	197,536
HG00542	14,508,482	233,361	118,001	194,485
HG04042	14,402,704	237,714	121,857	200,689
HG04047	14,396,313	241,119	122,219	201,805
HG03803	14,494,994	239,048	121,658	199,744
HG03802	14,391,464	242,583	125,083	204,909
HG03800	14,492,776	239,973	122,140	200,687
HG03805	14,399,813	238,858	122,646	201,801
HG03809	14,476,965	232,259	117,119	231,736
HG03808	14,400,275	239,243	122,010	201,502
HG03548	14,288,813	278,164	141,849	244,337
HG02860	14,396,982	273,150	138,596	237,494
HG02861	14,298,135	275,478	140,585	239,011
NA20581	14,504,739	233,661	119,848	197,591
NA20582	14,419,701	232,525	119,533	195,564
NA20585	14,417,685	233,206	118,789	194,593
HG03625	14,414,259	233,810	119,264	195,596
NA20587	14,400,592	239,189	123,672	200,995
NA20586	14,489,829	237,384	123,338	201,180
NA20589	14,404,594	237,361	121,757	199,700
NA20588	14,488,213	237,891	123,435	203,160
HG03624	14,480,694	240,917	125,039	204,456
NA18553	14,404,264	238,791	121,259	199,392
HG04134	14,481,342	240,203	124,396	204,748
HG00129	14,513,216	231,384	117,540	193,955
HG00128	14,413,664	233,990	119,805	197,145
HG04131	14,492,995	238,650	121,611	200,344
HG00125	14,410,912	235,448	120,268	197,935
HG00127	14,410,923	234,260	121,956	196,996
HG00126	14,490,192	236,225	123,720	201,166
HG00121	14,418,473	232,075	119,395	195,721
HG00120	14,410,649	235,753	120,402	197,476
HG00123	14,405,797	237,730	121,436	198,362
HG00122	14,410,807	235,788	120,449	196,630
HG01468	14,423,730	230,987	117,213	194,085
NA18557	14,506,507	233,630	119,045	195,161
HG01462	14,364,640	252,021	128,779	214,107
HG01461	14,479,564	244,794	123,243	205,803
HG01465	14,411,113	235,353	119,338	197,827
HG01464	14,468,569	248,262	126,259	209,065
NA20127	14,295,279	276,076	142,493	240,106
NA20126	14,410,093	267,842	136,906	230,943
NA20832	14,410,479	235,971	120,088	197,655
HG00638	14,372,798	248,158	128,242	211,898
HG00631	14,501,808	235,864	121,227	197,095
HG00632	14,403,464	238,688	122,695	199,696
HG00634	14,506,830	233,708	119,783	195,300
HG00637	14,489,427	241,405	122,195	202,933
HG02282	14,285,876	278,511	144,769	241,624
HG02283	14,414,001	268,380	135,473	231,608
HG02281	14,407,604	272,076	137,011	233,223
HG02286	14,410,245	235,189	119,949	198,271
HG02284	14,415,256	268,406	134,896	229,912
HG02285	14,491,751	236,480	122,813	200,088
NA20905	14,497,280	237,573	120,260	199,323
NA20904	14,481,838	240,940	124,221	204,418
HG01098	14,390,754	242,548	124,601	204,425
NA20906	14,398,393	239,427	122,644	202,029

HG02048	14,391,588	242,987	125,215	203,149
NA20900	14,397,802	239,835	123,784	203,568
NA20903	14,499,407	236,664	121,020	198,775
NA20902	14,399,947	239,143	122,552	200,863
HG01092	14,371,745	250,318	127,746	211,706
HG02047	14,509,043	234,030	118,167	195,158
HG02040	14,508,186	234,113	118,061	195,393
NA20908	14,399,012	239,749	122,329	202,224
HG01094	14,489,313	240,818	121,654	203,175
HG01095	14,381,368	245,584	127,003	207,973
HG00705	14,409,362	236,363	119,935	197,508
HG00704	14,510,700	233,193	117,626	193,784
HG00707	14,510,470	232,994	117,925	193,881
HG00701	14,503,942	234,462	119,360	196,730
NA19682	14,491,771	238,129	122,488	200,954
NA19681	14,392,859	240,957	123,559	202,739
HG00708	14,408,817	236,061	120,454	198,175
NA19684	14,397,507	240,566	123,099	201,753
HG03095	14,288,954	278,180	141,227	242,612
NA19118	14,297,442	274,520	140,743	239,798
NA19119	14,398,957	273,197	138,220	234,995
NA19448	14,417,242	268,526	134,701	228,811
NA19449	14,297,146	275,741	140,176	238,860
NA19445	14,293,781	276,700	140,466	240,068
NA19446	14,292,116	276,762	142,160	241,088
NA19440	14,293,647	277,036	142,252	240,604
NA19117	14,418,662	267,886	133,660	229,452
NA19114	14,290,053	276,926	143,280	241,731
NA19443	14,396,509	273,856	138,288	236,328
NA19664	14,482,035	240,943	123,791	204,509
NA19661	14,492,207	240,167	122,234	200,319
NA19663	14,402,792	238,912	121,509	200,221
HG03247	14,293,516	276,945	140,965	240,116
HG03246	14,420,361	266,763	133,213	228,561
HG03241	14,288,218	278,876	142,697	243,188
HG03240	14,414,971	269,016	134,740	230,657
HG03428	14,291,952	277,424	141,713	241,560
HG01384	14,393,818	240,663	123,506	202,809
HG01383	14,498,842	236,720	120,890	198,604
HG01389	14,481,306	236,379	120,610	216,697
NA18648	14,510,821	232,393	118,357	194,268
NA18645	14,510,490	232,550	117,742	194,689
NA18644	14,412,416	234,861	119,779	197,075
NA18647	14,466,696	233,317	119,240	234,560
NA18646	14,410,480	235,190	120,738	197,564
NA18641	14,411,035	236,043	120,042	197,117
NA18640	14,411,113	235,376	120,228	197,921
NA18643	14,510,796	231,972	118,089	195,581
NA18642	14,410,693	235,932	120,192	197,649
NA19206	14,294,896	276,604	141,013	239,785
HG00410	14,397,216	240,656	124,172	201,451
NA19204	14,286,403	279,108	143,794	242,902
NA19200	14,392,412	274,306	140,638	237,529
NA19201	14,296,598	275,692	140,541	238,964
HG00419	14,407,537	237,206	120,475	198,582
HG02374	14,488,748	237,759	122,811	202,955
NA19209	14,293,627	276,596	141,586	240,573
HG02375	14,487,354	239,351	122,176	202,644
HG02462	14,283,609	279,885	145,856	243,657
HG02461	14,412,983	270,084	135,918	230,467
HG02464	14,391,006	275,293	140,363	238,407
HG02465	14,282,958	279,843	145,549	243,696
HG01272	14,380,326	247,116	125,069	208,378
HG02265	14,526,271	226,230	115,002	189,262
HG02266	14,428,171	229,225	117,062	191,381
HG01271	14,489,989	239,831	121,294	202,563
HG02260	14,417,003	232,936	119,123	195,096
HG01277	14,508,754	234,063	118,276	195,621
HG02262	14,489,463	236,731	123,302	201,654

HG01275	14,414,987	233,431	119,529	195,264
HG03391	14,406,295	272,064	135,992	233,733
HG03397	14,403,856	273,151	137,455	234,318
HG03394	14,410,891	270,473	135,676	232,601
NA19062	14,507,623	233,721	118,878	195,895
NA19063	14,487,917	239,138	122,771	201,846
NA19060	14,489,473	238,453	122,614	201,675
HG01077	14,370,801	248,908	127,874	211,711
NA19066	14,484,014	240,235	124,413	202,963
NA19067	14,501,044	235,411	120,228	197,909
NA19064	14,405,597	237,682	121,393	198,893
HG01073	14,395,454	241,867	123,082	203,081
NA19068	14,500,471	237,655	120,529	196,941
HG01079	14,483,270	243,667	122,589	204,441
HG04238	14,497,987	236,563	120,631	199,501
HG03025	14,297,901	274,918	140,618	240,792
HG03024	14,401,636	271,643	137,637	235,432
HG03027	14,410,627	270,898	136,579	231,464
HG03021	14,497,703	237,302	120,615	198,796
HG03022	14,410,401	235,374	119,966	197,914
HG03028	14,282,391	279,259	146,058	245,408
HG02676	14,284,341	279,646	145,358	243,741
HG02675	14,389,671	275,657	141,276	238,492
HG02679	14,281,644	281,298	146,680	244,841
HG02678	14,422,220	266,860	134,425	228,087
HG03731	14,389,917	242,275	125,484	203,964
HG03730	14,389,976	242,796	125,206	204,112
HG03733	14,409,692	235,787	120,179	198,777
HG03736	14,400,050	239,508	122,084	201,895
HG03738	14,505,157	233,769	119,294	196,908
NA12814	14,494,135	235,800	122,047	200,710
NA12815	14,412,059	235,548	119,496	196,939
NA12812	14,499,164	235,926	120,301	198,517
NA12813	14,411,120	235,618	120,246	197,276
HG00273	14,508,896	233,473	118,500	195,481
HG03944	14,402,823	237,837	121,643	200,464
HG01892	14,475,103	242,186	126,423	206,310
HG01893	14,384,094	244,666	125,313	206,571
HG01890	14,412,112	270,076	135,866	231,135
HG00368	14,406,369	236,685	121,933	199,646
HG03652	14,491,447	240,390	122,295	200,423
HG03653	14,392,650	242,331	123,396	203,297
HG01894	14,299,254	274,491	140,876	238,542
HG00381	14,414,784	234,186	119,736	196,444
HG03829	14,399,457	239,038	122,475	201,315
HG00383	14,415,521	234,165	118,869	195,925
HG00382	14,512,529	232,021	118,247	193,798
HG00384	14,409,670	236,002	120,688	198,212
HG00272	14,408,089	236,208	121,145	198,260
NA18934	14,410,405	270,398	135,633	231,412
NA18933	14,292,772	276,453	142,253	240,113
HG03539	14,298,444	274,408	140,966	238,336
HG00187	14,507,009	232,975	119,795	195,968
HG03538	14,402,506	270,757	137,422	234,408
HG02703	14,286,183	279,108	145,006	242,648
HG02702	14,410,546	270,895	136,973	231,889
NA18939	14,410,276	235,292	119,667	198,259
HG02700	14,400,410	239,562	122,240	200,705
NA11894	14,409,163	235,458	120,818	198,565
NA11893	14,507,073	233,738	119,367	196,050
NA11892	14,409,708	236,368	121,106	198,414
HG03949	14,401,746	238,522	121,602	200,057
HG00257	14,410,522	236,840	119,601	197,300
HG00256	14,507,922	234,072	118,613	195,602
HG00255	14,405,756	237,547	121,899	199,074
HG00254	14,412,282	234,491	120,085	197,435
HG00253	14,409,573	235,902	120,426	198,228
HG00252	14,511,916	232,177	117,656	193,677
HG00251	14,511,589	232,697	118,378	195,133

HG03817	14,404,029	237,924	121,130	199,835
HG00259	14,409,170	236,171	120,758	198,735
HG00258	14,410,627	236,350	120,210	197,173
HG02819	14,416,069	268,959	134,197	231,053
HG02813	14,420,109	267,027	133,708	229,836
HG02811	14,289,154	279,067	142,253	242,272
HG02810	14,424,544	265,300	134,010	226,806
HG02817	14,298,991	274,557	140,227	238,938
HG02816	14,415,947	269,421	134,248	230,254
HG02814	14,293,704	276,523	141,187	241,214
HG00138	14,490,595	237,486	122,436	201,306
HG04140	14,498,933	237,462	119,708	197,927
HG04146	14,489,434	239,491	122,236	201,866
HG04144	14,392,324	242,129	123,717	203,292
HG00132	14,408,235	235,961	121,440	198,277
HG00133	14,403,910	238,596	121,201	198,841
HG00130	14,416,455	232,623	118,829	196,685
HG00131	14,492,184	236,093	122,456	200,626
HG00136	14,507,602	233,612	119,176	195,925
HG00137	14,419,706	232,254	118,692	194,363
HG02272	14,432,563	226,384	116,144	189,468
HG01479	14,504,878	234,582	119,209	196,378
HG01474	14,402,038	237,942	121,939	199,748
HG02923	14,409,972	271,301	136,868	231,819
HG02922	14,279,705	281,982	146,321	244,859
HG02807	14,406,343	273,156	137,606	232,792
NA20826	14,406,433	236,936	121,541	198,869
NA20827	14,508,808	233,058	118,613	194,767
NA20822	14,399,576	238,981	124,226	202,392
NA20821	14,399,086	239,248	124,355	200,997
NA20828	14,410,573	235,509	120,792	198,553
HG00626	14,414,476	233,765	119,971	197,463
HG00625	14,511,338	231,750	118,559	194,632
HG00622	14,482,760	240,667	124,934	203,194
HG00623	14,401,899	238,434	123,016	200,758
HG00620	14,409,202	236,409	120,519	197,936
HG01710	14,404,049	237,427	121,294	200,131
HG00628	14,511,820	232,139	118,605	194,387
HG00629	14,410,438	235,330	121,542	198,101
HG02131	14,500,963	236,022	120,025	197,828
HG04090	14,400,235	239,462	121,651	201,037
HG04093	14,466,986	234,205	119,505	234,426
HG04094	14,500,292	236,948	119,730	197,654
HG04096	14,486,508	238,473	123,331	203,146
HG04099	14,399,561	239,050	122,105	201,972
HG04098	14,478,160	242,427	124,880	205,217
NA20895	14,501,279	235,657	119,972	198,205
HG02079	14,500,163	236,772	121,058	197,808
HG02078	14,400,976	239,509	122,638	200,145
NA20910	14,400,969	237,500	123,042	201,467
NA20911	14,499,238	237,039	120,375	198,547
HG02070	14,499,593	237,161	120,792	197,095
HG02073	14,484,384	241,051	123,490	203,226
HG02072	14,394,704	242,208	123,790	202,094
HG02075	14,398,281	239,989	123,259	201,574
HG00335	14,455,609	236,711	122,203	238,593
HG02076	14,489,046	238,796	123,663	201,218
HG01669	14,504,601	234,810	119,539	197,015
HG01668	14,410,297	234,602	120,358	198,540
HG00717	14,409,916	236,297	119,507	197,269
NA20890	14,503,881	234,975	119,598	197,638
HG04152	14,478,922	241,176	125,116	205,289
HG04153	14,397,226	241,063	122,673	202,737
NA19471	14,294,312	277,085	140,598	240,617
NA19473	14,296,180	275,659	140,684	239,463
NA19472	14,292,483	276,309	141,965	241,274
NA19475	14,290,205	277,914	141,425	241,700
NA19474	14,295,631	276,594	140,558	238,968
HG04155	14,498,839	236,748	120,832	198,833

NA19651	14,394,734	241,618	122,446	202,719
HG02382	14,486,392	239,412	122,522	202,215
HG03258	14,409,067	271,910	136,301	231,955
NA19652	14,496,493	238,994	120,589	199,064
NA19655	14,499,347	236,669	120,242	198,196
NA19654	14,395,360	241,960	123,548	202,453
NA19309	14,397,083	272,616	139,222	237,227
NA19308	14,395,403	273,417	138,908	237,909
NA19307	14,392,215	274,655	140,010	238,015
NA19658	14,501,932	236,143	119,438	197,621
HG02389	14,510,966	232,493	117,858	194,886
NA11918	14,408,611	235,766	120,777	198,476
HG01101	14,466,333	247,553	127,306	209,976
NA21087	14,498,328	238,183	120,055	198,378
NA21086	14,399,500	240,312	122,296	200,986
HG01102	14,382,379	245,394	125,852	208,098
NA12829	14,507,991	233,764	118,900	195,692
HG03086	14,286,746	279,163	142,537	243,007
NA21088	14,400,174	239,496	121,924	200,854
NA19783	14,505,746	234,610	119,365	195,513
NA19782	14,392,589	242,695	123,978	203,647
NA19780	14,497,864	238,306	120,167	198,615
NA19786	14,501,579	235,952	120,236	198,277
NA19785	14,395,595	240,590	123,646	203,078
NA19789	14,564,596	245,073	126,216	207,387
NA19788	14,392,786	243,379	123,652	202,850
HG00409	14,503,733	236,239	120,078	196,654
HG00406	14,509,200	232,889	117,714	194,931
HG00407	14,407,424	236,518	120,522	198,499
HG00404	14,408,104	236,090	121,675	198,720
HG00403	14,490,317	236,850	123,442	201,525
HG04019	14,480,289	240,217	124,882	205,383
NA20517	14,409,121	235,635	121,406	198,132
HG00331	14,410,700	235,555	120,522	197,277
HG02497	14,296,185	276,221	140,723	239,018
HG02496	14,420,340	267,317	133,428	228,037
HG02494	14,396,719	240,388	123,699	201,817
HG02493	14,494,681	238,389	121,508	200,170
HG02491	14,392,584	241,920	125,216	203,280
HG02490	14,481,436	240,657	125,120	204,164
HG03388	14,406,382	270,922	136,290	232,842
HG02250	14,500,346	235,622	120,045	198,148
HG02253	14,508,840	233,029	117,759	195,081
HG02252	14,405,841	235,907	121,386	200,210
HG02255	14,418,695	267,675	134,331	228,278
HG02256	14,305,992	271,592	139,154	236,130
HG02259	14,521,973	227,561	116,165	190,391
HG01286	14,473,558	245,452	125,316	207,894
HG03382	14,407,347	273,038	135,001	232,496
HG01284	14,390,765	241,842	123,211	203,822
HG03385	14,416,863	268,411	133,915	229,700
HG01281	14,400,858	238,677	122,402	201,703
HG01280	14,498,803	237,131	121,000	198,393
NA19099	14,295,568	275,608	140,863	240,752
HG01046	14,416,745	233,917	118,434	195,833
HG01049	14,365,481	251,641	128,662	213,155
HG01048	14,491,614	239,925	122,017	201,351
NA19095	14,291,590	276,792	141,805	241,020
NA19093	14,290,336	277,598	144,080	243,363
NA19092	14,415,067	269,255	134,622	230,618
NA19091	14,492,721	235,577	122,512	200,592
NA19090	14,411,558	235,034	119,768	197,799
NA20518	14,505,975	233,561	119,644	196,682
NA19750	14,500,160	236,043	120,647	198,614
HG03039	14,409,310	272,325	136,638	232,619
NA12890	14,403,880	237,360	122,568	199,946
NA19010	14,410,342	236,006	120,206	197,276
HG03190	14,400,076	271,104	137,958	235,600
HG02660	14,478,658	241,429	126,511	205,672

HG02661	14,386,576	243,942	126,566	205,419
HG02666	14,387,361	277,028	141,957	239,086
HG02667	14,299,345	274,080	141,491	238,097
HG03729	14,490,718	240,650	123,100	200,929
HG03727	14,491,828	240,073	122,932	200,726
HG03722	14,395,437	240,606	124,581	202,349
HG03720	14,481,934	239,977	124,167	204,984
HG01869	14,406,865	236,738	120,838	199,044
HG01868	14,410,076	235,558	119,790	198,058
HG01867	14,509,535	233,364	118,815	195,662
HG01866	14,489,121	237,464	122,712	202,273
HG03629	14,492,978	240,032	122,607	201,289
HG01864	14,505,770	234,420	118,958	196,377
HG01863	14,411,006	235,160	120,068	197,971
HG01862	14,410,002	236,413	119,367	197,472
HG01861	14,507,886	233,236	118,353	195,478
HG01860	14,509,778	232,753	117,588	195,251
NA18924	14,290,755	278,025	142,660	241,182
NA18923	14,412,613	269,965	135,760	231,027
HG02715	14,388,522	276,621	141,288	239,128
HG02716	14,281,704	280,806	145,955	245,148
HG02181	14,411,370	235,458	119,492	197,946
HG02180	14,411,168	236,158	119,634	197,594
NA11881	14,508,891	232,567	118,739	195,810
NA12045	14,511,296	233,716	117,907	194,350
NA12044	14,406,528	237,250	121,462	198,598
NA12046	14,407,010	236,844	121,322	199,256
NA12043	14,497,894	235,386	120,815	199,152
HG02185	14,412,569	235,637	119,896	196,765
NA18613	14,510,029	233,120	118,186	194,436
HG00244	14,504,839	234,810	119,188	196,215
HG00245	14,412,244	235,546	118,687	197,468
HG00246	14,507,785	233,830	118,735	194,900
HG00240	14,403,990	237,598	122,739	200,806
NA18610	14,409,025	236,416	120,290	197,737
HG00242	14,508,112	233,786	119,230	195,409
HG00243	14,486,311	237,911	124,246	203,129
HG03824	14,495,303	238,865	120,455	198,990
HG03826	14,394,703	240,714	123,921	203,376
HG03821	14,498,130	237,642	119,939	198,929
HG01708	14,509,430	233,580	118,551	194,722
HG03823	14,398,733	239,270	122,651	202,710
HG02808	14,292,340	276,420	143,569	240,894
NA18595	14,410,222	236,598	119,789	197,744
NA12275	14,371,108	235,197	120,785	235,934
HG00657	14,408,643	236,686	120,542	197,708
HG02804	14,409,551	271,728	136,643	232,441
HG02805	14,295,517	275,511	143,358	240,170
NA12273	14,403,674	237,750	122,001	200,359
HG02223	14,413,346	234,461	120,110	197,739
NA18591	14,410,959	235,436	119,818	197,254
HG01917	14,497,265	235,367	120,982	198,626
HG01444	14,400,244	239,293	121,669	201,883
HG01447	14,388,762	244,028	124,224	205,771
HG01914	14,421,433	266,539	134,340	226,966
HG01441	14,402,892	238,334	121,714	200,028
HG01912	14,397,802	272,608	138,782	236,913
HG04156	14,399,288	239,854	122,493	201,741
HG04158	14,479,121	241,473	125,242	205,266
HG04159	14,400,971	239,013	122,284	201,742
HG01918	14,413,060	235,015	120,541	195,909
HG01241	14,401,904	270,794	139,539	234,357
HG00525	14,410,002	235,529	120,940	197,871
HG02938	14,412,923	269,903	135,812	231,840
NA20853	14,403,053	238,426	121,123	201,230
NA20852	14,505,254	234,358	119,669	197,320
NA20851	14,402,645	238,563	121,383	200,176
NA20850	14,487,919	238,544	123,128	202,534
NA20856	14,405,095	237,106	121,192	199,682

NA20854	14,397,945	241,114	121,965	201,216
NA20859	14,403,027	237,784	121,338	200,443
NA20858	14,501,014	236,157	120,141	197,742
HG00653	14,505,734	234,459	118,825	196,279
HG00651	14,412,860	234,600	120,152	196,697
HG01709	14,514,571	232,588	116,747	194,220
NA12272	14,509,211	232,998	118,803	195,294
HG00656	14,488,697	238,013	123,407	202,284
HG00654	14,412,293	234,904	119,771	197,046
HG01702	14,415,685	232,970	119,381	196,705
HG01700	14,518,188	229,948	116,697	192,467
HG01707	14,410,896	235,077	120,706	197,838
HG01704	14,405,194	237,288	121,233	199,666
HG01705	14,506,190	234,177	119,122	196,379
HG00097	14,409,402	235,530	120,419	197,574
HG00096	14,489,297	237,881	122,990	201,912
HG00099	14,409,772	235,676	119,613	197,366
HG03563	14,291,025	277,772	142,132	242,224
HG04080	14,497,195	237,788	121,208	199,655
HG02067	14,487,520	238,847	122,708	202,653
HG02064	14,501,739	235,483	119,813	197,533
HG02060	14,408,707	236,944	120,173	198,076
HG02061	14,488,439	237,484	122,626	202,473
HG00232	14,414,964	233,548	120,328	195,770
HG02069	14,394,314	242,561	124,707	202,184
HG01676	14,405,940	237,448	121,371	198,515
HG01675	14,507,988	233,041	119,106	195,291
HG01672	14,491,394	236,485	122,850	201,458
HG01673	14,409,356	236,049	120,907	197,271
HG01670	14,412,506	234,060	120,072	197,373
NA21114	14,478,450	241,434	125,343	207,072
HG01678	14,508,148	233,596	118,930	195,635
HG01679	14,410,281	235,032	120,760	197,390
NA19466	14,418,997	268,236	133,030	228,352
NA19467	14,302,170	274,189	139,687	238,029
NA19462	14,295,315	276,032	141,315	240,112
NA19463	14,289,421	278,060	142,112	241,040
NA19461	14,417,721	267,044	133,700	229,812
NA19468	14,297,167	275,137	140,862	239,829
HG03048	14,409,916	270,886	137,034	232,341
NA12748	14,505,288	234,685	119,903	196,432
HG02390	14,493,157	236,586	121,817	200,742
HG02391	14,507,162	233,825	118,873	195,309
HG02392	14,509,161	233,387	118,422	194,514
NA19318	14,407,690	271,330	136,766	232,423
HG02395	14,504,192	235,036	118,151	196,164
NA19648	14,412,644	234,984	119,320	196,517
NA19649	14,485,001	239,044	123,560	203,893
NA19314	14,293,756	275,564	141,381	240,041
HG02399	14,487,293	237,964	123,144	202,158
NA19316	14,289,038	278,047	142,555	240,910
NA19317	14,397,225	272,471	139,400	236,842
NA19310	14,297,217	276,108	140,066	239,369
NA19312	14,419,588	267,560	133,413	228,741
NA21094	14,500,545	236,369	120,123	197,984
NA21095	14,502,625	235,669	120,054	198,108
NA21097	14,400,187	239,307	122,355	200,730
NA21090	14,495,101	238,314	120,982	199,631
NA21091	14,505,759	234,598	119,332	197,063
NA21092	14,496,153	238,172	121,467	199,303
NA21093	14,498,744	236,830	120,086	198,138
NA21098	14,498,214	238,289	120,945	198,384
NA21099	14,502,863	236,793	119,671	197,603
NA19792	14,486,157	242,479	123,792	202,934
NA19794	14,399,771	239,666	122,462	201,703
NA19795	14,494,488	238,742	120,798	200,464
NA19904	14,401,882	270,976	138,238	233,621
HG00479	14,409,125	236,153	120,019	197,774
HG00478	14,489,762	237,595	122,248	201,806

HG00473	14,405,589	237,639	120,822	198,993
HG00472	14,509,445	233,269	117,560	195,193
HG00476	14,410,446	236,129	119,367	198,129
HG00475	14,506,946	233,973	118,368	195,872
HG02489	14,393,797	274,486	139,704	236,556
HG02484	14,415,581	268,250	134,112	229,321
HG02485	14,307,666	270,968	139,517	235,058
HG02481	14,412,117	269,838	135,600	231,332
NA19088	14,504,630	234,550	119,497	195,387
NA19089	14,508,150	234,217	118,778	195,104
HG01058	14,390,963	242,938	123,932	204,123
HG00500	14,510,285	232,958	117,929	194,538
NA19084	14,411,156	236,152	119,722	197,034
NA19085	14,504,158	235,752	118,471	196,685
HG01054	14,489,564	240,061	122,406	201,445
NA19087	14,410,938	235,356	120,292	197,995
HG01052	14,352,869	254,987	131,813	217,331
NA19081	14,415,773	234,533	118,553	195,557
NA19082	14,487,391	238,534	123,680	202,167
NA19083	14,506,618	233,630	118,711	196,625
NA19144	14,395,054	274,237	138,650	237,352
NA19147	14,297,892	274,179	140,444	239,942
HG02142	14,400,106	239,990	122,882	200,862
HG03280	14,411,272	269,581	134,688	231,941
HG02380	14,514,557	231,944	116,763	192,328
HG03815	14,497,754	236,904	121,425	200,147
HG03899	14,501,263	236,012	119,852	197,770
HG02658	14,423,139	232,377	118,556	193,063
HG02655	14,402,414	237,670	123,005	201,035
HG02654	14,490,297	240,165	122,912	200,659
HG02657	14,488,519	240,368	123,894	202,064
HG02651	14,495,742	238,498	121,635	199,337
HG02652	14,397,185	240,310	123,298	202,931
HG03753	14,494,936	238,113	120,430	200,340
HG03752	14,406,648	236,906	121,357	199,272
HG03750	14,476,586	242,572	126,223	206,052
HG03757	14,388,182	242,984	125,967	205,305
HG03756	14,391,559	241,541	125,936	204,277
HG03755	14,494,470	239,246	120,823	199,704
HG03754	14,386,518	244,294	126,261	204,707
NA12872	14,505,752	234,437	119,073	195,892
HG01248	14,385,151	244,619	125,157	206,602
NA12874	14,496,315	234,968	121,404	199,188
NA12878	14,408,871	235,801	120,305	198,289
HG03585	14,477,922	241,943	125,111	206,419
HG03631	14,390,115	241,887	125,974	205,787
HG03634	14,396,317	241,499	122,853	202,674
HG01498	14,396,130	241,171	123,301	201,808
HG03636	14,499,550	237,009	120,365	198,602
HG01874	14,395,706	242,051	123,661	201,303
HG01870	14,398,732	240,398	123,221	199,734
HG01871	14,395,702	241,857	123,902	201,517
HG01872	14,495,615	239,139	122,037	198,991
HG01873	14,492,101	240,122	122,777	199,375
HG02728	14,387,737	244,299	125,716	204,775
NA18912	14,286,924	279,436	143,335	241,765
NA18915	14,390,221	275,556	141,177	239,135
NA18917	14,416,170	269,030	134,633	230,553
NA18916	14,289,072	278,006	142,097	241,944
HG02721	14,410,148	271,094	136,957	232,073
HG02722	14,280,807	280,821	145,969	244,239
HG02725	14,385,088	244,744	126,932	205,198
HG02724	14,489,134	239,338	123,726	202,660
HG02727	14,494,007	239,720	122,001	200,086
NA18615	14,411,214	235,778	119,768	197,502
NA07357	14,510,984	232,089	118,355	195,335
NA12058	14,405,940	237,399	121,512	198,313
HG00271	14,486,076	238,951	123,600	203,705
HG03838	14,392,064	242,158	125,070	203,746

HG02836	14,419,078	268,021	134,304	229,203
HG00275	14,411,984	234,660	120,373	197,264
HG00274	14,411,491	234,936	120,517	196,927
HG00277	14,505,880	233,243	120,226	196,054
HG00276	14,409,846	235,642	120,915	197,487
HG03832	14,396,405	240,468	123,024	202,785
HG00278	14,513,382	231,666	117,396	193,533
HG03830	14,497,889	237,508	120,136	199,753
NA12828	14,410,602	235,408	120,991	198,403
HG02839	14,418,853	267,707	133,634	230,212
HG03521	14,413,343	270,220	135,863	230,772
HG04219	14,479,026	241,104	124,303	205,216
HG01456	14,389,536	243,657	123,876	204,504
HG01455	14,496,036	238,414	121,668	199,767
HG04164	14,472,243	243,612	126,590	206,939
HG01459	14,396,671	241,327	122,644	202,018
HG04161	14,476,389	242,057	125,997	206,954
HG04162	14,394,210	241,714	123,565	203,847
NA18611	14,509,006	233,618	117,870	195,626
NA20845	14,493,856	238,537	121,218	200,969
NA20846	14,506,614	234,108	119,010	196,353
NA20847	14,402,685	238,171	121,658	200,840
NA20849	14,398,193	240,212	121,954	201,376
HG00640	14,475,772	242,515	126,848	206,795
HG00641	14,397,248	240,569	122,114	201,768
HG02394	14,508,108	233,610	118,040	196,007
NA19319	14,409,250	271,251	136,988	232,482
HG02396	14,508,800	233,491	118,246	195,188
HG02798	14,388,044	276,670	141,539	238,911
HG02398	14,508,800	232,795	118,114	195,650
HG00250	14,408,148	235,931	121,452	197,916
NA19315	14,294,013	276,342	140,868	240,316
HG02783	14,486,256	241,955	124,215	202,073
HG02013	14,412,077	269,852	135,398	230,617
HG02012	14,296,914	275,275	140,789	239,542
HG02010	14,295,214	276,165	141,834	239,479
HG02017	14,487,096	237,742	123,491	201,969
HG02016	14,409,998	236,213	119,799	198,426
HG02014	14,407,940	272,680	138,021	231,360
HG02019	14,412,212	235,061	119,455	198,033
HG01603	14,506,582	234,500	120,090	196,651
HG01602	14,408,270	237,106	120,579	198,014
HG01600	14,405,838	237,640	120,981	199,097
HG01607	14,410,290	235,404	121,401	197,410
HG01606	14,507,128	233,985	118,835	195,854
HG01605	14,406,190	237,020	121,104	198,217
HG01608	14,485,172	239,226	123,295	203,061
HG04186	14,398,937	239,242	122,737	201,654
HG04185	14,496,286	238,621	120,263	199,286
HG04183	14,401,602	239,134	122,055	201,141
HG04182	14,498,062	237,306	120,934	198,940
HG04180	14,382,635	244,617	127,589	206,809
HG04189	14,394,483	241,252	123,175	203,285
HG04188	14,475,857	242,926	125,065	206,832
NA19328	14,292,811	277,017	141,728	240,648
NA19321	14,291,060	277,763	142,266	241,978
NA19320	14,293,179	277,338	140,613	240,970
NA19323	14,291,860	276,741	142,920	240,976
NA19324	14,293,792	275,452	142,729	241,073
NA19327	14,288,281	278,267	143,818	241,856
HG02128	14,501,394	236,469	119,957	197,673
HG02127	14,404,516	238,480	122,152	199,426
HG02122	14,500,841	236,523	120,664	197,669
HG02121	14,401,867	239,259	122,329	200,647
NA07037	14,402,788	238,562	122,782	199,884
HG01119	14,400,800	238,149	122,140	200,949
HG01113	14,403,478	238,821	121,548	199,564
HG01112	14,452,276	238,123	122,428	239,619
HG01111	14,382,409	244,738	126,062	208,361

HG01110	14,490,568	240,542	121,706	201,568
NA19257	14,296,077	276,130	140,181	238,990
HG00463	14,504,580	234,569	119,051	196,498
HG00464	14,410,586	235,763	120,344	197,977
NA19747	14,497,863	238,122	120,862	198,713
NA19746	14,383,605	243,298	126,898	206,151
NA19741	14,513,478	231,209	117,282	193,521
NA19740	14,402,307	237,574	121,933	200,782
NA19749	14,397,819	240,162	122,922	201,309
HG02239	14,401,308	237,833	122,362	201,118
HG02238	14,509,539	232,917	117,714	194,735
HG02233	14,502,544	235,576	119,303	197,255
HG02232	14,406,355	236,792	120,729	198,362
HG02231	14,508,623	233,073	118,147	195,084
HG02230	14,408,317	235,940	121,265	198,910
HG02236	14,510,398	232,792	118,413	194,695
HG02235	14,409,892	235,585	120,723	198,461
HG01029	14,415,516	234,379	119,511	196,771
HG01028	14,505,031	234,304	119,066	195,785
HG00513	14,409,753	235,510	120,626	196,848
HG01491	14,494,345	235,314	122,017	199,937
HG02345	14,386,719	244,281	124,469	204,862
HG02343	14,402,515	270,616	138,495	234,013
HG03298	14,410,580	270,790	135,151	231,479
HG03297	14,293,157	277,081	140,816	240,450
HG03294	14,298,422	275,284	139,940	238,549
HG03295	14,413,903	268,242	135,734	231,387
HG02348	14,420,464	231,955	118,332	194,188
HG01334	14,491,312	237,464	122,075	200,822
NA12400	14,411,954	234,543	120,410	196,996
HG02648	14,507,490	233,667	118,768	195,565
HG02649	14,409,700	235,812	120,609	197,596
HG02642	14,411,870	270,870	136,389	231,923
HG02643	14,284,859	279,871	144,977	244,251
HG02646	14,282,883	280,243	145,022	244,225
HG02645	14,406,626	272,391	137,688	232,430
HG03740	14,482,652	240,088	125,321	203,525
HG03741	14,393,276	242,045	124,181	202,333
HG03742	14,501,125	236,186	119,823	198,131
HG03743	14,495,689	238,511	121,577	199,104
HG03744	14,480,331	241,293	124,737	205,854
HG03745	14,502,721	235,402	119,720	197,511
HG03746	14,491,228	240,977	122,590	200,770
HG00881	14,487,252	238,635	122,605	202,463
NA12843	14,406,241	237,885	121,709	198,753
NA12842	14,508,320	233,521	119,206	195,617
NA18528	14,403,370	238,900	122,400	199,657
HG02736	14,495,556	237,327	121,344	199,923
HG02737	14,396,158	240,297	123,440	203,018
HG02734	14,412,739	234,497	121,204	197,763
HG00867	14,408,163	237,223	120,509	198,969
HG02733	14,491,394	240,809	123,294	201,016
HG02731	14,397,683	241,744	122,129	202,001
HG01841	14,409,914	235,633	119,908	197,462
HG01840	14,484,361	240,361	124,209	203,451
HG03607	14,396,132	240,867	122,766	202,786
HG01842	14,488,866	238,539	122,103	201,606
HG01845	14,403,112	237,953	121,459	199,819
HG01844	14,504,904	235,543	118,691	195,686
HG03603	14,497,518	236,959	120,747	199,871
HG01846	14,486,031	239,458	123,472	202,912
NA18908	14,416,935	268,347	134,508	230,119
NA18909	14,294,148	276,883	141,380	240,597
NA18907	14,290,366	278,176	142,048	242,311
HG01593	14,408,571	235,114	120,851	197,647
HG01597	14,408,928	237,164	120,114	197,933
HG01596	14,489,476	236,542	123,222	201,296
HG01595	14,410,952	235,380	120,227	197,612
HG01599	14,407,311	237,681	120,254	198,504

NA07347	14,499,265	236,122	121,116	198,193
HG02586	14,285,032	279,006	145,192	243,432
HG03518	14,406,004	271,897	135,995	232,692
HG02820	14,298,987	274,145	140,098	240,313
HG04214	14,396,189	241,186	123,075	201,848
HG04212	14,402,210	238,129	121,601	200,133
HG04211	14,517,578	230,080	116,801	191,638
HG04210	14,492,950	239,658	120,775	199,672
HG00266	14,407,111	236,131	122,617	199,329
HG00267	14,509,546	232,802	119,396	195,698
HG00264	14,524,557	229,530	115,036	189,918
HG00265	14,506,227	233,996	119,812	195,962
HG03515	14,411,047	269,811	134,490	231,742
HG03514	14,293,542	276,542	141,696	240,256
HG00260	14,508,755	232,814	118,343	194,557
HG00261	14,412,795	234,277	119,550	197,738
NA20754	14,505,683	234,511	119,130	196,101
NA19819	14,308,714	270,729	138,696	234,987
NA20755	14,508,519	233,175	118,684	195,519
HG02589	14,285,762	279,986	144,777	242,395
NA19818	14,431,220	262,838	132,131	224,359
NA18976	14,398,577	240,387	123,595	201,282
HG01933	14,391,593	242,806	124,017	203,500
HG01932	14,513,826	230,980	118,014	193,398
HG01935	14,515,340	230,490	117,083	193,028
HG01936	14,409,081	235,639	120,960	199,054
HG01939	14,410,264	235,783	119,926	197,463
HG01938	14,523,102	226,939	116,025	190,973
HG04171	14,390,867	242,708	123,796	204,349
HG04176	14,485,982	241,222	122,494	202,854
HG04177	14,397,022	240,152	122,200	202,859
HG00982	14,487,593	238,115	122,838	202,519
HG03995	14,398,423	240,102	122,549	201,667
HG03991	14,500,481	236,897	119,991	197,809
HG03990	14,485,472	239,133	124,005	203,461
HG03999	14,478,543	241,487	124,739	206,571
HG03998	14,497,219	238,110	120,338	199,214
NA20878	14,404,229	237,640	120,785	199,165
NA20875	14,401,126	238,476	122,702	200,947
NA20874	14,398,869	240,035	122,221	202,562
NA20877	14,403,157	238,688	121,699	200,573
NA20876	14,400,278	238,998	122,031	201,782
NA20870	14,503,155	235,636	119,319	197,393
NA20872	14,406,421	237,256	121,514	199,931
HG00675	14,400,201	239,548	123,129	200,918
HG00674	14,501,605	235,719	120,041	198,591
HG00671	14,504,952	234,851	118,941	196,206
HG00672	14,412,040	235,403	119,810	196,845
NA20783	14,509,883	234,036	117,662	194,313
NA20787	14,490,033	237,927	122,522	202,210
NA20786	14,408,980	236,246	120,522	198,207
NA20785	14,506,637	233,765	119,056	196,496
NA20276	14,303,035	272,883	140,360	238,197
NA20274	14,336,516	259,903	134,944	224,684
NA20278	14,438,130	260,916	131,382	221,772
HG02002	14,513,119	231,183	117,726	193,822
HG02003	14,416,071	233,539	118,664	195,473
HG02006	14,327,252	264,706	136,320	226,428
HG02008	14,506,058	234,506	119,639	195,163
HG02009	14,392,629	274,395	139,929	238,306
HG01610	14,510,030	232,893	118,603	194,703
NA18951	14,408,792	236,176	120,500	198,365
HG01612	14,405,693	237,433	121,306	199,052
HG01613	14,406,439	237,251	121,076	198,413
HG01615	14,507,473	233,339	118,934	195,521
HG01617	14,490,041	237,062	122,735	201,778
HG01618	14,412,280	235,057	119,362	197,022
HG01619	14,503,329	234,603	120,144	196,630
NA19098	14,396,034	273,295	138,989	236,895

NA19401	14,291,190	277,029	142,462	241,400
NA19403	14,298,682	274,348	140,691	238,832
NA19404	14,293,875	276,029	141,721	240,881
HG01055	14,388,562	244,480	123,785	205,543
HG04194	14,494,335	239,092	121,383	200,204
HG04195	14,400,727	238,792	121,333	202,460
HG00189	14,508,298	233,779	119,328	195,860
HG00188	14,490,545	237,825	122,655	201,600
HG00183	14,486,855	238,230	123,766	202,826
HG00182	14,507,717	233,921	118,710	195,684
HG00181	14,504,403	235,195	119,951	197,047
HG00180	14,410,663	235,540	121,213	198,204
HG04198	14,475,269	242,720	125,644	206,609
HG00186	14,489,945	237,286	123,045	201,371
HG00185	14,504,401	235,016	119,575	196,866
NA18639	14,514,922	231,095	116,952	193,469
HG01191	14,389,930	242,268	124,436	204,613
HG01190	14,483,672	243,794	122,403	204,071
NA19625	14,311,999	270,461	138,531	233,120
HG02138	14,489,376	237,764	122,545	201,469
HG02139	14,412,043	234,929	119,527	197,048
HG02134	14,487,760	238,683	122,761	202,048
HG02136	14,410,683	234,978	120,678	197,790
HG02137	14,503,336	234,407	119,318	198,018
HG02130	14,411,000	235,896	119,196	198,309
NA19086	14,511,502	233,055	118,080	193,933
HG02133	14,407,531	236,318	119,964	198,856
NA20897	14,487,140	239,134	123,787	202,570
NA20896	14,403,826	238,265	120,557	200,515
HG00457	14,488,091	237,964	122,134	202,336
NA20894	14,399,430	239,127	122,607	201,597
HG01108	14,316,152	267,752	137,695	231,687
NA20892	14,400,250	239,782	121,997	200,885
NA20891	14,501,868	236,059	119,840	197,551
HG00452	14,409,587	236,253	119,882	198,131
HG01104	14,491,239	239,584	121,756	201,431
HG01105	14,392,521	241,025	123,924	204,903
HG01107	14,456,618	250,945	127,791	213,156
HG00458	14,412,484	234,777	119,625	196,743
NA20899	14,401,934	239,192	120,827	200,839
NA19728	14,421,187	231,104	119,728	193,585
NA19248	14,394,993	273,789	139,267	236,425
HG00351	14,493,650	236,157	122,485	201,067
HG00693	14,409,581	236,741	120,425	197,884
HG00692	14,507,326	234,048	118,747	195,188
HG00690	14,412,097	235,742	119,724	196,562
HG00356	14,411,598	235,806	120,163	197,422
HG01198	14,383,947	244,373	124,996	207,052
NA19247	14,302,952	272,585	139,749	237,409
HG00699	14,410,788	235,444	120,730	197,268
HG00698	14,488,939	237,345	122,668	201,104
NA19755	14,396,433	240,021	123,219	202,378
NA19756	14,496,753	238,860	120,125	199,839
HG03199	14,420,125	267,175	134,211	228,463
HG03198	14,295,444	275,541	141,315	239,762
NA19752	14,407,846	236,006	121,386	199,336
HG03195	14,296,084	275,213	140,788	240,619
HG03196	14,417,726	268,540	133,142	229,567
NA19758	14,402,602	237,733	122,792	198,941
NA19759	14,501,896	235,631	120,053	196,483
HG03193	14,414,413	269,841	134,692	230,386
HG03354	14,293,058	276,622	141,155	240,953
HG03352	14,415,891	268,796	134,220	230,043
HG03351	14,294,717	276,662	141,152	239,477
HG01051	14,477,640	244,995	123,931	206,672
NA18599	14,405,403	237,171	121,158	200,113
HG02220	14,413,125	235,119	119,094	197,321
HG02221	14,513,689	231,272	117,607	193,052
NA18597	14,403,828	238,145	121,324	198,802

NA18596	14,375,520	234,227	120,453	235,272
HG02224	14,494,827	235,403	122,001	199,677
NA18593	14,410,529	236,041	120,229	197,838
NA18592	14,411,680	235,569	119,740	196,531
NA19334	14,410,644	270,002	136,453	231,886
NA19332	14,329,865	268,061	133,297	229,431
HG00524	14,509,819	233,458	117,439	194,920
NA19331	14,409,305	271,581	136,367	232,235
NA18757	14,490,711	237,057	122,492	201,884
HG01031	14,506,410	233,884	118,752	196,169
NA19338	14,296,094	276,845	141,113	239,009
NA18577	14,404,110	238,164	121,975	200,044
NA18574	14,408,914	235,693	120,148	198,390
NA18573	14,407,904	237,378	120,421	197,991
NA18572	14,511,013	233,094	118,319	194,373
NA18571	14,409,445	236,312	120,395	197,762
NA18570	14,406,805	236,969	120,453	198,136
NA18579	14,407,126	236,845	120,333	198,666
HG02087	14,409,650	234,996	119,961	198,857
HG02355	14,508,793	232,873	118,406	195,361
HG02356	14,507,076	234,391	118,010	195,467
HG02351	14,506,066	234,247	118,702	196,078
HG02353	14,482,848	239,995	124,180	204,457
HG01325	14,471,583	244,547	126,253	208,729
HG01326	14,390,128	241,178	124,638	204,668
HG01323	14,379,769	245,214	126,221	208,833
HG03911	14,478,344	242,112	124,873	205,557
HG03775	14,494,428	239,527	121,918	200,336
HG03774	14,399,407	239,475	122,005	201,457
HG03777	14,484,026	240,277	123,034	203,628
HG03771	14,488,005	241,220	123,681	202,222
HG03770	14,391,074	242,779	125,054	203,900
HG03773	14,498,211	237,801	120,959	197,272
HG03772	14,393,491	241,844	124,874	202,130
HG03660	14,495,850	238,355	121,896	200,197
HG03779	14,477,726	242,895	125,373	205,856
HG03778	14,495,978	237,762	121,712	199,746
HG03175	14,410,364	271,316	135,066	232,006
HG03172	14,397,055	272,736	138,107	236,190
HG01857	14,401,596	239,420	121,966	200,140
HG01855	14,405,982	237,471	121,586	199,078
HG01852	14,505,051	235,277	118,060	195,772
HG01853	14,405,470	237,956	121,102	199,225
HG01850	14,405,386	237,711	120,819	198,944
HG01851	14,407,249	237,180	120,853	198,216
HG01858	14,405,417	237,974	120,861	198,851
HG01859	14,405,651	236,683	121,473	198,706
HG00851	14,411,156	236,211	119,276	197,004
HG03619	14,400,921	239,395	122,339	200,587
HG03611	14,394,670	242,034	122,976	203,443
HG03616	14,382,039	246,765	127,314	206,210
HG03615	14,494,262	239,141	121,751	200,365
HG02585	14,410,888	271,058	136,006	231,383
NA18979	14,409,903	236,171	119,455	198,824
NA18978	14,402,335	238,789	122,579	200,332
HG02580	14,303,785	273,600	139,278	236,340
HG02583	14,289,440	278,384	143,665	242,826
HG02582	14,390,940	274,795	141,853	239,005
NA18973	14,411,119	236,158	119,993	197,408
NA18972	14,413,811	234,997	119,285	195,971
NA18971	14,500,067	236,936	120,509	197,914
NA18970	14,502,230	236,046	119,997	196,715
NA18977	14,496,550	236,554	121,012	198,666
HG02588	14,391,255	274,979	141,138	238,596
NA18975	14,398,385	239,857	124,190	201,428
NA18974	14,498,608	236,916	121,795	197,918
NA19923	14,289,217	264,832	135,145	266,764
HG03121	14,284,045	280,214	144,755	243,630
NA20901	14,492,741	238,546	121,401	201,259

HG02049	14,393,369	241,812	124,599	202,580
HG01583	14,477,710	242,278	124,933	205,885
HG01586	14,479,832	241,587	124,339	204,513
HG01589	14,503,078	234,592	120,053	196,514
HG04206	14,496,513	238,033	120,340	199,065
HG04200	14,394,808	240,211	123,688	202,524
HG04202	14,402,215	239,405	121,788	200,469
HG04209	14,406,323	236,740	121,419	199,441
HG01097	14,495,576	238,393	121,251	200,222
HG02691	14,399,905	239,277	121,965	201,648
HG02690	14,508,617	233,590	118,667	195,004
HG02694	14,425,234	230,569	117,402	192,805
HG02697	14,391,354	242,538	125,123	202,805
HG02696	14,490,696	240,490	123,139	200,991
HG02699	14,502,773	234,498	120,146	197,176
HG01926	14,527,747	226,419	114,814	188,279
HG01927	14,423,355	231,221	118,037	192,661
HG01924	14,380,789	244,048	125,752	207,223
HG01923	14,526,372	225,638	115,168	189,734
HG01920	14,517,494	228,101	117,335	191,763
HG01921	14,419,050	232,705	118,105	194,001
HG00327	14,407,497	237,218	120,885	197,376
HG00326	14,408,370	236,092	121,804	198,573
HG00325	14,507,497	233,609	120,096	195,107
HG00324	14,378,884	232,793	119,713	234,219
HG00323	14,409,604	235,403	121,568	197,950
HG00321	14,510,227	233,147	118,188	194,240
HG00320	14,411,742	235,157	120,303	196,947
HG03604	14,397,392	240,019	123,218	202,048
HG04062	14,393,963	242,264	123,004	202,410
HG00329	14,508,219	233,953	118,762	195,192
HG00328	14,412,676	233,908	120,689	196,743
HG01536	14,594,134	235,145	119,914	197,806
HG03986	14,385,873	244,641	125,966	205,454
HG03985	14,499,964	236,459	119,542	197,911
HG03989	14,403,557	237,523	121,277	199,508
NA20868	14,396,115	241,590	123,115	202,420
NA20869	14,399,981	239,662	122,187	202,018
NA20318	14,427,635	264,335	131,839	225,295
NA20314	14,406,183	237,751	122,307	198,409
NA20863	14,482,202	241,502	124,142	204,445
NA20861	14,504,740	234,513	118,940	198,084
NA20866	14,498,925	236,160	120,887	198,703
NA20867	14,499,250	237,235	120,673	198,279
NA20864	14,498,766	237,300	120,275	198,432
HG00662	14,505,970	234,763	118,573	196,091
HG00663	14,412,814	235,320	119,877	196,695
HG01843	14,407,256	236,166	121,143	199,288
NA20790	14,410,022	235,436	120,793	198,062
NA20792	14,508,957	233,103	118,979	195,521
NA20795	14,409,342	235,837	120,557	198,544
NA20796	14,513,775	231,695	116,831	193,714
NA20797	14,408,766	235,269	121,066	198,819
NA20798	14,498,179	236,264	121,363	198,131
NA20799	14,409,554	236,140	120,567	197,850
HG03520	14,283,304	280,455	144,451	244,218
NA21137	14,387,958	244,461	125,240	204,749
NA21135	14,499,941	237,159	119,783	197,613
HG02031	14,408,689	236,118	120,212	198,729
HG02032	14,489,427	239,020	122,513	200,942
HG01915	14,296,845	275,258	140,577	240,547
HG01628	14,406,520	236,412	120,894	199,701
NA19113	14,416,576	268,183	134,231	230,628
HG01625	14,488,487	237,916	123,072	202,411
HG01624	14,508,381	233,207	118,155	195,821
HG01626	14,409,989	235,508	120,318	198,630
HG01620	14,404,554	236,915	121,633	199,191
HG01623	14,407,046	237,231	120,024	199,298
NA19439	14,300,088	274,984	139,317	238,496

NA19438	14,298,694	274,541	140,635	240,108
NA19435	14,293,287	277,023	141,801	240,513
NA19434	14,298,955	274,908	139,911	238,713
NA19437	14,293,729	275,664	142,461	241,062
NA19436	14,298,101	274,318	141,034	239,389
NA19431	14,296,498	274,611	140,394	240,296
NA19430	14,406,055	270,985	136,564	233,185
HG00190	14,508,568	232,784	119,396	195,333
NA19185	14,295,877	275,621	141,117	239,788
NA19184	14,412,978	269,644	134,631	231,332
NA19189	14,419,593	267,364	134,892	228,355
NA19065	14,406,519	237,590	120,807	198,632
HG00380	14,414,388	234,058	119,216	197,330
HG02108	14,308,684	271,097	138,948	235,681
HG02102	14,411,195	235,106	120,754	196,457
HG02105	14,428,429	228,644	117,135	191,988
HG02104	14,517,589	229,777	116,862	191,836
HG02107	14,381,106	279,323	143,125	241,482
NA20884	14,501,192	236,103	120,232	198,772
HG00443	14,408,589	236,125	120,042	198,010
NA20886	14,405,294	237,355	121,456	199,388
NA20887	14,503,519	235,681	119,798	197,411
HG00446	14,409,618	236,163	120,081	197,770
NA20881	14,399,651	238,791	122,806	201,801
NA20882	14,401,715	238,865	121,987	201,396
HG00445	14,503,640	235,496	119,267	197,150
HG01131	14,391,689	242,743	124,166	204,343
HG01130	14,495,542	238,353	121,167	199,965
HG01133	14,500,826	237,122	119,833	197,577
HG00449	14,410,770	235,429	120,235	198,160
NA20888	14,405,201	237,526	121,397	199,771
NA20889	14,503,546	235,094	119,345	197,752
HG01137	14,398,112	239,531	123,410	201,408
HG01136	14,483,618	243,151	123,328	203,056
HG00684	14,408,320	236,886	120,411	198,484
HG02968	14,413,812	270,125	134,739	230,106
HG00683	14,502,905	234,900	119,902	196,628
HG00689	14,506,018	233,687	119,464	196,334
NA19761	14,393,986	241,581	123,762	202,694
NA19762	14,490,513	240,339	122,056	200,958
NA19764	14,387,819	243,917	125,081	205,240
NA19984	14,426,356	265,795	132,583	226,667
NA19982	14,414,443	269,656	135,001	229,611
HG02215	14,411,658	235,462	120,446	197,154
NA19704	14,265,499	273,341	139,061	274,934
HG02219	14,512,634	231,978	117,916	194,976
HG03342	14,293,660	277,048	140,945	240,151
HG03343	14,414,815	269,668	133,906	228,455
NA18582	14,407,907	237,363	120,064	199,232
NA19347	14,395,114	273,915	138,487	236,083
NA18749	14,487,446	238,737	122,939	202,487
NA12717	14,403,923	237,744	122,853	200,512
NA12716	14,507,594	234,211	119,943	195,354
HG00536	14,502,008	235,579	119,891	197,044
HG00537	14,414,658	234,402	119,340	196,567
HG00534	14,408,594	236,161	120,542	198,104
NA18747	14,507,532	233,710	118,435	195,823
NA18740	14,506,119	234,507	118,910	195,595
HG00533	14,511,177	232,773	117,914	194,806
HG00530	14,488,941	237,029	123,780	201,669
HG00531	14,412,241	235,469	119,981	196,977
NA18564	14,366,784	237,584	120,967	238,058
NA18565	14,408,779	235,647	120,615	197,844
NA18566	14,409,257	235,242	120,393	198,176
NA18567	14,407,815	237,250	120,172	197,820
NA18560	14,407,674	236,834	120,511	198,029
NA18561	14,483,275	239,264	124,211	203,843
NA18562	14,508,091	234,305	117,838	195,114
NA18563	14,506,522	233,895	118,991	196,134

HG02360	14,506,713	234,579	118,073	195,446
HG02364	14,483,245	239,081	124,503	203,670
HG02367	14,508,481	234,008	117,597	195,418
HG01311	14,495,447	238,820	120,406	199,714
NA20862	14,400,565	239,232	122,443	201,312
NA12873	14,412,681	234,025	120,347	197,354
NA20317	14,307,697	271,015	139,365	234,118
HG03762	14,398,929	238,546	122,414	202,659
HG03760	14,394,955	241,163	123,372	203,456
HG03767	14,487,264	237,836	123,511	203,053
HG03765	14,397,456	239,272	123,186	202,443
HG03419	14,286,740	279,058	142,339	242,530
HG03166	14,391,772	273,562	139,134	238,425
HG03160	14,418,180	267,912	134,203	230,506
HG03162	14,294,585	275,743	141,276	240,451
HG03163	14,422,544	265,884	133,181	227,743
HG03168	14,293,163	276,679	141,656	239,527
HG03169	14,418,146	267,228	134,218	229,205
NA12342	14,508,271	233,397	118,660	194,856
HG03202	14,393,133	274,164	139,401	237,137
HG00105	14,487,028	239,169	124,473	201,801
HG02756	14,408,778	271,938	136,725	231,765
HG02757	14,285,393	280,145	144,330	242,764
NA12889	14,488,403	237,755	123,504	202,182
HG02759	14,407,888	268,575	136,906	233,308
HG00844	14,507,700	234,188	118,304	195,962
NA18968	14,401,598	239,318	123,267	200,330
NA18969	14,413,444	234,224	119,761	196,534
HG02597	14,501,064	235,490	120,289	197,986
HG02594	14,406,846	272,271	137,720	232,582
HG02595	14,287,097	278,956	144,097	243,254
NA18960	14,486,764	239,398	123,046	202,353
NA18961	14,508,259	233,869	118,713	194,497
NA18962	14,509,879	233,327	118,070	194,742
NA18963	14,371,469	235,914	120,193	236,002
NA18964	14,415,628	233,984	119,493	195,880
NA18965	14,489,984	237,372	122,254	202,092
NA18966	14,501,150	236,487	120,915	197,391
NA18967	14,493,353	236,834	121,600	199,878
NA19735	14,517,386	230,358	116,780	192,118
NA11992	14,503,130	235,026	120,476	197,277
HG03572	14,288,660	277,496	141,742	243,048
HG03571	14,411,183	270,281	134,708	230,978
HG03577	14,411,322	269,409	135,620	231,722
NA11994	14,506,907	235,281	118,578	195,259
NA11995	14,407,144	235,918	121,827	198,220
HG01405	14,483,406	242,894	124,365	203,877
HG03578	14,295,247	275,681	141,515	240,449
NA19657	14,400,442	238,544	121,821	201,034
HG04235	14,495,746	237,937	120,756	200,161
HG04239	14,476,534	242,901	125,680	206,279
HG00554	14,393,887	241,396	124,035	203,875
NA19360	14,414,928	270,319	134,491	229,337
HG00650	14,508,617	233,538	118,240	195,250
HG00556	14,510,395	232,708	117,758	194,729
HG02688	14,388,458	244,352	125,940	204,685
HG02687	14,473,616	243,538	126,726	207,540
HG02684	14,514,728	230,107	118,896	192,933
HG02685	14,400,344	239,312	123,944	200,630
HG02682	14,396,344	240,788	123,501	201,861
HG02681	14,500,361	237,330	120,288	198,387
HG03888	14,392,167	242,075	124,993	203,773
HG03882	14,389,446	242,974	125,097	204,630
HG03887	14,458,753	237,644	121,238	237,968
HG03886	14,390,265	242,820	125,631	204,605
HG03885	14,494,877	238,933	121,756	199,473
HG03884	14,398,401	239,160	124,263	201,957
HG01489	14,403,919	238,722	120,667	199,267
HG01488	14,485,089	242,383	122,821	203,720

HG01958	14,296,584	274,338	141,531	239,146
HG01953	14,506,650	235,497	120,210	194,893
HG01951	14,423,701	231,638	117,627	193,300
HG01950	14,511,128	232,588	117,655	194,785
HG01485	14,437,426	258,337	133,230	221,019
HG01956	14,296,841	275,890	141,192	239,898
HG01954	14,408,569	235,977	122,121	197,322
HG00334	14,407,916	235,369	120,950	198,516
NA20412	14,308,371	271,178	138,964	235,143
NA12155	14,487,957	238,133	123,359	202,312
HG00337	14,408,468	236,508	121,094	197,937
HG00330	14,410,589	234,837	120,528	198,011
HG03096	14,401,815	274,361	138,300	236,005
HG00332	14,406,277	237,298	121,941	198,694
NA19075	14,509,896	233,472	117,804	194,489
HG03097	14,275,139	282,890	146,568	247,675
HG00338	14,510,190	232,709	118,153	194,085
HG00339	14,411,524	235,527	120,203	197,025
HG03401	14,288,750	278,407	141,684	242,908
HG02979	14,293,457	275,897	140,961	240,411
HG00879	14,410,917	235,725	120,332	197,592
HG03099	14,286,057	279,669	145,100	242,966
HG02970	14,295,675	276,284	140,818	240,334
HG02971	14,409,509	270,860	136,196	231,352
HG03974	14,477,721	241,842	124,676	206,504
HG03973	14,402,465	238,202	121,422	201,134
HG02976	14,292,768	277,658	141,936	241,176
HG02977	14,411,168	270,619	135,371	231,503
NA18910	14,416,833	268,923	134,411	230,119
NA20321	14,304,836	273,629	139,963	236,274
NA20320	14,293,609	276,694	141,486	240,547
NA18548	14,505,102	234,601	119,216	196,011
HG03123	14,281,548	280,779	145,716	246,152
NA12234	14,402,884	238,469	122,217	199,766
HG01556	14,452,604	251,520	129,222	214,558
HG01551	14,341,085	260,200	134,144	222,987
HG01550	14,472,666	244,218	126,303	207,032
NA20529	14,408,542	237,254	120,499	197,638
NA20528	14,489,436	237,658	122,805	202,738
NA20527	14,505,865	234,471	119,176	195,866
NA20525	14,505,730	234,166	120,008	196,298
NA20524	14,508,793	232,490	118,418	195,683
NA20522	14,410,448	235,656	120,317	198,182
NA20521	14,507,677	232,120	119,548	195,604
NA20520	14,505,469	234,354	119,028	196,208
HG02028	14,413,609	235,059	119,269	197,186
HG02029	14,492,042	236,877	122,210	201,770
NA21128	14,503,616	234,848	119,443	197,586
NA21129	14,493,134	238,865	122,269	200,782
NA21124	14,493,403	238,826	122,093	199,570
NA21125	14,384,624	245,451	126,388	206,002
NA21126	14,496,893	238,276	121,566	199,562
NA21127	14,485,917	240,700	123,991	202,791
NA21120	14,398,075	239,625	122,911	202,023
NA21122	14,402,426	238,565	121,285	200,059
NA21123	14,480,845	240,825	124,604	204,561
HG01632	14,398,456	239,575	124,386	201,038
HG01630	14,504,527	234,194	119,103	197,530
HG01631	14,507,216	234,600	119,331	195,281
NA19428	14,396,037	273,101	139,223	237,196
NA19429	14,417,040	268,196	133,664	228,883
HG03511	14,325,410	269,405	134,337	230,509
HG02035	14,501,671	236,388	119,956	197,821
HG02116	14,507,558	233,846	118,672	195,217
NA19190	14,292,021	276,245	142,192	242,625
NA19197	14,290,466	277,645	142,647	241,430
HG02111	14,294,463	275,770	142,168	241,059
NA21133	14,501,200	235,938	119,517	197,988
NA19198	14,395,026	273,698	138,914	237,219

HG01124	14,477,513	244,613	125,455	206,190
HG01125	14,388,136	243,910	124,983	206,173
HG01122	14,394,798	241,039	123,828	203,482
HG01121	14,496,762	236,976	121,580	198,826
NA21130	14,481,580	240,734	123,739	203,918
NA12413	14,510,817	232,131	118,351	194,604
NA12414	14,407,890	236,880	121,032	197,905
HG00102	14,408,670	234,882	122,306	199,118
NA19779	14,389,961	243,179	124,681	204,043
NA19776	14,385,196	244,903	125,422	206,146
NA19777	14,482,664	244,050	123,982	203,500
NA19774	14,496,465	238,118	120,921	198,818
NA18748	14,509,291	233,257	118,151	195,220
NA19770	14,388,110	243,063	126,008	205,152
NA19771	14,477,646	243,570	124,979	205,080
HG03378	14,285,086	278,844	142,897	244,245
HG03370	14,414,791	268,734	134,563	230,743
HG03372	14,294,868	276,690	140,388	239,931
HG03376	14,416,632	268,594	134,242	230,745
NA20814	14,506,712	234,048	118,986	196,314
HG01075	14,491,555	239,290	120,578	201,679
NA19350	14,396,200	273,893	138,482	236,611
NA19351	14,289,348	277,450	143,190	242,468
NA19456	14,297,551	275,537	140,401	238,879
NA19355	14,291,737	277,022	142,012	240,780
HG00543	14,403,605	238,641	121,008	199,025
HG01070	14,392,866	241,850	124,037	204,149
HG03082	14,287,653	277,893	142,659	243,294
HG03081	14,412,838	269,727	134,052	232,010
NA18552	14,409,766	235,513	120,611	197,834
NA18555	14,407,467	237,604	120,412	197,921
HG01072	14,473,531	244,811	125,053	205,952
HG03085	14,272,252	283,454	147,381	246,986
HG03084	14,402,673	274,166	138,683	234,640
NA18559	14,485,585	239,048	123,988	203,097
NA18558	14,506,539	233,765	119,094	196,113
HG03088	14,292,892	276,874	141,748	241,416
NA18486	14,415,711	268,557	134,651	230,270
HG02379	14,506,427	234,572	118,586	195,016
NA18489	14,288,079	277,642	142,972	242,401
NA18488	14,293,286	277,228	141,347	241,059
HG02373	14,485,719	238,551	123,693	202,945
HG02371	14,487,553	238,945	122,075	202,058
HG01308	14,473,034	247,882	124,448	207,829
HG01305	14,485,779	242,387	122,532	204,287
HG01302	14,466,646	244,908	128,011	209,985
HG01303	14,389,929	243,810	124,214	204,538
HG03796	14,385,875	244,691	127,182	205,562
HG03793	14,389,831	242,874	124,715	203,973
HG03792	14,484,946	238,960	123,576	203,835
HG03790	14,500,084	235,754	120,073	197,867
NA18745	14,500,115	234,544	120,142	197,982
HG03157	14,413,201	269,862	135,000	231,405
HG03159	14,296,779	274,844	140,710	240,753
HG00378	14,410,402	235,537	120,440	197,018
HG00379	14,411,549	234,714	120,795	197,446
NA19451	14,420,630	267,825	133,094	227,596
HG03687	14,502,943	234,745	120,103	197,318
NA18954	14,413,163	234,636	119,435	197,414
NA18957	14,411,953	235,115	120,299	197,280
NA18956	14,408,757	236,914	120,742	198,003
HG02769	14,287,597	278,566	143,926	242,608
HG02768	14,408,229	272,132	136,487	233,098
NA18953	14,491,798	237,333	122,815	200,241
NA18952	14,485,312	239,034	123,663	202,517
HG03681	14,495,815	237,765	122,256	199,846
NA18959	14,481,466	241,203	124,500	203,357
HG02760	14,291,156	277,983	141,627	241,227
HG02763	14,306,709	271,266	138,462	235,068

HG02568	14,297,419	274,484	141,223	239,467
HG02562	14,285,537	279,592	144,909	243,979
HG02561	14,406,629	271,629	138,094	233,527
HG03833	14,497,754	237,613	120,139	198,976
HG03836	14,406,248	236,989	120,898	199,155
HG03837	14,522,623	227,603	116,073	192,075
HG00235	14,416,220	233,227	119,493	196,254
HG00234	14,509,642	231,920	118,485	194,770
HG00237	14,371,957	235,372	120,637	236,165
HG00236	14,405,178	236,877	121,881	199,481
HG00231	14,409,452	235,824	121,075	198,353
HG03565	14,410,934	270,965	134,910	232,303
HG00233	14,405,757	237,203	121,986	199,242
HG03567	14,283,356	279,771	143,219	244,041
HG00239	14,406,807	236,217	121,836	199,590
HG00238	14,408,614	236,196	122,068	198,480
HG01486	14,379,401	245,566	129,268	208,374
NA18550	14,407,570	237,226	120,297	197,905
HG04229	14,489,677	240,351	121,705	201,135
HG04227	14,394,046	241,854	123,566	204,251
HG04225	14,484,969	241,672	123,466	203,310
HG04222	14,473,719	244,491	125,318	206,446
HG03898	14,397,896	240,260	123,590	202,608
HG01497	14,483,811	241,318	124,361	202,925
HG01494	14,489,866	241,627	122,051	201,439
HG01495	14,381,810	246,637	126,560	206,864
HG01492	14,399,448	239,403	121,883	201,660
HG01066	14,496,037	238,000	120,912	199,630
HG03890	14,497,719	237,546	120,729	199,576
HG03894	14,402,143	238,482	121,538	200,955
HG03895	14,407,465	236,581	120,348	198,694
HG03896	14,503,612	235,628	120,015	196,763
HG03897	14,402,395	239,265	121,350	199,942
HG01948	14,364,520	250,183	129,596	212,306
HG01941	14,502,055	234,962	120,450	196,910
HG01942	14,383,149	231,088	119,050	232,710
HG01944	14,497,787	237,334	120,429	198,181
HG01945	14,416,623	232,910	119,034	195,218
HG01947	14,502,313	235,711	119,951	197,466
NA12144	14,509,625	232,519	118,651	195,613
HG02944	14,406,202	272,554	137,831	232,966
HG02947	14,403,666	272,414	136,126	234,123
HG02946	14,293,219	276,496	142,139	240,725
HG02941	14,388,925	275,511	141,376	239,739
HG00304	14,410,110	235,720	120,668	197,691
HG02943	14,285,785	279,722	143,971	243,958
HG00306	14,413,431	233,971	119,947	197,197
HG00309	14,410,290	235,543	120,369	198,155
HG00308	14,491,975	236,383	122,707	201,313
HG03410	14,291,160	277,279	141,759	242,178
HG02787	14,392,425	242,345	124,376	202,995
HG02786	14,493,481	239,506	121,739	200,418
HG02784	14,387,398	243,610	126,353	204,604
HG03968	14,402,248	238,275	122,144	200,763
HG03969	14,499,647	237,135	120,052	198,593
HG02780	14,510,915	233,084	117,738	194,716
HG03965	14,498,219	237,507	120,164	198,957
HG03967	14,499,953	236,129	119,559	197,295
HG03960	14,492,659	238,433	122,104	200,910
HG02789	14,491,479	240,019	122,623	201,078
HG03963	14,497,385	237,485	120,254	199,498
HG04039	14,496,541	238,275	120,571	199,018
HG04038	14,402,295	238,311	122,022	201,250
HG04035	14,398,418	239,947	122,859	202,419
HG04033	14,497,864	238,216	120,115	198,489
NA20538	14,466,202	239,863	124,253	221,936
NA20539	14,512,460	231,977	117,945	194,939
NA20534	14,486,136	238,280	124,480	203,207
NA20535	14,413,216	234,602	119,387	197,542

NA20536	14,506,023	234,100	118,964	195,790
NA20530	14,428,489	232,068	116,463	191,765
NA20531	14,409,907	235,289	119,945	198,166
NA20532	14,507,029	233,662	119,488	196,792
NA20533	14,424,259	232,221	116,796	193,870
NA21119	14,499,757	237,471	120,297	197,854
NA21118	14,497,662	237,465	120,626	199,028
NA20756	14,408,619	235,835	121,210	198,734
NA20757	14,411,009	235,320	120,829	197,565
NA20752	14,507,619	233,752	118,876	195,976
NA20753	14,404,370	237,336	122,181	200,788
NA21111	14,498,871	236,793	119,888	198,989
NA21110	14,400,970	238,937	121,734	201,352
NA21113	14,500,731	236,606	119,421	198,144
NA21112	14,479,672	241,289	124,745	204,532
NA21115	14,482,001	240,054	124,140	204,306
NA20759	14,507,127	233,327	118,828	196,006
NA21117	14,498,918	236,438	120,168	199,455
NA21116	14,500,673	237,250	119,820	198,223
NA07048	14,510,052	232,825	117,944	194,961
NA06989	14,410,937	235,067	120,073	197,483
NA12399	14,514,076	230,658	118,058	193,958
NA20334	14,316,583	268,250	138,064	231,961
NA20332	14,308,362	272,371	138,758	235,207
HG01060	14,492,718	238,478	122,587	202,018
HG00448	14,504,805	235,400	118,642	195,902
NA20339	14,298,427	274,407	140,393	239,605
HG02166	14,412,734	234,651	119,658	196,757
HG02165	14,413,182	234,446	118,763	196,818
HG02164	14,418,348	233,994	117,936	194,908
HG04001	14,404,015	237,681	121,608	200,147
NA19160	14,410,997	269,887	136,006	231,597
NA19669	14,400,989	239,387	121,477	199,509
HG01795	14,406,401	237,573	120,824	198,502
HG01794	14,411,981	235,566	119,334	197,641
HG01797	14,411,621	235,988	119,799	197,180
HG01796	14,410,298	236,419	119,998	198,009
HG01791	14,508,376	232,196	118,195	196,197
HG01790	14,414,983	234,159	120,178	196,325
HG01799	14,409,600	235,621	120,300	198,041
HG01798	14,408,761	236,323	120,433	198,010
HG03366	14,292,949	277,395	142,129	241,050
HG03367	14,414,261	269,194	134,811	230,635
HG03363	14,295,920	275,503	141,473	239,943
NA19703	14,418,323	267,048	135,311	228,867
NA19701	14,298,070	274,460	140,818	237,590
NA19700	14,420,378	267,266	134,077	227,477
NA19707	14,322,327	267,898	135,793	230,588
HG03369	14,256,604	276,136	141,013	277,694
HG01197	14,495,340	238,491	120,776	199,983
HG02778	14,398,290	239,939	122,286	201,945
HG00559	14,504,102	234,712	119,872	196,403
NA12778	14,408,478	235,057	121,459	198,582
NA19207	14,397,511	272,488	138,450	236,573
HG00551	14,379,070	245,556	126,076	206,367
HG00553	14,483,201	244,029	123,084	204,789
NA12777	14,512,885	231,870	118,660	194,941
NA12776	14,411,017	235,803	120,129	197,840
NA12775	14,491,548	235,281	123,183	201,440
HG00557	14,409,676	235,923	119,958	198,570
NA19017	14,283,724	279,986	144,693	244,040
HG03091	14,288,372	277,450	143,014	242,724
HG01879	14,363,132	271,622	137,985	272,256
NA19012	14,520,353	228,845	116,818	190,719
NA19011	14,406,356	237,291	120,397	199,178
NA18549	14,498,865	236,718	120,596	198,155
NA18546	14,505,110	234,801	119,304	196,317
NA18547	14,409,806	236,780	120,007	197,311
NA18544	14,506,754	234,728	118,323	195,795

NA18545	14,414,785	233,641	119,307	197,446
NA18542	14,407,738	236,770	120,376	198,011
NA18543	14,503,891	234,416	119,388	196,676
NA19019	14,287,029	279,532	143,489	242,755
NA18541	14,407,132	237,783	120,991	197,622
NA19385	14,408,410	272,202	136,336	232,095
NA19384	14,410,761	270,803	135,329	231,533
NA19383	14,396,748	272,797	138,596	236,702
NA19380	14,376,585	269,526	134,892	268,102
NA19076	14,510,097	232,017	118,151	194,611
HG02309	14,295,089	275,768	141,914	240,222
HG02308	14,298,465	273,748	140,784	239,824
NA18498	14,418,145	267,256	134,203	229,385
NA18499	14,289,706	278,083	142,687	242,913
HG02301	14,410,845	235,779	119,790	196,763
HG02307	14,420,985	266,717	133,131	229,304
HG02304	14,502,215	235,896	119,528	197,364
HG01378	14,400,937	239,046	122,694	200,450
HG01372	14,397,025	240,771	122,597	202,577
HG01377	14,484,231	243,394	122,582	203,396
HG01375	14,383,032	245,440	125,288	207,645
HG01374	14,504,402	234,606	119,345	197,562
NA19043	14,408,459	270,804	137,395	233,536
HG03788	14,490,462	240,367	123,013	200,741
HG03789	14,386,905	243,489	126,121	205,724
HG03598	14,394,032	241,975	122,513	202,650
HG03784	14,394,128	240,883	124,360	202,518
HG03785	14,484,392	239,534	124,260	203,118
HG03786	14,496,126	237,853	121,555	199,397
HG03787	14,386,790	244,515	125,523	205,268
HG03780	14,400,883	239,062	122,132	201,274
HG03781	14,389,485	243,197	125,580	204,925
HG03782	14,399,409	240,189	122,165	201,561
HG02410	14,498,769	237,782	121,091	198,380
HG02419	14,293,505	277,036	141,774	241,273
HG01205	14,379,319	247,437	126,850	208,327
HG01204	14,490,725	239,972	121,709	201,692
HG01200	14,504,201	236,071	119,676	196,954
HG01809	14,410,643	235,821	120,149	197,627
HG01808	14,405,836	237,057	120,966	199,614
HG01805	14,407,036	236,647	120,861	198,420
HG01804	14,408,683	236,640	119,877	198,582
HG01807	14,410,950	236,057	119,906	197,249
HG01806	14,409,268	236,415	120,159	198,654
HG01801	14,406,648	237,414	120,548	198,730
HG01800	14,415,364	233,766	118,852	195,849
HG01802	14,410,582	236,074	119,694	198,069
NA18942	14,412,866	234,974	119,538	197,171
NA18943	14,484,461	239,901	123,685	202,695
NA18940	14,508,585	233,303	118,563	194,773
NA18941	14,415,518	233,148	119,773	197,101
NA18946	14,407,157	237,181	120,496	198,210
NA18947	14,406,848	236,610	121,578	198,972
NA18944	14,505,453	235,032	118,845	196,607
NA18945	14,506,553	234,111	119,180	195,343
HG02772	14,286,453	279,319	143,558	242,981
NA18948	14,504,669	234,785	119,284	196,584
HG02771	14,407,513	272,541	136,687	233,057
HG02774	14,476,632	242,821	124,677	205,925
HG02775	14,396,466	240,323	123,725	202,547
HG02574	14,288,090	277,629	144,207	242,899
HG02577	14,299,764	274,011	140,374	236,789
HG02570	14,404,823	273,019	137,851	233,485
HG02571	14,282,337	280,197	145,112	244,672
HG02573	14,389,784	276,467	140,850	238,507
HG00956	14,406,361	237,352	121,360	199,070
HG03559	14,409,398	270,932	134,936	232,554
HG03558	14,283,873	279,854	143,376	244,610
HG03557	14,290,743	277,647	141,733	241,641

HG03556	14,411,982	270,028	135,015	231,345
HG01974	14,486,844	238,276	124,386	201,523
HG01977	14,506,736	235,054	119,182	194,232
HG01976	14,393,913	242,082	125,180	201,488
HG01971	14,368,670	251,743	129,789	210,665
HG01970	14,497,575	234,202	122,726	198,366
HG01973	14,398,902	239,211	124,720	200,235
HG01979	14,447,545	239,046	123,796	240,946
NA12154	14,510,747	232,492	118,707	194,589
HG02952	14,291,644	276,847	142,161	241,474
HG02953	14,414,327	268,519	134,906	230,721
HG00318	14,415,140	233,394	119,607	196,163
HG00319	14,410,614	235,843	120,389	198,874
HG00315	14,414,550	234,847	119,242	196,220
HG00313	14,416,181	233,334	120,198	196,145
HG00310	14,508,877	233,458	118,428	194,918
HG00311	14,490,505	236,512	124,032	201,475
HG02790	14,388,720	243,088	125,957	204,237
HG02792	14,496,886	238,311	120,542	199,704
HG02793	14,393,451	241,867	123,568	203,153
HG03951	14,394,277	241,642	122,538	203,289
HG03950	14,490,435	241,054	122,942	200,554
HG03953	14,502,559	236,369	119,201	197,867
HG03955	14,415,995	233,775	118,552	196,783
HG02799	14,284,331	280,144	144,547	243,783
NA19346	14,416,721	269,359	133,952	229,248
NA12718	14,406,536	236,665	122,175	199,123
NA19670	14,497,885	236,166	120,930	199,406
HG01571	14,509,168	232,556	118,384	195,209
HG01572	14,424,608	230,611	117,019	192,322
HG04029	14,410,333	236,006	120,540	197,764
HG01577	14,497,379	238,308	120,428	198,686
HG04025	14,403,726	238,023	120,902	199,627
HG04026	14,408,605	235,689	120,381	199,222
HG04020	14,501,276	236,132	120,012	197,519
HG04022	14,496,195	237,820	121,240	199,720
HG04023	14,503,261	235,197	119,665	197,511
NA20509	14,503,468	234,415	120,635	197,503
NA20508	14,409,786	235,836	120,668	198,299
HG01896	14,300,489	274,169	140,823	238,098
NA20503	14,400,861	239,022	123,063	200,962
NA20502	14,417,213	231,756	119,340	196,134
NA20505	14,411,378	234,670	119,714	198,592
NA20504	14,409,620	235,928	120,545	199,056
NA20507	14,407,083	236,780	121,513	199,392
NA20506	14,427,476	231,545	117,184	192,724
NA20761	14,408,258	235,705	120,602	198,169
HG03851	14,479,393	241,743	124,776	205,208
NA20763	14,509,235	232,541	118,646	194,854
NA20762	14,507,210	234,058	118,713	195,356
NA20765	14,508,633	234,101	118,323	195,444
NA20764	14,409,698	234,966	121,020	198,357
NA20767	14,504,319	233,991	119,534	196,916
NA20766	14,411,011	234,945	119,977	197,837
NA20769	14,410,300	235,549	120,163	197,445
NA20768	14,407,508	236,853	120,357	198,063
NA20291	14,392,108	275,581	139,835	237,262
NA07056	14,413,686	233,392	119,585	197,084
NA20294	14,301,799	274,698	140,542	237,732
NA12546	14,508,937	233,665	118,810	195,221
NA20296	14,307,129	271,786	139,565	236,012
NA20299	14,320,001	268,074	137,971	230,166
NA20298	14,396,108	273,493	139,071	236,626
NA12383	14,403,437	237,399	122,282	200,804
HG00149	14,508,390	232,933	119,277	195,510
HG00148	14,509,051	232,655	118,738	195,318
HG00146	14,411,325	234,928	119,743	196,564
HG00145	14,499,801	235,410	120,345	198,237
HG00143	14,508,005	233,346	118,830	195,475

HG00142	14,489,431	237,846	123,133	201,908
HG00141	14,504,366	234,218	119,489	196,286
HG00140	14,509,978	232,928	118,191	194,697
NA20342	14,429,842	264,051	132,707	223,854
NA20340	14,417,115	268,390	134,115	228,565
NA20346	14,399,348	272,653	139,148	234,967
HG01849	14,507,311	234,342	118,651	195,608
NA20348	14,405,102	271,122	137,530	233,394
HG02178	14,411,157	235,287	120,509	197,475
HG02179	14,411,383	235,259	119,634	197,407
NA19171	14,412,227	269,873	135,492	231,230
NA19172	14,292,133	276,448	142,406	241,065
HG01140	14,389,144	244,000	124,923	203,895
HG01142	14,474,210	246,383	124,610	207,212
HG01148	14,502,951	234,728	119,786	196,539
HG01149	14,388,240	244,276	124,503	204,954
HG01783	14,489,057	238,306	122,992	200,926
HG01781	14,484,633	238,805	123,730	203,658
HG01786	14,412,349	234,946	119,583	197,528
HG01784	14,402,354	238,335	122,043	200,061
HG01785	14,493,385	236,589	121,966	200,134
HG01789	14,510,012	233,319	117,997	194,747
NA21106	14,400,360	239,208	121,712	201,220
NA21107	14,499,359	236,753	119,647	198,563
NA21104	14,497,159	237,256	120,387	198,985
NA21105	14,496,762	238,213	120,652	199,515
NA21102	14,395,399	241,917	123,174	202,240
NA21103	14,396,123	240,790	123,726	201,958
NA21100	14,475,390	243,985	125,601	205,654
NA21101	14,396,162	240,700	123,342	202,152
NA21108	14,403,795	237,995	121,642	199,940
NA21109	14,479,480	240,863	125,495	204,946
HG03313	14,420,666	266,170	134,135	228,835
NA19719	14,393,898	241,901	122,365	203,251
HG03311	14,417,668	268,283	134,068	229,118
NA19711	14,422,778	266,255	132,732	228,030
NA19712	14,299,181	274,872	140,031	238,300
NA19713	14,309,371	270,962	138,635	234,069
NA19716	14,400,208	239,829	122,081	201,981
NA19717	14,497,572	238,269	120,353	198,857
HG03697	14,497,776	236,916	120,764	199,387
HG00565	14,489,825	238,005	123,405	201,948
HG00566	14,411,653	234,850	121,380	196,896
HG00560	14,411,577	234,988	120,680	197,970
HG00766	14,408,325	236,254	120,240	198,462
NA19378	14,289,466	278,038	142,569	242,448
NA19379	14,288,602	278,288	142,800	241,930
NA12760	14,508,894	233,546	118,495	195,133
NA12761	14,407,872	236,524	121,613	199,776
NA12762	14,505,677	234,604	119,293	196,060
NA12763	14,368,769	236,409	122,338	237,851
NA19376	14,393,094	274,395	139,539	238,418
NA19377	14,294,472	276,161	140,777	240,755
NA19374	14,416,018	269,116	134,845	229,867
NA19375	14,417,741	268,568	133,893	229,426
NA19004	14,505,086	235,752	119,053	195,995
NA19005	14,505,012	234,360	118,950	196,353
NA18539	14,411,160	235,831	120,364	197,357
NA19007	14,489,175	237,705	122,907	201,669
NA19000	14,502,127	235,731	119,823	196,332
NA19001	14,405,497	237,583	120,810	199,173
NA19002	14,410,358	236,261	120,190	196,393
NA19003	14,412,467	235,501	120,301	197,608
NA18533	14,406,763	236,891	120,410	198,736
NA18532	14,406,626	237,147	120,816	198,800
NA18531	14,401,913	238,768	122,720	200,341
NA18530	14,509,012	233,531	118,152	195,493
NA18537	14,408,881	236,560	120,867	197,900
NA19009	14,507,404	233,398	118,334	195,984

NA18535	14,408,089	235,752	121,144	197,971
NA18534	14,505,246	233,976	119,090	195,746
NA19394	14,415,840	268,748	134,496	229,115
NA19395	14,290,312	278,631	142,381	241,524
NA19397	14,397,660	273,069	138,496	235,455
NA19390	14,290,094	277,961	141,505	241,524
NA19391	14,291,605	278,126	141,896	241,414
HG03229	14,404,039	237,076	121,272	201,147
HG03228	14,497,739	236,961	121,378	198,841
HG03225	14,396,748	273,904	138,328	237,373
HG03224	14,390,611	274,191	139,508	238,594
NA19399	14,288,419	280,245	141,703	241,507
HG02318	14,299,198	274,148	140,019	238,614
HG02312	14,403,611	238,402	122,138	199,516
HG02314	14,415,021	268,935	134,965	230,241
HG02315	14,297,877	274,546	140,529	238,622
HG02317	14,417,031	267,495	134,268	229,559
HG01369	14,389,738	243,484	124,231	204,638
NA18629	14,508,832	233,589	117,820	195,494
NA18628	14,408,333	236,724	120,168	197,953
HG01360	14,391,067	243,223	124,563	203,278
NA18626	14,405,097	237,902	121,731	199,258
NA18625	14,412,212	235,188	119,554	197,133
HG01363	14,384,380	244,920	124,613	207,526
NA18623	14,483,487	240,666	123,136	203,041
NA18622	14,502,195	235,994	119,481	196,891
HG01366	14,379,064	246,496	126,017	208,695
NA18620	14,507,518	234,304	118,233	195,351
HG03139	14,393,430	274,321	139,123	237,590
HG02407	14,502,321	235,992	118,308	196,878
HG02401	14,488,439	238,479	122,329	202,213
HG02402	14,505,630	234,170	119,342	195,902
HG03133	14,411,982	270,646	136,565	231,463
HG03132	14,287,519	279,148	143,606	242,131
HG03130	14,420,048	266,528	134,168	228,966
HG02408	14,503,904	236,038	120,353	196,481
HG03136	14,406,568	271,796	136,760	233,307
HG03135	14,247,003	278,245	144,861	281,825
HG01312	14,387,321	243,879	124,480	205,810
HG01812	14,411,277	235,579	119,866	197,651
HG01813	14,408,906	236,631	120,388	198,513
HG01810	14,503,878	235,769	119,447	196,318
HG01811	14,510,695	233,375	117,726	194,113
HG01816	14,526,835	225,969	114,901	189,495
HG01817	14,406,579	237,261	120,337	198,682
HG01815	14,407,635	236,531	121,140	198,430
NA20885	14,499,579	237,244	119,900	198,847
HG02549	14,294,295	276,731	140,805	240,800
HG03046	14,286,267	277,595	145,513	242,301
HG03045	14,411,569	270,970	136,071	231,906
HG03040	14,288,887	278,330	143,576	242,522
HG02541	14,403,946	270,495	137,563	234,571
HG02545	14,418,477	268,075	133,326	229,244
HG03049	14,289,430	278,053	144,091	242,077
HG02546	14,300,384	274,053	139,421	238,057
HG01765	14,508,092	232,739	118,510	195,830
HG02891	14,299,161	274,498	139,842	239,437
HG02890	14,429,800	265,064	131,572	225,594
HG02896	14,307,773	272,765	139,017	236,723
HG02895	14,402,226	270,767	137,050	235,228
HG03547	14,389,911	275,689	139,886	239,251
HG01961	14,523,187	227,095	115,666	190,065
HG01967	14,478,246	241,670	126,539	204,392
HG01965	14,386,083	243,495	125,546	206,491
HG01968	14,411,988	235,547	120,813	195,838
HG03812	14,496,000	237,928	121,751	199,242
HG03436	14,415,714	268,376	134,544	230,087
HG03437	14,293,514	277,388	140,826	239,749
HG03432	14,395,029	273,330	138,505	237,165

HG03433	14,399,365	272,963	138,631	235,701
HG03814	14,397,271	239,691	122,272	202,087
HG03439	14,410,855	270,872	135,118	232,748
HG03947	14,403,018	238,423	121,322	201,123
HG00369	14,485,042	239,217	124,646	202,698
HG03945	14,403,583	238,645	120,904	200,914
HG03943	14,493,991	239,122	121,827	200,595
HG03940	14,395,116	241,045	123,524	202,634
HG03941	14,481,494	240,371	124,545	204,836
HG00362	14,407,205	236,535	122,698	198,886
HG00361	14,391,050	230,055	117,226	230,459
HG00360	14,482,119	240,079	125,243	203,747
HG00367	14,412,587	234,580	120,274	197,147
HG00366	14,510,473	232,993	118,589	194,477
HG00365	14,403,180	238,049	122,457	199,533
HG00364	14,407,075	236,752	122,044	199,650
NA11831	14,519,035	229,766	116,335	192,340
NA11830	14,415,979	233,658	119,501	196,294
NA11832	14,408,061	236,869	120,714	198,537
NA19729	14,512,438	231,794	118,398	192,215
NA20516	14,508,962	231,854	118,814	195,792
HG04018	14,397,094	238,770	123,502	202,429
NA20514	14,404,969	236,484	123,083	201,050
NA20515	14,507,586	232,765	118,950	194,665
NA20512	14,509,100	232,995	117,755	195,573
NA20513	14,505,515	235,661	119,375	196,322
NA20510	14,503,565	235,641	119,337	196,387
NA20511	14,509,831	233,110	118,163	195,112
HG01566	14,403,229	238,244	121,256	199,440
HG01565	14,507,098	233,900	119,952	195,908
HG04015	14,497,779	236,764	121,350	199,237
HG04014	14,399,934	239,346	122,196	201,956
HG04017	14,502,443	235,504	120,067	198,233
NA20519	14,510,951	232,157	118,489	195,415
NA20778	14,505,290	234,714	119,558	196,679
NA20774	14,412,125	234,444	120,521	197,994
NA20775	14,410,696	235,412	120,252	197,463
NA20772	14,408,260	236,297	120,324	198,290
NA20773	14,408,426	236,448	120,562	197,536
NA20770	14,509,156	233,010	117,921	195,494
NA20771	14,410,794	235,934	119,386	197,296
NA20289	14,297,647	274,665	142,093	238,825
NA20287	14,301,417	273,679	140,196	236,706
NA20282	14,304,209	272,600	140,672	235,573
NA20281	14,417,680	267,995	134,729	228,268
HG00158	14,410,194	235,456	120,282	197,968
HG00159	14,509,085	233,111	118,592	194,396
HG00154	14,414,023	234,605	119,932	196,081
HG00155	14,510,483	232,006	117,994	194,918
HG00157	14,502,785	234,378	120,315	198,188
HG00150	14,406,720	236,384	121,287	199,081
HG00151	14,511,991	232,038	118,019	194,317
NA20351	14,413,981	266,801	135,595	230,588
NA20355	14,302,827	273,162	139,538	236,344
NA20356	14,418,439	267,761	133,989	228,873
NA20357	14,287,837	278,370	143,435	242,602
NA20359	14,309,047	271,348	140,237	234,290
NA19149	14,298,814	274,285	140,199	239,311
NA19141	14,399,079	272,387	138,777	235,919
HG02144	14,295,847	275,219	141,173	240,543
NA19143	14,291,949	276,823	142,077	240,704
HG02146	14,521,716	227,178	116,235	190,882
HG02141	14,507,900	233,350	118,460	196,014
HG02140	14,407,809	237,048	120,055	198,151
HG02143	14,408,677	269,685	136,915	231,673
NA19146	14,396,710	272,128	139,336	236,967
HG01174	14,398,569	240,148	122,756	201,776
HG01177	14,393,914	240,865	123,230	204,769
HG01176	14,487,734	242,178	122,476	202,756

HG01171	14,369,925	249,211	127,564	210,982
HG01170	14,486,486	241,684	122,213	203,533
HG01173	14,480,372	241,582	124,535	204,751
HG01779	14,410,126	235,702	120,547	198,248
HG01777	14,505,706	234,804	119,247	196,646
HG01776	14,413,436	234,738	118,855	196,734
HG01775	14,512,100	232,335	117,276	194,942
HG01773	14,410,591	235,716	120,387	198,521
HG01771	14,502,983	236,098	120,294	197,016
HG01770	14,409,039	236,276	120,687	197,018
NA12283	14,402,307	237,938	122,853	200,380
NA12282	14,503,561	235,670	120,033	196,877
NA12287	14,412,463	235,236	119,961	196,574
NA12286	14,483,015	240,316	124,666	203,766
HG03300	14,261,502	273,521	140,331	276,864
HG03301	14,414,572	270,005	134,288	229,490
HG03303	14,291,340	276,702	142,155	241,001
HG03304	14,395,238	273,785	139,538	237,550
NA12751	14,407,018	236,850	122,829	199,720
NA12750	14,499,317	236,393	121,498	198,166
HG02090	14,494,037	238,442	121,159	201,352
HG04141	14,396,666	240,392	122,987	201,722
HG02095	14,294,689	277,177	141,489	240,506
HG00139	14,505,225	233,821	119,940	196,082
HG01682	14,506,214	233,498	119,240	196,144
HG01680	14,490,714	237,760	122,046	200,829
HG01686	14,509,985	233,189	117,955	194,300
HG01685	14,403,109	237,793	122,107	200,167
HG01684	14,406,614	237,490	121,042	198,147
NA19031	14,414,842	269,480	134,107	229,719
NA19030	14,290,153	277,628	143,907	242,340
NA12749	14,410,003	235,434	120,186	198,127
NA19035	14,417,520	268,361	134,288	229,631
NA19037	14,294,946	276,596	140,206	240,128
NA19036	14,293,201	276,555	141,735	239,748
NA18520	14,298,410	274,782	140,768	238,698
NA19038	14,289,269	278,332	141,661	240,822
NA18522	14,417,695	268,727	133,395	228,527
NA18523	14,294,049	276,361	142,597	241,168
NA18525	14,410,865	235,948	119,715	196,935
NA18526	14,410,649	235,590	119,926	198,558
HG02325	14,298,766	275,010	139,309	238,353
HG03238	14,397,944	240,667	122,367	201,652
HG02086	14,409,337	235,634	120,665	198,227
HG02323	14,425,451	265,255	132,362	228,144
HG02322	14,300,856	272,902	140,788	238,082
HG03234	14,499,713	236,721	119,813	198,963
HG03235	14,396,597	240,835	122,444	201,362
HG03237	14,500,068	236,055	120,092	198,636
HG01878	14,393,180	242,197	124,675	201,670
HG01354	14,406,010	237,015	121,263	198,292
HG01357	14,403,378	238,658	122,263	199,521
HG01356	14,492,634	240,107	121,853	200,735
HG01351	14,405,013	237,724	121,849	199,339
HG01350	14,499,421	237,043	119,765	198,512
HG01353	14,495,139	238,202	121,044	199,118
HG01699	14,506,785	233,412	118,287	196,046
HG01359	14,499,262	237,143	120,054	198,571
NA19725	14,395,724	241,167	123,600	202,561
NA19726	14,497,738	238,114	120,671	198,295
NA18638	14,505,747	234,486	119,329	196,289
NA19720	14,494,735	238,596	121,393	199,909
NA19723	14,473,516	244,693	126,306	205,976
NA19722	14,399,242	239,897	122,082	201,898
NA18634	14,406,415	236,144	121,699	199,315
NA18635	14,504,875	234,612	119,475	197,022
NA18636	14,482,942	240,795	123,728	203,650
NA18637	14,502,962	235,714	119,410	196,868
NA18630	14,413,989	234,097	119,949	196,689

NA18631	14,408,813	235,606	120,007	197,841
NA18632	14,501,192	236,469	120,237	197,818
NA18633	14,502,400	236,002	119,120	196,658
HG03129	14,294,861	275,819	142,228	239,774
HG02433	14,425,960	265,742	132,641	227,316
NA19256	14,417,612	267,777	133,902	229,418
HG02439	14,418,468	267,414	134,822	229,511
NA19922	14,425,819	264,606	133,594	225,664
NA19921	14,311,274	270,259	138,681	233,552
NA19920	14,414,837	268,710	134,967	230,868
HG03124	14,407,304	273,137	136,471	233,285
HG03126	14,292,445	277,092	141,961	241,632
HG03127	14,413,843	269,525	133,951	231,238
HG01578	14,405,331	237,420	121,149	198,948
NA19159	14,291,961	276,796	142,092	241,296
HG02557	14,415,600	269,344	134,548	229,288
HG02554	14,400,154	271,542	137,627	235,094
HG02555	14,289,182	278,572	142,582	240,998
HG02558	14,297,069	274,728	140,534	238,488
HG03054	14,406,972	272,591	135,325	232,714
HG03055	14,285,736	279,159	142,418	243,832
HG03057	14,403,973	273,175	137,681	233,538
HG03052	14,278,780	282,130	145,581	246,422
HG03058	14,277,208	282,361	147,055	246,583
HG02881	14,415,992	268,029	135,076	229,712
HG02882	14,293,587	276,775	141,362	240,147
HG02884	14,418,739	267,983	133,434	229,445
HG02885	14,298,950	274,412	140,400	239,562
HG00978	14,411,343	235,258	119,273	197,976
HG02887	14,398,965	272,653	138,440	235,727
HG02888	14,296,340	276,303	141,159	239,548
NA18858	14,294,949	275,787	140,906	239,724
HG02332	14,419,426	267,532	133,780	228,838
NA18853	14,414,336	269,717	135,729	230,865
NA18856	14,378,956	267,878	134,055	268,882
NA20760	14,409,228	235,408	121,212	197,595
HG01997	14,416,624	232,840	119,882	194,616
HG01992	14,411,826	234,539	120,624	196,354
HG01991	14,485,973	239,014	123,987	201,703
HG01990	14,418,500	267,673	133,728	230,063
HG03442	14,391,034	274,685	139,973	238,815
HG03446	14,291,050	277,176	141,701	241,716
HG03445	14,405,808	271,830	136,204	233,586
HG03449	14,292,067	276,759	142,350	241,411
HG03685	14,503,034	237,082	119,832	197,705
HG03684	14,387,409	243,651	126,124	204,240
HG03931	14,398,863	239,415	122,360	201,668
HG03686	14,498,338	236,854	119,925	199,132
HG03937	14,402,703	238,395	122,040	201,111
HG03680	14,496,606	239,113	121,227	198,963
HG03934	14,397,379	240,065	122,243	202,958
HG00371	14,488,645	237,572	123,862	202,073
HG00372	14,511,379	232,445	118,898	194,144
HG00373	14,411,102	235,094	120,646	196,945
HG03689	14,400,680	238,713	121,504	200,769
HG00375	14,507,865	234,275	119,268	195,047
HG00376	14,410,898	235,973	120,019	197,637
HG01139	14,478,513	244,259	124,256	206,725
HG02837	14,306,071	271,599	139,186	236,019
NA10847	14,403,729	237,586	123,265	200,484
HG04006	14,492,861	239,326	122,286	200,797
HG04002	14,499,994	236,447	120,344	198,295
HG04003	14,499,570	236,877	119,928	198,847
NA11829	14,487,818	238,050	123,661	202,535
NA06985	14,404,299	238,506	121,782	198,930
NA06984	14,510,601	232,279	118,459	195,365
NA06986	14,490,122	238,090	123,394	201,265
HG01519	14,414,038	234,255	120,079	197,189
HG01518	14,509,967	232,754	118,697	194,350

HG01513	14,412,727	234,924	120,212	196,050
HG01512	14,510,777	231,768	118,329	194,777
HG01510	14,409,664	235,873	121,106	198,436
HG01516	14,414,483	234,072	120,360	195,943
HG01515	14,506,551	233,906	119,318	195,375
HG03593	14,496,091	238,164	120,771	200,515
HG03595	14,397,768	239,678	122,862	202,809
HG03594	14,496,078	237,702	120,704	199,353
HG03849	14,389,671	242,757	125,554	205,208
HG03848	14,495,623	238,261	120,922	199,773
HG03846	14,494,468	238,320	121,657	200,053
HG03844	14,495,888	237,816	122,310	199,795
NA07051	14,499,531	233,980	120,657	198,568
NA18609	14,500,831	234,037	121,143	197,919
HG00160	14,495,096	235,182	121,820	200,011
NA19731	14,418,303	232,798	119,808	193,237
NA20362	14,402,443	271,925	139,052	234,267
HG01182	14,467,670	246,659	126,626	209,730
HG02152	14,411,689	235,522	119,638	196,899
HG02153	14,411,918	234,890	120,170	197,069
HG02150	14,521,764	227,687	116,083	190,404
HG02151	14,407,360	237,838	119,933	198,453
HG02156	14,411,344	235,187	120,190	197,558
HG02154	14,415,771	233,788	119,070	196,096
HG02155	14,409,920	236,066	120,330	197,861
HG01168	14,405,762	237,370	121,042	198,928
HG01162	14,391,875	243,158	123,780	203,820
HG01161	14,463,789	247,920	127,791	210,260
HG01167	14,451,140	252,284	129,628	214,720
HG01164	14,492,488	240,509	120,687	201,057
HG01768	14,366,038	237,256	121,585	237,738
NA18605	14,501,193	236,258	119,569	196,584
HG01766	14,406,105	237,060	121,196	200,243
HG01767	14,488,797	239,141	122,486	201,773
HG01761	14,499,096	237,450	121,536	198,492
HG01762	14,398,296	239,285	123,926	199,838
HG03575	14,293,167	275,884	141,402	241,163
HG02088	14,490,151	237,188	122,138	201,693
HG02089	14,407,544	237,030	121,148	198,423
HG00589	14,512,258	231,844	117,778	194,775
HG02081	14,410,378	235,738	119,719	198,281
HG02082	14,505,839	233,635	118,866	195,605
HG00584	14,412,960	234,980	119,043	196,650
HG00583	14,509,027	232,878	118,577	195,334
HG02085	14,512,036	232,970	117,112	194,021
HG00581	14,410,945	234,721	120,701	197,722
HG00580	14,502,408	234,995	120,531	196,465
HG00740	14,391,079	243,353	123,115	204,965
HG00743	14,391,804	242,312	124,184	203,915
HG00742	14,494,212	237,987	121,350	199,453
NA19152	14,282,947	280,683	144,317	244,308
NA19153	14,415,114	269,176	134,490	230,384
HG01694	14,504,370	234,900	119,334	196,628
HG01695	14,405,029	237,453	121,466	199,312
HG01697	14,409,236	236,542	120,242	198,067
NA19028	14,416,132	268,551	134,393	230,296
NA19026	14,410,738	271,095	136,244	230,918
NA19027	14,418,960	267,725	133,755	229,500
NA19024	14,286,960	279,333	143,662	242,933
NA19025	14,417,781	268,133	133,272	229,342
NA19023	14,282,169	280,130	144,480	244,996
NA19020	14,391,092	274,318	139,914	238,968
HG03209	14,393,967	274,688	138,885	237,322
HG02330	14,415,602	266,955	135,060	230,254
HG02337	14,297,776	274,270	141,856	239,101
HG02334	14,428,084	263,603	132,177	225,531
HG02339	14,300,992	274,221	140,146	238,634
HG01342	14,336,625	261,300	134,887	223,233
HG01341	14,500,291	237,502	119,566	197,944

HG01344	14,500,345	236,327	120,135	198,357
HG01345	14,397,007	240,540	122,264	202,998
HG01348	14,405,593	237,324	121,226	198,789
NA19732	14,520,093	228,521	117,110	190,768
NA18608	14,504,493	235,177	118,830	196,730
HG01187	14,482,048	242,739	123,566	205,026
NA19734	14,408,105	236,210	121,132	198,302
HG01183	14,387,357	244,147	124,560	205,486
NA18603	14,497,360	236,244	120,545	198,467
NA18602	14,410,356	235,776	120,639	197,674
HG01188	14,361,749	254,301	128,887	214,261
NA18606	14,511,466	233,081	117,488	194,428
HG03115	14,406,025	273,055	137,861	232,676
HG03114	14,284,846	278,733	144,225	242,718
HG03117	14,288,138	278,266	142,364	242,512
HG03111	14,292,505	277,244	140,717	240,248
HG03112	14,394,507	273,729	139,396	237,312
HG03673	14,387,163	244,034	126,330	205,214
HG03118	14,396,271	273,606	138,462	236,696
HG02429	14,399,223	272,136	137,532	235,755
HG02427	14,300,051	274,177	140,397	237,875
HG02425	14,387,774	242,609	126,566	206,020
HG01440	14,478,093	245,218	123,938	205,592
HG02420	14,432,428	262,101	132,093	223,896
NA18517	14,296,268	274,782	141,171	240,405
NA18516	14,411,930	269,577	135,547	231,028
NA18511	14,293,249	276,729	141,481	241,273
NA18510	14,394,314	273,720	139,655	237,555
NA19175	14,417,097	268,064	134,503	230,467
HG01134	14,389,690	243,267	124,155	204,691
NA18519	14,409,503	270,825	136,098	231,727
HG01443	14,501,855	236,276	119,579	196,420
NA18999	14,412,523	234,794	119,460	197,544
NA18998	14,371,269	235,478	120,292	236,742
HG02521	14,507,110	233,444	118,844	195,737
NA18991	14,411,877	234,906	120,516	198,173
NA18990	14,505,248	233,133	119,103	196,363
NA18993	14,412,415	235,680	119,100	197,522
NA18992	14,426,618	230,574	115,912	192,433
NA18995	14,492,095	236,747	122,395	200,446
NA18994	14,514,426	231,475	117,520	193,446
NA18997	14,413,258	235,250	119,131	196,430
HG03061	14,295,522	276,289	139,684	240,418
HG03060	14,412,297	270,151	134,664	231,341
HG03063	14,412,936	270,590	134,061	231,057
HG03064	14,286,737	277,886	143,115	244,479
HG03066	14,409,627	271,145	135,596	232,847
HG03069	14,409,140	270,779	136,021	232,188
NA11920	14,404,107	238,122	121,826	200,195
NA18865	14,397,136	272,278	138,536	237,679
NA18864	14,289,506	277,789	141,473	241,393
NA18867	14,294,984	276,214	141,735	240,412
NA18861	14,293,565	276,705	141,147	240,937
HG03291	14,294,198	275,772	142,427	241,327
NA18868	14,408,420	271,633	136,711	232,121
NA19116	14,298,559	274,454	140,693	239,571
HG02635	14,285,083	279,909	145,032	242,785
HG02634	14,410,483	271,018	136,371	232,007
HG00442	14,505,048	234,731	118,534	195,437
HG01865	14,487,398	238,772	122,673	202,045
HG01988	14,422,436	262,857	134,813	225,539
HG01989	14,306,088	271,346	139,286	236,689
HG01985	14,292,987	278,282	140,031	239,628
HG01986	14,419,281	267,855	133,145	227,581
HG01980	14,396,962	240,632	124,249	200,694
HG01982	14,513,438	231,351	117,677	193,772
HG00345	14,510,593	233,147	118,154	194,997
HG03451	14,405,322	271,754	137,409	234,575
HG03452	14,277,916	281,094	145,232	245,794

HG00346	14,412,749	234,524	120,636	196,377
HG00341	14,504,534	234,522	119,972	196,170
HG03455	14,287,212	278,674	142,246	243,547
HG00343	14,414,215	233,909	119,986	197,643
HG03457	14,406,693	271,813	136,722	232,898
HG03458	14,292,437	277,192	141,761	242,246
HG00349	14,414,250	233,488	119,368	196,648
HG03692	14,392,880	242,394	124,811	202,946
HG03693	14,486,338	242,209	123,594	202,193
HG03922	14,390,191	242,709	125,514	205,525
HG03691	14,499,403	236,626	120,286	198,517
HG03696	14,522,557	227,468	115,848	190,182
HG03925	14,397,490	240,595	122,258	202,542
HG03926	14,495,292	238,315	121,672	200,132
HG03695	14,485,228	238,735	123,417	203,825
HG02981	14,410,770	269,759	134,917	231,509
HG03698	14,401,258	238,578	121,782	201,443
HG02982	14,415,002	269,223	134,504	230,472
NA19372	14,415,044	268,704	133,871	230,354
NA10851	14,494,040	236,588	121,672	200,571
NA06994	14,505,819	233,942	120,076	196,670
HG04070	14,425,641	229,459	117,035	192,573
HG04076	14,396,870	240,241	123,291	202,072
HG04075	14,401,723	238,222	122,363	200,746
HG01509	14,486,667	238,833	123,945	203,431
HG01500	14,506,386	234,751	118,761	196,385
HG01501	14,410,440	234,588	120,744	198,376
HG01503	14,510,169	233,082	117,876	194,853
HG01504	14,407,146	236,959	121,404	198,562
HG01506	14,509,615	233,531	118,388	195,225
HG01507	14,412,079	235,236	119,875	198,113
HG02855	14,297,042	275,094	141,449	239,900
HG02854	14,413,546	269,763	134,492	230,442
HG03858	14,400,345	238,545	122,462	201,218
HG02852	14,299,458	274,567	139,456	238,470
HG02851	14,412,878	269,545	134,993	231,567
HG03589	14,400,280	238,893	121,678	202,292
HG03854	14,504,075	235,086	118,973	197,188
HG03856	14,502,758	235,332	119,045	197,813
HG03857	14,393,883	242,425	122,747	203,267
HG03850	14,502,194	235,862	119,753	197,760
HG03583	14,288,523	278,151	141,849	242,614
NA12156	14,412,072	234,871	120,398	197,328
NA18949	14,411,243	235,469	120,227	197,218
HG00336	14,486,921	237,951	123,690	202,378
HG00176	14,400,063	239,054	123,424	200,565
HG00177	14,412,970	233,932	121,539	196,966
HG00174	14,410,564	235,711	120,579	197,830
HG03875	14,485,897	238,784	123,493	202,705
HG00173	14,410,711	235,401	121,364	198,284
HG00171	14,408,165	235,924	121,892	197,459
NA07000	14,410,659	234,955	120,204	197,890
HG00178	14,409,658	235,278	121,637	197,715
HG00179	14,406,892	236,365	121,810	199,014
HG01435	14,393,365	241,868	124,078	203,450
HG01437	14,504,493	234,653	119,520	196,373
HG01431	14,488,638	240,551	122,364	202,773
HG01432	14,408,251	236,374	120,507	198,852
HG02522	14,404,959	238,278	121,001	199,164
HG04107	14,497,978	238,035	120,738	199,000
HG04106	14,392,537	240,515	125,218	202,683
HG01438	14,416,878	233,428	119,228	195,875
HG04100	14,494,343	238,468	121,312	200,663
HG03120	14,408,506	271,384	136,612	232,377
HG02084	14,409,117	236,522	119,890	198,425
HG02383	14,487,228	239,004	122,863	202,337
HG03259	14,312,819	268,227	139,301	233,417
HG02386	14,449,531	237,169	123,740	240,485
HG02385	14,492,271	235,711	122,664	201,226

HG02384	14,515,665	232,586	116,051	193,633
HG01757	14,394,494	242,301	123,944	201,432
HG01756	14,492,617	239,766	122,595	199,722
NA19239	14,415,178	269,033	134,086	229,743
NA19238	14,298,161	275,550	140,301	239,548
NA19236	14,410,789	270,176	136,047	231,287
NA19235	14,294,380	276,635	141,213	240,442
HG03978	14,470,923	244,503	127,036	208,375
HG03977	14,396,080	240,281	122,548	202,606
HG03976	14,497,725	237,325	120,682	199,318
HG02973	14,368,770	273,052	136,634	270,187
HG00598	14,485,125	239,461	124,439	203,507
HG00599	14,400,338	238,794	123,777	200,620
HG00595	14,505,098	235,323	119,147	195,717
HG00596	14,414,141	234,137	120,514	196,768
HG00590	14,411,248	235,671	120,570	196,942
HG00592	14,489,496	237,645	122,733	202,017
HG00593	14,407,122	237,425	121,587	198,680
NA19121	14,415,715	269,058	134,728	229,909
HG03971	14,479,006	241,557	124,915	205,266
HG00759	14,405,908	237,114	120,933	198,870
NA19129	14,296,025	275,214	141,333	240,306
NA19059	14,410,248	234,972	120,786	197,417
NA19058	14,510,802	233,744	117,839	194,189
HG03212	14,288,584	277,661	142,149	242,352
NA19057	14,411,665	235,831	119,507	197,775
NA19056	14,511,544	232,327	117,436	194,273
NA19055	14,505,736	235,209	119,026	196,380
NA19054	14,407,369	237,849	121,010	197,747
NA19835	14,298,105	275,121	140,163	238,728
NA19834	14,426,304	265,417	132,259	225,406
NA18616	14,408,201	236,372	120,715	198,144
NA18617	14,408,300	236,978	120,472	197,741
NA18614	14,409,052	236,782	120,094	198,452
HG02182	14,409,410	235,844	120,477	197,939
NA18612	14,503,859	234,800	119,173	196,205
HG02184	14,414,449	234,344	119,878	196,145
HG02187	14,410,430	236,966	120,164	198,251
HG02186	14,416,263	233,919	118,918	196,243
HG02188	14,414,919	234,437	119,393	196,314
NA18618	14,412,020	235,316	120,924	197,599
NA18619	14,411,900	234,842	120,657	197,853
NA21089	14,397,735	240,768	122,713	200,899
HG03103	14,394,354	272,692	139,663	236,816
HG03100	14,391,802	274,477	140,904	237,915
NA19901	14,292,325	277,410	142,062	239,653
NA19900	14,428,881	263,639	131,847	226,362
HG03105	14,292,897	277,134	141,206	240,954
HG03108	14,283,636	279,234	145,866	243,805
HG03109	14,391,817	275,266	140,909	238,293
NA19909	14,307,587	272,480	137,972	235,089
NA19908	14,419,998	267,046	133,761	227,632
HG02450	14,289,952	277,200	142,842	241,307
HG02455	14,422,629	266,717	133,765	226,634
NA18502	14,296,469	274,960	141,401	240,005
NA18501	14,415,773	270,135	134,490	230,347
NA18507	14,412,404	269,151	135,586	231,355
NA18504	14,414,361	269,907	134,009	229,793
NA18505	14,291,316	277,750	142,094	241,300
HG01242	14,323,125	265,251	137,082	229,044
NA18508	14,292,571	277,808	140,769	241,817
NA19080	14,412,171	235,021	119,455	196,348
HG01247	14,480,238	244,608	122,745	205,128
NA18986	14,484,023	240,672	124,410	202,910
NA18987	14,418,740	232,708	118,154	194,289
NA18984	14,512,385	232,347	117,750	193,447
NA18985	14,490,455	237,078	122,150	201,636
NA18982	14,506,618	234,642	118,518	195,900
NA18983	14,502,355	235,898	119,840	196,940

NA18980	14,409,256	236,625	120,621	198,229
NA18981	14,407,941	237,604	120,345	198,472
NA12827	14,492,434	236,520	123,481	201,063
NA18988	14,501,308	237,026	120,021	196,358
NA18989	14,481,853	240,525	124,877	203,538
HG00732	14,385,411	244,747	126,445	204,226
HG02536	14,422,113	266,687	133,783	227,153
HG02537	14,285,417	280,429	142,700	243,226
HG03078	14,401,531	273,613	138,232	235,170
HG03079	14,288,281	277,466	143,326	243,822
HG03077	14,370,625	270,788	135,297	271,065
HG03074	14,407,341	271,492	136,287	233,213
HG03072	14,412,867	270,141	134,534	231,552
HG03073	14,284,875	278,820	143,264	244,884
NA11930	14,482,627	240,369	125,035	203,117
NA11931	14,412,099	234,355	120,689	197,288
NA11932	14,503,002	236,032	120,433	196,736
NA11933	14,406,764	236,942	121,650	198,562
NA18873	14,292,869	275,994	142,068	241,019
NA18870	14,286,956	278,503	143,908	244,407
NA18871	14,414,464	269,166	134,940	229,889
NA18876	14,294,908	276,108	140,928	240,441
NA18877	14,412,187	270,001	135,260	231,280
NA18874	14,416,502	268,332	134,294	230,492
NA18878	14,293,843	276,512	141,610	240,561
NA18879	14,414,566	268,611	135,064	230,940
HG00864	14,413,866	234,509	119,424	196,945
HG02628	14,408,479	272,081	136,998	231,987
HG02629	14,283,366	279,434	145,467	244,615
HG01848	14,406,563	236,416	121,303	199,083
HG02624	14,284,070	279,689	145,044	242,971
HG02620	14,409,705	271,657	136,394	232,121
HG02621	14,282,870	281,152	144,708	244,386
HG02623	14,410,627	271,485	136,548	231,968
NA20758	14,485,762	239,276	123,455	203,842
NA18950	14,403,217	238,860	121,573	199,798
HG02974	14,283,204	280,473	145,039	243,761
NA19006	14,504,557	234,958	119,039	197,430
HG00353	14,411,710	234,582	120,201	197,539
HG00350	14,417,201	233,173	118,990	196,346
NA18538	14,409,217	236,772	120,754	198,067
HG03469	14,397,910	274,902	138,628	235,315
HG00357	14,414,787	233,800	120,060	196,302
HG00355	14,414,983	232,668	119,948	196,501
HG03464	14,275,238	282,571	146,515	246,893
HG00358	14,494,319	234,850	121,917	200,117
HG03461	14,293,291	277,074	140,920	241,649
HG03460	14,408,922	270,647	135,453	232,509
HG03919	14,397,634	240,181	122,108	202,240
HG03668	14,394,502	240,734	123,805	203,559
HG03667	14,495,719	237,514	121,482	199,790
HG03914	14,482,285	240,942	123,733	203,926
HG03917	14,479,019	241,067	124,758	206,020
HG03916	14,400,487	239,073	122,140	201,021
HG03663	14,493,108	239,837	121,853	201,110
HG03910	14,400,845	238,450	121,822	200,952
HG03913	14,398,723	239,582	122,359	200,998
HG03600	14,494,912	238,471	120,907	200,188
HG01847	14,409,312	235,507	121,447	198,002
NA18536	14,508,339	233,218	118,383	195,593
HG04060	14,493,008	238,012	122,078	199,782
HG04061	14,496,971	238,295	120,678	199,163
HG01537	14,404,326	237,877	122,431	199,883
HG04063	14,402,876	238,667	121,798	200,439
HG01531	14,404,932	237,525	122,475	200,307
HG01530	14,503,375	235,930	120,366	196,659
NA11840	14,407,408	236,092	121,539	198,644
NA11843	14,486,503	238,220	123,393	202,150
NA12003	14,510,614	232,912	118,809	195,364

NA12005	14,510,125	232,872	118,466	194,362
NA12004	14,407,176	235,678	121,149	199,421
NA12006	14,409,065	236,171	120,914	197,823
NA20544	14,504,586	233,740	119,663	196,725
NA20541	14,409,356	235,408	120,117	197,625
NA20540	14,417,479	233,285	119,199	195,579
NA20543	14,501,206	235,662	119,744	198,333
NA20542	14,408,605	236,515	120,453	199,052
HG03869	14,494,439	239,102	121,814	199,892
HG03868	14,392,035	241,349	124,917	203,775
HG02840	14,298,722	274,533	141,033	239,220
HG03861	14,401,419	239,177	121,815	200,492
HG03863	14,401,713	239,280	121,462	201,135
HG03862	14,408,122	235,491	122,292	199,026
HG03864	14,492,716	238,612	122,723	200,812
HG03867	14,484,621	239,269	123,370	204,615
HG03866	14,504,814	235,353	118,782	197,523
NA19393	14,407,113	271,335	137,181	233,060
HG00103	14,504,414	234,470	120,041	196,568
NA12347	14,507,223	233,528	119,059	195,961
HG00101	14,488,080	237,357	124,938	202,803
HG00100	14,404,502	238,141	121,894	198,428
HG00107	14,492,238	236,190	123,790	200,594
HG00106	14,404,344	237,065	122,547	199,473
NA12340	14,510,805	232,175	118,362	194,239
NA12341	14,408,144	236,081	122,058	198,736
HG00451	14,510,766	232,653	117,676	194,702
HG00109	14,489,714	237,586	122,308	201,954
HG00108	14,509,789	232,897	118,688	194,557
NA12348	14,408,752	236,084	121,714	198,348
HG01403	14,381,616	245,490	125,982	208,495
HG01402	14,481,761	243,050	123,353	204,858
HG04118	14,397,347	240,518	122,424	201,967
HG03485	14,290,470	276,922	142,126	242,320
HG03484	14,408,291	271,087	135,431	232,359
HG02023	14,503,698	235,500	119,426	196,779
HG02020	14,510,357	233,590	118,119	194,344
HG03488	14,400,615	238,946	121,407	202,341
HG02026	14,503,078	235,823	119,831	197,313
HG01746	14,410,670	235,486	120,571	197,053
HG01747	14,488,791	237,082	123,123	202,104
NA20819	14,406,331	236,031	121,204	199,043
NA20818	14,412,621	234,490	120,078	197,677
NA20815	14,507,753	233,465	118,598	196,192
HG02025	14,403,528	238,215	122,276	199,910
NA20813	14,409,638	235,582	120,873	198,081
NA20812	14,487,767	237,702	122,892	202,920
NA20811	14,509,376	233,582	118,122	194,883
NA20810	14,498,427	236,137	120,576	198,561
HG00619	14,508,036	233,769	118,882	194,656
HG00614	14,410,601	236,003	120,028	197,463
HG00613	14,507,206	233,283	119,063	195,335
HG00611	14,409,788	236,275	121,037	197,806
HG00610	14,506,775	233,607	119,042	195,622
HG03380	14,291,955	276,783	142,046	242,076
NA21142	14,400,374	238,859	121,733	200,788
NA21143	14,393,222	241,531	123,357	203,525
NA21141	14,398,272	240,131	122,246	201,598
NA21144	14,398,256	239,218	122,910	202,458
NA19225	14,296,671	276,233	140,143	239,630
NA19222	14,288,194	278,708	142,694	242,058
NA19223	14,415,693	268,593	134,683	230,832
NA19130	14,413,379	269,008	134,735	230,512
NA19131	14,279,593	281,258	145,490	244,585
HG00729	14,399,371	239,465	123,298	200,597
HG00728	14,485,291	239,790	123,792	203,476
NA19137	14,288,455	277,999	143,151	242,237
NA19138	14,412,652	270,311	135,152	230,810
HG01598	14,408,369	236,105	120,442	198,586

HG03267	14,291,534	276,948	141,631	241,492
HG01047	14,497,694	238,072	120,798	199,295
HG03265	14,415,364	269,445	134,087	230,545
NA19041	14,397,106	272,602	138,486	236,088
NA19042	14,293,440	277,503	141,420	240,905
HG03268	14,397,029	272,362	138,892	237,248
NA18627	14,408,507	236,642	120,785	198,155
NA19096	14,418,383	268,138	133,859	229,396
HG01362	14,461,936	246,663	127,819	212,568
NA18624	14,485,872	238,616	123,790	203,254
HG00344	14,414,757	233,339	120,332	196,813
HG02190	14,408,908	236,386	120,300	198,144
HG01365	14,480,757	243,291	123,805	204,931
HG00437	14,407,542	237,053	120,100	198,829
HG00436	14,491,627	236,533	122,236	201,216
NA18621	14,485,423	239,685	123,333	203,191
HG00342	14,488,243	238,214	123,139	201,493
NA19913	14,299,226	274,125	140,201	236,934
NA19916	14,418,634	267,552	133,918	229,321
NA19917	14,294,976	275,890	141,337	239,995
NA19914	14,307,879	271,520	138,593	234,508
HG02449	14,396,993	272,270	140,062	236,348
HG02442	14,418,815	267,752	134,288	228,547
HG02445	14,426,966	264,366	133,104	226,254
HG01259	14,502,130	236,138	120,153	197,787
HG01250	14,492,228	239,835	122,051	201,581
HG01251	14,395,667	240,558	123,566	202,560
HG01253	14,493,338	239,214	121,929	200,918
HG01254	14,363,305	250,293	130,716	214,002
HG01256	14,497,525	237,786	121,286	198,711
HG01257	14,394,381	241,135	123,987	202,516
HG03920	14,494,458	238,761	120,957	200,094
HG04216	14,393,544	241,312	123,488	203,373
NA12830	14,403,056	238,779	122,362	200,139
HG03690	14,424,844	231,196	116,911	193,614
HG03189	14,288,920	278,310	142,213	241,972
HG02508	14,293,701	276,279	141,509	240,641
HG02505	14,295,582	275,404	142,154	239,511
HG02501	14,426,983	265,396	132,470	225,582
HG02502	14,300,378	274,191	141,207	237,016
HG03009	14,496,362	237,368	120,887	199,898
HG03694	14,494,553	237,959	121,369	200,420
HG00269	14,405,728	237,755	121,963	197,717
HG03007	14,391,958	241,809	124,746	203,692
HG03006	14,472,825	243,946	126,564	207,836
HG03928	14,398,856	240,394	122,021	201,421
HG02983	14,297,677	275,467	140,083	238,563
HG00290	14,484,397	239,651	125,118	203,505
HG00262	14,414,697	235,096	119,011	195,680
HG00263	14,410,596	235,725	119,915	196,768
HG03517	14,295,493	275,383	140,888	240,315
NA18881	14,284,979	279,075	144,963	243,660
HG02113	14,412,939	234,606	119,263	196,980
HG03717	14,391,234	242,990	124,663	203,258
HG03716	14,473,880	243,853	126,169	207,184
HG03714	14,391,135	242,196	124,954	203,989
HG03713	14,491,057	240,029	122,747	201,496
HG03711	14,484,791	239,208	123,369	202,625
HG02611	14,286,293	279,007	144,669	243,306
HG02610	14,410,821	270,913	136,210	232,464
HG02613	14,416,290	268,859	135,087	230,053
HG02614	14,287,666	277,884	145,009	243,632
HG03718	14,484,082	239,538	123,539	203,644
HG02406	14,509,405	233,206	118,330	195,001
HG03478	14,407,979	271,337	135,748	233,231
HG03479	14,286,867	277,797	142,580	243,208
HG03472	14,404,548	273,163	137,595	234,191
HG03473	14,288,638	278,710	141,725	242,593
HG03470	14,285,452	278,656	142,910	244,144

HG03476	14,286,834	278,415	142,768	243,595
HG03908	14,473,103	243,987	125,955	207,096
HG03679	14,495,288	239,403	121,423	199,734
HG03902	14,484,315	240,758	123,340	203,495
HG03900	14,480,728	241,190	124,851	204,639
HG03907	14,399,983	238,682	121,726	202,473
HG03672	14,499,809	237,753	119,541	197,281
HG03905	14,498,419	236,905	120,775	199,306
HG02409	14,504,692	234,891	119,081	196,801
HG01522	14,411,391	235,154	120,807	196,549
HG01085	14,483,809	241,775	123,662	203,913
HG01521	14,509,121	232,711	120,014	195,292
HG04059	14,395,878	240,642	123,398	202,985
HG01527	14,492,583	239,033	122,575	200,219
HG01524	14,505,138	234,617	119,818	196,787
HG01525	14,403,423	237,779	122,559	200,272
HG04054	14,400,438	239,273	122,285	201,074
HG01528	14,361,961	239,450	123,194	238,370
HG04056	14,514,494	231,095	117,387	193,993
HG03874	14,385,658	244,442	126,028	205,534
HG02054	14,296,440	275,748	141,007	238,695
HG03872	14,498,290	237,029	120,688	199,329
HG03873	14,395,571	240,766	122,722	203,153
HG03870	14,481,968	240,048	123,852	204,887
HG03871	14,493,441	238,934	122,525	200,635
HG02052	14,290,918	277,255	142,936	242,311
HG02870	14,294,693	276,273	141,688	239,762
HG01083	14,387,175	244,117	125,320	206,212
HG02879	14,287,573	278,292	145,196	244,250
HG02878	14,392,177	275,343	139,942	238,201
HG01082	14,476,705	246,233	124,065	207,246
HG00110	14,415,475	233,847	118,968	195,864
HG00111	14,410,983	235,360	121,016	197,335
HG00112	14,511,338	231,889	118,795	195,239
HG00113	14,511,738	232,119	117,723	194,453
HG00114	14,469,695	233,329	119,710	233,436
HG00115	14,507,732	233,603	118,846	196,498
HG00116	14,508,244	233,234	118,948	195,364
HG00117	14,510,898	233,857	117,944	192,990
HG00118	14,405,189	237,392	122,250	199,588
HG00119	14,487,062	237,875	123,584	202,694
HG01414	14,385,776	244,538	124,072	206,922
HG01412	14,484,851	242,875	124,459	204,258
HG01413	14,487,877	241,437	121,875	202,154
HG03490	14,507,609	233,620	118,239	195,758
HG03491	14,397,672	239,691	122,761	202,023
HG03499	14,293,779	276,920	141,870	239,829
HG02397	14,488,247	237,910	123,226	201,905
NA12489	14,408,969	236,486	120,567	197,772
NA20809	14,509,068	232,760	118,431	195,023
HG04173	14,497,236	237,851	121,115	198,887
NA20804	14,410,272	235,268	119,843	197,833
NA20805	14,501,534	234,768	120,088	197,213
NA20806	14,487,797	237,926	123,004	203,053
NA20807	14,412,702	234,556	119,587	197,627
NA20800	14,415,463	233,653	119,462	196,423
NA20801	14,488,041	237,752	122,926	202,182
NA20802	14,408,332	235,949	120,493	197,835
NA20803	14,491,263	237,062	122,354	201,445
HG00608	14,412,063	235,042	119,559	196,459
NA12249	14,415,045	234,054	120,292	195,760
HG00607	14,490,639	237,377	122,723	200,456
HG02299	14,516,256	231,031	116,894	191,902
HG02298	14,405,684	237,774	122,097	199,347
NA19210	14,409,572	271,026	136,311	233,214
NA19213	14,412,090	270,387	135,121	231,145
HG02291	14,514,876	230,871	117,680	191,690
NA19214	14,292,113	276,803	141,234	240,803
HG02292	14,418,235	232,474	119,751	194,019

HG01089	14,382,755	245,441	126,265	207,265
HG01088	14,480,517	243,702	123,805	205,905
HG02058	14,500,180	237,102	120,430	197,446
HG02057	14,393,743	242,330	124,032	202,186
HG01086	14,389,253	243,129	124,266	204,790
HG02053	14,411,082	270,328	134,753	231,018
NA19107	14,415,113	268,991	133,897	230,478
HG02051	14,414,697	269,108	134,629	230,513
HG02050	14,507,107	233,981	118,559	196,035
NA19454	14,389,762	275,162	140,656	237,580
HG02147	14,420,433	232,570	118,835	193,257
HG00739	14,500,655	236,815	119,544	198,706
NA19108	14,295,687	275,120	141,326	240,697
NA19457	14,295,347	276,134	141,659	240,429
HG00731	14,494,342	238,428	122,225	200,072
NA19455	14,419,218	267,777	133,169	229,373
NA19102	14,284,277	279,505	144,386	243,206
HG00734	14,378,531	246,460	126,789	208,656
NA19452	14,417,760	268,019	133,691	229,119
HG00736	14,478,457	241,440	124,875	205,450
HG00737	14,385,687	244,452	125,186	205,599

Rare and Common Variants in 1000Genome data

Chromosome	Rare Variants				Common Variants			
	**SNP**	**INS**	**DEL**	**MAC**	**SNP**	**INS**	**DEL**	**MAC**
1	236,232	3,575	5,480	1,228	67,276	8,180	10,612	1,910
2	273,255	3,751	6,699	1,534	76,700	8,998	12,735	2,425
3	223,547	3,301	5,576	1,249	64,811	8,297	10,978	2,100
4	249,922	1,991	4,561	1,004	65,473	3,840	5,523	1,035
5	205,315	3,361	5,244	1,220	59,263	7,801	10,410	1,911
6	191,183	2,870	5,174	1,055	57,744	6,928	9,524	1,840
7	174,749	2,745	4,261	810	53,069	6,178	8,542	1,389
8	171,643	2,476	4,184	925	49,148	5,441	7,194	1,515
9	128,810	1,815	2,846	574	36,960	4,349	5,841	1,083
10	143,404	3,225	1,972	708	43,093	4,903	6,769	1,280
11	145,849	2,054	3,397	804	43,272	4,926	6,704	1,330
12	142,612	2,321	3,762	855	42,966	5,266	7,278	1,331
13	117,271	1,995	3,347	643	34,622	5,000	6,470	1,266
14	97,928	1,564	2,372	594	29,010	3,787	4,726	916
15	85,535	1,290	2,058	437	24,484	2,839	3,942	646
16	87,690	935	1,766	498	24,392	2,721	3,292	663
17	77,265	1,002	1,704	413	21,693	2,419	3,410	524
18	87,754	1,258	2,108	526	25,720	3,302	4,306	739
19	54,126	695	1,129	344	16,925	1,640	2,228	545
20	61,133	921	1,464	320	17,652	1,760	2,550	546
21	41,789	676	1,056	271	12,354	1,469	2,167	487
22	33,643	493	740	177	10,208	949	1,404	222
X	150,487	3,431	7,273	1,260	33,168	3,254	4,568	1,170
Y	3,344	37	58	0	346	5	11	0
**Total**	3,184,486	47,782	78,231	17,449	910,349	104,252	141,184	26,873
