# Supplementary material for: A reference catalog of DNA palindromes in the human genome and their variations in 1000 Genomes
Source: Hum Genome Var. 2020 Nov 20;7:40. doi: 10.1038/s41439-020-00127-5 (PMC7680136; doi:10.1038/s41439-020-00127-5)
Supplement: Supplementary file 5 — Supplementary Table 5 [file 41439_2020_127_MOESM5_ESM.pdf]

Chr	Ref_mid	Ref_len	Ref	Alt	Position	AC	DISEASE/TRAIT	REGION	CHR_POS	GENE	SNPS	CONTEXT	P-VALUE	PalindromeChange
6	32282854	16	A	G	32282854	525	Rheumatoid arthritis	6p21.32	32315077	C6orf10, LOC101929163	rs6910071	intron_variant	1.00E-299	Perfect_to_near
6	6318784	24	C	A	6318795	740	End-stage coagulation	6p25.1	6318562	F13A1	rs5985	nissense_varia	3.00E-186	Non_identical
6	32663999	18	G	C	32663999	2678	Rheumatoid arthritis	6p21.32	32696222	HLA-DQB1 - LOC10272501	rs6457620	intron_variant	4.00E-186	Perfect_to_near
10	123337334	16	A	G	123337335	2551	Breast cancer	10q26.13	121577821	FGFR2	rs2981579	intron_variant	2.00E-170	Near_to_perfect
1	114377568	0	A	G	114377568	4871	Rheumatoid arthritis	1p13.2	113834946	PTPN22, AP4B1-AS1	rs2476601	nissense_varia	9.00E-170	New
2	234668563	0	C	T	234668570	1773	Blood metabolite levels	2q37.1	233759924	A8, UGT1A10, UGT1A9, UC	rs887829	intron_variant	3.00E-168	New
11	61579763	14	T	C	61579760	1400	urated fatty acid levels (dil	11q12.2	61812288	FADS1	rs174555	intron_variant	5.00E-168	Near_to_perfect
2	234668563	0	C	T	234668570	1773	Blood metabolite levels	2q37.1	233759924	A8, UGT1A10, UGT1A9, UC	rs887829	intron_variant	2.00E-152	New
2	234668563	0	C	T	234668570	1773	Blood metabolite levels	2q37.1	233759924	A8, UGT1A10, UGT1A9, UC	rs887829	intron_variant	2.00E-140	New
14	64235558	8	G	T	64235556	1119	Blood metabolite ratios	14q23.2	63768838	SGPP1 - SYNE2	rs7157785	itergenic_varia	1.00E-133	Perfect_to_near
4	155482744	16	G	A	155482743	806	Fibrinogen	4q31.3	154561591	PLRG1 - FGB	rs1800789	ream_gene_va	2.00E-127	Near_to_perfect
2	234668563	0	C	T	234668570	1773	Blood metabolite levels	2q37.1	233759924	A8, UGT1A10, UGT1A9, UC	rs887829	intron_variant	3.00E-114	New
1	114377568	0	A	G	114377568	4871	pe 1 diabetes autoantibod	1p13.2	113834946	PTPN22, AP4B1-AS1	rs2476601	nissense_varia	2.00E-111	New
22	24999103	0	C	G	24999104	1539	e levels (gamma-glutamyl	22q11.23	24603137	GGT1	rs2073398	intron_variant	1.00E-109	New
6	32577380	18	A	G	32577380	994	Rheumatoid arthritis	6p21.32	32609603	HLA-DRB1 - HLA-DQA1	rs660895	intron_variant	1.00E-108	Perfect_to_near
2	234668563	0	C	T	234668570	1773	Blood metabolite levels	2q37.1	233759924	A8, UGT1A10, UGT1A9, UC	rs887829	intron_variant	4.00E-95	New
10	114758351	14	C	T	114758349	1141	Type 2 diabetes	10q25.2	112998590	TCF7L2	rs7903146	intron_variant	4.00E-94	Near_to_perfect
16	57005473	16	C	A	57005479	1568	HDL cholesterol	16q13	56971567	CETP	rs1532624	intron_variant	9.00E-94	Non_identical
13	44471878	16	A	G	44471877	1576	Leprosy	13q14.11	43897741	LACC1 - DGKZP1	rs9567307	intron_variant	1.00E-93	Perfect_to_near
16	56995229	30	C	A	56995236	2737	pid metabolism phenotype	16q13	56961324	HERPUD1 - CETP	rs1800775	ream_gene_va	3.00E-93	New
16	56995229	30	C	A	56995236	2737	HDL cholesterol	16q13	56961324	HERPUD1 - CETP	rs1800775	ream_gene_va	4.00E-93	New
16	56995229	30	C	A	56995236	2737	pid metabolism phenotype	16q13	56961324	HERPUD1 - CETP	rs1800775	ream_gene_va	5.00E-87	New
14	64235558	8	G	T	64235556	1119	Blood metabolite levels	14q23.2	63768838	SGPP1 - SYNE2	rs7157785	itergenic_varia	7.00E-87	Perfect_to_near
3	122003759	16	G	T	122003757	472	Calcium levels	3q21.1	122284910	CASR	rs1801725	nissense_varia	9.00E-86	Perfect_to_near
1	114377568	0	A	G	114377568	4871	Type 1 diabetes	1p13.2	113834946	PTPN22, AP4B1-AS1	rs2476601	nissense_varia	9.00E-85	New
16	56995229	30	C	A	56995236	2737	pid metabolism phenotype	16q13	56961324	HERPUD1 - CETP	rs1800775	ream_gene_va	1.00E-81	New
1	114377568	0	A	G	114377568	4871	Type 1 diabetes	1p13.2	113834946	PTPN22, AP4B1-AS1	rs2476601	nissense_varia	2.00E-80	New
16	56995229	30	C	A	56995236	2737	pid metabolism phenotype	16q13	56961324	HERPUD1 - CETP	rs1800775	ream_gene_va	8.00E-78	New
10	114758351	14	C	T	114758349	1141	Type 2 diabetes	10q25.2	112998590	TCF7L2	rs7903146	intron_variant	8.00E-75	Near_to_perfect
10	114758351	14	C	T	114758349	1141	Type 2 diabetes	10q25.2	112998590	TCF7L2	rs7903146	intron_variant	9.00E-75	Near_to_perfect
2	234668563	0	C	T	234668570	1773	Metabolic traits	2q37.1	233759924	A8, UGT1A10, UGT1A9, UC	rs887829	intron_variant	3.00E-74	New
1	114377568	0	A	G	114377568	4871	Rheumatoid arthritis	1p13.2	113834946	PTPN22, AP4B1-AS1	rs2476601	nissense_varia	9.00E-74	New
16	56995229	30	C	A	56995236	2737	HDL cholesterol	16q13	56961324	HERPUD1 - CETP	rs1800775	ream_gene_va	1.00E-73	New
6	31312327	22	T	G	31312326	349	athic membranous nephro	6p21.33	31344549	LOC105375015 - HLA-B	rs3134792	itergenic_varia	6.00E-72	Perfect_to_near
7	2795953	16	A	G	2795957	1052	Height	7p22.3	2756323	GNA12, AMZ1	rs798497	intron_variant	2.00E-71	Near_to_perfect
2	234173494	0	G	A	234173503	1966	Crohn's disease	2q37.1	233264857	ATG16L1	rs12994997	intron_variant	4.00E-70	New
2	234668563	0	C	T	234668570	1773	Bilirubin levels	2q37.1	233759924	A8, UGT1A10, UGT1A9, UC	rs887829	intron_variant	1.00E-69	New
13	42951446	8	C	T	42951449	1421	Bone mineral density	13q14.11	42377313	AKAP11 - LOC105370177	rs9533090	itergenic_varia	5.00E-68	Near_to_perfect
16	57005473	16	C	A	57005479	1568	pid metabolism phenotype	16q13	56971567	CETP	rs1532624	intron_variant	1.00E-66	Non_identical
14	64235558	8	G	T	64235556	1119	Sphingolipid levels	14q23.2	63768838	SGPP1 - SYNE2	rs7157785	itergenic_varia	9.00E-66	Perfect_to_near
14	64235558	8	G	T	64235556	1119	Glycerophospholipid levels	14q23.2	63768838	SGPP1 - SYNE2	rs7157785	itergenic_varia	4.00E-63	Perfect_to_near
19	45392254	24	C	T	45392254	553	Alzheimer's disease neuro	19q13.32	44888997	PVRL2	rs6857	rime_UTR_var	2.00E-62	Near_to_perfect
16	56995229	30	C	A	56995236	2737	pid metabolism phenotype	16q13	56961324	HERPUD1 - CETP	rs1800775	ream_gene_va	2.00E-59	New
9	136154866	18	G	T	136154867	803	atological and biochemical	9q34.2	133279294	ABO - SURF6	rs495828	ream_gene_va	4.00E-59	Near_to_perfect
6	32680921	22	T	G	32680928	443	Crohn's disease	6p21.32	32713151	HLA-DQB1 - LOC10272501	rs7765379	itergenic_varia	9.00E-59	Non_identical
14	64232391	18	G	A	64232386	769	Sphingolipid levels	14q23.2	63765668	SGPP1 - SYNE2	rs17101394	stream_gene_v	3.00E-57	Non_identical
14	67975821	18	A	C	67975822	1898	Glycerophospholipid levels	14q24.1	67509105	TMEM229B	rs1077989	intron_variant	9.00E-57	Perfect_to_near
2	234668563	0	C	T	234668570	1773	Blood metabolite levels	2q37.1	233759924	A8, UGT1A10, UGT1A9, UC	rs887829	intron_variant	2.00E-56	New
6	31632139	20	C	A	31632134	668	athic membranous nephro	6p21.33	31664357	GPANK1	rs3130618	nissense_varia	2.00E-56	Non_identical
16	57005473	16	C	A	57005479	1568	pid metabolism phenotype	16q13	56971567	CETP	rs1532624	intron_variant	3.00E-55	Non_identical
10	63721175	20	C	T	63721176	3225	hoblastic leukemia (B-cell	10q21.2	61961417	ARID5B	rs7090445	intron_variant	5.00E-54	Perfect_to_near
2	632351	16	A	G	632348	4396	Body mass index	2p25.3	632348	C105373352 - LOC105373	rs13021737	itergenic_varia	5.00E-54	Non_identical
16	56995229	30	C	A	56995236	2737	pid metabolism phenotype	16q13	56961324	HERPUD1 - CETP	rs1800775	ream_gene_va	1.00E-53	New
10	114758351	14	C	T	114758349	1141	Type 2 diabetes	10q25.2	112998590	TCF7L2	rs7903146	intron_variant	2.00E-51	Near_to_perfect
2	632351	16	A	G	632348	4396	Body mass index	2p25.3	632348	C105373352 - LOC105373	rs13021737	itergenic_varia	1.00E-50	Non_identical
12	88890672	16	A	G	88890671	3222	Testicular germ cell tumor	12q21.32	88496894	KITLG	rs995030	rime_UTR_var	2.00E-50	Near_to_perfect
1	21766455	18	A	C	21766453	1546	zyme levels (alkaline phos	1p36.12	21439960	NBPF3	rs1976403	ream_gene_va	2.00E-50	Perfect_to_near
7	99266314	14	G	A	99266318	2339	Blood metabolite levels	7q22.1	99668695	CYP3A5, ZSCAN25	rs4646450	intron_variant	5.00E-50	Near_to_perfect
4	187174684	18	A	G	187174683	2750	Blood metabolite levels	4q35.2	186253529	KLKB1	rs4253311	intron_variant	4.00E-48	Perfect_to_near
19	45392254	24	C	T	45392254	553	Neuritic plaque	19q13.32	44888997	PVRL2	rs6857	rime_UTR_var	3.00E-47	Near_to_perfect
9	136142214	20	T	G	136142217	1985	Protein biomarker				rs644234		4.00E-47	Absent
19	45392254	24	C	T	45392254	553	Neurofibrillary tangles	19q13.32	44888997	PVRL2	rs6857	rime_UTR_var	5.00E-47	Near_to_perfect
12	122395774	8	A	G	122395777	4227	Urinary metabolites	12q24.31	121957871	WDR66	rs830124	intron_variant	1.00E-46	Perfect_to_near
12	21368718	24	T	C	21368722	1076	Blood metabolite levels	12p12.1	21215788	SLCO1B1	rs4363657	intron_variant	9.00E-46	Near_to_perfect
14	67975821	18	A	C	67975822	1898	Glycerophospholipid levels	14q24.1	67509105	TMEM229B	rs1077989	intron_variant	1.00E-45	Perfect_to_near
10	114758351	14	C	T	114758349	1141	Type 2 diabetes	10q25.2	112998590	TCF7L2	rs7903146	intron_variant	5.00E-44	Near_to_perfect
19	45392254	24	C	T	45392254	553	Neurofibrillary tangles	19q13.32	44888997	PVRL2	rs6857	rime_UTR_var	5.00E-44	Near_to_perfect
2	233053960	0	A	G	233053961	911	Height	2q37.1	232189251	DIS3L2	rs6728302	intron_variant	8.00E-44	New
4	951946	22	T	C	951947	701	Parkinson's disease	4p16.3	958159	TMEM175	rs34311866	nissense_varia	1.00E-43	Near_to_perfect
16	89708086	24	G	A	89708096	1001	Homocysteine levels	16q24.3	89641688	DPEP1 - CHMP1A	rs154657	stream_gene_v	2.00E-43	Non_identical
20	44545044	22	C	T	44545048	2734	Metabolite levels	20q13.12	45916409	PLTP - PCIF1	rs4810479	ream_gene_va	2.00E-42	Perfect_to_near
2	234639305	0	G	A	234639310	2011	Bilirubin levels	2q37.1	233730664	1A7, UGT1A5, UGT1A6, UC	rs11891311	intron_variant	1.00E-41	New
2	234184413	0	G	A	234184417	1868	Crohn's disease	2q37.1	233275771	SCARNA5, ATG16L1	rs3792109	g_transcript_ex	7.00E-41	New
10	114758351	14	C	T	114758349	1141	Type 2 diabetes	10q25.2	112998590	TCF7L2	rs7903146	intron_variant	2.00E-40	Near_to_perfect
14	67975821	18	A	C	67975822	1898	Glycerophospholipid levels	14q24.1	67509105	TMEM229B	rs1077989	intron_variant	6.00E-40	Perfect_to_near
2	632351	16	A	G	632348	4396	Body mass index	2p25.3	632348	C105373352 - LOC105373	rs13021737	itergenic_varia	7.00E-40	Non_identical
16	57005473	16	C	A	57005479	1568	pid metabolism phenotype	16q13	56971567	CETP	rs1532624	intron_variant	1.00E-39	Non_identical
5	78316475	14	C	T	78316476	1532	od and toenail selenium le	5q14.1	79020653	DMGDH	rs921943	intron_variant	2.00E-39	Near_to_perfect
22	18915346	16	C	T	18915347	628	Amino acid levels	22q11.21	18927834	PRODH	rs2238732	intron_variant	3.00E-39	Perfect_to_near

10	114758351	14	C	T	114758349	1141	Type 2 diabetes	10q25.2	112998590	TCF7L2	rs7903146	intron_variant	2.00E-38	Near_to_perfect
19	45392254	24	C	T	45392254	553	Alzheimer's disease neuro	19q13.32	44888997	PVRL2	rs6857	rime_UTR_var	3.00E-38	Near_to_perfect
7	38128333	20	T	C	38128326	3221	Bone mineral density	7p14.1	38088724	LOC105375236 - STARD3N	rs6959212	ntergenic_varia	4.00E-38	Non_identical
5	150277902	18	A	G	150277909	798	nflammatory bowel diseas	5q33.1	150898347	ZNF300, IRGM	rs11741861	intron_variant	3.00E-37	Non_identical
14	106172881	20	G	A	106172880	2104	ulin G index levels in multi	14q32.33	105706543	LOC105378185 - IGHA1	rs11621145	ream_gene_va	4.00E-37	Near_to_perfect
3	122003759	16	G	T	122003757	472	Calcium levels	3q21.1	122284910	CASR	rs1801725	nissense_varia	6.00E-37	Perfect_to_near
19	49228268	24	T	C	49228272	1082	y metabolites (H-NMR fea	19q13.33	48725015	RASIP1	rs2287921	g_transcript_ex	3.00E-36	Perfect_to_near
6	10969141	10	G	T	10969141	3432	hospholipid levels (plasma	6p24.2	10968908	SYCP2L	rs4713103	intron_variant	3.00E-36	Perfect_to_near
10	114758351	14	C	T	114758349	1141	Type 2 diabetes	10q25.2	112998590	TCF7L2	rs7903146	intron_variant	1.00E-35	Near_to_perfect
11	61579763	14	T	C	61579760	1400	Glycerophospholipid levels	11q12.2	61812288	FADS1	rs174555	intron_variant	1.00E-35	Near_to_perfect
10	114758351	14	C	T	114758349	1141	Type 2 diabetes	10q25.2	112998590	TCF7L2	rs7903146	intron_variant	3.00E-35	Near_to_perfect
6	31391399	14	T	C	31391401	388	HIV-1 control	6p21.33	31423624	MICA - LOC105375017	rs4418214	intron_variant	1.00E-34	Perfect_to_near
10	114758351	14	C	T	114758349	1141	Type 2 diabetes	10q25.2	112998590	TCF7L2	rs7903146	intron_variant	2.00E-34	Near_to_perfect
20	33545618	18	A	G	33545616	406	factors and hematological	20q11.22	35176751	47B, EDEM2 - PROCR, PR6	6088735, rs606	nissense_varia	4.00E-34	Perfect_to_near
11	102695104	20	G	A	102695108	2310	atrix metalloproteinase leve	11q22.2	102824377	WTAPP1	rs495366	intron_variant	6.00E-34	Perfect_to_near
9	136154866	18	G	T	136154867	803	Blood metabolite ratios	9q34.2	133279294	ABO - SURF6	rs495828	ream_gene_va	6.00E-34	Near_to_perfect
6	31263750	8	T	C	31263751	1136	Nasopharyngeal carcinoma	6p21.33	31295974	RPL3P2 - LOC105375015	rs2894207	intron_variant	3.00E-33	Perfect_to_near
1	114377568	0	A	G	114377568	4871	Rheumatoid arthritis	1p13.2	113834946	PTPN22, AP4B1-AS1	rs2476601	nissense_varia	5.00E-33	New
12	28600248	14	C	T	28600244	611	Height	12p11.22	28447311	CCDC91	rs11049611	intron_variant	3.00E-32	Bigger
12	88890672	16	A	G	88890671	3222	Testicular germ cell tumor	12q21.32	88496894	KITLG	rs995030	rime_UTR_var	1.00E-31	Near_to_perfect
10	114758351	14	C	T	114758349	1141	Type 2 diabetes	10q25.2	112998590	TCF7L2	rs7903146	intron_variant	2.00E-31	Near_to_perfect
10	123337334	16	A	G	123337335	2551	Breast cancer	10q26.13	121577821	FGFR2	rs2981579	intron_variant	4.00E-31	Near_to_perfect
2	234668563	0	C	T	234668570	1773	bilirubin levels in HIV-1 inf	2q37.1	233759924	A8, UGT1A10, UGT1A9, UG	rs887829	intron_variant	7.00E-31	New
6	50845488	22	A	G	50845490	873	Body mass index	6p12.3	50877777	RPS17P5 - FTH1P5	rs2207139	ntergenic_varia	8.00E-31	Near_to_perfect
10	114758351	14	C	T	114758349	1141	be 2 diabetes and other tra	10q25.2	112998590	TCF7L2	rs7903146	intron_variant	1.00E-30	Near_to_perfect
8	135650483	12	G	A	135650483	1806	Height	8q24.22	134638240	ZFAT	rs1036821	intron_variant	1.00E-30	Near_to_perfect
4	155482744	16	G	A	155482743	806	Fibrinogen	4q31.3	154561591	PLRG1 - FGB	rs1800789	ream_gene_va	2.00E-30	Near_to_perfect
12	88890672	16	A	G	88890671	3222	Testicular germ cell tumor	12q21.32	88496894	KITLG	rs995030	rime_UTR_var	3.00E-30	Near_to_perfect
10	114758351	14	C	T	114758349	1141	Type 2 diabetes	10q25.2	112998590	TCF7L2	rs7903146	intron_variant	9.00E-30	Near_to_perfect
6	32665429	22	T	G	32665420	595	Follicular lymphoma	6p21.32	32697643	HLA-DQB1 - LOC10272501	rs10484561	intron_variant	1.00E-29	Near_to_perfect
6	30017066	14	T	C	30017071	838	pid metabolism phenotype	6p22.1	30049294	ZNRD1-AS1	rs6917603	intron_variant	3.00E-29	Perfect_to_near
5	131741693	8	A	G	131741696	3572	Blood metabolite levels	5q31.1	132406004	SLC22A5 - C5orf56	rs2405522	ream_gene_va	4.00E-29	Perfect_to_near
6	50845488	22	A	G	50845490	873	Body mass index	6p12.3	50877777	RPS17P5 - FTH1P5	rs2207139	ntergenic_varia	4.00E-29	Perfect_to_near
20	36844035	8	C	T	36844038	1950	Heart rate	20q11.23	38215636	KIAA1755	rs6127471	intron_variant	5.00E-29	Near_to_perfect
12	40792301	22	G	A	40792300	298	nflammatory bowel diseas	12q12	40398498	MUC19, LOC105369736	rs11564258	intron_variant	6.00E-29	Perfect_to_near
16	11693531	22	G	A	11693536	2207	QT interval	16p13.13	11599680	LITAF	rs735951	intron_variant	2.00E-28	Perfect_to_near
4	82150007	22	T	G	82150006	1861	Height	4q21.21	81228852	PRKG2 - RASGEF1B	rs788867	ntergenic_varia	9.00E-28	Perfect_to_near
5	78316475	14	C	T	78316476	1532	ood trace element (Se leve	5q14.1	79020653	DMGDH	rs921943	intron_variant	9.00E-28	Near_to_perfect
19	45392254	24	C	T	45392254	553	Neuritic plaque	19q13.32	44888997	PVRL2	rs6857	rime_UTR_var	2.00E-27	Near_to_perfect
2	102965392	10	A	C	102965392	648	Serum protein levels (sST2	2q12.1	102348932	IL1RL1	rs12999542	intron_variant	2.00E-27	Perfect_to_near
8	6821624	0	T	C	6821617	2572	IgA nephropathy	8p23.1	6964095	DEFA9P - DEFA10P	rs2738058	ream_gene_va	2.00E-27	New
6	32409534	22	G	A	32409530	2545	Systemic sclerosis	6p21.32	32441753	HLA-DRA	rs3129882	intron_variant	2.00E-27	Non_identical
4	146794619	8	T	C	146794621	1362	e levels (gamma-glutamyl	4q31.21	145873469	ZNF827	rs4547811	intron_variant	3.00E-27	Perfect_to_near
9	136166344	24	C	T	136166346	1108	m alkaline phosphatase le	9q34.2	133290774	ABO - SURF6	rs7025162	ntergenic_varia	3.00E-27	Perfect_to_near
14	92482946	14	C	T	92482948	1206	Height	14q32.12	92016604	TRIP11	rs7158300	intron_variant	4.00E-27	Near_to_perfect
6	20679710	20	A	G	20679709	2067	Type 2 diabetes	6p22.3	20679478	CDKAL1	rs7756992	intron_variant	2.00E-26	Perfect_to_near
8	128539361	16	T	G	128539360	4478	Prostate cancer	8q24.21	127527115	CASC8 - LOC105375754	rs7837688	ntergenic_varia	1.00E-25	Perfect_to_near
19	49228268	24	T	C	49228272	1082	Retinal vascular caliber	19q13.33	48725015	RASIP1	rs2287921	g_transcript_ex	2.00E-25	Perfect_to_near
1	152440174	8	A	T	152440176	3682	Atopic dermatitis	1q21.3	152467700	CRNN - LCE5A	rs11205006	ntergenic_varia	2.00E-25	Perfect_to_near
13	42951446	8	C	T	42951449	1421	one mineral density (spine	13q14.11	42377313	AKAP11 - LOC105370177	rs9533090	ntergenic_varia	5.00E-25	Near_to_perfect
2	234668563	0	C	T	234668570	1773	sis-related traits in sickle c	2q37.1	233759924	A8, UGT1A10, UGT1A9, UG	rs887829	intron_variant	5.00E-25	New
8	57200363	18	A	G	57200362	681	Height	8q12.1	56287803	CHCHD7 - SDR16C5	rs7815909	ntergenic_varia	5.00E-25	Perfect_to_near
6	139839431	28	C	A	139839423	1932	Mean corpuscular volume	6q24.1	139518286	OC645434 - LOC10537802	rs643381	atory_region_v	5.00E-25	Smaller
2	234668563	0	C	T	234668570	1773	Metabolite levels	2q37.1	233759924	A8, UGT1A10, UGT1A9, UG	rs887829	intron_variant	9.00E-25	New
16	57005473	16	C	A	57005479	1568	HDL cholesterol	16q13	56971567	CETP	rs1532624	intron_variant	1.00E-24	Non_identical
9	136131459	28	G	A	136131461	766	erum carcinoembryonic an	9q34.2	133256074	ABO	rs8176741	nonymous_vari	2.00E-24	Non_identical
2	234674476	0	A	G	234674476	1624	bilirubin levels in HIV-1 inf	2q37.1	233765830	UGT1A9, UGT1A1, UGT1A8	rs929596	intron_variant	3.00E-24	New
12	33576986	22	A	C	33576990	4144	Heart rate	12p11.1	33424055	SYT10	rs7980799	intron_variant	6.00E-24	Near_to_perfect
1	156255447	20	G	A	156255456	1016	Glycated hemoglobin level	1q22	156285665	TMEM79	rs6684514	nissense_varia	1.00E-23	Near_to_perfect
11	75276179	8	A	C	75276178	3484	Height	11q13.5	75565133	SERPINH1	rs606452	intron_variant	2.00E-23	Near_to_perfect
12	111386125	8	T	C	111386127	156	Gout	12q24.11	110948323	LINC01405 - LOC10536998	rs2188380	intron_variant	2.00E-23	Perfect_to_near
10	114758351	14	C	T	114758349	1141	Type 2 diabetes	10q25.2	112998590	TCF7L2	rs7903146	intron_variant	3.00E-23	Near_to_perfect
2	232982263	0	A	T	232982257	102	Height	2q37.1	232117547	DIS3L2	rs11677466	intron_variant	3.00E-23	New
19	10397401	16	C	T	10397403	1602	le levels of adhesion mole	19p13.2	10286727	ICAM1 - ICAM4	rs3093030	g_transcript_ex	4.00E-23	Perfect_to_near
1	149906408	16	T	C	149906413	1145	Height	1q21.2	149934520	MTMR11	rs11205303	nissense_varia	4.00E-23	Perfect_to_near
22	50971257	28	T	C	50971266	3100	Red blood cell traits	22q13.33	50532837	ODF3B - LOC102724608	rs140522	ream_gene_va	5.00E-23	Near_to_perfect
6	32680921	22	T	G	32680928	443	Rheumatoid arthritis	6p21.32	32713151	HLA-DQB1 - LOC10272501	rs7765379	intron_variant	5.00E-23	Non_identical
2	234668563	0	C	T	234668570	1773	Serum metabolite levels	2q37.1	233759924	A8, UGT1A10, UGT1A9, UG	rs887829	intron_variant	8.00E-23	New
2	234611093	0	T	G	234611094	1318	bilirubin levels in HIV-1 inf	2q37.1	233702448	UGT1A8, UGT1A7, UGT1A9	rs17863787	intron_variant	1.00E-22	New
12	96027757	8	A	G	96027759	1324	Breast cancer	12q22	95633983	USP44 - PGAM1P5	rs17356907	intron_variant	2.00E-22	Perfect_to_near
2	234668563	0	C	T	234668570	1773	Bilirubin levels	2q37.1	233759924	A8, UGT1A10, UGT1A9, UG	rs887829	intron_variant	2.00E-22	New
4	81157698	20	T	C	81157703	1268	Systolic blood pressure	4q21.21	80236549	LOC105377304	rs1902859	atory_region_v	2.00E-22	Near_to_perfect
6	32736690	16	A	T	32736695	1071	omplement C3 and C4 leve	6p21.32	32768918	HLA-DQB2 - HLA-DOB	rs9276606	ntergenic_varia	3.00E-22	Perfect_to_near
X	68798698	18	A	G	68798703	383	development (time to first	Xq13.1	69578860	FAM155B - EDA	rs11796357	ntergenic_varia	3.00E-22	Near_to_perfect
17	68190824	22	G	A	68190826	2568	development (time to first	17q24.3	70194685	KCNJ2 - CALM2P1	rs8079702	ntergenic_varia	4.00E-22	Perfect_to_near
2	632351	16	A	G	632348	4396	Body mass index	2p25.3	632348	C105373352 - LOC105373	rs13021737	ntergenic_varia	4.00E-22	Non_identical
10	63779871	18	C	T	63779871	744	Rheumatoid arthritis	10q21.2	62020112	ARID5B	rs71508903	intron_variant	5.00E-22	Perfect_to_near
8	6821624	0	T	C	6821617	2572	IgA nephropathy	8p23.1	7041476	DEFA9P - DEFA10P, DEFA	rs2738058, rs	ream_gene_va	5.00E-22	New
15	45641216	22	C	A	45641225	3595	Chronic kidney disease	15q21.1	45349027	LOC100533853 - GATM	rs2453533	ntergenic_varia	5.00E-22	Non_identical
10	114758351	14	C	T	114758349	1141	Type 2 diabetes	10q25.2	112998590	TCF7L2	rs7903146	intron_variant	6.00E-22	Near_to_perfect

17	40527543	28	A	G	40527544	2051	nflammatory bowel diseas	17q21.2	42375526	STAT3	rs12942547	intron_variant	6.00E-22	Perfect_to_near
20	32333183	18	G	T	32333181	1148	Height	20q11.22	33745375	ZNF341	rs7274811	intron_variant	6.00E-22	Perfect_to_near
16	88298117	18	C	T	88298124	2705	Central corneal thickness	16q24.2	88264518	ZNF469	rs12447690	intron_variant	6.00E-22	Non_identical
1	20227722	22	G	C,T	20227723	1874,30	Ulcerative colitis	1p36.13	19901230	OTUD3	rs4654925	intron_variant	9.00E-22	Near_to_perfect
8	90662938	18	T	C	90662941	3018	Leprosy	8q21.3	89650713	C105375632 - LOC101929	rs160451	intron_variant	1.00E-21	Near_to_perfect
12	66224459	8	A	C	66224461	144	Polycystic ovary syndrome	12q14.3	65830681	HMGA2	rs2272046	intron_variant	2.00E-21	Near_to_perfect
1	114377568	0	A	G	114377568	4871	Rheumatoid arthritis	1p13.2	113834946	PTPN22, AP4B1-AS1	rs2476601	nissense_variar	2.00E-21	New
20	12969398	16	A	G	12969400	3547	Glycerophospholipid levels	20p12.1	12988752	LOC101929486 - SPTLC3	rs680379	itergenic_variar	2.00E-21	Near_to_perfect
17	38121991	10	G	A	38121993	2158	Asthma (childhood onset)	17q21.1	39965740	GSDMA	rs3894194	nissense_variar	3.00E-21	Perfect_to_near
19	45392254	24	C	T	45392254	553	cerebral amyloid angiopath	19q13.32	44888997	PVRL2	rs6857	rime_UTR_var	3.00E-21	Near_to_perfect
1	67627826	12	A	G	67627828	204	gt-Koyanagi-Harada syndro	1p31.3	67162145	IL23R	rs117633859	intron_variant	3.00E-21	Near_to_perfect
2	241569693	22	C	T	241569692	755	nflammatory bowel diseas	2q37.3	240630275	GPR35	rs3749171	nissense_variar	3.00E-21	Near_to_perfect
10	65133160	16	A	C	65133156	1021	Mean platelet volume	10q21.3	63373396	JMJD1C	rs2393967	intron_variant	3.00E-21	Non_identical
3	22592324	16	G	A	22592321	1495	Motion sickness	3p24.3	22550830	ZNF385D - RANP7	rs11129078	itergenic_variar	3.00E-21	Non_identical
10	114758351	14	C	T	114758349	1141	Type 2 diabetes	10q25.2	112998590	TCF7L2	rs7903146	intron_variant	4.00E-21	Near_to_perfect
1	159685132	0	A	G	159685136	760	C-reactive protein	1q23.2	159715346	CRP - RPL27P2	rs3093059	ream_gene_va	4.00E-21	New
12	40792301	22	G	A	40792300	298	Crohn's disease	12q12	40398498	MUC19, LOC105369736	rs11564258	intron_variant	6.00E-21	Perfect_to_near
16	52575907	10	A	G	52575907	1770	Breast cancer (early onset	16q12.1	52541995	TOX3	rs4784223	intron_variant	6.00E-21	Perfect_to_near
10	114758351	14	C	T	114758349	1141	Proinsulin levels	10q25.2	112998590	TCF7L2	rs7903146	intron_variant	2.00E-20	Near_to_perfect
5	176517329	0	T	C	176517326	4272	Height	5q35.2	177090325	FGFR4	rs422421	intron_variant	2.00E-20	New
6	31099579	18	G	A	31099577	464	Behcet's disease	6p21.33	31131800	PSORS1C1	rs4959053	intron_variant	2.00E-20	Perfect_to_near
2	241819793	20	G	A	241819796	3743	Blood metabolite levels	2q37.3	240880379	AGXT - C2orf54	rs4675874	g_transcript_ex	3.00E-20	Perfect_to_near
8	118185729	20	A	G	118185733	1302	ose-related traits (interacti	8q24.11	117173494	SLC30A8, LOC105375716	rs11558471	rime_UTR_var	3.00E-20	Near_to_perfect
16	57005473	16	C	A	57005479	1568	Cholesterol	16q13	56971567	CETP	rs1532624	intron_variant	3.00E-20	Non_identical
19	49209006	14	T	C	49209010	1681	Metabolic traits	19q13.33	48705753	FUT2	rs503279	rime_UTR_var	4.00E-20	Near_to_perfect
4	81157698	20	T	C	81157703	1268	Diastolic blood pressure	4q21.21	80236549	LOC105377304	rs1902859	atory_region_v	4.00E-20	Near_to_perfect
4	89064579	8	A	C	89064581	2481	Serum uric acid levels	4q22.1	88143429	ABCG2	rs3114018	intron_variant	4.00E-20	Perfect_to_near
6	32577380	18	A	G	32577380	994	IgA nephropathy	6p21.32	32609603	HLA-DRB1 - HLA-DQA1	rs660895	intron_variant	4.00E-20	Perfect_to_near
13	32972625	8	A	T	32972626	22	Lung cancer	13q13.1	32398489	BRCA2	rs11571833	stop_gained	5.00E-20	Near_to_perfect
22	37581487	14	C	A	37581485	2254	Graves' disease	22q12.3	37185445	C1QTNF6	rs229527	nissense_variar	5.00E-20	Near_to_perfect
15	100513164	22	C	T	100513158	2785	Height	15q26.3	99972953	LOC102724973, ADAMTS1	rs2573625	rime_UTR_var	6.00E-20	Near_to_perfect
1	114377568	0	A	G	114377568	4871	etes and autoimmune thyr	1p13.2	113834946	PTPN22, AP4B1-AS1	rs2476601	nissense_variar	7.00E-20	New
2	234667579	0	A	C	234667582	2755	bilirubin levels in HIV-1 inf	2q37.1	233758936	A9, UGT1A4, UGT1A7, UG	rs3755319	intron_variant	9.00E-20	New
2	234668563	0	C	T	234668570	1773	Bilirubin levels	2q37.1	233759924	A8, UGT1A10, UGT1A9, UC	rs887829	intron_variant	9.00E-20	New
10	51524970	18	C	T	51524971	2629	Prostate cancer	10q11.22	46070851	MSMB - LOC105378288	rs3123078	atory_region_v	1.00E-19	Perfect_to_near
13	32972625	8	A	T	32972626	22	Lung cancer	13q13.1	32398489	BRCA2	rs11571833	stop_gained	2.00E-19	Near_to_perfect
6	50845488	22	A	G	50845490	873	Body mass index	6p12.3	50877777	RPS17P5 - FTH1P5	rs2207139	itergenic_variar	2.00E-19	Near_to_perfect
19	55819849	14	C	T	55819845	1555	he and menopause (age a	19q13.42	55308477	BRSK1	rs1172822	intron_variant	2.00E-19	Non_identical
10	94462877	14	C	T	94462882	2285	Type 2 diabetes	10q23.33	92703125	HHEX - EXOC6	rs1111875	atory_region_v	3.00E-19	Near_to_perfect
10	114758351	14	C	T	114758349	1141	Type 2 diabetes	10q25.2	112998590	TCF7L2	rs7903146	intron_variant	3.00E-19	Near_to_perfect
14	69034679	8	C	T	69034682	425	Breast cancer	14q24.1	68567965	RAD51B	rs999737	intron_variant	3.00E-19	Perfect_to_near
6	50845488	22	A	G	50845490	873	Obesity	6p12.3	50877777	RPS17P5 - FTH1P5	rs2207139	itergenic_variar	3.00E-19	Near_to_perfect
6	135427161	20	T	C	135427159	832	Red blood cell traits	6q23.3	135106021	HBS1L - LOC105378010	rs9389269	ream_gene_va	3.00E-19	Perfect_to_near
19	49228268	24	T	C	49228272	1082	y metabolites (H-NMR fea	19q13.33	48725015	RASIP1	rs2287921	g_transcript_ex	7.00E-19	Perfect_to_near
X	68798698	18	A	G	68798703	383	ooth development (numbe	Xq13.1	69578860	FAM155B - EDA	rs11796357	itergenic_variar	7.00E-19	Near_to_perfect
2	71560664	18	A	T	71560665	763	Height	2p13.2	71333535	ZNF638	rs3771381	intron_variant	8.00E-19	Near_to_perfect
4	47563444	12	G	A	47563448	1119	Sphingolipid levels	4p12	47561431	ATP10D	rs10938494	intron_variant	8.00E-19	Near_to_perfect
9	126525213	20	A	G	126525212	2073	Polycystic ovary syndrome	9q33.3	123762933	DENND1A	rs2479106	intron_variant	8.00E-19	Perfect_to_near
4	38784722	8	T	A	38784724	1871	cobacter pylori serologic st	4p14	38783103	TLR10 - TLR1	rs10004195	ream_gene_va	1.00E-18	Near_to_perfect
2	37960612	12	A	T	37960613	95	Height	2p22.2	37733470	LOC105374465	rs17511102	intron_variant	2.00E-18	Near_to_perfect
6	32592734	16	A	G	32592737	449	Nasopharyngeal carcinoma	6p21.32	32624960	HLA-DRB1 - HLA-DQA1	rs28421666	intron_variant	2.00E-18	Perfect_to_near
8	118185729	20	A	G	118185733	1302	Proinsulin levels	8q24.11	117173494	SLC30A8, LOC105375716	rs11558471	rime_UTR_var	3.00E-18	Near_to_perfect
12	2344954	14	A	G	2344960	2486	Schizophrenia	12p13.33	2235794	CACNA1C	rs2007044	intron_variant	3.00E-18	Bigger
7	56079093	0	T	C	56079094	3313	Amino acid levels	7p11.2	56011401	PSPH	rs4947534	rime_UTR_var	4.00E-18	New
20	44749239	26	T	C	44749251	3664	Rheumatoid arthritis	20q13.12	46120612	CD40	rs4239702	intron_variant	4.00E-18	Smaller
6	466036	18	C	T	466033	1616	Freckles	6p25.3	466033	LOC105374875	rs1540771	itergenic_variar	4.00E-18	Non_identical
11	2810730	8	C	T	2810731	564	Height	11p15.5	2789501	KCNQ1	rs2237886	intron_variant	5.00E-18	Perfect_to_near
4	73322562	12	T	C	73322565	17	pid metabolism phenotype	4q13.3	72456848	ADAMTS3	rs115136538	intron_variant	5.00E-18	Perfect_to_near
1	11280991	18	T	C	11280994	763	Corneal curvature	1p36.22	11220937	MTOR	rs74225573	intron_variant	6.00E-18	Near_to_perfect
4	116196304	22	A	T	116196306	119	Resistin levels	4q26	115275150	MRPS33P3 - KRT18P21	rs13144478	itergenic_variar	6.00E-18	Near_to_perfect
4	81157698	20	T	C	81157703	1268	Hypertension	4q21.21	80236549	LOC105377304	rs1902859	atory_region_v	8.00E-18	Near_to_perfect
5	172595306	18	C	T	172595308	1474	tile hypertrophic pyloric ste	5q35.1	173168305	BNIP1 - RPL7AP33	rs29784	stream_gene_v	8.00E-18	Near_to_perfect
6	32572249	20	T	C	32572251	564	lar sclerosis Hodgkin lymp	6p21.32	32604474	163 - HLA-DRA; BTNL2 - H	rs9268542, rs	intron_variant	8.00E-18	Near_to_perfect
14	67975821	18	A	C	67975822	1898	hospholipid levels (plasma	14q24.1	67509105	TMEM229B	rs1077989	intron_variant	9.00E-18	Perfect_to_near
17	44019711	14	G	A	44019712	2015	ogressive supranuclear pa	17q21.31	45942346	MAPT	rs242557	intron_variant	9.00E-18	Perfect_to_near
14	79899450	16	C	T	79899454	2817	Obesity	14q31.1	79433111	NRXN3	rs7141420	intron_variant	1.00E-17	Perfect_to_near
2	234668563	0	C	T	234668570	1773	Serum metabolite levels	2q37.1	233759924	A8, UGT1A10, UGT1A9, UC	rs887829	intron_variant	1.00E-17	New
2	204721748	20	T	C	204721752	2669	Graves' disease	2q33.2	203857029	LOC105373844	rs1024161	itergenic_variar	2.00E-17	Perfect_to_near
12	115346420	16	C	T	115346424	3269	PR interval	12q24.21	114908619	C105369999 - LOC102723	rs1896312	itergenic_variar	3.00E-17	Near_to_perfect
15	73652166	22	A	G	73652174	906	Atrial fibrillation	15q24.1	73359833	HCN4	rs7164883	intron_variant	3.00E-17	Non_identical
19	45411944	8	T	C	45411941	754	1-42 levels in Alzheimer's	19q13.32	44908684	APOE	rs429358	nissense_variar	4.00E-17	Perfect_to_near
19	11347490	20	T	C	11347493	1244	HDL cholesterol	19p13.2	11236817	DOCK6	rs737337	nonymous_vari	5.00E-17	Near_to_perfect
5	147842355	14	T	C	147842353	2154	lmonary function (interacti	5q32	148462790	HTR4	rs11168048	intron_variant	5.00E-17	Perfect_to_near
16	50756773	12	C	G,T	50756774	2,259	Crohn's disease	16q12.1	50722863	NOD2	rs5743289	intron_variant	6.00E-17	Perfect_to_near
11	75276179	8	A	C	75276178	3484	Height	11q13.5	75565133	SERPINH1	rs606452	intron_variant	1.00E-16	Near_to_perfect
11	116653295	8	C	T	116653296	4301	cumference - Triglycerides	11q23.3	116782580	ZPR1	rs2075290	intron_variant	1.00E-16	Near_to_perfect
2	231097122	0	T	C	231097129	668	Crohn's disease	2q37.1	230232414	SP140	rs6716753	intron_variant	1.00E-16	New
7	56097264	8	C	G	56097265	3315	ysteine levels (post-methi	7p11.2	56029572	PSPH	rs4948102	intron_variant	1.00E-16	Near_to_perfect
20	44749239	26	T	C	44749251	3664	Rheumatoid arthritis	20q13.12	46120612	CD40	rs4239702	intron_variant	1.00E-16	Smaller
6	32448606	16	C	T	32448599	2476	Rheumatoid arthritis	6p21.32	32480822	HLA-DRA - HLA-DRB5	rs7748270	stream_gene_v	1.00E-16	Non_identical

8	19824495	28	T	C	19824492	1677	olesterol - Triglycerides (H	8p21.3	19966981	LPL	rs13702	rime_UTR_var	1.00E-16	Non_identical
11	4790576	20	A	G	4790575	243	Malaria	11p15.4	4769345	OR51F1	rs12788102	onymous_vari	2.00E-16	Near_to_perfect
13	24432469	10	A	G	24432467	2866	Myopia (pathological)	13q12.12	23858328	MIPEP	rs9318086	intron_variant	2.00E-16	Perfect_to_near
17	69108749	14	G	T	69108753	2880	Prostate cancer	17q24.3	71112612	CASC17	rs1859962	intron_variant	2.00E-16	Near_to_perfect
1	149906408	16	T	C	149906413	1145	Infant length	1q21.2	149934520	MTMR11	rs11205303	nissense_varia	2.00E-16	Perfect_to_near
20	12969398	16	A	G	12969400	3547	Sphingolipid levels	20p12.1	12988752	LOC101929486 - SPTLC3	rs680379	itergenic_varia	2.00E-16	Near_to_perfect
22	37581487	14	C	A	37581485	2254	Vitiligo	22q12.3	37185445	C1QTNF6	rs229527	nissense_varia	2.00E-16	Near_to_perfect
2	60720753	18	G	A	60720757	2405	F-cell distribution	2p16.1	60498316	BCL11A	0172646, rs46	intron_variant	2.00E-16	Perfect_to_near
2	60722039	8	C	T	60722040	1211	F-cell distribution	2p16.1	60498316	BCL11A	0172646, rs46	intron_variant	2.00E-16	Perfect_to_near
6	16288192	20	G	A	16288192	3535	Blood metabolite levels	6p22.3	16287961	GMPR	rs6459467	intron_variant	2.00E-16	Near_to_perfect
6	32663999	18	G	C	32663999	2678	Height	6p21.32	32696222	HLA-DQB1 - LOC10272501	rs6457620	intron_variant	2.00E-16	Perfect_to_near
6	50845488	22	A	G	50845490	873	Body mass index	6p12.3	50877777	RPS17P5 - FTH1P5	rs2207139	itergenic_varia	2.00E-16	Near_to_perfect
6	111696091	22	T	C	111696091	202	Psoriasis	6q21	111374888	REV3L	rs458017	nissense_varia	2.00E-16	Near_to_perfect
17	46974743	24	T	C	46974734	2128	Height	17q21.32	48897372	ATP5G1 - UBE2Z	rs318095	intron_variant	2.00E-16	Non_identical
1	159326883	16	A	G	159326880	424	IgE levels	1q23.2	159357090	OR10J3 - OR10J1	rs4656784	intron_variant	2.00E-16	Non_identical
7	150543722	8	T	G	150543721	840	Blood metabolite ratios	7q36.1	150846633	LOC105375567, AOC1	rs1005390	intron_variant	3.00E-16	Near_to_perfect
9	136154866	18	G	T	136154867	803	Venous thromboembolism	9q34.2	133279294	ABO - SURF6	rs495828	ream_gene_va	3.00E-16	Near_to_perfect
1	158575731	10	C	T	158575729	1318	Red blood cell traits	1q23.1	158605939	OR10X1 - OR10Z1	rs857684	ream_gene_va	4.00E-16	Near_to_perfect
5	37046633	18	A	G	37046626	3137	Height	5p13.2	37046524	NIPBL	rs301901	intron_variant	4.00E-16	Smaller
2	234597313	0	A	T	234597321	1387	oilirubin levels in HIV-1 inf	2q37.1	233688675	A10, UGT1A8, UGT1A7, UC	rs10173355	intron_variant	5.00E-16	New
10	114758351	14	C	T	114758349	1141	Type 2 diabetes	10q25.2	112998590	TCF7L2	rs7903146	intron_variant	6.00E-16	Near_to_perfect
3	195941217	0	C	G	195941216	3481	Blood metabolite levels	3q29	196214345	LOC105374304	rs7642243	nissense_varia	8.00E-16	New
9	136141861	20	C	T	136141870	708	Venous thromboembolism				rs2519093		8.00E-16	Non_identical
15	51530495	12	G	A	51530495	1157	Height	15q21.2	51238298	CYP19A1, PIRC66	rs16964211	intron_variant	1.00E-15	Near_to_perfect
5	172595306	18	C	T	172595308	1474	ile hypertrophic pyloric ste	5q35.1	173168305	BNIP1 - RPL7AP33	rs29784	stream_gene_v	1.00E-15	Near_to_perfect
10	114758351	14	C	T	114758349	1141	Type 2 diabetes	10q25.2	112998590	TCF7L2	rs7903146	intron_variant	2.00E-15	Near_to_perfect
10	114758351	14	C	T	114758349	1141	Type 2 diabetes	10q25.2	112998590	TCF7L2	rs7903146	intron_variant	2.00E-15	Near_to_perfect
6	26091176	16	C	G	26091179	366	Diastolic blood pressure	6p22.2	26090951	HFE	rs1799945	nissense_varia	2.00E-15	Perfect_to_near
2	165540806	26	T	C	165540800	544	HDL cholesterol	2q24.3	164684290	COBLL1	rs12328675	rime_UTR_var	2.00E-15	Non_identical
10	63779871	18	C	T	63779871	744	Rheumatoid arthritis	10q21.2	62020112	ARID5B	rs71508903	intron_variant	3.00E-15	Perfect_to_near
11	5264145	12	T	G	5264146	1033	alassemia/hemoglobin E c	11p15.4	5242916	HBBP1	rs2071348	intron_variant	3.00E-15	Near_to_perfect
12	117323367	14	T	C	117323367	249	ocortical brain region volur	12q24.22	116885562	HRK - FBXW8	rs77956314	ream_gene_va	3.00E-15	Near_to_perfect
15	79235445	14	T	C	79235446	1843	Type 1 diabetes	15q25.1	78943104	CTSH	rs3825932	intron_variant	3.00E-15	Near_to_perfect
20	44746982	0	T	C	44746982	3862	Chronic hepatitis B infectio	20q13.12	46118343	CD40	rs1883832	rime_UTR_var	3.00E-15	New
6	32341351	8	G	A	32341353	2367	Coronary heart disease	6p21.32	32373576	LOC101929163	rs9268402	ream_gene_va	3.00E-15	Perfect_to_near
15	99271136	8	A	G	99271135	2755	Urate levels	15q26.3	98727906	IGF1R	rs6598541	intron_variant	5.00E-15	Perfect_to_near
16	11691751	18	T	C	11691753	2528	QT interval	16p13.13	11597897	LITAF	rs8049607	intron_variant	5.00E-15	Perfect_to_near
8	9183600	16	A	G	9183596	4541	Glycemic traits (pregnancy	8p23.1	9326086	LOC157273	rs4841132	g_transcript_ex	5.00E-15	Non_identical
11	61579763	14	T	C	61579760	1400	Trans fatty acid levels	11q12.2	61812288	FADS1	rs174555	intron_variant	6.00E-15	Near_to_perfect
16	11691751	18	T	C	11691753	2528	QT interval	16p13.13	11597897	LITAF	rs8049607	intron_variant	6.00E-15	Perfect_to_near
12	88953951	18	G	A	88953959	3136	Testicular cancer	12q21.32	88560182	KITLG	rs4474514	intron_variant	6.00E-15	Non_identical
8	120044829	14	G	A	120044829	3748	one mineral density (spine	8q24.12	119032590	COLEC10	rs6469804	intron_variant	7.00E-15	Perfect_to_near
9	117083796	18	C	A,T	117083803	134,2	n generation potential phe	9q32	114321523	LOC105376224 - ORM1	rs150611042	ream_gene_va	7.00E-15	Non_identical
20	12969398	16	A	G	12969400	3547	Sphingolipid levels	20p12.1	12988752	LOC101929486 - SPTLC3	rs680379	itergenic_varia	8.00E-15	Near_to_perfect
7	151406003	16	C	T	151406005	788	Red blood cell traits	7q36.1	151708919	PRKAG2	rs10480300	intron_variant	8.00E-15	Near_to_perfect
14	79899450	16	C	T	79899454	2817	Body mass index	14q31.1	79433111	NRXN3	rs7141420	intron_variant	9.00E-15	Perfect_to_near
3	129050763	20	T	C	129050756	4246	Height	3q21.3	129331913	H1FX-AS1 - RPL32P3	rs6439167	ream_gene_va	9.00E-15	Non_identical
10	114758351	14	C	T	114758349	1141	Type 2 diabetes	10q25.2	112998590	TCF7L2	rs7903146	intron_variant	1.00E-14	Near_to_perfect
14	79899450	16	C	T	79899454	2817	Body mass index	14q31.1	79433111	NRXN3	rs7141420	intron_variant	1.00E-14	Perfect_to_near
17	68190824	22	G	A	68190826	2568	ooth development (numbe	17q24.3	70194685	KCNJ2 - CALM2P1	rs8079702	itergenic_varia	1.00E-14	Perfect_to_near
1	47347423	0	A	T	47347427	1014	Blood metabolite levels	1p33	46881755	CYP4Z2P	rs6663731	intron_variant	1.00E-14	New
20	22050501	8	A	G	22050503	3112	Male-pattern baldness	20p11.22	22069865	LINC01432	rs1160312	intron_variant	1.00E-14	Near_to_perfect
2	60608754	22	T	C	60608759	834	Mean corpuscular volume	2p16.1	60381624	LOC105374756 - MIR4432	rs2540917	intron_variant	1.00E-14	Near_to_perfect
5	56168713	8	C	T	56168712	223	Breast cancer (early onset	5q11.2	56872885	MAP3K1	rs2229882	onymous_vari	1.00E-14	Near_to_perfect
10	114758351	14	C	T	114758349	1141	ose-related traits (interacti	10q25.2	112998590	TCF7L2	rs7903146	intron_variant	2.00E-14	Near_to_perfect
11	116653295	8	C	T	116653296	4301	olesterol - Triglycerides (H	11q23.3	116782580	ZPR1	rs2075290	intron_variant	2.00E-14	Near_to_perfect
15	58723423	12	A	G	58723426	1997	HDL cholesterol	15q21.3	58431227	LIPC, LOC102724766	rs1077835	intron_variant	2.00E-14	Near_to_perfect
5	134499090	0	C	A	134499092	2701	Colorectal cancer	5q31.1	135163402	C5orf66	rs647161	intron_variant	2.00E-14	New
6	11042911	16	G	A	11042909	969	Metabolic traits	6p24.2	11042676	ELOVL2	rs9393903	intron_variant	2.00E-14	Near_to_perfect
7	56079093	0	T	C	56079094	3313	Blood metabolite levels	7p11.2	56011401	PSPH	rs4947534	rime_UTR_var	2.00E-14	New
8	118184780	8	C	T	118184783	1278	Type 2 diabetes	8q24.11	117172544	LOC105375716, SLC30A8	rs13266634	nissense_varia	2.00E-14	Near_to_perfect
11	59939301	24	T	G	59939307	2737	Alzheimer's disease	11q12.2	60171834	MS4A6A	rs610932	stream_gene_v	2.00E-14	Smaller
13	43116125	18	C	T	43116133	3885	Bone mineral density	13q14.11	42541997	LOC105370177 - TNFSF11	rs1021188	itergenic_varia	2.00E-14	Non_identical
1	197781190	18	C	T	197781198	878	Primary biliary cirrhosis	1q31.3	197812068	DENND1B - C1orf53	rs12134279	atory_region_v	2.00E-14	Non_identical
9	28414346	18	A	G	28414339	1020	Body mass index	9p21.1	28414341	LINGO2	rs10968576	intron_variant	2.00E-14	Smaller
19	10577842	24	C	A	10577843	943	Inflammatory skin disease	19p13.2	10467167	PDE4A	rs1051738	nissense_varia	3.00E-14	Perfect_to_near
7	150543722	8	T	G	150543721	840	Blood metabolite levels	7q36.1	150846633	LOC105375567, AOC1	rs1005390	intron_variant	3.00E-14	Near_to_perfect
3	162161613	24	T	C	162161616	1280	Coronary heart disease	3q26.1	162449608	OTOL1 - LINC01192	924705, rs678	itergenic_varia	4.00E-14	Near_to_perfect
13	43116125	18	C	T	43116133	3885	Bone mineral density	13q14.11	42541997	LOC105370177 - TNFSF11	rs1021188	itergenic_varia	4.00E-14	Non_identical
5	141513198	14	A	T	141513204	3337	nflammatory bowel diseas	5q31.3	142133639	NDFIP1	rs6863411	intron_variant	4.00E-14	Non_identical
19	45411944	8	T	C	45411941	754	heimer's disease biomark	19q13.32	44908684	APOE	rs429358	nissense_varia	5.00E-14	Perfect_to_near
2	234158833	0	C	T	234158839	1963	Crohn's disease	2q37.1	233250193	INPP5D - ATG16L1	rs10210302	intron_variant	5.00E-14	New
2	177042638	20	A	C	177042633	4094	Ovarian cancer	2q31.1	176177905	HAGLR	rs2072590	g_transcript_ex	5.00E-14	Non_identical
2	74202577	8	T	C	74202578	1025	stemic lupus erythematos	2p13.1	73975451	DGUOK-AS1	rs4852324	intron_variant	6.00E-14	Near_to_perfect
6	135423210	30	T	C	135423209	1520	ther erythrocyte phenotype	6q23.3	135102071	HBS1L - LOC105378010	rs9373124	intron_variant	7.00E-14	Perfect_to_near
8	27195120	14	T	C	27195121	1581	heimer's disease (late ons	8p21.2	27337604	PTK2B	rs28834970	intron_variant	7.00E-14	Perfect_to_near
9	28414346	18	A	G	28414339	1020	Body mass index	9p21.1	28414341	LINGO2	rs10968576	intron_variant	7.00E-14	Smaller
6	10969141	10	G	T	10969141	3432	hospholipid levels (plasma	6p24.2	10968908	SYCP2L	rs4713103	intron_variant	8.00E-14	Perfect_to_near
17	61763184	16	T	C	61763185	1691	Height	17q23.3	63685825	MAP3K3, LOC101927898	rs3785574	intron_variant	9.00E-14	Near_to_perfect
19	6668977	20	C	T	6668972	941	Multiple sclerosis	19p13.3	6668961	TNFSF14	rs1077667	intron_variant	9.00E-14	Near_to_perfect

2	234827661	0	G	A	234827661	281	Migraine	2q37.1	233919016	TRPM8	rs6741751	intron_variant	9.00E-14	New
2	177042638	20	A	C	177042633	4094	Epithelial ovarian cancer	2q31.1	176177905	HAGLR	rs2072590	g_transcript_ex	9.00E-14	Non_identical
12	14653867	16	C	T	14653867	1337	Testicular germ cell tumor	12p13.1	14500933	ATF7IP	rs2900333	rime_UTR_var	1.00E-13	Near_to_perfect
15	100514612	8	T	C	100514614	3403	Height	15q26.3	99974409	ADAMTS17	rs2573652	nissense_variai	1.00E-13	Near_to_perfect
16	792185	14	A	G	792190	2698	Height	16p13.3	742190	NARFL - MSLN	rs11648796	intron_variant	1.00E-13	Perfect_to_near
19	49209006	14	T	C	49209010	1681	Blood metabolite ratios	19q13.33	48705753	FUT2	rs503279	rime_UTR_var	1.00E-13	Near_to_perfect
1	203155883	0	G	C	203155882	3934	YKL-40 levels	1q32.1	203186754	CHI3L1	rs4950928	ream_gene_va	1.00E-13	New
2	234183359	0	A	G	234183368	1983	Crohn's disease	2q37.1	233274722	ATG16L1	rs2241880	nissense_variai	1.00E-13	New
3	36858580	0	C	T	36858583	1037	Schizophrenia	3p22.2	36817092	C105377030 - LOC105377(	rs75968099	stream_gene_v	1.00E-13	New
7	143110759	18	G	A	143110762	2164	heimer's disease (late ons	7q35	143413669	EPHA1-AS1	rs11771145	intron_variant	1.00E-13	Near_to_perfect
11	2810730	8	C	T	2810731	564	Height	11p15.5	2789501	KCNQ1	rs2237886	intron_variant	2.00E-13	Perfect_to_near
15	60781514	16	T	C	60781513	1275	Menarche (age at onset)	15q22.2	60489314	RORA, RORA-AS1	rs3743266	rime_UTR_var	2.00E-13	Near_to_perfect
1	49589845	20	A	G	49589847	2270	Body mass index	1p33	49124175	LOC105378706, AGBL4	rs657452	intron_variant	2.00E-13	Near_to_perfect
1	157669277	12	C	T	157669278	2315	Graves' disease	1q23.1	157699488	FCRL3	rs3761959	intron_variant	2.00E-13	Perfect_to_near
22	25002076	34	C	T	25002081	2193	Blood metabolite levels	22q11.23	24606114	GGT1	rs2330809	intron_variant	2.00E-13	Near_to_perfect
2	160867057	22	G	A	160867059	4058	Bilirubin levels	2q24.2	160010548	PLA2R1	rs2667011	intron_variant	2.00E-13	Perfect_to_near
7	106411859	12	T	C	106411858	840	Blood pressure	7q22.3	106771412	CCDC71L - PIK3CG	rs17477177	ream_gene_va	2.00E-13	Near_to_perfect
9	109599052	16	G	A	109599046	946	Height	9q31.2	106836765	LINC01505 - LOC10537620	rs7027110	ntergenic_variai	2.00E-13	Near_to_perfect
15	78802876	28	C	T	78802869	3438	histogram emphysema pa	15q25.1	78510527	HYKK	rs9788721	intron_variant	2.00E-13	Smaller
1	25674778	20	C	T	25674785	1677	ythrocyte sedimentation ra	1p36.11	25348294	TMEM50A	rs3091242	intron_variant	2.00E-13	Non_identical
6	32680921	22	T	G	32680928	443	Enteric fever	6p21.32	32713151	HLA-DQB1 - LOC10272501	rs7765379	intron_variant	2.00E-13	Non_identical
12	88890672	16	A	G	88890671	3222	Testicular germ cell tumor	12q21.32	88496894	KITLG	rs995030	rime_UTR_var	3.00E-13	Near_to_perfect
16	56995229	30	C	A	56995236	2737	Triglycerides	16q13	56961324	HERPUD1 - CETP	rs1800775	ream_gene_va	3.00E-13	New
2	231116869	0	C	T	231116874	507	Crohn's disease	2q37.1	230252159	SP140	rs7423615	intron_variant	3.00E-13	New
7	110898918	10	T	C	110898915	2142	Schizophrenia	7q31.1	111258859	IMMP2L	rs13240464	intron_variant	3.00E-13	Smaller
7	127862802	12	A	G	127862802	3837	Type 2 diabetes	7q32.1	128222749	LOC101928423	rs791595	atory_region_v	3.00E-13	Near_to_perfect
13	54102212	20	G	A	54102206	783	Body mass index	13q14.3	53528071	LINC01065 - LOC10537021	rs12429545	ntergenic_variai	3.00E-13	Non_identical
8	9183600	16	A	G	9183596	4541	Glycemic traits (pregnancy	8p23.1	9326086	LOC157273	rs4841132	g_transcript_ex	3.00E-13	Non_identical
9	28414346	18	A	G	28414339	1020	Body mass index	9p21.1	28414341	LINGO2	rs10968576	intron_variant	3.00E-13	Smaller
15	89863930	8	T	G	89863928	2245	Menopause (age at onset)	15q26.1	89320697	POLG	rs2307449	intron_variant	4.00E-13	Perfect_to_near
19	45392254	24	C	T	45392254	553	Verbal declarative memory	19q13.32	44888997	PVRL2	rs6857	rime_UTR_var	4.00E-13	Near_to_perfect
19	45392254	24	C	T	45392254	553	Verbal declarative memory	19q13.32	44888997	PVRL2	rs6857	rime_UTR_var	4.00E-13	Near_to_perfect
2	204721748	20	T	C	204721752	2669	Alopecia areata	2q33.2	203857029	LOC105373844	rs1024161	ntergenic_variai	4.00E-13	Perfect_to_near
7	44231213	20	T	G	44231216	2140	Metabolic syndrome	7p13	44191617	GCK - YKT6	rs3757840	intron_variant	4.00E-13	Perfect_to_near
8	6821624	0	T	C	6821617	2572	IgA nephropathy	8p23.1	7041476	DEFA9P - DEFA10P, DEFA	rs2738058, rs	ream_gene_va	4.00E-13	New
9	117592632	14	T	C	117592638	623	rative colitis or Crohn's dis	9q32	114830358	TNFSF15 - TNFSF8	rs2006996	atory_region_v	4.00E-13	Near_to_perfect
6	32448606	16	C	T	32448599	2476	Rheumatoid arthritis	6p21.32	32480822	HLA-DRA - HLA-DRB5	rs7748270	stream_gene_v	4.00E-13	Non_identical
1	49589845	20	A	G	49589847	2270	Body mass index	1p33	49124175	LOC105378706, AGBL4	rs657452	intron_variant	5.00E-13	Near_to_perfect
1	151907180	22	A	G	151907173	1558	Acylcarnitine levels	1q21.3	151934697	LOC105373470	rs10494270	ntergenic_variai	5.00E-13	Smaller
4	113855479	18	C	G	113855477	1137	al exhaled nitric oxide (ch	4q25	112934321	ANK2	rs12500579	intron_variant	5.00E-13	Perfect_to_near
5	4029786	18	C	T	4029789	910	Myocardial infarction	5p15.33	4029676	C105374626 - LOC105374	rs11748327	stream_gene_v	5.00E-13	Near_to_perfect
14	73081065	18	A	G	73081068	3811	e protein and white blood	14q24.2	72614360	DPF3	rs2526932	stream_gene_v	6.00E-13	Near_to_perfect
20	12969398	16	A	G	12969400	3547	saturated fatty acid levels	20p12.1	12988752	LOC101929486 - SPTLC3	rs680379	ntergenic_variai	6.00E-13	Near_to_perfect
2	234668563	0	C	T	234668570	1773	Serum metabolite levels	2q37.1	233759924	A8, UGT1A10, UGT1A9, UC	rs887829	intron_variant	6.00E-13	New
4	111334730	18	G	T	111334730	2906	Metabolic traits	4q25	110413574	LOC105377362 - ENPEP	rs2087160	intron_variant	7.00E-13	Near_to_perfect
17	6940389	10	G	A	6940393	4550	Type 2 diabetes	17p13.1	7037074	SLC16A13	rs312457	intron_variant	8.00E-13	Near_to_perfect
1	197631143	8	G	A	197631141	1169	nflammatory bowel diseas	1q31.3	197662011	DENND1B	rs2488389	intron_variant	8.00E-13	Near_to_perfect
3	169518456	16	T	C	169518455	2110	Interstitial lung disease	3q26.2	169800667	LRRC34	rs6793295	nissense_variai	8.00E-13	Perfect_to_near
5	95850245	18	A	C	95850250	2058	Body mass index	5q15	96514546	LOC101929710	rs261967	intron_variant	8.00E-13	Perfect_to_near
10	114758351	14	C	T	114758349	1141	Body mass index	10q25.2	112998590	TCF7L2	rs7903146	intron_variant	1.00E-12	Near_to_perfect
11	32364187	18	G	A	32364187	898	Tuberculosis	11p13	32342641	RCN1 - WT1	rs2057178	intron_variant	1.00E-12	Perfect_to_near
19	45411944	8	T	C	45411941	754	Lewy body disease	19q13.32	44908684	APOE	rs429358	nissense_variai	1.00E-12	Perfect_to_near
19	49228268	24	T	C	49228272	1082	y metabolites (H-NMR fea	19q13.33	48725015	RASIP1	rs2287921	g_transcript_ex	1.00E-12	Perfect_to_near
2	234183359	0	A	G	234183368	1983	Crohn's disease	2q37.1	233274722	ATG16L1	rs2241880	nissense_variai	1.00E-12	New
2	234825089	0	T	C	234825093	2706	Migraine	2q37.1	233916448	MSL3P1 - TRPM8	rs10166942	ream_gene_va	1.00E-12	New
2	239694638	16	C	A	239694631	2329	Male-pattern baldness	2q37.3	238785990	C105373962 - LOC100287	rs9287638	stream_gene_v	1.00E-12	Near_to_perfect
5	176517329	0	T	C	176517326	4272	Height	5q35.2	177090325	FGFR4	rs422421	intron_variant	1.00E-12	New
8	90823683	10	A	G	90823687	2542	Leprosy	8q21.3	89811459	RIPK2 - OSGIN2	rs40457	atory_region_v	1.00E-12	Near_to_perfect
13	54102212	20	G	A	54102206	783	Body mass index	13q14.3	53528071	LINC01065 - LOC10537021	rs12429545	ntergenic_variai	1.00E-12	Non_identical
15	78802876	28	C	T	78802869	3438	histogram emphysema pa	15q25.1	78510527	HYKK	rs9788721	intron_variant	1.00E-12	Smaller
2	27152873	8	C	T	27152874	1341	ose-related traits (interacti	2p23.3	26930006	DPYSL5	rs1371614	intron_variant	2.00E-12	Perfect_to_near
2	134434826	18	T	C	134434824	1012	Height	2q21.2	133677253	NCKAP5 - LOC105373626	rs7567288	atory_region_v	2.00E-12	Perfect_to_near
2	177022154	28	C	G	177022158	4104	Motion sickness	2q31.1	176157430	HOXD3	rs2551802	intron_variant	2.00E-12	Near_to_perfect
2	239306263	20	A	C	239306268	214	Iris characteristics	2q37.3	238397627	TRAF3IP1	rs3739070	nissense_variai	2.00E-12	Near_to_perfect
4	1078184	16	G	A	1078187	3873	ecombination rate (female	4p16.3	1084399	RNF212	rs1670533	intron_variant	2.00E-12	Perfect_to_near
5	55001897	14	G	A	55001899	2971	Height	5q11.2	55706071	SLC38A9	rs11958779	intron_variant	2.00E-12	Absent
1	22492878	22	G	C	22492887	1184	mineral density (paediatric	1p36.12	22166394	LOC105376850	rs3920498	ntergenic_variai	2.00E-12	Non_identical
6	32809855	24	G	A	32809848	669	Nephropathy	6p21.32	32842071	PSMB8	rs9357155	intron_variant	2.00E-12	Smaller
15	89042469	16	C	T	89042467	1353	Menarche (age at onset)	15q25.3	88499236	MRPS11 - DET1	rs12915845	intron_variant	3.00E-12	Near_to_perfect
2	237933975	0	A	C	237933966	1755	Motion sickness	2q37.3	237025323	LOC105373951 - LOC9346	rs2318131	atory_region_v	3.00E-12	New
3	52833218	10	G	A	52833219	2426	lisorder, bipolar disorder, r	3p21.1	52799203	ITIH3	rs2535629	intron_variant	3.00E-12	Perfect_to_near
6	133315795	0	T	C	133315804	4098	Bone mineral density	6q23.2	132994665	LOC105378008 - LINC0032	rs271170	stream_gene_v	3.00E-12	New
9	136154866	18	G	T	136154867	803	Red blood cell count	9q34.2	133279294	ABO - SURF6	rs495828	ream_gene_va	3.00E-12	Near_to_perfect
4	187149538	10	G	A	187149540	2905	Metabolite levels	4q35.2	186228386	KLKB1	rs1912826	intron_variant	4.00E-12	Perfect_to_near
6	21430732	0	T	C	21430728	2347	Crohn's disease	6p22.3	21430497	CDKAL1 - LINC00581	rs12663356	ntergenic_variai	4.00E-12	New
19	45411944	8	T	C	45411941	754	Lewy body disease	19q13.32	44908684	APOE	rs429358	nissense_variai	5.00E-12	Perfect_to_near
19	49144784	20	C	T	49144790	1149	erum carcinoembryonic an	19q13.33	48641533	SEC1P, CA11	rs11880333	intron_variant	5.00E-12	Non_identical
15	62396942	12	G	A	62396942	2375	ose-related traits (interacti	15q22.2	62104743	NPM1P47 - C2CD4B	rs7173964	atory_region_v	6.00E-12	Near_to_perfect
17	33324384	18	C	T	33324382	1555	QT interval	17q12	34997363	LIG3	rs2074518	g_transcript_ex	6.00E-12	Perfect_to_near
2	25373303	20	G	A	25373298	2984	Height	2p23.3	25150429	EFR3B	rs13428823	intron_variant	6.00E-12	Non_identical

2	234825089	0	T	C	234825093	2706	Migraine	2q37.1	233916448	MSL3P1 - TRPM8	rs10166942	ream_gene_va	6.00E-12	New
10	94462877	14	C	T	94462882	2285	Type 2 diabetes	10q23.33	92703125	HHEX - EXOC6	rs1111875	atory_region_v	7.00E-12	Near_to_perfect
12	49676009	20	T	C	49676010	2914	Prostate cancer	12q13.12	49282227	TUBA1C - LOC101927267	rs10875943	atory_region_v	7.00E-12	Perfect_to_near
19	49118371	12	G	A	49118371	593	erum carcinoembryonic an	19q13.33	48615114	FAM83E - RPL18	rs2292342	ream_gene_va	7.00E-12	Perfect_to_near
2	234625622	0	C	T	234625622	2087	bilirubin levels in HIV-1 inf	2q37.1	233716976	A8, UGT1A10, UGT1A6, UC	rs1875263	intron_variant	7.00E-12	New
10	96405321	22	C	T	96405329	1690	ocoumarol maintenance do	10q23.33	94645572	HELLS - CYP2C18	rs12772169	stream_gene_v	8.00E-12	Non_identical
10	114758351	14	C	T	114758349	1141	Type 2 diabetes	10q25.2	112998590	TCF7L2	rs7903146	intron_variant	8.00E-12	Near_to_perfect
11	62915348	8	C	G	62915346	89	Sex hormone levels	11q12.3	63147874	SLC22A24 - SLC22A25	rs112295236	intron_variant	8.00E-12	Near_to_perfect
2	177037316	18	A	G	177037311	4099	lucinous ovarian carcinom	2q31.1	176172583	HOXD3	rs711830	rime_UTR_var	8.00E-12	Non_identical
6	26091176	16	C	G	26091179	366	Systolic blood pressure	6p22.2	26090951	HFE	rs1799945	nissense_varia	8.00E-12	Perfect_to_near
15	96708284	20	T	C	96708291	1565	hormone-binding globulin l	15q26.2	96165062	NR2F2-AS1	rs8023580	intron_variant	8.00E-12	Bigger
2	219908366	18	T	G	219908369	967	Height	2q35	219043647	CCDC108 - IHH	rs12470505	ream_gene_va	9.00E-12	Perfect_to_near
10	16919054	18	T	C	16919052	315	Urinary albumin excretion	10p13	16877053	CUBN	rs1801239	nissense_varia	1.00E-11	Near_to_perfect
10	114758351	14	C	T	114758349	1141	Body mass index	10q25.2	112998590	TCF7L2	rs7903146	intron_variant	1.00E-11	Near_to_perfect
14	25928176	20	C	A	25928179	1529	Body mass index	14q12	25458973	HMGN2P6 - LOC10537041(	rs10132280	itergenic_varia	1.00E-11	Perfect_to_near
14	25928176	20	C	A	25928179	1529	Body mass index	14q12	25458973	HMGN2P6 - LOC10537041(	rs10132280	itergenic_varia	1.00E-11	Perfect_to_near
14	79899450	16	C	T	79899454	2817	Body mass index	14q31.1	79433111	NRXN3	rs7141420	intron_variant	1.00E-11	Perfect_to_near
16	56995229	30	C	A	56995236	2737	Blood metabolite levels	16q13	56961324	HERPUD1 - CETP	rs1800775	ream_gene_va	1.00E-11	New
1	200935864	22	T	C	200935866	769	Crohn's disease	1q32.1	200966738	MROH3P - KIF21B	rs11584383	stream_gene_v	1.00E-11	Near_to_perfect
2	220028896	14	A	G	220028900	3074	Height	2q35	219164178	SLC23A3	rs6753739	intron_variant	1.00E-11	Perfect_to_near
3	87134800	8	A	G	87134800	379	Prostate cancer	3p12.1	87085650	LOC285232 - LINC00506	rs17023900	ream_gene_va	1.00E-11	Near_to_perfect
4	145650021	16	G	A	145650021	2635	Height	4q31.21	144728869	HHIP	rs1492820	intron_variant	1.00E-11	Near_to_perfect
4	156511458	18	G	T	156511459	2749	Coronary heart disease	4q32.1	155590307	LOC105377504 - GUCY1A3	rs1842896	itergenic_varia	1.00E-11	Perfect_to_near
5	147842355	14	T	C	147842353	2154	Pulmonary function	5q32	148462790	HTR4	rs11168048	intron_variant	1.00E-11	Perfect_to_near
6	31842592	16	T	A	31842598	473	e age-related macular deg	6p21.33	31874821	SLC44A4	rs12661281	nissense_varia	1.00E-11	Near_to_perfect
9	136154866	18	G	T	136154867	803	atological and biochemical	9q34.2	133279294	ABO - SURF6	rs495828	ream_gene_va	1.00E-11	Near_to_perfect
2	66756967	22	A	G	66756976	3458	PR interval	2p14	66529844	MEIS1	rs3891585	intron_variant	1.00E-11	Bigger
9	28414346	18	A	G	28414339	1020	Body mass index	9p21.1	28414341	LINGO2	rs10968576	intron_variant	1.00E-11	Smaller
11	59997661	18	C	T	59997666	1919	ciated phospholipase A2 a	11q12.2	60230193	MS4A4E	rs600550	intron_variant	2.00E-11	Near_to_perfect
11	60832287	12	T	C	60832282	3652	Multiple sclerosis	11q12.2	61064810	LOC105369325	rs650258	intron_variant	2.00E-11	Bigger
16	75390306	0	T	A	75390316	2532	Pulmonary function	16q23.1	75356418	CFDP1	rs2865531	intron_variant	2.00E-11	New
19	38735608	18	C	T	38735613	2292	Prostate cancer	19q13.2	38244973	DPF1 - PPP1R14A	rs8102476	intron_variant	2.00E-11	Perfect_to_near
1	114377568	0	A	G	114377568	4871	Rheumatoid arthritis	1p13.2	113834946	PTPN22, AP4B1-AS1	rs2476601	nissense_varia	2.00E-11	New
22	41587553	20	T	A	41587556	1189	Schizophrenia	22q13.2	41191552	EP300-AS1	rs9607782	intron_variant	2.00E-11	Near_to_perfect
2	102851708	10	T	C	102851708	1592	Serum protein levels (sST2	2q12.1	102235248	IL1RL2	rs2302612	nissense_varia	2.00E-11	Perfect_to_near
4	89811195	20	G	T	89811195	3265	Interstitial lung disease	4q22.1	88890044	FAM13A	rs2609255	intron_variant	2.00E-11	Perfect_to_near
6	32381736	24	T	A	32381736	3250	ip ratio adjusted for body r	6p21.32	32413959	BTNL2 - HLA-DRA	rs7759742	atory_region_v	2.00E-11	Near_to_perfect
7	17287268	20	A	T	17287269	2287	Coffee consumption	7p21.1	17247645	C105375170 - LOC101927(	rs6968865	itergenic_varia	2.00E-11	Near_to_perfect
7	99115675	8	G	A	99115677	853	Blood metabolite levels	7q22.1	99518054	ZKSCAN5	rs12533251	intron_variant	2.00E-11	Near_to_perfect
8	60178720	16	C	A	60178721	1558	Hyperopia	8q12.1	59266162	LOC100505501 - NUDT15P	rs10089517	itergenic_varia	2.00E-11	Near_to_perfect
9	100591458	18	C	T	100591463	3285	Thyroid hormone levels	9q22.33	97829181	PTCSC2	rs7045138	itergenic_varia	2.00E-11	Near_to_perfect
2	44065097	22	G	A	44065090	336	Cholesterol, total	2p21	43837951	ABCG5, ABCG8	rs6756629	nissense_varia	2.00E-11	Smaller
11	32364187	18	G	A	32364187	898	Tuberculosis	11p13	32342641	RCN1 - WT1	rs2057178	intron_variant	3.00E-11	Perfect_to_near
14	79936964	8	T	C	79936964	1031	Body mass index	14q31.1	79470621	NRXN3	rs10150332	intron_variant	3.00E-11	Perfect_to_near
17	69108749	14	G	T	69108753	2880	Prostate cancer	17q24.3	71112612	CASC17	rs1859962	intron_variant	3.00E-11	Near_to_perfect
18	46450972	16	A	G	46450976	4381	Colorectal cancer	18q21.1	48924606	SMAD7	rs7229639	intron_variant	3.00E-11	Near_to_perfect
19	45411944	8	T	C	45411941	754	Lewy body disease	19q13.32	44908684	APOE	rs429358	nissense_varia	3.00E-11	Perfect_to_near
20	60969456	18	C	T	60969451	1014	Colorectal cancer	20q13.33	62394395	CABLES2	rs2427308	intron_variant	3.00E-11	Near_to_perfect
20	60969456	18	C	T	60969451	1014	Colorectal cancer	20q13.33	62394395	CABLES2	rs2427308	intron_variant	3.00E-11	Near_to_perfect
3	85884151	20	A	G	85884150	439	Obesity	3p12.1	85835000	CADM2	rs13078807	intron_variant	3.00E-11	Near_to_perfect
4	89713119	12	C	T	89713121	3041	ip ratio adjusted for body r	4q22.1	88791970	FAM13A	rs9991328	intron_variant	3.00E-11	Perfect_to_near
5	1282416	8	G	A	1282414	2045	Prostate cancer	5p15.33	1282299	TERT	rs7725218	intron_variant	3.00E-11	Perfect_to_near
8	118185729	20	A	G	118185733	1302	asting glucose-related trai	8q24.11	117173494	SLC30A8, LOC105375716	rs11558471	rime_UTR_var	3.00E-11	Near_to_perfect
10	114758351	14	C	T	114758349	1141	Body mass index	10q25.2	112998590	TCF7L2	rs7903146	intron_variant	4.00E-11	Near_to_perfect
11	64478061	10	G	A	64478063	3597	Urate levels	11q13.1	64710591	NRXN2	rs478607	intron_variant	4.00E-11	Perfect_to_near
11	133822566	8	A	G	133822569	457	Schizophrenia	11q25	133952674	IGSF9B	rs75059851	intron_variant	4.00E-11	Near_to_perfect
3	85884151	20	A	G	85884150	439	Body mass index	3p12.1	85835000	CADM2	rs13078807	intron_variant	4.00E-11	Near_to_perfect
6	31391399	14	T	C	31391401	388	HIV-1 susceptibility	6p21.33	31423624	MICA - LOC105375017	rs4418214	intron_variant	4.00E-11	Perfect_to_near
6	32381736	24	T	A	32381736	3250	ip ratio adjusted for body r	6p21.32	32413959	BTNL2 - HLA-DRA	rs7759742	atory_region_v	4.00E-11	Near_to_perfect
12	65718295	20	T	G	65718299	135	Hippocampal volume	12q14.3	65324519	MSRB3	rs17178006	intron_variant	5.00E-11	Perfect_to_near
14	25928176	20	C	A	25928179	1529	Body mass index	14q12	25458973	HMGN2P6 - LOC10537041(	rs10132280	itergenic_varia	5.00E-11	Perfect_to_near
14	36738361	18	C	T	36738361	57	Thyroid cancer	14q13.3	36269155	C101927199 - LOC10272з	rs116909374	atory_region_v	5.00E-11	Near_to_perfect
1	47347423	0	A	T	47347427	1014	Blood metabolite levels	1p33	46881755	CYP4Z2P	rs6663731	intron_variant	5.00E-11	New
2	181996040	14	A	G	181996045	2901	Celiac disease	2q31.3	181131318	LOC101927156	rs13010713	intron_variant	5.00E-11	Perfect_to_near
2	230224037	0	T	C	230224031	3897	lmonary function (interacti	2q36.3	229359315	DNER	rs7594321	intron_variant	5.00E-11	New
2	233406992	0	T	G	233406998	4098	Refractive error	2q37.1	232542288	CHRNG	rs1881492	intron_variant	5.00E-11	New
2	177042638	20	A	C	177042633	4094	Ovarian cancer	2q31.1	176177905	HAGLR	rs2072590	g_transcript_ex	5.00E-11	Non_identical
6	31074024	20	A	C	31074030	3418	Follicular lymphoma	6p21.33	31106253	HCG22 - C6orf15	rs6457327	stream_gene_v	5.00E-11	Non_identical
11	20253599	20	A	G	20253599	3252	ticoid treatment in asthma	11p15.1	20232053	LOC105376583 - HTATIP2	rs1353649	itergenic_varia	6.00E-11	Perfect_to_near
4	155389244	8	T	C	155389247	112	Bilirubin levels	4q31.3	154468095	DCHS2	rs17031671	intron_variant	6.00E-11	Near_to_perfect
5	64625510	8	C	T	64625512	1175	Corneal structure	5q12.3	65329685	ADAMTS6	rs2307121	intron_variant	6.00E-11	Perfect_to_near
7	16707859	12	A	G	16707861	545	mer's disease (cognitive d	7p21.1	16668236	BZW2	rs58370486	intron_variant	6.00E-11	Near_to_perfect
19	55819849	14	C	T	55819845	1555	Menopause (age at onset)	19q13.42	55308477	BRSK1	rs1172822	intron_variant	6.00E-11	Non_identical
3	170725548	20	G	A	170725542	997	e levels (gamma-glutamyl	3q26.2	171007753	SLC2A2	rs10513686	intron_variant	6.00E-11	Non_identical
11	128492736	8	T	C	128492739	2282	Follicular lymphoma	11q24.3	128622844	C105369568 - LOC101929	rs4937362	ream_gene_va	7.00E-11	Perfect_to_near
3	52567016	24	G	T	52567014	2301	ip ratio adjusted for body r	3p21.1	52532998	NT5DC2	rs12489828	intron_variant	7.00E-11	Perfect_to_near
8	53125735	10	A	C	53125734	1082	Motion sickness	8q11.23	52213174	ST18	rs2360806	intron_variant	7.00E-11	Near_to_perfect
10	97395957	20	T	C	97395962	1001	Blood metabolite levels	10q24.1	95636205	ALDH18A1	rs56322409	intron_variant	8.00E-11	Near_to_perfect
2	233379938	0	G	A	233379941	753	Refractive error	2q37.1	232515231	ECEL1 - PRSS56	rs1656404	itergenic_varia	8.00E-11	New
5	40486892	20	G	T	40486896	1814	Self-reported allergy	5p13.1	40486794	C105374736 - LOC105374	rs7720838	atory_region_v	8.00E-11	Near_to_perfect

11	74345542	20	T	G	74345550	1637	Colorectal cancer	11q13.4	74634505	POLD3	rs3824999	intron_variant	8.00E-11	Non_identical
11	119099909	12	G	C	119099906	708	Platelet count	11q23.3	119229196	CBL	rs4938642	intron_variant	8.00E-11	Non_identical
10	45958879	8	A	C,G	45958881	1,028,250	Blood metabolite levels	10q11.21	45463433	8-Mar	rs2291429	nissense_variai	9.00E-11	Near_to_perfect
17	38069949	8	C	T	38069949	3345	Asthma	17q21.1	39913696	GSDMB	rs7216389	intron_variant	9.00E-11	Near_to_perfect
2	204729152	18	C	T	204729153	2171	Myasthenia gravis	2q33.2	203864430	LOC105373844	rs231770	ream_gene_va	9.00E-11	Perfect_to_near
2	234827661	0	G	A	234827661	281	Migraine without aura	2q37.1	233919016	TRPM8	rs6741751	intron_variant	9.00E-11	New
16	88298117	18	C	T	88298124	2705	Central corneal thickness	16q24.2	88264518	ZNF469	rs12447690	intron_variant	9.00E-11	Non_identical
12	121420254	0	A	G	121420260	2814	C-reactive protein	12q24.31	120982457	HNF1A	rs7979473	intron_variant	1.00E-10	New
13	61113738	0	G	A	61113739	1277	Menopause (age at onset)	13q21.2	60539605	TDRD3	rs4886238	intron_variant	1.00E-10	New
15	31708262	10	C	T	31708263	982	ip ratio adjusted for body r	15q13.3	31416060	KLF13	rs8042543	intron_variant	1.00E-10	Perfect_to_near
15	59487928	18	C	G	59487930	355	Metabolite levels	15q22.2	59195731	MYO1E	rs2306786	intron_variant	1.00E-10	Perfect_to_near
16	20400832	24	C	T	20400839	1027	al function-related traits (s	16p12.3	20389517	PDILT	rs11864909	intron_variant	1.00E-10	Non_identical
19	45392254	24	C	T	45392254	553	heimer's disease biomark	19q13.32	44888997	PVRL2	rs6857	rime_UTR_var	1.00E-10	Near_to_perfect
5	134499090	0	C	A	134499092	2701	Colorectal cancer	5q31.1	135163402	C5orf66	rs647161	intron_variant	1.00E-10	New
6	32577380	18	A	G	32577380	994	IgA nephropathy	6p21.32	32609603	HLA-DRB1 - HLA-DQA1	rs660895	intron_variant	1.00E-10	Perfect_to_near
6	135423210	30	T	C	135423209	1520	White blood cell types	6q23.3	135102071	HBS1L - LOC105378010	rs9373124	intron_variant	1.00E-10	Perfect_to_near
7	120903815	8	A	T	120903815	1363	Bone mineral density	7q31.31	121263761	CPED1, LOC102724514	rs4609139	intron_variant	1.00E-10	Perfect_to_near
18	46587647	22	G	A	46587654	3069	Height	18q21.1	49061284	DYM	rs1787200	intron_variant	1.00E-10	Non_identical
1	22492878	22	G	C	22492887	1184	nineral density (paediatric	1p36.12	22166394	LOC105376850	rs3920498	itergenic_variai	1.00E-10	Non_identical
10	114758351	14	C	T	114758349	1141	Type 2 diabetes	10q25.2	112998590	TCF7L2	rs7903146	intron_variant	2.00E-10	Near_to_perfect
10	123337334	16	A	G	123337335	2551	Breast cancer	10q26.13	121577821	FGFR2	rs2981579	intron_variant	2.00E-10	Near_to_perfect
11	117075565	8	C	T	117075566	386	liovascular disease risk fa	11q23.3	117204850	TAGLN - PCSK7	rs508487	rime_UTR_var	2.00E-10	Perfect_to_near
20	46095645	20	G	A	46095649	179	Osteoarthritis (hip)	20q13.12	47466905	LOC102723442 - NCOA3	rs6094710	atory_region_v	2.00E-10	Perfect_to_near
2	231106716	0	C	T	231106724	465	Multiple sclerosis	2q37.1	230242009	SP140	rs10201872	intron_variant	2.00E-10	New
6	26091176	16	C	G	26091179	366	Hypertension	6p22.2	26090951	HFE	rs1799945	nissense_variai	2.00E-10	Perfect_to_near
9	136925665	10	G	T	136925663	574	Platelet count	9q34.2	134060541	BRD3	rs11789898	intron_variant	2.00E-10	Near_to_perfect
1	162014626	18	A	G	162014632	3091	QT interval	1q23.3	162044842	OLFML2B - NOS1AP	rs2880058	itergenic_variai	2.00E-10	Bigger
6	32409534	22	G	A	32409530	2545	Parkinson's disease	6p21.32	32441753	HLA-DRA	rs3129882	intron_variant	2.00E-10	Non_identical
7	120983346	24	A	G	120983343	1301	Bone mineral density	7q31.31	121343289	WNT16 - FAM3C	rs10242100	stream_gene_v	2.00E-10	Non_identical
8	9183600	16	A	G	9183596	4541	ulin-related traits (interactio	8p23.1	9326086	LOC157273	rs4841132	g_transcript_ex	2.00E-10	Non_identical
13	22131899	8	C	T	22131897	1629	PR segment	13q12.11	21557758	MICU2	rs2798269	intron_variant	3.00E-10	Near_to_perfect
17	69108749	14	G	T	69108753	2880	Prostate cancer	17q24.3	71112612	CASC17	rs1859962	intron_variant	3.00E-10	Near_to_perfect
1	154991390	0	T	C	154991389	878	ip ratio adjusted for body r	1q21.3	155018913	DCST2	rs905938	intron_variant	3.00E-10	New
1	154991390	0	T	C	154991389	878	ip ratio adjusted for body r	1q21.3	155018913	DCST2	rs905938	intron_variant	3.00E-10	New
2	31411292	18	T	C	31411287	141	Eosinophilic esophagitis	2p23.1	31188421	CAPN14	rs77569859	intron_variant	3.00E-10	Near_to_perfect
3	52567016	24	G	T	52567014	2301	ip ratio adjusted for body r	3p21.1	52532998	NT5DC2	rs12489828	intron_variant	3.00E-10	Perfect_to_near
3	52567016	24	G	T	52567014	2301	ip ratio adjusted for body r	3p21.1	52532998	NT5DC2	rs12489828	intron_variant	3.00E-10	Perfect_to_near
4	89713119	12	C	T	89713121	3041	ip ratio adjusted for body r	4q22.1	88791970	FAM13A	rs9991328	intron_variant	3.00E-10	Perfect_to_near
9	15289578	18	C	T	15289578	4481	HDL cholesterol	9p22.3	15289580	TTC39B	rs471364	intron_variant	3.00E-10	Near_to_perfect
2	44065097	22	G	A	44065090	336	LDL cholesterol	2p21	43837951	ABCG5, ABCG8	rs6756629	nissense_variai	3.00E-10	Smaller
2	165540806	26	T	C	165540800	544	HDL cholesterol	2q24.3	164684290	COBLL1	rs12328675	rime_UTR_var	3.00E-10	Non_identical
2	177042638	20	A	C	177042633	4094	Ovarian cancer	2q31.1	176177905	HAGLR	rs2072590	g_transcript_ex	3.00E-10	Non_identical
16	20400832	24	C	T	20400839	1027	function-related traits (eGF	16p12.3	20389517	PDILT	rs11864909	intron_variant	4.00E-10	Non_identical
16	50756773	12	C	G,T	50756774	2,259	nflammatory bowel diseas	16q12.1	50722863	NOD2	rs5743289	intron_variant	4.00E-10	Perfect_to_near
3	43785572	8	G	A	43785574	904	Inflammatory skin disease	3p21.33	43744082	ABHD5 - LOC105377054	rs75594032	itergenic_variai	4.00E-10	Near_to_perfect
3	52506425	14	C	T	52506426	4823	Waist-hip ratio	3p21.1	52472410	NISCH	rs6784615	intron_variant	4.00E-10	Perfect_to_near
5	134499090	0	C	A	134499092	2701	Colorectal cancer	5q31.1	135163402	C5orf66	rs647161	intron_variant	4.00E-10	New
6	19785594	16	C	T	19785588	3335	Endometriosis	6p22.3	19785357	C105374962 - LOC100506	rs7739264	intron_variant	4.00E-10	Smaller
6	32668103	8	G	A	32668100	1703	Lymphoma	6p21.32	32700323	HLA-DQB1 - LOC10272501	rs2647045	intron_variant	4.00E-10	Near_to_perfect
6	159801027	8	A	G	159801024	1296	protein (a) - cholesterol le	6q25.3	159379992	LOC105378084	rs1544167	ream_gene_va	4.00E-10	Bigger
11	74345542	20	T	G	74345550	1637	Colorectal cancer	11q13.4	74634505	POLD3	rs3824999	intron_variant	4.00E-10	Non_identical
22	32867522	14	G	A	32867528	979	Hematological parameters	22q12.3	32471541	BPIFC - FBXO7	rs9609565	ream_gene_va	4.00E-10	Non_identical
4	109016818	16	C	A	109016824	2598	hronic lymphocytic leukem	4q25	108095668	LEF1	rs898518	intron_variant	4.00E-10	Non_identical
1	154991390	0	T	C	154991389	878	ip ratio adjusted for body r	1q21.3	155018913	DCST2	rs905938	intron_variant	5.00E-10	New
4	89713119	12	C	T	89713121	3041	ip ratio adjusted for body r	4q22.1	88791970	FAM13A	rs9991328	intron_variant	5.00E-10	Perfect_to_near
8	128815027	14	T	C	128815029	3586	Allergic sensitization	8q24.21	127802783	MIR1204 - PVT1	rs4410871	intron_variant	5.00E-10	Near_to_perfect
9	126525213	20	A	G	126525212	2073	Polycystic ovary syndrome	9q33.3	123762933	DENND1A	rs2479106	intron_variant	5.00E-10	Perfect_to_near
6	32409534	22	G	A	32409530	2545	Parkinson's disease	6p21.32	32441753	HLA-DRA	rs3129882	intron_variant	5.00E-10	Non_identical
10	80925580	26	G	C	80925577	1701	Height	10q22.3	79165820	ZMIZ1	rs7916441	intron_variant	6.00E-10	Non_identical
10	93348115	14	G	T	93348120	1987	Smoking behavior	10q23.32	91588363	HECTD2-AS1	rs1329650	atory_region_v	6.00E-10	Near_to_perfect
10	94462877	14	C	T	94462882	2285	Type 2 diabetes	10q23.33	92703125	HHEX - EXOC6	rs1111875	atory_region_v	6.00E-10	Near_to_perfect
10	94462877	14	C	T	94462882	2285	Type 2 diabetes	10q23.33	92703125	HHEX - EXOC6	rs1111875	atory_region_v	6.00E-10	Near_to_perfect
12	14653867	16	C	T	14653867	1337	Testicular germ cell cance	12p13.1	14500933	ATF7IP	rs2900333	rime_UTR_var	6.00E-10	Near_to_perfect
16	30482492	0	T	C	30482494	2968	Ulcerative colitis	16p11.2	30471173	SEPHS2 - ITGAL	rs11150589	ream_gene_va	6.00E-10	New
2	240900123	8	C	T	240900121	1249	nt perception (isobutyralde	2q37.3	239960704	NDUFA10	rs13424612	rime_UTR_var	6.00E-10	Near_to_perfect
7	100453219	24	T	C	100453208	457	Resting heart rate	7q22.1	100855586	SLC12A9	rs314370	g_transcript_ex	6.00E-10	Near_to_perfect
9	136154866	18	G	T	136154867	803	atological and biochemical	9q34.2	133279294	ABO - SURF6	rs495828	ream_gene_va	6.00E-10	Near_to_perfect
1	154991390	0	T	C	154991389	878	ip ratio adjusted for body r	1q21.3	155018913	DCST2	rs905938	intron_variant	7.00E-10	New
10	56777028	20	T	C	56777024	2694	Sporadic pituitary adenoma	10q21.1	55017264	PCDH15 - MTRNR2L5	rs10763170	intron_variant	7.00E-10	Non_identical
18	46587647	22	G	A	46587654	3069	Height	18q21.1	49061284	DYM	rs1787200	intron_variant	7.00E-10	Non_identical
12	131456443	14	G	A	131456449	450	rophic lateral sclerosis (sp	12q24.33	130971904	ADGRD1	rs11061269	intron_variant	8.00E-10	Near_to_perfect
1	114377568	0	A	G	114377568	4871	Myasthenia gravis	1p13.2	113834946	PTPN22, AP4B1-AS1	rs2476601	nissense_variai	8.00E-10	New
4	82165791	8	T	C	82165790	1956	Height	4q21.21	81244636	PRKG2 - RASGEF1B	rs994014	itergenic_variai	8.00E-10	Perfect_to_near
1	2387105	14	C	T	2387101	3122	Schizophrenia	1p36.32	2455662	PLCH2	rs4648845	intron_variant	9.00E-10	Non_identical
1	112392365	22	G	T	112392360	923	onse to smallpox (secrete	1p13.2	111849738	KCND3	rs12044963	intron_variant	9.00E-10	Non_identical
11	2857193	12	A	C	2857194	1617	Type 2 diabetes	11p15.4	2835964	KCNQ1	rs2237895	intron_variant	1.00E-09	Near_to_perfect
11	102887231	16	A	G	102887229	540	iisocyanate-induced asthn	11q22.3	103016500	MMP13 - RPL21P96	rs74380195	itergenic_variai	1.00E-09	Perfect_to_near
15	31708262	10	C	T	31708263	982	ip ratio adjusted for body r	15q13.3	31416060	KLF13	rs8042543	intron_variant	1.00E-09	Perfect_to_near
17	26694859	10	G	A	26694861	2780	Osteoprotegerin levels	17q11.2	28367840	VTN	rs704	nissense_variai	1.00E-09	Perfect_to_near
1	249168435	16	G	A	249168436	920	Myopia (pathological)	1q44	248874237	PGBD2	rs12032643	itergenic_variai	1.00E-09	Near_to_perfect

2	234679388	0	C	T	234679384	1043	Cholesterol, total	2q37.1	233770738	JGT1A7, UGT1A5, UGT1A9	rs11563251	rime_UTR_var	1.00E-09	New
3	52567016	24	G	T	52567014	2301	p ratio adjusted for body r	3p21.1	52532998	NT5DC2	rs12489828	intron_variant	1.00E-09	Perfect_to_near
3	133508465	18	A	T	133508464	1623	Iron status biomarkers	3q22.1	133789620	LOC105374116, SRPRB	rs1830084	intron_variant	1.00E-09	Near_to_perfect
5	31020519	18	A	G	31020521	1299	e levels (gamma-glutamyl	5p13.3	31020414	LOC102723637 - RPL19P1	rs6888304	ntergenic_varia	1.00E-09	Near_to_perfect
6	31312327	22	T	G	31312326	349	Psoriasis	6p21.33	31344549	LOC105375015 - HLA-B	rs3134792	ntergenic_varia	1.00E-09	Perfect_to_near
7	38136278	24	T	C	38136277	2777	one mineral density (spine	7p14.1	38096675	LOC105375236 - STARD3N	rs1524058	ntergenic_varia	1.00E-09	Perfect_to_near
8	126481472	0	A	T	126481475	3560	Adiponectin levels	8q24.13	125469233	LOC105375745	rs2980879	intron_variant	1.00E-09	New
11	69238128	16	C	T	69238123	445	Renal cell carcinoma	11q13.3	69423355	C105369370 - LOC102724	rs11263654	ream_gene_va	1.00E-09	Non_identical
10	35287642	20	T	G	35287650	1486	Crohn's disease	10p11.21	34998722	LOC105376492	rs17582416	ntergenic_varia	2.00E-09	Smaller
11	75276179	8	A	C	75276178	3484	Height	11q13.5	75565133	SERPINH1	rs606452	intron_variant	2.00E-09	Near_to_perfect
11	116653295	8	C	T	116653296	4301	Metabolic syndrome	11q23.3	116782580	ZPR1	rs2075290	intron_variant	2.00E-09	Near_to_perfect
12	48419614	18	A	C	48419618	191	Prostate cancer	12q13.11	48025835	LOC105369750	rs80130819	ream_gene_va	2.00E-09	Perfect_to_near
15	51530495	12	G	A	51530495	1157	Height	15q21.2	51238298	CYP19A1, PIRC66	rs16964211	intron_variant	2.00E-09	Near_to_perfect
15	80432224	0	A	G	80432222	2181	Type 2 diabetes	15q25.1	80139880	ZFAND6 - FAH	rs11634397	stream_gene_v	2.00E-09	New
16	82519227	8	C	T	82519228	3926	icrostructure (global fractio	16q23.3	82485623	C100419639 - LOC101928	rs1991867	ntergenic_varia	2.00E-09	Perfect_to_near
17	59239220	18	A	G	59239221	3043	al function-related traits (E	17q23.2	61161860	BCAS3	rs11868441	intron_variant	2.00E-09	Perfect_to_near
19	6690773	20	C	T	6690771	401	omplement C3 and C4 leve	19p13.3	6690760	C3	rs3745567	intron_variant	2.00E-09	Perfect_to_near
1	38633874	0	A	T	38633879	763	Rheumatoid arthritis	1p34.3	38168207	LOC105378654	rs12140275	ntergenic_varia	2.00E-09	New
1	222164944	14	A	G	222164948	954	Colorectal cancer	1q41	221991606	C105372950 - LOC105372	rs6687758	atory_region_v	2.00E-09	Near_to_perfect
2	46537599	12	A	G	46537604	2132	Renal cell carcinoma	2p21	46310465	LOC105374583, EPAS1	rs7579899	intron_variant	2.00E-09	Near_to_perfect
2	68570773	18	G	C,T	68570772	2015,18	Metabolite levels	2p14	68343640	LOC102724389	rs9309413	ntergenic_varia	2.00E-09	Near_to_perfect
2	152957412	16	T	C	152957411	1399	to chemotherapy in breas	2q23.3	152100897	CACNB4 - STAM2	rs3820706	intron_variant	2.00E-09	Near_to_perfect
2	234508962	0	C	T	234508963	955	bilirubin levels in HIV-1 inf	2q37.1	233600317	USP40 - UGT1A8	rs2741012	ream_gene_va	2.00E-09	New
4	88799711	18	T	C	88799710	1506	Bone mineral density	4q22.1	87878558	MEPE - HSP90AB3P	rs1463104	ntergenic_varia	2.00E-09	Perfect_to_near
6	128390979	8	T	C	128390980	4544	Menarche (age at onset)	6q22.33	128069835	PTPRK, LOC101928140	rs6938574	intron_variant	2.00E-09	Perfect_to_near
6	32741876	18	T	C	32741868	1433	Lymphoma	6p21.32	32774091	HLA-DQB2 - HLA-DOB	rs2621416	ntergenic_varia	2.00E-09	Smaller
7	38128333	20	T	C	38128326	3221	Height	7p14.1	38088724	LOC105375236 - STARD3N	rs6959212	ntergenic_varia	2.00E-09	Non_identical
8	9183600	16	A	G	9183596	4541	Metabolite levels	8p23.1	9326086	LOC157273	rs4841132	g_transcript_ex	2.00E-09	Non_identical
13	43128580	8	T	C	43128577	1742	eral density (paediatric, up	13q14.11	42554441	LOC105370177 - TNFSF11	rs9525638	atory_region_v	3.00E-09	Perfect_to_near
13	73728135	18	G	T	73728139	2305	Prostate cancer	13q22.1	73154002	RNU6-66P - LINC00393	rs9600079	ntergenic_varia	3.00E-09	Perfect_to_near
16	56995229	30	C	A	56995236	2737	Lipid traits	16q13	56961324	HERPUD1 - CETP	rs1800775	ream_gene_va	3.00E-09	New
18	73098949	20	C	A	73098949	514	Motion sickness	18q22.3	75386994	TSHZ1 - LOC105372200	rs10514168	ream_gene_va	3.00E-09	Near_to_perfect
19	11347490	20	T	C	11347493	1244	HDL cholesterol	19p13.2	11236817	DOCK6	rs737337	nonymous_vari	3.00E-09	Near_to_perfect
1	156255447	20	G	A	156255456	1016	puscular hemoglobin conc	1q22	156285665	TMEM79	rs6684514	nissense_varia	3.00E-09	Near_to_perfect
1	163441281	14	T	C	163441286	2025	Motion sickness	1q23.3	163471496	C100422212 - LOC105371	rs4076764	ream_gene_va	3.00E-09	Perfect_to_near
1	169543132	12	G	A	169543131	1359	Optic disc area	1q24.2	169573893	F5	rs12406092	intron_variant	3.00E-09	Near_to_perfect
1	218698025	12	C	T	218698027	1339	histogram emphysema pa	1q41	218524685	TGFB2 - LOC105372924	rs1690789	intron_variant	3.00E-09	Near_to_perfect
2	166978752	8	C	A	166978750	1062	l lobe epilepsy with hippoc	2q24.3	166122240	SCN1A, LOC101929680	rs7587026	intron_variant	3.00E-09	Near_to_perfect
2	230224037	0	T	C	230224031	3897	lmonary function (interacti	2q36.3	229359315	DNER	rs7594321	intron_variant	3.00E-09	New
6	26091176	16	C	G	26091179	366	Iron status biomarkers	6p22.2	26090951	HFE	rs1799945	nissense_varia	3.00E-09	Perfect_to_near
7	28174986	16	T	C	28174986	2629	Rheumatoid arthritis	7p15.1	28135367	JAZF1	rs67250450	intron_variant	3.00E-09	Perfect_to_near
7	137074842	0	T	C	137074844	1392	Schizophrenia	7q33	137390098	DGKI	rs3735025	rime_UTR_var	3.00E-09	New
9	3929428	0	C	T	3929424	3924	heimer's disease biomark	9p24.2	3929424	GLIS3	rs514716	intron_variant	3.00E-09	New
21	36738246	18	G	A	36738242	3168	Rheumatoid arthritis	21q22.12	35365944	RUNX1 - LOC100506403	rs8133843	intron_variant	3.00E-09	Non_identical
3	17124372	28	A	G	17124384	3008	Myocardial infarction	3p24.3	17082892	PLCL2	rs4618210	intron_variant	3.00E-09	Smaller
4	15737941	24	A	G	15737937	1547	Parkinson's disease	4p15.32	15736314	BST1	rs4538475	intron_variant	3.00E-09	Bigger
13	43128580	8	T	C	43128577	1742	Cortical thickness	13q14.11	42554441	LOC105370177 - TNFSF11	rs9525638	atory_region_v	4.00E-09	Perfect_to_near
17	73687494	18	G	A	73687495	1116	Rotator cuff tears	17q25.1	75691415	SAP30BP	rs820218	g_transcript_ex	4.00E-09	Perfect_to_near
1	38633874	0	A	T	38633879	763	Rheumatoid arthritis	1p34.3	38168207	LOC105378654	rs12140275	ntergenic_varia	4.00E-09	New
1	114377568	0	A	G	114377568	4871	Crohn's disease	1p13.2	113834946	PTPN22, AP4B1-AS1	rs2476601	nissense_varia	4.00E-09	New
21	45709153	0	G	A	45709153	1315	Rheumatoid arthritis	21q22.3	44289270	AIRE	rs2075876	g_transcript_ex	4.00E-09	New
22	49812498	24	C	A	49812502	117	ponse to smallpox (secrete	22q13.33	49418853	C22orf34	rs17000918	intron_variant	4.00E-09	Near_to_perfect
2	127950988	20	C	T	127950997	643	Protein C levels	2q14.3	127193421	CYP27C1	rs4321325	intron_variant	4.00E-09	Near_to_perfect
2	128351503	12	C	T	128351504	65	Protein C levels	2q14.3	127593929	MYO7B	rs13419716	intron_variant	4.00E-09	Perfect_to_near
4	180356844	8	C	T	180356846	2465	Motion sickness	4q34.3	179435692	C105377563 - LOC105377	rs1378552	intron_variant	4.00E-09	Near_to_perfect
6	73643287	8	C	A	73643289	3454	Refractive error	6q13	72933566	KCNQ5	rs7744813	intron_variant	4.00E-09	Near_to_perfect
7	28174986	16	T	C	28174986	2629	Rheumatoid arthritis	7p15.1	28135367	JAZF1	rs67250450	intron_variant	4.00E-09	Perfect_to_near
7	151406003	16	C	T	151406005	788	Urate levels	7q36.1	151708919	PRKAG2	rs10480300	intron_variant	4.00E-09	Near_to_perfect
17	45425125	22	G	A	45425115	2058	LDL cholesterol	17q21.32	47347749	EFCAB13	rs7206971	intron_variant	4.00E-09	Non_identical
17	38121991	10	G	A	38121993	2158	Asthma	17q21.1	39965740	GSDMA	rs3894194	nissense_varia	5.00E-09	Perfect_to_near
2	234184413	0	G	A	234184417	1868	Crohn's disease	2q37.1	233275771	SCARNA5, ATG16L1	rs3792109	g_transcript_ex	5.00E-09	New
2	237105519	0	G	A	237105518	1116	Educational attainment	2q37.2	236196875	ASB18	rs13401104	intron_variant	5.00E-09	New
3	17859365	28	T	C	17859366	3269	Schizophrenia	3p24.3	17817874	LOC105376975 - PP1P	rs4330281	intron_variant	5.00E-09	Near_to_perfect
4	31397616	0	G	A	31397618	2975	Lipid traits	4p15.1	31395996	C102723778 - LOC105374	rs6448771	ntergenic_varia	5.00E-09	New
5	95850245	18	A	C	95850250	2058	Body mass index	5q15	96514546	LOC101929710	rs261967	intron_variant	5.00E-09	Perfect_to_near
10	94481927	26	G	A	94481917	2148	Multiple sclerosis	10q23.33	92722160	HHEX - EXOC6	rs7923837	ntergenic_varia	5.00E-09	Non_identical
5	45364879	14	T	C	45364875	1999	Schizophrenia	5p12	45364773	HCN1	rs1501357	intron_variant	5.00E-09	Bigger
11	116633869	18	G	A	116633862	445	bolic syndrome (bivariate t	11q23.3	116763146	BUD13	rs11820589	nissense_varia	6.00E-09	Smaller
19	45406671	10	G	A	45406673	1240	Cognitive function	19q13.32	44903416	TOMM40	rs10119	rime_UTR_var	6.00E-09	Perfect_to_near
19	49208985	16	A	G	49208978	1564	Serum lipase activity	19q13.33	48705721	FUT2	rs632111	rime_UTR_var	6.00E-09	Near_to_perfect
3	141137035	22	G	A	141137035	824	Height	3q23	141418193	ZBTB38	rs9825379	intron_variant	6.00E-09	Near_to_perfect
6	77168087	16	C	T	77168086	2166	Menarche (age at onset)	6q14.1	76458369	IMPG1 - LOC105377860	rs9447700	ntergenic_varia	6.00E-09	Near_to_perfect
13	54102212	20	G	A	54102206	783	Body mass index	13q14.3	53528071	LINC01065 - LOC10537021	rs12429545	ntergenic_varia	6.00E-09	Non_identical
8	9184684	20	T	C	9184691	4485	HDL cholesterol	8p23.1	9327181	LOC157273	rs6601299	intron_variant	6.00E-09	Non_identical
11	34805846	18	A	G	34805849	2504	tive pulmonary disease-rel	11p13	34784302	LOC102723568	rs7929679	ntergenic_varia	7.00E-09	Absent
18	57849022	16	G	A	57849023	1206	HDL cholesterol	18q21.32	60181790	C105372154 - LOC105372	rs12967135	ntergenic_varia	7.00E-09	Perfect_to_near
1	18979880	0	G	T	18979874	1450	Orofacial clefts	1p36.13	18653380	PAX7	rs742071	intron_variant	7.00E-09	New
1	218698025	12	C	T	218698027	1339	histogram emphysema pa	1q41	218524685	TGFB2 - LOC105372924	rs1690789	intron_variant	7.00E-09	Near_to_perfect
6	32575734	8	A	G	32575735	376	emia vasculitis in chronic h	6p21.32	32607958	HLA-DRB1 - HLA-DQA1	rs9461776	intron_variant	7.00E-09	Near_to_perfect
6	90657783	0	T	C	90657783	2861	IgG glycosylation	6q15	89948064	BACH2	rs404256	intron_variant	7.00E-09	New

7	94061400	10	G	T	94061404	24	l in Tripanosoma cruzi ser	7q21.3	94432092	COL1A2 - LOC105375404	rs115744676	stream_gene_v	7.00E-09	Near_to_perfect
8	126481472	0	A	T	126481475	3560	Adiponectin levels	8q24.13	125469233	LOC105375745	rs2980879	intron_variant	7.00E-09	New
8	126523521	16	C	T	126523523	4132	Triglycerides	8q24.13	125511281	LOC105375745	rs4360309	intron_variant	7.00E-09	Perfect_to_near
4	75160817	22	C	T	75160824	756	ponse to smallpox (secrete	4q13.3	74295107	LOC105377276, MTHFD2L	rs16850864	intron_variant	7.00E-09	Non_identical
10	44864165	10	C	T	44864168	169	(ddI/d4T) in HIV-1 infectio	10q11.21	44368720	LOC105378278 - CXCL12	rs266095	stream_gene_v	8.00E-09	Near_to_perfect
11	31409442	22	A	G	31409438	37	Glaucoma	11p13	31387891	DNAJC24	rs542340	intron_variant	8.00E-09	Bigger
13	43143027	20	C	T	43143029	1643	density (paediatric, total bo	13q14.11	42568893	TNFSF11	rs17536328	intron_variant	8.00E-09	Perfect_to_near
1	79238010	22	T	C	79238015	1145	Bipolar disorder	1p31.1	78772330	LOC652549 - ADGRL4	rs4650608	ntergenic_varia	8.00E-09	Near_to_perfect
1	79238010	22	T	C	79238015	1145	nizophrenia or bipolar disor	1p31.1	78772330	LOC652549 - ADGRL4	rs4650608	ntergenic_varia	8.00E-09	Near_to_perfect
1	184020941	16	G	A	184020945	1522	Height	1q25.3	184051811	TSEN15	rs2274432	nissense_varia	8.00E-09	Near_to_perfect
1	218698025	12	C	T	218698027	1339	histogram emphysema pa	1q41	218524685	TGFB2 - LOC105372924	rs1690789	intron_variant	8.00E-09	Near_to_perfect
2	142767431	8	C	T	142767433	1606	Motion sickness	2q22.2	142009864	LRP1B	rs34311235	intron_variant	8.00E-09	Near_to_perfect
2	193848338	8	C	A	193848340	1339	Schizophrenia	2q32.3	192983614	PCGEM1 - LOC645314	rs59979824	ntergenic_varia	8.00E-09	Near_to_perfect
4	87958396	16	G	A	87958395	2162	ystemic lupus erythematos	4q21.3	87037243	AFF1	rs340630	intron_variant	8.00E-09	Near_to_perfect
6	20679710	20	A	G	20679709	2067	Type 2 diabetes	6p22.3	20679478	CDKAL1	rs7756992	intron_variant	8.00E-09	Perfect_to_near
8	128815027	14	T	C	128815029	3586	Multiple sclerosis	8q24.21	127802783	MIR1204 - PVT1	rs4410871	intron_variant	8.00E-09	Near_to_perfect
8	9183600	16	A	G	9183596	4541	ose-related traits (interacti	8p23.1	9326086	LOC157273	rs4841132	g_transcript_ex	8.00E-09	Non_identical
10	62179809	18	C	T	62179812	617	Bipolar disorder	10q21.2	60420054	ANK3	rs10994336	intron_variant	9.00E-09	Near_to_perfect
14	35852476	0	T	C	35852486	2532	Inflammatory skin disease	14q13.2	35383280	PSMA6 - RPLP0P3	rs12884468	stream_gene_v	9.00E-09	New
16	49062588	10	C	A	49062590	2695	Body mass index	16q12.1	49028679	C105371240 - LOC10537112	rs2080454	ntergenic_varia	9.00E-09	Near_to_perfect
1	222164944	14	A	G	222164948	954	Colorectal cancer	1q41	221991606	C105372950 - LOC1053729	rs6687758	atory_region_v	9.00E-09	Near_to_perfect
2	145359906	8	C	T	145359909	699	Epilepsy (generalized)	2q22.3	144602342	LINC01412 - TEX41	rs10496964	ntergenic_varia	9.00E-09	Near_to_perfect
2	233077057	0	A	G	233077064	894	Height	2q37.1	232212354	DIS3L2	rs7571816	intron_variant	9.00E-09	New
3	141463290	18	A	C	141463298	930	patitis C induced liver fibro	3q23	141744456	RNF7	rs16851720	intron_variant	9.00E-09	Near_to_perfect
9	25804281	0	A	G	25804285	3485	Motion sickness	9p21.2	25804287	LINC01241	rs1782032	intron_variant	9.00E-09	New
17	26610256	18	C	T	26610252	322	rophic lateral sclerosis (sp	17q11.2	28283226	KRT18P55	rs34517613	intron_variant	9.00E-09	Non_identical
1	66086199	24	A	T	66086194	2824	C-reactive protein	1p31.3	65620511	LEPR	rs10889569	intron_variant	9.00E-09	Non_identical
20	57614008	18	A	G	57614002	1040	Mean platelet volume	20q13.32	59038947	SLMO2-ATP5E, SLMO2	rs151361	intron_variant	9.00E-09	Non_identical
10	63779871	18	C	T	63779871	744	Rheumatoid arthritis	10q21.2	62020112	ARID5B	rs71508903	intron_variant	1.00E-08	Perfect_to_near
10	114758351	14	C	T	114758349	1141	Type 2 diabetes	10q25.2	112998590	TCF7L2	rs7903146	intron_variant	1.00E-08	Near_to_perfect
11	61417471	8	G	A	61417472	1553	hospholipid levels (plasma	11q12.2	61650000	RPLP0P2 - DAGLA	rs1692120	stream_gene_v	1.00E-08	Near_to_perfect
11	102742758	24	G	T	102742761	311	histogram emphysema pa	11q22.2	102872031	MMP12	rs17368659	intron_variant	1.00E-08	Perfect_to_near
12	122781895	10	G	A	122781897	164	Body mass index	12q24.31	122297350	CLIP1	rs11057405	intron_variant	1.00E-08	Perfect_to_near
13	74520193	20	T	A	74520186	1842	QRS duration	13q22.1	73946049	KLF12	rs1886512	intron_variant	1.00E-08	Near_to_perfect
13	79410575	16	T	C	79410574	116	duct disorder (symptom co	13q31.1	78836439	LINC00331	rs11838918	ntergenic_varia	1.00E-08	Near_to_perfect
17	69125607	10	G	T	69125606	1453	lmonary function (interacti	17q24.3	71129465	CASC17	rs11654749	intron_variant	1.00E-08	Near_to_perfect
19	22359436	24	C	T	22359440	2210	Telomere length	19p12	22176638	PCGF7P - ZNF676	rs412658	g_transcript_ex	1.00E-08	Near_to_perfect
1	22587727	22	T	C	22587728	137	Colorectal cancer	1p36.12	22261235	LOC105376850 - MIR4418	rs72647484	ntergenic_varia	1.00E-08	Near_to_perfect
1	67685598	28	A	C	67685598	1963	Crohn's disease	1p31.3	67264945	41, IL23R, IL23R - LOC100	65804, rs1120	stream_gene_v	1.00E-08	Perfect_to_near
1	67730634	0	G	T	67730628	2171	Crohn's disease	1p31.3	67264945	41, IL23R, IL23R - LOC100	65804, rs1120	stream_gene_v	1.00E-08	New
1	109821507	18	T	G	109821511	3246	Coronary artery disease	1p13.3	109278889	CELSR2 - PSRC1	rs602633	stream_gene_v	1.00E-08	Perfect_to_near
1	114377568	0	A	G	114377568	4871	Crohn's disease	1p13.2	113834946	PTPN22, AP4B1-AS1	rs2476601	nissense_varia	1.00E-08	New
1	114377568	0	A	G	114377568	4871	Rheumatoid arthritis	1p13.2	113834946	PTPN22, AP4B1-AS1	rs2476601	nissense_varia	1.00E-08	New
20	16459308	18	G	A	16459309	1580	Intelligence	20p12.1	16480070	KIF16B	32541, rs6044	intron_variant	1.00E-08	Near_to_perfect
2	69287947	0	A	G	69287943	2815	Height	2p13.3	69060811	ANTXR1	rs4315565	intron_variant	1.00E-08	New
2	80281175	22	A	G	80281173	376	mer's disease (cognitive d	2p12	80054047	CTNNA2	rs6738962	intron_variant	1.00E-08	Perfect_to_near
2	174326852	18	C	T	174326845	544	rement (high sodium and p	2q31.1	173462117	OC105373744 - LOC64399	rs10930597	ntergenic_varia	1.00E-08	Smaller
2	227092798	24	A	G	227092802	3759	Body mass index	2q36.3	226228086	OC646736 - LOC10537391	rs2176040	ntergenic_varia	1.00E-08	Perfect_to_near
2	234827661	0	G	A	234827661	281	Migraine - clinic-based	2q37.1	233919016	TRPM8	rs6741751	intron_variant	1.00E-08	New
3	169082625	22	G	A	169082633	2424	Nasopharyngeal carcinoma	3q26.2	169364845	MECOM	rs6774494	intron_variant	1.00E-08	Smaller
3	186650792	10	A	G	186650790	4657	nduced liver injury (flucloxa	3q27.3	186933001	ST6GAL1	rs10937275	intron_variant	1.00E-08	Near_to_perfect
4	6270055	10	G	A	6270056	3719	pe 2 diabetes and other tra	4p16.1	6268329	LOC285484 - WFS1	rs4689388	ream_gene_va	1.00E-08	Near_to_perfect
9	3929428	0	C	T	3929424	3924	heimer's disease biomark	9p24.2	3929424	GLIS3	rs514716	intron_variant	1.00E-08	New
13	54102212	20	G	A	54102206	783	Body mass index	13q14.3	53528071	LINC01065 - LOC10537021	rs12429545	ntergenic_varia	1.00E-08	Non_identical
17	45425125	22	G	A	45425115	2058	Cholesterol, total	17q21.32	47347749	EFCAB13	rs7206971	intron_variant	1.00E-08	Non_identical
1	67722571	14	C	A	67722567	1965	Crohn's disease	1p31.3	67264945	41, IL23R, IL23R - LOC100	65804, rs1120	stream_gene_v	1.00E-08	Non_identical
6	127493605	18	A	T	127493611	2607	Metabolite levels	6q22.33	127172466	RSPO3, LOC105377988	rs6569474	intron_variant	1.00E-08	Non_identical
8	23777014	18	A	G	23777006	1525	Urate levels	8p21.2	23919493	STC1 - LOC105379328	rs17786744	ntergenic_varia	1.00E-08	Non_identical
9	118143940	16	C	T	118143933	911	Leprosy	9q33.1	115381654	1-Dec	rs10817758	intron_variant	1.00E-08	Bigger
10	94462877	14	C	T	94462882	2285	Type 2 diabetes	10q23.33	92703125	HHEX - EXOC6	rs1111875	atory_region_v	2.00E-08	Near_to_perfect
11	111275130	16	G	A	111275133	2459	Primary biliary cirrhosis	11q23.1	111404408	BTG4	rs4938534	intron_variant	2.00E-08	Near_to_perfect
11	120311196	22	A	T	120311198	1157	Intraocular pressure	11q23.3	120440489	ARHGEF12	rs4936518	intron_variant	2.00E-08	Perfect_to_near
12	122781895	10	G	A	122781897	164	Body mass index	12q24.31	122297350	CLIP1	rs11057405	intron_variant	2.00E-08	Perfect_to_near
13	31819327	16	T	C	31819325	1006	-related macular degenera	13q12.3	31245188	B3GALTL	rs9542236	intron_variant	2.00E-08	Perfect_to_near
13	43128580	8	T	C	43128577	1742	eral density (paediatric, up	13q14.11	42554441	LOC105370177 - TNFSF11	rs9525638	atory_region_v	2.00E-08	Perfect_to_near
16	75390306	0	T	A	75390316	2532	lmonary function (interacti	16q23.1	75356418	CFDP1	rs2865531	intron_variant	2.00E-08	New
18	46450972	16	A	G	46450976	4381	Colorectal cancer	18q21.1	48924606	SMAD7	rs7229639	intron_variant	2.00E-08	Near_to_perfect
18	62169090	20	A	T	62169092	1175	Trans fatty acid levels	18q22.1	64501857	OC284294 - LOC10537216	rs10469266	ntergenic_varia	2.00E-08	Near_to_perfect
1	41530873	8	T	C	41530871	3315	Height	1p34.2	41065199	SCMH1	rs6686842	intron_variant	2.00E-08	Near_to_perfect
1	49589845	20	A	G	49589847	2270	Body mass index	1p33	49124175	LOC105378706, AGBL4	rs657452	intron_variant	2.00E-08	Near_to_perfect
1	154991390	0	T	C	154991389	878	Birth length	1q21.3	155018913	DCST2	rs905938	intron_variant	2.00E-08	New
22	50971257	28	T	C	50971266	3100	Multiple sclerosis	22q13.33	50532837	ODF3B - LOC102724608	rs140522	ream_gene_va	2.00E-08	Near_to_perfect
2	37960612	12	A	T	37960613	95	Height	2p22.2	37733470	LOC105374465	rs17511102	intron_variant	2.00E-08	Near_to_perfect
2	43553950	0	T	C	43553949	3530	Prostate cancer	2p21	43326810	THADA	rs1465618	intron_variant	2.00E-08	New
2	162029109	10	T	G	162029113	260	(ddI/d4T) in HIV-1 infectio	2q24.2	161172602	TANK	rs7568498	intron_variant	2.00E-08	Perfect_to_near
2	232378227	0	T	C	232378231	3000	Height	2q37.1	231513520	LINC00471	rs6750795	ce_region_vari	2.00E-08	New
2	235302801	0	C	T	235302810	503	rement (high sodium and p	2q37.1	234394166	C105373933 - LOC1053739	rs11887188	ntergenic_varia	2.00E-08	New
2	239095424	8	G	A	239095422	1345	hospholipid levels (plasma	2q37.3	238186781	ILKAP	rs12472274	g_transcript_ex	2.00E-08	Perfect_to_near
3	16955259	22	G	A	16955259	1860	Primary biliary cirrhosis	3p24.3	16913767	PLCL2	rs1372072	intron_variant	2.00E-08	Perfect_to_near
3	181167583	8	T	A	181167585	1418	Schizophrenia	3q26.33	181449797	SOX2-OT	rs9841616	intron_variant	2.00E-08	Near_to_perfect

5	32804533	16	T	C	32804528	3011	Blood pressure	5p13.3	32804422	NPR3 - LOC340113	rs1173766	ıtergenic_variaı	2.00E-08	Non_identical
5	121178514	12	A	G	121178515	661	olume (Cerebrospinal fluic	5q23.1	121842820	)C105379149, LOC1053791	rs6887649	ıtergenic_variaı	2.00E-08	Near_to_perfect
6	116443733	18	T	A	116443735	2231	-related macular degenera	6q22.1	116122572	NT5DC1, COL10A1	rs3812111	intron_variant	2.00E-08	Near_to_perfect
7	26397234	20	C	A	26397239	3188	ip ratio adjusted for body r	7p15.2	26357619	SNX10	rs1534696	intron_variant	2.00E-08	Near_to_perfect
7	103527375	16	C	T	103527369	4041	Otosclerosis	7q22.1	103886922	RELN	rs3914132	intron_variant	2.00E-08	Smaller
8	27442126	8	C	A	27442127	204	Schizophrenia	8p21.1	27584610	EPHX2 - LOC105379341	rs73229090	intron_variant	2.00E-08	Near_to_perfect
8	40484241	24	T	C	40484239	3697	Fasting plasma glucose	8p11.21	40626720	ZMAT4	rs2722425	intron_variant	2.00E-08	Perfect_to_near
X	147042771	0	C	T	147042774	552	ytoplasmic antibody-asso	Xq27.3	147961254	FMR1 - FMR1NB	rs5904818	ıtergenic_variaı	2.00E-08	New
13	54102212	20	G	A	54102206	783	Body mass index	13q14.3	53528071	.INC01065 - LOC10537021	rs12429545	ıtergenic_variaı	2.00E-08	Non_identical
21	36738246	18	G	A	36738242	3168	Rheumatoid arthritis	21q22.12	35365944	RUNX1 - LOC100506403	rs8133843	intron_variant	2.00E-08	Non_identical
3	81792106	20	C	A	81792112	2109	Body mass index	3p12.2	81742961	GBE1	rs3849570	intron_variant	2.00E-08	Non_identical
5	134366194	16	A	G	134366200	2802	Testicular germ cell tumor	5q31.1	135030510	PITX1	rs3805663	intron_variant	2.00E-08	Non_identical
7	89977751	26	A	G	89977760	2055	Homocysteine levels	7q21.13	90348446	GTPBP10	rs42648	intron_variant	2.00E-08	Smaller
11	43864275	22	T	C	43864278	3594	Body mass index	11p11.2	43842728	HSD17B12	rs2176598	intron_variant	3.00E-08	Perfect_to_near
11	43864275	22	T	C	43864278	3594	Body mass index	11p11.2	43842728	HSD17B12	rs2176598	intron_variant	3.00E-08	Perfect_to_near
14	23977010	0	A	G	23977010	2608	Resting heart rate	14q11.2	23507801	NGDN - THTPA	rs223116	intron_variant	3.00E-08	New
15	99258705	12	A	T	99258710	2415	Fasting plasma glucose	15q26.3	98715481	IGF1R	rs2018860	intron_variant	3.00E-08	Perfect_to_near
16	30918485	18	C	G	30918487	1782	Triglycerides	16p11.2	30907166	CTF1 - FBXL19-AS1	rs11649653	intron_variant	3.00E-08	Near_to_perfect
16	60314656	16	G	T	60314656	501	hospholipid levels (plasma	16q21	60280752	OC101927580 - LOC72915	rs9932186	ıtergenic_variaı	3.00E-08	Perfect_to_near
16	68591231	8	A	C	68591230	4080	Ulcerative colitis	16q22.1	68557327	ZFP90	rs1728785	intron_variant	3.00E-08	Perfect_to_near
17	64779437	22	G	T	64779430	3000	Height	17q24.2	66783312	PRKCA	rs3889237	intron_variant	3.00E-08	Non_identical
17	77273509	16	G	A	77273513	490	rate levels (BMI interactio	17q25.3	79277431	RBFOX3	rs898534	intron_variant	3.00E-08	Near_to_perfect
17	80898090	0	T	C	80898088	4666	n in asthma (oral corticost	17q25.3	82940212	TBCD	rs9896933	intron_variant	3.00E-08	New
18	57622282	14	T	C	57622287	3319	ronic lymphocytic leukem	18q21.32	59955055	.OC105372151 - NFE2L3P1	rs4368253	atory_region_v	3.00E-08	Near_to_perfect
1	218698025	12	C	T	218698027	1339	histogram emphysema pa	1q41	218524685	TGFB2 - LOC105372924	rs1690789	intron_variant	3.00E-08	Near_to_perfect
20	1941167	16	G	A,C	1941171	922,779	Aortic root size	20p13	1960525	LOC727993	rs6045676	intron_variant	3.00E-08	Near_to_perfect
2	165528871	16	C	T	165528876	1986	Waist-hip ratio	2q24.3	164672366	LOC101929615 - COBLL1	rs13389219	intron_variant	3.00E-08	Near_to_perfect
2	232988576	0	A	C	232988582	2862	Height	2q37.1	232123872	DIS3L2	rs3103267	intron_variant	3.00E-08	New
2	234818868	0	G	A	234818869	1180	sure measurement (cold p	2q37.1	233910224	MSL3P1 - TRPM8	rs7577262	ıtergenic_variaı	3.00E-08	New
2	237058144	0	T	A,C	237058144	20,868	Educational attainment	2q37.2	236149500	AGAP1 - LOC105373944	rs11687170	stream_gene_v	3.00E-08	New
2	237105519	0	G	A	237105518	1116	Educational attainment	2q37.2	236196875	ASB18	rs13401104	intron_variant	3.00E-08	New
3	41828302	18	A	G	41828300	415	'eloma and monoclonal ga	3p22.1	41786808	ULK4	rs73071352	intron_variant	3.00E-08	Perfect_to_near
3	72934372	16	T	C	72934371	1545	Motion sickness	3p13	72885220	GXYLT2	rs1847202	ream_gene_va	3.00E-08	Near_to_perfect
5	58676044	22	A	G	58676049	840	Sleep-related phenotypes	5q11.2	59380223	PDE4D	rs1823068	intron_variant	3.00E-08	Perfect_to_near
5	153680745	36	A	T	153680747	1388	Schizophrenia	5q33.2	154301187	GALNT10	rs11740474	intron_variant	3.00E-08	Perfect_to_near
6	50836281	8	G	A	50836279	877	Obesity (extreme)	6p12.3	50868566	RPS17P5 - FTH1P5	rs734597	ıtergenic_variaı	3.00E-08	Near_to_perfect
6	162161618	16	C	T	162161619	3689	Disc degeneration (lumbar	6q26	161740587	PARK2	rs926849	intron_variant	3.00E-08	Near_to_perfect
8	89760308	8	A	G	89760311	1589	lisorder, bipolar disorder, r	8q21.3	88748082	C105375629 - LOC105375	rs7004633	ıtergenic_variaı	3.00E-08	Perfect_to_near
9	116131694	20	A	G	116131695	930	Lead levels in blood	9q32	113369415	BSPRY	rs10121150	intron_variant	3.00E-08	Perfect_to_near
9	136154866	18	G	T	136154867	803	ensin-converting enzyme a	9q34.2	133279294	ABO - SURF6	rs495828	ream_gene_va	3.00E-08	Near_to_perfect
15	72161411	22	T	C	72161403	50	Height	15q23	71869062	MYO9A	rs12902421	intron_variant	3.00E-08	Smaller
2	174212886	20	G	A	174212894	4151	Breast cancer	2q31.1	173348166	RPS2P18 - CDCA7	rs1550623	intron_variant	3.00E-08	Smaller
3	81792106	20	C	A	81792112	2109	Body mass index	3p12.2	81742961	GBE1	rs3849570	intron_variant	3.00E-08	Non_identical
6	32409534	22	G	A	32409530	2545	Parkinson's disease	6p21.32	32441753	HLA-DRA	rs3129882	intron_variant	3.00E-08	Non_identical
9	28414346	18	A	G	28414339	1020	Obesity	9p21.1	28414341	LINGO2	rs10968576	intron_variant	3.00E-08	Smaller
11	14774588	18	A	G	14774591	1751	Vitamin D levels	11p15.2	14753045	PDE3B	rs1007392	intron_variant	4.00E-08	Near_to_perfect
11	79077192	8	A	G	79077193	523	Bipolar disorder	11q14.1	79366149	TENM4	rs12576775	intron_variant	4.00E-08	Near_to_perfect
11	79077192	8	A	G	79077193	523	lisorder, bipolar disorder, r	11q14.1	79366149	TENM4	rs12576775	intron_variant	4.00E-08	Near_to_perfect
12	14413931	12	G	C	14413931	1679	Breast cancer	12p13.1	14260997	GRIN2B - ATF7IP	rs12422552	atory_region_v	4.00E-08	Perfect_to_near
12	29917261	26	C	A	29917265	940	Schizophrenia	12p11.22	29764332	TMTC1	rs679087	intron_variant	4.00E-08	Absent
12	48419614	18	A	C	48419618	191	Prostate cancer	12q13.11	48025835	LOC105369750	rs80130819	ream_gene_va	4.00E-08	Perfect_to_near
12	57645787	8	T	A	57645789	657	levels in overweight indiv	12q13.3	57252006	STAC3 - R3HDM2	rs11172134	intron_variant	4.00E-08	Near_to_perfect
14	24771285	14	G	A	24771285	753	effect on language impair	14q12	24302079	NOP9	rs4280164	nissense_variaı	4.00E-08	Perfect_to_near
15	28356858	8	C	T	28356859	886	Vitiligo	15q13.1	28111713	HERC2	rs1129038	rime_UTR_var	4.00E-08	Perfect_to_near
16	24675590	16	T	C	24675589	115	ner's disease (cognitive d	16p12.1	24664268	LINC01567	rs8045064	intron_variant	4.00E-08	Near_to_perfect
16	68591231	8	A	C	68591230	4080	Ulcerative colitis	16q22.1	68557327	ZFP90	rs1728785	intron_variant	4.00E-08	Perfect_to_near
18	57849022	16	G	A	57849023	1206	HDL cholesterol	18q21.32	60181790	C105372154 - LOC105372	rs12967135	ıtergenic_variaı	4.00E-08	Perfect_to_near
1	207977082	12	A	G	207977083	1269	Schizophrenia	1q32.2	207803738	C1orf132	rs7523273	g_transcript_ex	4.00E-08	Near_to_perfect
22	50435489	20	A	G	50435480	3040	Ulcerative colitis	22q13.33	49997051	IL17REL	rs5771069	nissense_variaı	4.00E-08	Near_to_perfect
2	166978752	8	C	A	166978750	1062	l lobe epilepsy with hippoc	2q24.3	166122240	SCN1A, LOC101929680	rs7587026	intron_variant	4.00E-08	Near_to_perfect
2	174326852	18	C	T	174326845	544	rement (high sodium and p	2q31.1	173462117	OC105373744 - LOC64399	rs10930597	ıtergenic_variaı	4.00E-08	Smaller
2	174326852	18	C	T	174326845	544	e measurement (low sodiu	2q31.1	173462117	OC105373744 - LOC64399	rs10930597	ıtergenic_variaı	4.00E-08	Smaller
2	237805680	0	T	G	237805687	731	n in asthma (oral corticost	2q37.3	236897044	C105373949 - LOC105373	rs7599706	ıtergenic_variaı	4.00E-08	New
4	89713119	12	C	T	89713121	3041	ip ratio adjusted for body r	4q22.1	88791970	FAM13A	rs9991328	intron_variant	4.00E-08	Perfect_to_near
5	44365545	20	C	A	44365545	2336	Prostate cancer	5p12	44365443	FGF10	rs2121875	intron_variant	4.00E-08	Near_to_perfect
5	158604961	16	G	A	158604963	2444	Mean platelet volume	5q33.3	159177955	RNF145	rs10076782	intron_variant	4.00E-08	Perfect_to_near
7	142274852	16	G	A	142274854	2796	Narcolepsy	7q34	142357119	TRBV7-2 - TRBV8-1	rs2854536	intron_variant	4.00E-08	Near_to_perfect
8	26206073	10	C	T	26206077	845	Height	8p21.2	26348561	PPP2R2A	rs1594829	intron_variant	4.00E-08	Near_to_perfect
1	230307176	18	T	C	230307182	3177	Metabolic syndrome	1q42.13	230171436	GALNT2	rs4846922	intron_variant	4.00E-08	Non_identical
7	37374517	18	G	A	37374510	429	Primary biliary cirrhosis	7p14.1	37334906	ELMO1	rs6974491	intron_variant	4.00E-08	Smaller
10	114758351	14	C	T	114758349	1141	Type 2 diabetes	10q25.2	112998590	TCF7L2	rs7903146	intron_variant	5.00E-08	Near_to_perfect
12	21368718	24	T	C	21368722	1076	Bilirubin levels	12p12.1	21215788	SLCO1B1	rs4363657	intron_variant	5.00E-08	Near_to_perfect
13	32972625	8	A	T	32972626	22	Breast cancer	13q13.1	32398489	BRCA2	rs11571833	stop_gained	5.00E-08	Near_to_perfect
13	110818600	0	T	G	110818598	1444	Arterial stiffness	13q34	110166251	COL4A1	rs3742207	nissense_variaı	5.00E-08	New
2	75926566	18	T	C	75926565	1275	Hippocampal atrophy	2p12	75699439	GCFC2	rs2298948	intron_variant	5.00E-08	Perfect_to_near
2	234679388	0	C	T	234679384	1043	LDL cholesterol	2q37.1	233770738	JGT1A7, UGT1A5, UGT1A9	rs11563251	rime_UTR_var	5.00E-08	New
3	18721736	18	T	C	18721736	69	lychlorinated biphenyl leve	3p24.3	18680244	C101927805 - LOC105376	rs76942353	intron_variant	5.00E-08	Near_to_perfect
6	32381736	24	T	A	32381736	3250	ip ratio adjusted for body r	6p21.32	32413959	BTNL2 - HLA-DRA	rs7759742	atory_region_v	5.00E-08	Near_to_perfect
7	26397234	20	C	A	26397239	3188	ip ratio adjusted for body r	7p15.2	26357619	SNX10	rs1534696	intron_variant	5.00E-08	Near_to_perfect
8	85079708	20	T	C	85079709	3858	Body mass index	8q21.2	84167474	LOC105375932 - RALYL	rs2033732	ream_gene_va	5.00E-08	Near_to_perfect

8	118184780	8	C	T	118184783	1278	Type 2 diabetes	8q24.11	117172544	LOC105375716, SLC30A8	rs13266634	nissense_variai	5.00E-08	Near_to_perfect
8	118184780	8	C	T	118184783	1278	3lycated hemoglobin level	8q24.11	117172544	LOC105375716, SLC30A8	rs13266634	nissense_variai	5.00E-08	Near_to_perfect
8	118184780	8	C	T	118184783	1278	Type 2 diabetes	8q24.11	117172544	LOC105375716, SLC30A8	rs13266634	nissense_variai	5.00E-08	Near_to_perfect
8	118184780	8	C	T	118184783	1278	Type 2 diabetes	8q24.11	117172544	LOC105375716, SLC30A8	rs13266634	nissense_variai	5.00E-08	Near_to_perfect
9	139265593	18	G	A	139265596	6	ocyte chemoattractant prot	9q34.3	136371144	CARD9	rs34971035	ce_region_vari	5.00E-08	Perfect_to_near
20	48955428	16	T	C	48955424	3030	nflammatory bowel diseas	20q13.13	50338887	.INC01271 - LOC10537265	rs913678	atory_region_v	5.00E-08	Non_identical
4	118334626	20	T	C	118334619	241	odosum in inflammatory bo	4q26	117413463	.OC105377390 - LINC0137	rs6828740	g_transcript_ex	5.00E-08	Non_identical
4	154864754	24	T	C	154864750	3084	\ methylation (parent-of-or	4q31.3	153943598	SFRP2 - LOC101927947	rs13135284	ntergenic_variai	5.00E-08	Smaller
5	172939420	24	C	T	172939426	1310	Prostate cancer	5q35.2	173512423	LOC105377732	rs6869841	ntergenic_variai	5.00E-08	Smaller
6	117114034	22	G	A	117114025	1607	C-reactive protein levels	6q22.1	116792862	GPRC6A	rs6901250	ıonymous_vari	5.00E-08	Smaller
11	41915368	10	C	A	41915366	670	Type 2 diabetes	11p12	41893816	C105376639 - LOC101928	rs9300039	ntergenic_variai	6.00E-08	Near_to_perfect
14	79899450	16	C	T	79899454	2817	Body mass index	14q31.1	79433111	NRXN3	rs7141420	intron_variant	6.00E-08	Perfect_to_near
16	792185	14	A	G	792190	2698	Height	16p13.3	742190	NARFL - MSLN	rs11648796	intron_variant	6.00E-08	Perfect_to_near
18	43317279	18	T	C	43317282	3555	Bladder cancer	18q12.3	45737317	LOC105372093, SLC14A1	rs10775480	intron_variant	6.00E-08	Near_to_perfect
1	222164944	14	A	G	222164948	954	ogressive supranuclear pa	1q41	221991606	C105372950 - LOC105372	rs6687758	atory_region_v	6.00E-08	Near_to_perfect
2	201580952	20	C	T	201580954	947	Intelligence	2q33.1	200717470	)X2P, AOX2P; LOC100507	563911, rs7589	g_transcript_ex	6.00E-08	Near_to_perfect
2	201582191	12	T	C	201582193	4143	Intelligence	2q33.1	200717470	)X2P, AOX2P; LOC100507	563911, rs7589	g_transcript_ex	6.00E-08	Near_to_perfect
3	87134800	8	A	G	87134800	379	Prostate cancer	3p12.1	87085650	LOC285232 - LINC00506	rs17023900	ream_gene_va	6.00E-08	Near_to_perfect
4	187174684	18	A	G	187174683	2750	Plasma renin activity level:	4q35.2	186253529	KLKB1	rs4253311	intron_variant	6.00E-08	Perfect_to_near
7	107207692	20	A	G	107207695	527	Osteoarthritis	7q22.3	107567250	DUS4L	rs4730250	intron_variant	6.00E-08	Perfect_to_near
8	89760308	8	A	G	89760311	1589	Schizophrenia	8q21.3	88748082	C105375629 - LOC105375	rs7004633	ntergenic_variai	6.00E-08	Perfect_to_near
8	118184780	8	C	T	118184783	1278	Type 2 diabetes	8q24.11	117172544	LOC105375716, SLC30A8	rs13266634	nissense_variai	6.00E-08	Near_to_perfect
1	56850678	18	C	A	56850686	804	erebrospinal AB1-42 leve	1p32.2	56385014	C105378743 - LOC105378	rs11206801	ntergenic_variai	6.00E-08	Non_identical
8	24044293	18	C	T	24044301	442	Migraine - clinic-based	8p21.2	24186788	LOC105379328 - ADAM28	rs11777116	ntergenic_variai	6.00E-08	Bigger
10	16997264	8	G	T	16997266	973	Colorectal cancer	10p13	16955267	CUBN	rs10904849	intron_variant	7.00E-08	Near_to_perfect
16	49062588	10	C	A	49062590	2695	Body mass index	16q12.1	49028679	C105371240 - LOC105371	rs2080454	ntergenic_variai	7.00E-08	Near_to_perfect
17	69125607	10	G	T	69125606	1453	lmonary function (interacti	17q24.3	71129465	CASC17	rs11654749	intron_variant	7.00E-08	Near_to_perfect
1	53871087	22	G	C	53871085	46	on in chronic obstructive p	1p32.3	53405413	C105378729 - LOC102724	rs114216682	ntergenic_variai	7.00E-08	Near_to_perfect
4	89064579	8	A	C	89064581	2481	Dental caries	4q22.1	88143429	ABCG2	rs3114018	intron_variant	7.00E-08	Perfect_to_near
5	133179081	16	C	T	133179080	1082	rophic lateral sclerosis (sp	5q31.1	133843389	FSTL4 - LOC105379182	rs2457174	ntergenic_variai	7.00E-08	Near_to_perfect
16	88321442	16	C	T	88321436	3916	Axial length	16q24.2	88287830	ZNF469	rs6540223	intron_variant	7.00E-08	Smaller
22	32049910	20	T	C	32049917	464	Intelligence	22q12.2	31653931	PISD	61746, rs1262	intron_variant	7.00E-08	Non_identical
11	31905535	18	C	T	31905534	1756	Body mass index	11p13	31883988	DKFZp686K1684	rs652722	intron_variant	8.00E-08	Perfect_to_near
15	79235445	14	T	C	79235446	1843	Type 1 diabetes	15q25.1	78943104	CTSH	rs3825932	intron_variant	8.00E-08	Near_to_perfect
1	34186192	16	C	T	34186193	881	Intelligence	1p35.1	33723829	CSMD2	7533254, rs52	intron_variant	8.00E-08	Near_to_perfect
2	233426528	0	C	T	233426526	1636	small cell lung cancer (sur	2q37.1	232561816	EIF4E2	rs1656402	intron_variant	8.00E-08	New
2	234406652	0	G	T	234406655	494	Bilirubin levels	2q37.1	233498009	USP40	rs6704644	intron_variant	8.00E-08	New
2	236795336	0	A	C	236795343	2176	Schizophrenia	2q37.2	235886699	AGAP1	rs13025591	intron_variant	8.00E-08	New
4	113855479	18	C	G	113855477	1137	al exhaled nitric oxide (ch	4q25	112934321	ANK2	rs12500579	intron_variant	8.00E-08	Perfect_to_near
8	118184780	8	C	T	118184783	1278	e 2 diabetes and other tra	8q24.11	117172544	LOC105375716, SLC30A8	rs13266634	nissense_variai	8.00E-08	Near_to_perfect
15	96708284	20	T	C	96708291	1565	hormone-binding globulin l	15q26.2	96165062	NR2F2-AS1	rs8023580	intron_variant	8.00E-08	Bigger
1	34180845	14	G	T	34180842	398	Intelligence	1p35.1	33723829	CSMD2	7533254, rs52	intron_variant	8.00E-08	Non_identical
2	161572788	18	G	A	161572781	2302	Educational attainment	2q24.2	160716270	C105373718 - LOC105373	rs6732189	ntergenic_variai	8.00E-08	Smaller
10	44753866	0	T	C	44753867	1666	Coronary heart disease	10q11.21	44258419	.INC00841 - LOC10192946	rs501120	stream_gene_v	9.00E-08	New
16	85975657	8	C	T	85975659	3961	hronic lymphocytic leukem	16q24.1	85942053	LOC105371388	rs305061	ntergenic_variai	9.00E-08	Near_to_perfect
18	64699971	18	C	T	64699974	218	il in Tripanosoma cruzi ser	18q22.1	67032737	CDH19 - MIR5011	rs73963343	ntergenic_variai	9.00E-08	Near_to_perfect
3	150467810	14	G	T	150467808	1438	Breast cancer	3q25.1	150750021	SIAH2	rs6788895	intron_variant	9.00E-08	Near_to_perfect
4	80946476	8	T	A	80946475	3298	Ankylosing spondylitis	4q21.21	80025321	ANTXR2	rs4389526	intron_variant	9.00E-08	Perfect_to_near
4	131133486	18	C	T	131133491	4261	Intelligence	4q28.3	130212336	C105377419 - LOC105377	099040, rs131	ntergenic_variai	9.00E-08	Near_to_perfect
6	51068721	10	C	T	51068717	1393	/ mass index in non-asthm	6p12.3	51101004	FTH1P5 - LOC105375085	rs7775861	ntergenic_variai	9.00E-08	Bigger
7	94006987	20	C	T	94006994	631	Intelligence	7q21.3	94379223	LOC101927525	465411, rs1322	ntergenic_variai	9.00E-08	Near_to_perfect
13	54102212	20	G	A	54102206	783	Body mass index	13q14.3	53528071	.INC01065 - LOC10537021	rs12429545	ntergenic_variai	9.00E-08	Non_identical
10	14945406	26	A	G	14945406	274	Migraine	10p13	14903407	SUV39H2	rs11594111	rime_UTR_var	1.00E-07	Near_to_perfect
10	114758351	14	C	T	114758349	1141	3lycated hemoglobin level	10q25.2	112998590	TCF7L2	rs7903146	intron_variant	1.00E-07	Near_to_perfect
11	2204295	22	G	T	2204288	2466	Type 2 diabetes	11p15.5	2183058	MIR4686 - ASCL2	rs11043007	ntergenic_variai	1.00E-07	Non_identical
11	2936951	18	G	A	2936952	365	Bilirubin levels	11p15.4	2915722	SLC22A18	rs16928809	intron_variant	1.00E-07	Near_to_perfect
11	9111554	14	A	G	9111558	4092	coprotein I (β2-GPI) p	11p15.4	9090011	SCUBE2	rs963167	intron_variant	1.00E-07	Near_to_perfect
11	11504225	18	G	A	11504228	204	to tocilizumab in rheumato	11p15.4	11482681	GALNT18	rs7940423	intron_variant	1.00E-07	Near_to_perfect
11	61616014	22	A	G	61616012	746	Trans fatty acid levels	11q12.2	61848540	FADS2	rs2851682	intron_variant	1.00E-07	Near_to_perfect
11	112968651	14	A	C	112968651	2089	Body mass index	11q23.2	113097929	NCAM1	rs1816537	intron_variant	1.00E-07	Perfect_to_near
16	82326815	24	G	A	82326805	338	Obesity-related traits	16q23.3	82293200	C105371365 - LOC100419	rs11863065	ntergenic_variai	1.00E-07	Smaller
18	29336590	18	A	G	29336591	328	Coronary artery calcificatio	18q12.1	31756628	LOC105372052	rs10502575	stream_gene_v	1.00E-07	Perfect_to_near
18	62418441	8	A	C	62418444	2028	ar degeneration (smoking	18q22.1	64751208	C105372167 - LOC101927	rs17073641	ntergenic_variai	1.00E-07	Near_to_perfect
19	9959010	12	G	A	9959014	1557	Sleep duration	19p13.2	9848338	PIN1	rs2287838	rime_UTR_var	1.00E-07	Perfect_to_near
1	114377568	0	A	G	114377568	4871	Type 1 diabetes	1p13.2	113834946	PTPN22, AP4B1-AS1	rs2476601	nissense_variai	1.00E-07	New
1	114377568	0	A	G	114377568	4871	Vitiligo	1p13.2	113834946	PTPN22, AP4B1-AS1	rs2476601	nissense_variai	1.00E-07	New
1	216716535	12	A	G	216716537	1339	Cardiac hypertrophy	1q41	216543195	ESRRG	rs12757165	intron_variant	1.00E-07	Near_to_perfect
2	158977795	16	A	G	158977794	1004	rebrospinal P-tau181p lev	2q24.1	158121282	UPP2	rs2074955	intron_variant	1.00E-07	Near_to_perfect
2	175377564	22	C	T	175377565	1399	rate levels (BMI interactio	2q31.1	174512837	LOC105373748 - WIPF1	rs12693043	ntergenic_variai	1.00E-07	Near_to_perfect
2	234565282	0	A	C	234565283	487	Bladder cancer	2q37.1	233656637	UGT1A8, UGT1A10	rs11892031	intron_variant	1.00E-07	New
2	234565282	0	A	C	234565283	487	Bladder cancer	2q37.1	233656637	UGT1A8, UGT1A10	rs11892031	intron_variant	1.00E-07	New
2	234827661	0	G	A	234827661	281	Migraine with aura	2q37.1	233919016	TRPM8	rs6741751	intron_variant	1.00E-07	New
3	2895691	20	T	C	2895684	3842	Intelligence	3p26.2	2854000	ITN4; LOC105376926, CNT	584516, rs4629	intron_variant	1.00E-07	Non_identical
3	39513278	8	C	T	39513278	631	mer's disease (cognitive d	3p22.1	39471787	MOBP	rs538867	intron_variant	1.00E-07	Near_to_perfect
3	122730912	16	G	A	122730910	1640	Esophageal cancer	3q21.1	123012063	SEMA5B	rs9868873	intron_variant	1.00E-07	Near_to_perfect
4	106578752	14	A	G	106578754	269	1 (forced expiratory volume	4q24	105657597	ARHGEF38	rs1982346	intron_variant	1.00E-07	Perfect_to_near
4	118035557	8	C	A	118035559	95	lychlorinated biphenyl leve	4q26	117114403	C105377388 - LOC105377	rs114452217	ntergenic_variai	1.00E-07	Perfect_to_near
5	21749343	16	T	C	21749348	358	to tocilizumab in rheumato	5p14.3	21749239	LOC105374684	rs12109285	intron_variant	1.00E-07	Near_to_perfect
5	32804533	16	T	C	32804528	3011	Blood pressure	5p13.3	32804422	NPR3 - LOC340113	rs1173766	ntergenic_variai	1.00E-07	Non_identical
6	31434113	22	A	G	31434111	695	e seizures (MMR vaccine-r	6p21.33	31466334	HCP5 - HCG26	rs3094604	intron_variant	1.00E-07	Perfect_to_near

6	32860750	18	G	A	32860752	671	Disc degeneration (lumbar	6p21.32	32892975	PPP1R2P1 - LOC10029414	rs9469300	ream_gene_va	1.00E-07	Near_to_perfect
6	87155253	18	A	T	87155254	347	Sexual dysfunction (female	6q14.3	86445536	RPL7P27 - LOC643962	rs13202860	itergenic_variai	1.00E-07	Near_to_perfect
6	157195975	16	C	G	157195980	2426	Sitting height ratio	6q25.3	156874846	ARID1B	rs2063714	intron_variant	1.00E-07	Near_to_perfect
7	34093999	0	A	G	34093997	964	Alzheimer's disease	7p14.3	34054385	BMPER	rs17169634	intron_variant	1.00E-07	New
7	125869117	16	G	T	125869122	1291	Hearing function	7q31.33	126229068	C105375487 - LOC10537	rs2687481	itergenic_variai	1.00E-07	Near_to_perfect
8	6821624	0	T	C	6821617	2572	IgA nephropathy	8p23.1	7041476	DEFA9P - DEFA10P, DEFA	rs2738058, rs	ream_gene_va	1.00E-07	New
8	95856912	8	A	G	95856911	743	Type 2 diabetes	8q22.1	94844683	INTS8	rs17359493	intron_variant	1.00E-07	Perfect_to_near
8	95856912	8	A	G	95856911	743	Type 2 diabetes	8q22.1	94844683	INTS8	rs17359493	intron_variant	1.00E-07	Perfect_to_near
8	124659324	12	C	A	124659329	3944	Electrocardiographic traits	8q24.13	123647089	KLHL38	rs11991744	intron_variant	1.00E-07	Perfect_to_near
11	124821581	16	T	A	124821575	4009	a in Tripanosoma cruzi se	11q24.2	124951679	LOC105369547	rs4408325	ream_gene_va	1.00E-07	Smaller
12	24389663	16	G	T	24389660	4195	onse to antipsychotic trea	12p12.1	24236726	LOC101928471, SOX5	rs1464500	intron_variant	1.00E-07	Non_identical
17	45425125	22	G	A	45425115	2058	Cholesterol, total	17q21.32	47347749	EFCAB13	rs7206971	intron_variant	1.00E-07	Non_identical
2	22568706	24	A	T	22568713	1155	ve pulmonary disease (mo	2p24.1	22345841	C105374321 - LOC10537	rs7569716	intron_variant	1.00E-07	Smaller
3	87241503	22	C	T	87241497	1706	Prostate cancer	3p11.2	87192347	LINC00506 - MIR4795	rs7629490	itergenic_variai	1.00E-07	Non_identical
7	46392573	14	T	G	46392567	134	nent response for severe	7p12.3	46352969	LOC105375265	rs17513961	itergenic_variai	1.00E-07	Bigger
8	66624237	16	A	G	66624232	226	IgG glycosylation	8q13.1	65711997	MTFR1	rs10504390	intron_variant	1.00E-07	Non_identical
10	16299952	0	C	T	16299951	153	Obesity	10p13	16257952	FAM188A - LOC102724039	rs10508503	itergenic_variai	2.00E-07	New
10	49985108	16	C	T	49985110	173	pressant treatment in majo	10q11.23	48777065	WDFY4	rs10857636	intron_variant	2.00E-07	Near_to_perfect
10	71588499	16	G	A	71588504	1938	ic fatty liver disease histol	10q22.1	69828748	COL13A1	rs1227756	intron_variant	2.00E-07	Near_to_perfect
10	80925580	26	G	C	80925577	1701	Sitting height ratio	10q22.3	79165820	ZMIZ1	rs7916441	intron_variant	2.00E-07	Non_identical
10	126649512	0	A	G	126649516	861	Height	10q26.13	124960947	ZRANB1	rs17152411	intron_variant	2.00E-07	New
11	98125406	0	A	G	98125404	2054	lar disorder and schizophr	11q22.1	98254676	C105369454 - LOC10536	rs2509843	itergenic_variai	2.00E-07	New
11	102742758	24	G	T	102742761	311	histogram emphysema pa	11q22.2	102872031	MMP12	rs17368659	intron_variant	2.00E-07	Perfect_to_near
11	112968651	14	A	C	112968651	2089	Body mass index	11q23.2	113097929	NCAM1	rs1816537	intron_variant	2.00E-07	Perfect_to_near
12	57645787	8	T	A	57645789	657	levels in overweight indiv	12q13.3	57252006	STAC3 - R3HDM2	rs11172134	intron_variant	2.00E-07	Near_to_perfect
12	91474419	22	C	T	91474422	704	pressure (alcohol consump	12q21.33	91080645	KERA - LUM	rs991427	itergenic_variai	2.00E-07	Near_to_perfect
12	105452437	24	G	A	105452442	706	IgG glycosylation	12q23.3	105058664	ALDH1L2	rs10861337	intron_variant	2.00E-07	Near_to_perfect
12	106949983	10	G	A	106949987	2304	Parkinson's disease	12q23.3	106556209	LOC100287944	rs4964469	intron_variant	2.00E-07	Near_to_perfect
14	69034679	8	C	T	69034682	425	Breast cancer	14q24.1	68567965	RAD51B	rs999737	intron_variant	2.00E-07	Perfect_to_near
14	81635323	8	C	T	81635323	414	IgG glycosylation	14q31.1	81168979	C101928462, LOC105370	rs10131728	intron_variant	2.00E-07	Perfect_to_near
14	90227248	8	C	G	90227251	3032	IgG glycosylation	14q32.11	89760907	CHORDC2P - EFCAB11	rs2093746	intron_variant	2.00E-07	Perfect_to_near
15	30193469	14	T	C	30193468	3200	onse to antipsychotic trea	15q13.1	29901265	TJP1	rs711355	intron_variant	2.00E-07	Perfect_to_near
15	38220390	22	T	A	38220391	3623	levels in overweight indiv	15q14	37928190	LOC105370773 - TMCO5A	rs4620914	intron_variant	2.00E-07	Perfect_to_near
16	20342570	0	T	A	20342572	725	Type 1 diabetes	16p12.3	20331250	GP2 - UMOD	rs12444268	ream_gene_va	2.00E-07	New
16	30918485	18	C	G	30918487	1782	Triglycerides	16p11.2	30907166	CTF1 - FBXL19-AS1	rs11649653	intron_variant	2.00E-07	Near_to_perfect
18	4773112	14	C	T	4773110	2394	White blood cell types	18p11.31	4773111	LOC105371968 - PPIAP14	rs7237848	stream_gene_v	2.00E-07	Near_to_perfect
18	75110357	24	T	A	75110360	2616	lychlorinated biphenyl leve	18q23	77398404	GALR1 - LINC01029	rs10469074	itergenic_variai	2.00E-07	Perfect_to_near
1	10694902	14	C	T	10694907	358	hol dependence (age at or	1p36.22	10634850	PEX14 - CASZ1	rs61776290	ream_gene_va	2.00E-07	Perfect_to_near
1	38994119	14	G	A	38994115	484	rophic lateral sclerosis (sp	1p34.3	38528443	LOC105378658	rs11590421	itergenic_variai	2.00E-07	Bigger
1	59690604	12	G	A	59690602	734	Cervical artery dissection	1p32.1	59224930	C105378754 - LOC10537	rs12402265	intron_variant	2.00E-07	Perfect_to_near
1	61831937	22	A	G	61831938	568	Bipolar disorder	1p31.3	61366266	NFIA	25777, rs1256	intron_variant	2.00E-07	Near_to_perfect
1	61831937	22	A	G	61831938	568	Bipolar disorder	1p31.3	61366266	NFIA	3, rs1125777, r	intron_variant	2.00E-07	Near_to_perfect
1	67685598	28	A	C	67685598	1963	Crohn's disease	1p31.3	67264945	41, IL23R, IL23R - LOC10	65804, rs1120	stream_gene_v	2.00E-07	Perfect_to_near
1	67730634	0	G	T	67730628	2171	Crohn's disease	1p31.3	67264945	41, IL23R, IL23R - LOC10	65804, rs1120	stream_gene_v	2.00E-07	New
1	200935864	22	T	C	200935866	769	Ulcerative colitis	1q32.1	200966738	MROH3P - KIF21B	rs11584383	stream_gene_v	2.00E-07	Near_to_perfect
20	48632602	18	T	C	48632610	1754	Psoriasis	20q13.13	50016073	SNAI1 - LOC105372654	rs7352944	atory_region_v	2.00E-07	New
21	47165425	0	A	G	47165429	4790	onse to smallpox (secrete	21q22.3	45745515	C105372840 - LOC10012	rs8127571	intron_variant	2.00E-07	New
22	37544483	8	G	A	37544486	714	Tuberculosis	22q12.3	37148446	IL2RB	rs3218255	intron_variant	2.00E-07	Near_to_perfect
22	50435489	20	A	G	50435480	3040	Ulcerative colitis	22q13.33	49997051	IL17REL	rs5771069	nissense_variai	2.00E-07	Near_to_perfect
2	3476412	22	C	A	3476422	1663	Neurofibrillary tangles	2p25.3	3472651	TRAPPC12	rs11675119	intron_variant	2.00E-07	Non_identical
2	21231589	18	G	A	21231592	72	ciated phospholipase A2 a	2p24.1	21008720	APOB	rs6413458	nonymous_vari	2.00E-07	Near_to_perfect
2	22736986	16	C	T	22736987	650	Self-rated health	2p24.1	22514115	LOC105374322	rs17043947	intron_variant	2.00E-07	Near_to_perfect
2	60720753	18	G	A	60720757	2405	F-cell distribution	2p16.1	60498316	BCL11A	0172646, rs46	intron_variant	2.00E-07	Perfect_to_near
2	60722039	8	C	T	60722040	1211	F-cell distribution	2p16.1	60498316	BCL11A	0172646, rs46	intron_variant	2.00E-07	Perfect_to_near
2	207244781	16	A	G	207244783	1092	Body mass index	2q33.3	206380059	ZDBF2 - HNRNPA1P51	rs972540	atory_region_v	2.00E-07	Perfect_to_near
3	18721736	18	T	C	18721736	69	lychlorinated biphenyl leve	3p24.3	18680244	C101927805 - LOC10537	rs76942353	intron_variant	2.00E-07	Near_to_perfect
3	35982129	20	C	T	35982130	299	Cognitive performance	3p22.3	35940638	LOC105377028 - STAC	rs6799705	itergenic_variai	2.00E-07	Perfect_to_near
3	43467893	12	T	C	43467895	577	Obesity-related traits	3p22.1	43426403	ANO10	rs7650267	intron_variant	2.00E-07	Near_to_perfect
5	28340173	8	G	A	28340173	1344	pressant treatment in majo	5p14.1	28340066	C105374698 - LOC10537	rs11949289	itergenic_variai	2.00E-07	Perfect_to_near
5	126978108	16	T	C	126978108	1684	id beta peptide concentra	5q23.2	127642416	PRRC1 - CTXN3	rs11241936	intron_variant	2.00E-07	Near_to_perfect
5	131723287	18	T	C	131723288	1785	Asthma	5q31.1	132387596	SLC22A5	rs2073643	intron_variant	2.00E-07	Near_to_perfect
6	21330295	0	G	T	21330293	2272	Prostate cancer	6p22.3	21330062	CDKAL1 - LINC00581	rs12198220	itergenic_variai	2.00E-07	New
6	32381736	24	T	A	32381736	3250	ip ratio adjusted for body r	6p21.32	32413959	BTNL2 - HLA-DRA	rs7759742	atory_region_v	2.00E-07	Near_to_perfect
6	32572249	20	T	C	32572251	564	lar sclerosis Hodgkin lymp	6p21.32	32604474	163 - HLA-DRA; BTNL2 - H	rs9268542, rs	intron_variant	2.00E-07	Near_to_perfect
6	55950372	0	G	A	55950374	695	Psychosis (atypical)	6p12.1	56085576	COL21A1	rs12196860	intron_variant	2.00E-07	New
6	128021800	22	A	C	128021803	218	response to smallpox vacc	6q22.33	127700658	LOC105377997 - THEMIS	rs17299841	itergenic_variai	2.00E-07	Perfect_to_near
7	22756463	18	C	T	22756463	1034	Bilirubin levels	7p15.3	22716844	LOC401312 - IL6	rs10155981	atory_region_v	2.00E-07	Near_to_perfect
8	81031819	8	A	G	81031822	3564	abolite levels (Pyroglutam	8q21.13	80119587	TPD52	rs10957961	intron_variant	2.00E-07	Perfect_to_near
8	85079708	20	T	C	85079709	3858	Body mass index	8q21.2	84167474	LOC105375932 - RALYL	rs2033732	ream_gene_va	2.00E-07	Near_to_perfect
8	89760308	8	A	G	89760311	1589	Schizophrenia	8q21.3	88748082	C105375629 - LOC10537	rs7004633	itergenic_variai	2.00E-07	Perfect_to_near
8	116813902	16	G	A	116813905	2361	to tocilizumab in rheumat	8q23.3	115801679	TRPS1 - LINC00536	rs800586	intron_variant	2.00E-07	Perfect_to_near
8	118184780	8	C	T	118184783	1278	Glycated hemoglobin level	8q24.11	117172544	LOC105375716, SLC30A8	rs13266634	nissense_variai	2.00E-07	Near_to_perfect
9	117049886	16	T	G	117049891	3791	Height	9q32	114287611	COL27A1	rs946053	intron_variant	2.00E-07	Near_to_perfect
13	44217069	20	T	C	44217064	2641	to tocilizumab in rheumat	13q14.11	43642928	ENOX1	rs4942242	intron_variant	2.00E-07	Smaller
17	47402819	32	C	T	47402807	926	Blood pressure	17q21.33	49325445	ZNF652	rs12940887	intron_variant	2.00E-07	Smaller
1	67722571	14	C	A	67722567	1965	Crohn's disease	1p31.3	67264945	41, IL23R, IL23R - LOC10	65804, rs1120	stream_gene_v	2.00E-07	Non_identical
3	30710236	20	A	G	30710243	931	Tonometry	3p24.1	30668751	TGFBR2	rs3773643	intron_variant	2.00E-07	Non_identical
3	81792106	20	C	A	81792112	2109	Body mass index	3p12.2	81742961	GBE1	rs3849570	intron_variant	2.00E-07	Non_identical
5	52179784	22	T	C	52179790	1131	lmonary function (interacti	5q11.2	52883958	ITGA1	rs2456203	intron_variant	2.00E-07	Smaller
6	31632139	20	C	A	31632134	668	seizures (MMR vaccine-r	6p21.33	31664357	GPANK1	rs3130618	nissense_variai	2.00E-07	Non_identical

6	148787168	20	C	T	148787159	323	Rotator cuff tears	6q24.3	148466023	SASH1	rs12527089	intron_variant	2.00E-07	Bigger
7	37374517	18	G	A	37374510	429	Celiac disease	7p14.1	37334906	ELMO1	rs6974491	intron_variant	2.00E-07	Smaller
8	56168051	18	C	T	56168059	149	ponse to smallpox (secrete	8q12.1	55255499	XKR4	rs12542677	intron_variant	2.00E-07	Non_identical
8	66624237	16	A	G	66624232	226	IgG glycosylation	8q13.1	65711997	MTFR1	rs10504390	intron_variant	2.00E-07	Non_identical
10	114874014	22	C	T	114874019	3928	sorder (body mass index ir	10q25.3	113114260	TCF7L2	rs290475	intron_variant	3.00E-07	Perfect_to_near
11	51394927	14	C	T	51394932	406	Body mass index	11q11	54724348	OR4A5 - TRIM48	rs1391576	ream_gene_va	3.00E-07	Near_to_perfect
12	4405385	12	A	G	4405389	1931	Colorectal cancer	12p13.32	4296223	CCND2	rs3217901	intron_variant	3.00E-07	Perfect_to_near
12	56070675	20	C	T	56070668	1381	Response to amphetamine	12q13.2	55676884	OR6C70 - METTL7B	rs55874825	ream_gene_va	3.00E-07	New
12	65718295	20	T	G	65718299	135	ocortical brain region volur	12q14.3	65324519	MSRB3	rs17178006	intron_variant	3.00E-07	Perfect_to_near
13	42951446	8	C	T	42951449	1421	Bone mineral density	13q14.11	42377313	AKAP11 - LOC105370177	rs9533090	ntergenic_variar	3.00E-07	Near_to_perfect
13	43143027	20	C	T	43143029	1643	density (paediatric, total bo	13q14.11	42568893	TNFSF11	rs17536328	intron_variant	3.00E-07	Perfect_to_near
13	64950424	8	T	C	64950422	2030	Rheumatoid arthritis	13q21.31	64376290	C105377815 - LOC105370?	rs1340319	ntergenic_variar	3.00E-07	Near_to_perfect
13	79410575	16	T	C	79410574	116	duct disorder (symptom co	13q31.1	78836439	LINC00331	rs11838918	ntergenic_variar	3.00E-07	Near_to_perfect
14	90227248	8	C	G	90227251	3032	IgG glycosylation	14q32.11	89760907	CHORDC2P - EFCAB11	rs2093746	intron_variant	3.00E-07	Perfect_to_near
15	31708262	10	C	T	31708263	982	ip ratio adjusted for body r	15q13.3	31416060	KLF13	rs8042543	intron_variant	3.00E-07	Perfect_to_near
15	55386742	18	A	G	55386743	381	Inflammatory skin disease	15q21.3	55094545	LOC105370829 - RSL24D1	rs1528473	stream_gene_v	3.00E-07	Near_to_perfect
17	16246016	0	A	C	16246016	1150	myotrophic lateral scleros	17p11.2	16342702	PIGL, CENPV	rs7477	rime_UTR_var	3.00E-07	New
17	16246016	0	A	C	16246016	1150	myotrophic lateral scleros	17p11.2	16342702	PIGL, CENPV	rs7477	rime_UTR_var	3.00E-07	New
17	34358298	0	T	C	34358297	1858	Pulmonary function	17q12	36031260	CCL23 - LOC105371746	rs712046	ntergenic_variar	3.00E-07	New
18	10078069	18	G	A	10078071	990	lmonary function (interacti	18p11.22	10078074	C105371983 - LOC105371?	rs8089099	ntergenic_variar	3.00E-07	Near_to_perfect
18	35351724	22	C	T	35351726	1870	Educational attainment	18q12.2	37771762	OC105372073, LOC105372C	rs1187220	ntergenic_variar	3.00E-07	Near_to_perfect
18	60080113	22	G	A	60080112	1221	onse to antipsychotic trea	18q21.33	62412879	TNFRSF11A - RPL17P44	rs2980976	ream_gene_va	3.00E-07	Near_to_perfect
1	49589845	20	A	G	49589847	2270	Body mass index	1p33	49124175	LOC105378706, AGBL4	rs657452	intron_variant	3.00E-07	Near_to_perfect
1	67600100	18	C	T	67600101	4211	l in Tripanosoma cruzi ser	1p31.3	67134418	C1orf141	rs12069782	intron_variant	3.00E-07	Near_to_perfect
1	156827701	12	A	G	156827703	9	lychlorinated biphenyl leve	1q23.1	156857911	NTRK1, INSRR	rs115699453	intron_variant	3.00E-07	Perfect_to_near
1	237598416	8	C	T	237598416	1096	IgG glycosylation	1q43	237435116	RYR2	rs7529251	intron_variant	3.00E-07	Near_to_perfect
2	40396087	20	C	T	40396078	2496	HIV-associated dementia	2p22.1	40168938	SLC8A1, SLC8A1-AS1	rs404005	intron_variant	3.00E-07	Perfect_to_near
4	74956381	0	A	T	74956372	3146	White blood cell types	4q13.3	74090655	PPBPP2 - CXCL2	rs546829	ream_gene_va	3.00E-07	New
5	6635593	18	G	C	6635591	2473	Response to amphetamine	5p15.31	6635478	SRD5A1	rs472402	intron_variant	3.00E-07	Absent
6	32205110	0	T	C	32205110	2240	Crohn's disease	6p21.32	32237333	NOTCH4 - LOC101929163	rs9267911	ntergenic_variar	3.00E-07	New
6	164186674	14	A	G	164186677	1726	nse to TNF antagonist trea	6q26	163765645	C105378104 - LOC105378?	rs10945919	ntergenic_variar	3.00E-07	Perfect_to_near
7	42325625	14	G	T	42325625	282	Inflammatory biomarkers	7p14.1	42286026	GLI3 - LOC105375249	rs12532960	atory_region_v	3.00E-07	Near_to_perfect
9	106793721	22	A	G	106793726	511	Orofacial clefts (interaction	9q31.1	104031445	.OC101928523 - SMC2-AS1	rs1536895	ntergenic_variar	3.00E-07	Perfect_to_near
9	119926422	20	T	A	119926426	4543	onse to antipsychotic trea	9q33.1	117164147	ASTN2	rs4838255	intron_variant	3.00E-07	Near_to_perfect
12	69762991	20	A	G	69762987	246	esponse to diuretic therap	12q15	69430244	LYZ, YEATS4, YEATS4 - L(	, rs315135, rs	ntergenic_variar	3.00E-07	Bigger
13	26923571	20	T	G	26923565	410	onse to measles-mumps-ru	13q12.13	26349428	CDK8	rs3736995	intron_variant	3.00E-07	Non_identical
16	52321982	18	T	C	52321976	88	macokinetics (acute lymph	16q12.1	52288064	CASC22 - LOC105371262	rs16951021	ntergenic_variar	3.00E-07	Non_identical
17	45425125	22	G	A	45425115	2058	LDL cholesterol	17q21.32	47347749	EFCAB13	rs7206971	intron_variant	3.00E-07	Non_identical
1	220977337	22	T	G	220977333	1370	early outgrowth colony for	1q41	220803991	1-Mar	rs6693017	intron_variant	3.00E-07	Non_identical
2	51667730	20	C	A	51667739	229	Cognitive performance	2p16.3	51440601	LOC730100	rs1206397	intron_variant	3.00E-07	Non_identical
2	220668731	16	C	T	220668738	2737	nic cleft lip with or without	2q35	219804017	C105373888 - LOC101928	rs3815854	ntergenic_variar	3.00E-07	Non_identical
3	10877620	22	C	T	10877611	2695	to tocilizumab in rheumato	3p25.3	10835926	SLC6A11	rs1809529	intron_variant	3.00E-07	Smaller
4	128523969	24	C	T	128523964	4936	Trans fatty acid levels	4q28.1	127602809	LOC105377412	rs1399212	ntergenic_variar	3.00E-07	Smaller
6	31802458	18	C	G	31802465	616	IgG glycosylation	6p21.33	31834688	HSPA1B - C6orf48	rs4711279	rime_UTR_var	3.00E-07	Non_identical
6	91207356	20	A	G	91207351	2060	cit hyperactivity disorder (	6q15	90497632	LOC105377891 - MAP3K7	rs806276	atory_region_v	3.00E-07	Non_identical
11	43864275	22	T	C	43864278	3594	Body mass index	11p11.2	43842728	HSD17B12	rs2176598	intron_variant	4.00E-07	Perfect_to_near
13	92501662	34	C	T	92501664	606	Obesity-related traits	13q31.3	91849410	GPC5	rs7328464	intron_variant	4.00E-07	Near_to_perfect
16	82326815	24	G	A	82326805	338	Obesity-related traits	16q23.3	82293200	C105371365 - LOC100419	rs11863065	ntergenic_variar	4.00E-07	Smaller
16	82646330	14	A	G	82646333	2999	emotherapy in breast canc	16q23.3	82612728	LOC101928392 - CDH13	rs3844412	ntergenic_variar	4.00E-07	Perfect_to_near
18	10078069	18	G	A	10078071	990	lmonary function (interacti	18p11.22	10078074	C105371983 - LOC105371?	rs8089099	ntergenic_variar	4.00E-07	Near_to_perfect
19	10213169	32	G	A	10213154	210	Protein quantitative trait loc	19p13.2	10102478	ANGPTL6	rs8109578	intron_variant	4.00E-07	Perfect_to_near
1	57126918	18	C	T	57126919	1120	Lymphocyte counts	1p32.2	56661246	PRKAA2	rs2746347	intron_variant	4.00E-07	Perfect_to_near
20	16311309	8	T	C	16311310	4046	Ileal carcinoids	20p12.1	16330665	KIF16B	rs2208059	intron_variant	4.00E-07	Near_to_perfect
21	44779690	22	C	T	44779680	4065	Metabolic syndrome	21q22.3	43359800	LOC101928399	rs496300	intron_variant	4.00E-07	Perfect_to_near
2	77223937	22	C	T	77223938	2793	IgG glycosylation	2p12	76996812	LOC101927907, LRRTM4	rs1470506	intron_variant	4.00E-07	Near_to_perfect
2	111599704	20	G	A	111599706	2376	IgA nephropathy	2q13	110842129	ACOXL	rs4849121	intron_variant	4.00E-07	Perfect_to_near
2	207244781	16	A	G	207244783	1092	Body mass index	2q33.3	206380059	ZDBF2 - HNRNPA1P51	rs972540	atory_region_v	4.00E-07	Perfect_to_near
5	43382859	0	T	C	43382858	2153	Metabolite levels (X-11787	5p12	43382756	CCL28	rs11951515	intron_variant	4.00E-07	New
5	67194467	14	C	T	67194469	936	Menarche (age at onset)	5q13.1	67898641	C101928858 - LOC105379	rs10940138	intron_variant	4.00E-07	Near_to_perfect
5	127382299	24	C	T	127382302	98	mer's disease (cognitive d	5q23.3	128046610	LINC01184	rs146579248	intron_variant	4.00E-07	Near_to_perfect
7	106495807	8	T	C	106495809	570	Menopause (age at onset)	7q22.3	106855364	CCDC71L - PIK3CG	rs17153527	ntergenic_variar	4.00E-07	Perfect_to_near
7	150510914	8	G	A	150510915	3236	olume in 1 second (enviro	7q36.1	150813827	TMEM176A - LOC10537556	rs2888674	ntergenic_variar	4.00E-07	Near_to_perfect
9	1721474	12	T	C	1721478	2344	Educational attainment	9p24.3	1721478	C102723803 - LOC105375	rs1478110	ntergenic_variar	4.00E-07	Perfect_to_near
12	88837238	14	T	G	88837244	143	Trans fatty acid levels	12q21.32	88443467	LOC105369884 - KITLG	rs11104877	ntergenic_variar	4.00E-07	Bigger
18	49940979	14	A	G	49940973	373	Eosinophilic esophagitis	18q21.2	52414603	DCC	rs9956738	intron_variant	4.00E-07	Bigger
1	116740457	24	C	A	116740450	699	Word reading	1p13.1	116197828	MAB21L3 - LOC105378919	rs4839516	ntergenic_variar	4.00E-07	Smaller
7	130754816	16	G	C	130754812	2823	taneous malignant melanc	7q32.3	131070053	LINC-PINT	rs4731742	intron_variant	4.00E-07	Non_identical
10	53493468	22	G	A	53493473	573	Asthma	10q21.1	51733713	PRKG1, LOC105378304	rs7922491	intron_variant	5.00E-07	Near_to_perfect
10	64872404	20	C	T	64872411	1398	IgG glycosylation	10q21.3	63112651	EGR2 - LOC105378328	rs10822136	stream_gene_v	5.00E-07	Absent
11	39031790	8	T	G	39031793	3649	ree thyroxine concentratio	11p12	39010243	C101928563 - LOC105376	rs7951105	ntergenic_variar	5.00E-07	Perfect_to_near
12	20521651	8	C	A	20521654	2187	Male infertility	12p12.2	20368720	PDE3A	rs10841496	intron_variant	5.00E-07	Perfect_to_near
13	74920183	8	C	A	74920186	821	Glucose homeostasis traits	13q22.1	74346049	LOC101927121	rs10492494	ntergenic_variar	5.00E-07	Near_to_perfect
14	96122412	22	A	G	96122408	1318	QT interval	14q32.13	95656071	TCL6	rs8015016	intron_variant	5.00E-07	Non_identical
16	7504848	0	G	A	7504854	1718	Metabolite levels	16p13.3	7454852	RBFOX1	rs9924951	intron_variant	5.00E-07	New
16	24898969	24	C	A	24898972	3711	ritis in systemic lupus ery	16p12.1	24887651	SLC5A11	rs274068	intron_variant	5.00E-07	Near_to_perfect
19	47661491	16	G	A	47661493	455	Multiple sclerosis	19q13.32	47158236	SAE1	rs307896	intron_variant	5.00E-07	Near_to_perfect
20	51557158	16	G	A	51557161	1173	y mass index in non-asthm	20q13.2	52940622	LOC105372668 - TSHZ2	rs6097169	ntergenic_variar	5.00E-07	Near_to_perfect
20	53267121	22	C	G	53267111	615	Functional MRI	20q13.2	54650572	DOK5	rs2023454	rime_UTR_var	5.00E-07	Near_to_perfect
2	174326852	18	C	T	174326845	544	e measurement (low sodiu	2q31.1	173462117	OC105373744 - LOC64399	rs10930597	ntergenic_variar	5.00E-07	Smaller
2	234502117	0	T	C	234502121	384	HIV-1 control	2q37.1	233593475	USP40 - UGT1A8	rs13394720	ntergenic_variar	5.00E-07	New

2	235210725	22	G	A	235210727	834	eoporosis-related phenoty	2q37.1	234302083	C105373933 - LOC105373	rs12151790	atory_region_v	5.00E-07	Perfect_to_near
3	41828302	18	A	G	41828300	415	Multiple myeloma	3p22.1	41786808	ULK4	rs73071352	intron_variant	5.00E-07	Perfect_to_near
3	45731448	10	C	T	45731451	1999	ressant treatment in majo	3p21.31	45689959	SACM1L	rs2742417	rime_UTR_var	5.00E-07	Near_to_perfect
4	5237152	22	G	A	5237153	284	mer's disease (cognitive d	4p16.2	5235426	STK32B	rs78647349	intron_variant	5.00E-07	Near_to_perfect
5	158424389	8	G	A	158424391	2934	py (neutropenia/leucopeni	5q33.3	158997383	EBF1	rs10040979	intron_variant	5.00E-07	Perfect_to_near
6	32856480	8	T	C	32856482	738	Disc degeneration (lumbar	6p21.32	32888705	PPP1R2P1 - LOC10029414	rs1029295	itergenic_varia	5.00E-07	Perfect_to_near
6	91845407	22	T	G	91845407	4057	rophic lateral sclerosis (sp	6q15	91135689	LOC105377893 - MIR4643	rs4424056	itergenic_varia	5.00E-07	Perfect_to_near
6	97500037	0	G	C	97500047	1274	Blood pressure	6q16.1	97052171	KLHL32	rs12195230	intron_variant	5.00E-07	New
6	124743506	16	A	G	124743507	4077	Celiac disease	6q22.31	124422361	NKAIN2	rs531930	intron_variant	5.00E-07	Near_to_perfect
7	85048375	34	G	A	85048380	33	lychlorinated biphenyl leve	7q21.11	85419064	SEMA3D - LOC105375380	rs142857449	ream_gene_va	5.00E-07	Near_to_perfect
7	113741102	8	T	G	113741105	1787	IgG glycosylation	7q31.1	114101050	FOXP2	rs6466479	intron_variant	5.00E-07	Perfect_to_near
8	29082282	16	G	A	29082285	113	l in Tripanosoma cruzi ser	8p12	29224768	KIF13B	rs75609241	intron_variant	5.00E-07	Perfect_to_near
8	118184780	8	C	T	118184783	1278	Type 2 diabetes	8q24.11	117172544	LOC105375716, SLC30A8	rs13266634	nissense_varia	5.00E-07	Near_to_perfect
9	92036419	0	A	C	92036427	1941	Orofacial clefts	9q22.2	89421512	SEMA4D	rs4132699	intron_variant	5.00E-07	New
9	100591458	18	C	T	100591463	3285	Thyroid hormone levels	9q22.33	97829181	PTCSC2	rs7045138	itergenic_varia	5.00E-07	Near_to_perfect
9	119314216	8	A	G	119314217	3005	lmonary function (interacti	9q33.1	116551938	ASTN2-AS1, ASTN2	rs13290997	intron_variant	5.00E-07	Perfect_to_near
10	73849630	20	G	A	73849639	1482	Insulin-like growth factors	10q22.1	72089881	SPOCK2 - ASCC1	rs1245541	ream_gene_va	5.00E-07	Non_identical
2	24168196	30	G	A	24168188	2576	Sitting height ratio	2p23.3	23945318	UBXN2A	rs60490158	intron_variant	5.00E-07	Smaller
2	31474235	20	G	C	31474242	1255	Estradiol levels	2p23.1	31251376	EHD3	rs597800	intron_variant	5.00E-07	Smaller
2	47240006	18	G	T	47240012	428	unction and prostate canc	2p21	47012873	TTC7A	rs10194115	intron_variant	5.00E-07	Non_identical
2	176991784	22	A	G	176991779	3510	Magnesium levels	2q31.1	176127051	LOC100129455	rs2592394	intron_variant	5.00E-07	Non_identical
6	30666675	22	C	G	30666669	317	IgG glycosylation	6p21.33	30698892	RPL7P4 - MDC1	rs9468811	ream_gene_va	5.00E-07	Non_identical
6	39183464	18	C	T	39183470	908	Migraine	6p21.2	39215694	KCNK5	rs10456100	intron_variant	5.00E-07	Non_identical
X	14950525	18	A	G	14950531	1985	hip circumference ratio (in	Xp22.2	14932409	MOSPD2 - LOC102724092	rs5980075	itergenic_varia	5.00E-07	Non_identical
12	119989644	12	A	G	119989646	884	Aging traits	12q24.23	119551841	LOC105370027	rs7137869	intron_variant	6.00E-07	Perfect_to_near
14	68243075	8	C	A	68243075	3492	rophic lateral sclerosis (sp	14q24.1	67776358	ZFYVE26	rs12891047	intron_variant	6.00E-07	Near_to_perfect
17	54278713	8	G	A	54278715	1688	Cannabis dependence	17q22	56201354	ANKFN1	rs1019238	intron_variant	6.00E-07	Perfect_to_near
2	17775031	18	A	G	17775032	649	chosis and Alzheimer's dis	2p24.2	17593765	VSNL1	rs4038131	intron_variant	6.00E-07	Perfect_to_near
2	240444221	18	C	A	240444219	151	l pressure (smoking intera	2q37.3	239522525	OC101928111 - LOC40104	rs11679072	itergenic_varia	6.00E-07	Perfect_to_near
2	242502948	24	C	T	242502956	3502	Brain structure	2q37.3	241563541	BOK	rs12479254	intron_variant	6.00E-07	Smaller
3	18721736	18	T	C	18721736	69	lychlorinated biphenyl leve	3p24.3	18680244	C101927805 - LOC105376	rs76942353	intron_variant	6.00E-07	Near_to_perfect
4	82126122	14	C	G	82126121	75	l pressure (smoking intera	4q21.21	81204967	PRKG2	rs17484474	nonymous_vari	6.00E-07	Near_to_perfect
5	142095951	18	A	T	142095952	1234	Trans fatty acid levels	5q31.3	142716387	FGF1 - LOC101926975	rs6890562	itergenic_varia	6.00E-07	Near_to_perfect
6	41154650	8	C	T	41154650	1526	heimer's disease (late ons	6p21.1	41186912	LOC105375056 - TREML2	rs9381040	stream_gene_v	6.00E-07	Near_to_perfect
12	111155538	18	T	C	111155531	643	Heart rate	12q24.11	110717726	PPP1CC	rs11065706	stream_gene_v	6.00E-07	Smaller
13	34641568	22	G	A	34641562	177	Vitamin D levels	13q13.2	34067425	C105370156 - LOC105370	rs12868495	itergenic_varia	6.00E-07	Non_identical
3	170163478	22	G	A	170163474	4281	lar disorder and schizophr	3q26.2	170445686	LOC105374214	rs6444931	intron_variant	6.00E-07	Non_identical
4	169409963	20	T	C	169409958	4235	olic fatty liver disease histo	4q32.3	168488807	DDX60L - PALLD	rs2710833	intron_variant	6.00E-07	Non_identical
6	10906262	26	A	G	10906256	982	Allergic rhinitis	6p24.2	10906023	SYCP2L	rs4713039	nonymous_vari	6.00E-07	Non_identical
6	30666675	22	C	G	30666669	317	IgG glycosylation	6p21.33	30698892	RPL7P4 - MDC1	rs9468811	ream_gene_va	6.00E-07	Non_identical
7	93691749	16	G	A	93691744	2565	Aging	7q21.3	94062432	BET1 - LOC101927525	rs9918668	intron_variant	6.00E-07	Non_identical
10	12051812	16	G	A	12051816	1667	e cancer (gene x gene inte	10p14	12009817	UPF2	rs10795917	intron_variant	7.00E-07	Near_to_perfect
10	64872404	20	C	T	64872411	1398	IgG glycosylation	10q21.3	63112651	EGR2 - LOC105378328	rs10822136	stream_gene_v	7.00E-07	Absent
10	114758351	14	C	T	114758349	1141	Metabolic syndrome	10q25.2	112998590	TCF7L2	rs7903146	intron_variant	7.00E-07	Near_to_perfect
10	129274502	24	T	C	129274503	2353	myotrophic lateral scleros	10q26.2	127476239	DOCK1 - NPS	rs4363506	itergenic_varia	7.00E-07	Perfect_to_near
13	36476444	8	A	G	36476447	1961	Height	13q13.3	35902310	LOC105370163, DCLK1	rs6563210	intron_variant	7.00E-07	Perfect_to_near
15	33126453	8	A	G	33126454	581	ler cancer (smoking intera	15q13.3	32834253	FMN1	rs1258767	intron_variant	7.00E-07	Perfect_to_near
15	57910159	16	G	A	57910164	2685	HDL cholesterol	15q21.3	57617966	MYZAP, GCOM1	rs937254	intron_variant	7.00E-07	Near_to_perfect
15	61060706	14	A	G	61060707	1065	ubcutaneous adipose tissu	15q22.2	60768508	RORA	rs12591650	intron_variant	7.00E-07	Near_to_perfect
15	99072902	8	G	A	99072905	1069	rthritis (juvenile idiopathic	15q26.3	98529676	LOC105371012	rs12719740	itergenic_varia	7.00E-07	Near_to_perfect
16	11691751	18	T	C	11691753	2528	QT interval	16p13.13	11597897	LITAF	rs8049607	intron_variant	7.00E-07	Perfect_to_near
18	38765657	18	C	T	38765659	415	Obesity	18q12.3	41185695	C105372082 - LOC105372	rs17697518	itergenic_varia	7.00E-07	Perfect_to_near
1	159818513	18	C	T	159818513	1840	ultiple sclerosis (OCB statu	1q23.2	159848723	LOC105373478, C1orf204	rs6659742	intron_variant	7.00E-07	Near_to_perfect
20	11157012	8	A	T	11157011	3201	ponse to citalopram treatm	20p12.2	11176363	LOC105372528	rs6040399	itergenic_varia	7.00E-07	Near_to_perfect
20	16536423	24	C	T	16536414	974	se to taxane treatment (do	20p12.1	16555769	KIF16B	rs6044112	intron_variant	7.00E-07	Perfect_to_near
20	20634105	18	T	C	20634106	383	IgG glycosylation	20p11.23	20653462	RALGAPA2	rs6132333	intron_variant	7.00E-07	Perfect_to_near
2	232298075	0	G	A	232298076	2342	lisorders (purging via subs	2q37.1	231433365	OC105373927 - ZBTB8OSF	rs12475512	stream_gene_v	7.00E-07	New
2	234818868	0	G	A	234818869	1180	sure measurement (cold p	2q37.1	233910224	MSL3P1 - TRPM8	rs7577262	itergenic_varia	7.00E-07	New
3	131574483	14	G	A	131574485	800	Body mass index	3q22.1	131855641	LOC105374113, CPNE4	rs9856151	intron_variant	7.00E-07	Near_to_perfect
3	194327097	10	T	C	194327098	4765	QT interval	3q29	194606369	TMEM44	rs789852	intron_variant	7.00E-07	Near_to_perfect
4	129890789	8	T	C	129890790	1431	ve pulmonary disease (mo	4q28.2	128969635	SCLT1	rs7655841	intron_variant	7.00E-07	Near_to_perfect
4	150672512	18	G	A	150672514	394	Survival in rectal cancer	4q31.23	149751362	LOC285423	rs17026425	intron_variant	7.00E-07	Perfect_to_near
4	190538062	0	A	C,G	190538063	7,421,753	Periodontitis (CDC/AAP)	4q35.2	189616909	OC105377615 - LINC0126	rs13145041	itergenic_varia	7.00E-07	New
6	80257277	18	C	A	80257281	1031	Hoarding	6q14.1	79547564	LOC105377866	rs3747767	itergenic_varia	7.00E-07	Perfect_to_near
6	148704952	18	G	A	148704954	1631	ype 2 diabetes nephropath	6q24.3	148383818	SASH1	rs6930576	intron_variant	7.00E-07	Near_to_perfect
7	2795953	16	A	G	2795957	1052	Height	7p22.3	2756323	GNA12, AMZ1	rs798497	intron_variant	7.00E-07	Near_to_perfect
9	21997014	14	T	A	21997015	781	hageal squamous cell carc	9p21.3	21997016	CDKN2B-AS1	rs61271866	intron_variant	7.00E-07	Perfect_to_near
9	79408142	12	T	C	79408144	1744	Hippocampal atrophy	9q21.2	76793228	PRUNE2	rs10781380	intron_variant	7.00E-07	Perfect_to_near
9	91540059	14	G	A	91540059	1824	ponse to statin therapy (LD	9q22.1	88925144	MIR4289 - C9orf47	rs1875620	itergenic_varia	7.00E-07	Near_to_perfect
9	113300833	20	C	T	113300835	2806	Pulmonary function decline	9q31.3	110538555	SVEP1	rs1889321	intron_variant	7.00E-07	Near_to_perfect
2	113529243	16	T	C	113529240	1089	otic treatment in schizophr	2q14.1	112771663	CKAP2L - IL1A	rs11677416	stream_gene_v	7.00E-07	Non_identical
3	1301138	22	C	A	1301130	1438	l in Tripanosoma cruzi ser	3p26.3	1259446	CNTN6	rs9815195	intron_variant	7.00E-07	Non_identical
3	105010894	12	A	G	105010890	1552	le dysfunction in type 1 dia	3q13.11	105292046	LOC105374023 - ALCAM	rs9810233	itergenic_varia	7.00E-07	Smaller
11	128492736	8	T	C	128492739	2282	Rheumatoid arthritis	11q24.3	128622844	C105369568 - LOC101929	rs4937362	ream_gene_va	8.00E-07	Perfect_to_near
13	70430081	0	C	T	70430093	603	Response to amphetamine	13q21.33	69855961	KLHL1	rs2325244	intron_variant	8.00E-07	New
15	60781514	16	T	C	60781513	1275	Menarche (age at onset)	15q22.2	60489314	RORA, RORA-AS1	rs3743266	rime_UTR_var	8.00E-07	Near_to_perfect
15	63312631	22	G	A	63312632	2525	Orofacial clefts	15q22.2	63020433	LOC100287243 - TPM1	rs1873147	atory_region_v	8.00E-07	Perfect_to_near
16	20031203	14	T	A	20031205	790	IgG glycosylation	16p12.3	20019883	LOC105371117 - GPR139	rs2764743	itergenic_varia	8.00E-07	Near_to_perfect
16	84446380	0	C	G	84446384	1932	tion deficit hyperactivity dis	16q24.1	84412778	ATP2C2	rs10514604	intron_variant	8.00E-07	New
19	6887725	0	G	A	6887736	642	Periodontitis	19p13.3	6887725	ADGRE1	rs3826782	intron_variant	8.00E-07	New

2	25276285	14	G	A	25276284	630	Height	2p23.3	25053415	EFR3B	rs6733301	intron_variant	8.00E-07	Perfect_to_near
2	123224399	8	C	T	123224400	1472	Food antigen IgG levels	2q14.3	122466824	C105373592 - LOC105373	rs4848780	itergenic_varia	8.00E-07	Perfect_to_near
2	177037316	18	A	G	177037311	4099	lucinous ovarian carcinom	2q31.1	176172583	HOXD3	rs711830	rime_UTR_var	8.00E-07	Non_identical
4	138582707	12	G	A	138582711	1046	Obesity-related traits	4q28.3	137661557	LOC101927414	rs17049741	itergenic_varia	8.00E-07	Perfect_to_near
5	32278232	16	A	G	32278233	150	reuptake inhibitors in maj	5p13.3	32278127	MTMR12	rs73069924	intron_variant	8.00E-07	Perfect_to_near
7	34697059	12	G	T	34697060	1255	IgG glycosylation	7p14.3	34657448	NPSR1-AS1	rs2530544	intron_variant	8.00E-07	Perfect_to_near
8	62055666	10	G	T	62055667	1502	rophic lateral sclerosis (sp	8q12.2	61143108	CLVS1	rs7830371	intron_variant	8.00E-07	Near_to_perfect
9	22770882	18	A	G	22770886	1343	sychotic therapy (extrapyra	9p21.3	22770887	LINC01239	rs10811771	intron_variant	8.00E-07	Perfect_to_near
9	74887701	16	A	C	74887703	1175	Suicidal ideation	9q21.13	72272787	GDA - LOC100289320	rs11143230	ream_gene_va	8.00E-07	Near_to_perfect
20	33909775	22	C	T	33909784	960	Height	20q11.22	35321981	UQCC1	rs6088792	intron_variant	8.00E-07	Non_identical
3	60995603	20	G	C	60995600	617	Blood pressure	3p14.2	61009928	FHIT	rs6782531	intron_variant	8.00E-07	Non_identical
6	34582267	22	T	G	34582274	182	on in Tripanosoma cruzi se	6p21.31	34614497	C6orf106	rs182503338	intron_variant	8.00E-07	Smaller
8	3088165	18	T	C	3088173	428	ner's disease (cognitive d	8p23.2	3230651	CSMD1	rs73660619	intron_variant	8.00E-07	Non_identical
9	122199208	22	A	T	122199203	2775	Height	9q33.1	119436925	LOC105376250	rs1331623	itergenic_varia	8.00E-07	Smaller
16	82326815	24	G	A	82326805	338	Obesity-related traits	16q23.3	82293200	C105371365 - LOC100419	rs11863065	itergenic_varia	9.00E-07	Smaller
17	54192520	8	A	G	54192523	3472	Cannabis dependence	17q22	56115162	ANKFN1	rs1431318	intron_variant	9.00E-07	Perfect_to_near
17	72975746	8	C	A	72975748	39	ier's disease in APOE e4-	17q25.1	74979653	HID1-AS1 - CDR2L	rs71380849	stream_gene_v	9.00E-07	Perfect_to_near
19	49228268	24	T	C	49228272	1082	nin B levels in ischemic st	19q13.33	48725015	RASIP1	rs2287921	g_transcript_ex	9.00E-07	Perfect_to_near
3	2895691	20	T	C	2895684	3842	Intelligence	3p26.2	2854000	CNTN4	rs11713158	intron_variant	9.00E-07	Non_identical
4	72028550	0	A	G	72028545	1192	al exhaled nitric oxide (ch	4q13.3	71162828	DCK - LOC102724821	rs7685921	itergenic_varia	9.00E-07	Near_to_perfect
4	72028550	0	A	G	72028545	1192	al exhaled nitric oxide (ch	4q13.3	71162828	DCK - LOC102724821	rs7685921	itergenic_varia	9.00E-07	Near_to_perfect
6	98019078	18	A	G	98019080	1226	ultiple sclerosis (OCB statu	6q16.1	97571204	LOC101927314	rs9320598	intron_variant	9.00E-07	Near_to_perfect
9	16385135	12	C	A	16385132	3091	evels (Dihydroxy docosat	9p22.3	16385134	C9orf92 - BNC2	rs9406636	itergenic_varia	9.00E-07	Non_identical
X	109939209	18	T	G	109939205	1470	Coronary heart disease	Xq23	110695977	CHRDL1	rs5943057	intron_variant	9.00E-07	Non_identical
10	52010715	16	C	T	52010708	822	verse metabolic effects in	10q11.23	50250948	ASAH2	rs10508921	ream_gene_va	9.00E-07	Bigger
13	94952840	20	T	C	94952832	606	Disc degeneration (lumbar	13q31.3	94300578	GPC6	rs9301951	intron_variant	9.00E-07	Smaller
15	55950086	14	C	A	55950082	814	emotherapy (neutropenia/l	15q21.3	55657884	PRTG	rs11071200	intron_variant	9.00E-07	Bigger
16	79406924	14	C	T	79406918	403	Type 2 diabetes	16q23.2	79373021	MAF	rs17797882	atory_region_v	9.00E-07	Smaller
3	85531205	24	A	T	85531199	597	ongevity (90 years and olde	3p12.1	85482049	CADM2	rs9841144	intron_variant	9.00E-07	Bigger
3	187413995	12	G	A	187413991	4842	ponse to citalopram treatm	3q27.3	187696203	SST - RTP2	rs6764050	stream_gene_v	9.00E-07	Smaller
8	80556494	18	G	A	80556500	210	reuptake inhibitors in maj	8q21.13	79644265	STMN2	rs113889867	intron_variant	9.00E-07	Non_identical
10	12438785	18	G	A	12438783	1638	Obesity-related traits	10p13	12396784	CAMK1D	rs10906142	intron_variant	1.00E-06	Near_to_perfect
10	12438785	18	G	A	12438783	1638	Obesity-related traits	10p13	12396784	CAMK1D	rs10906142	intron_variant	1.00E-06	Near_to_perfect
10	17130695	14	A	G	17130693	1154	olate pathway vitamin leve	10p13	17088694	CUBN	rs11254363	intron_variant	1.00E-06	Near_to_perfect
10	52568611	20	T	C	52568616	3929	ate levels in lean individu	10q11.23	50808856	A1CF	rs4256922	intron_variant	1.00E-06	Perfect_to_near
11	14098481	8	G	T	14098482	266	Inflammatory biomarkers	11p15.2	14076935	SPON1	rs17556665	intron_variant	1.00E-06	Near_to_perfect
11	26741261	22	C	T	26741262	327	iisocyanate-induced asthm	11p14.2	26719715	SLC5A12	rs7112383	intron_variant	1.00E-06	Perfect_to_near
11	42315890	0	A	G	42315886	4470	esponse to smallpox vacc	11p12	42294336	OC105376642 - HNRNPKP	rs7394570	itergenic_varia	1.00E-06	New
12	43253413	10	T	G	43253415	4783	Heart failure	12q12	42859612	C105369739 - LOC105369	rs1520832	itergenic_varia	1.00E-06	Perfect_to_near
12	96027757	8	A	G	96027759	1324	Breast size	12q22	95633983	USP44 - PGAM1P5	rs17356907	intron_variant	1.00E-06	Perfect_to_near
13	29368707	14	C	T	29368706	2130	Obesity-related traits	13q12.3	28794569	LOC105370138 - MTUS2	rs954108	intron_variant	1.00E-06	Near_to_perfect
14	90758889	0	G	T	90758891	736	Longevity	14q32.11	90292547	NRDE2	rs2282032	intron_variant	1.00E-06	New
15	31708262	10	C	T	31708263	982	ip ratio adjusted for body r	15q13.3	31416060	KLF13	rs8042543	intron_variant	1.00E-06	Perfect_to_near
15	33050423	16	C	T	33050423	2302	Orofacial clefts	15q13.3	32758222	GREM1 - FMN1	rs1258763	itergenic_varia	1.00E-06	Near_to_perfect
16	50756773	12	C	G,T	50756774	2,259	Crohn's disease	16q12.1	50722863	NOD2	rs5743289	intron_variant	1.00E-06	Perfect_to_near
16	64635403	14	C	T	64635403	296	abolite levels (Pyroglutam	16q21	64601500	C105371310 - LOC10537	rs16967753	stream_gene_v	1.00E-06	Near_to_perfect
16	79614117	22	T	G	79614116	1317	taining treatment in HIV-1	16q23.2	79580219	MAF	rs30388	itergenic_varia	1.00E-06	Perfect_to_near
16	82326815	24	G	A	82326805	338	Obesity-related traits	16q23.3	82293200	C105371365 - LOC100419	rs11863065	itergenic_varia	1.00E-06	Smaller
17	63642540	16	G	A	63642542	3944	ion deficit hyperactivity dis	17q24.1	65646424	CEP112	rs8074751	intron_variant	1.00E-06	Near_to_perfect
17	69108749	14	G	T	69108753	2880	Prostate cancer	17q24.3	71112612	CASC17	rs1859962	intron_variant	1.00E-06	Near_to_perfect
17	72268210	8	A	G	72268208	4587	formation processing spee	17q25.1	74272069	TTYH2 - DNAI2	rs7219585	ream_gene_va	1.00E-06	Near_to_perfect
18	53194958	20	T	C	53194961	52	Schizophrenia	18q21.2	55527730	TCF4	rs17512836	intron_variant	1.00E-06	Near_to_perfect
18	75605398	20	T	C	75605399	1991	emotherapy (neutropenia/le	18q23	77893443	GALR1 - LINC01029	rs9961113	itergenic_varia	1.00E-06	Perfect_to_near
19	45392254	24	C	T	45392254	553	-related macular degenera	19q13.32	44888997	PVRL2	rs6857	rime_UTR_var	1.00E-06	Near_to_perfect
19	45392254	24	C	T	45392254	553	-related macular degenera	19q13.32	44888997	PVRL2	rs6857	rime_UTR_var	1.00E-06	Near_to_perfect
19	45411944	8	T	C	45411941	754	heimer's disease biomark	19q13.32	44908684	APOE	rs429358	nissense_varia	1.00E-06	Perfect_to_near
1	79238010	22	T	C	79238015	1145	lisorder, bipolar disorder, r	1p31.1	78772330	LOC652549 - ADGRL4	rs4650608	itergenic_varia	1.00E-06	Near_to_perfect
1	84622511	28	G	T	84622513	2035	Breast cancer (male)	1p31.1	84156830	PRKACB	rs903263	intron_variant	1.00E-06	Near_to_perfect
1	92211016	24	G	A	92211020	227	Bone mineral density	1p22.1	91745463	TGFBR3	rs17131547	intron_variant	1.00E-06	Near_to_perfect
1	109417678	0	C	A	109417679	828	Fat distribution (HIV)	1p13.3	108875057	SPATA42 - GPSM2	rs7523050	intron_variant	1.00E-06	New
1	174552804	16	C	T	174552811	442	Migraine without aura	1q25.1	174583673	RABGAP1L	rs17301853	intron_variant	1.00E-06	Non_identical
1	185684617	18	C	T	185684623	77	tion in Tripanosoma cruzi s	1q25.3	185715491	LOC105371653 - HMCN1	rs74133262	itergenic_varia	1.00E-06	Near_to_perfect
1	232519146	20	A	C	232519150	2884	Immunoglobulin A	1q42.2	232383404	LOC105373198 - SIPA1L2	rs669408	itergenic_varia	1.00E-06	Near_to_perfect
20	11157012	8	A	T	11157011	3201	ponse to citalopram treatm	20p12.2	11176363	LOC105372528	rs6040399	itergenic_varia	1.00E-06	Near_to_perfect
20	20634105	18	T	C	20634106	383	IgG glycosylation	20p11.23	20653462	RALGAPA2	rs6132333	intron_variant	1.00E-06	Perfect_to_near
20	20634105	18	T	C	20634106	383	IgG glycosylation	20p11.23	20653462	RALGAPA2	rs6132333	intron_variant	1.00E-06	Perfect_to_near
20	26255986	0	C	T	26255996	849	n disease and lewy body p	20p11.1	26275360	IIR663AHG - LOC1002890	rs816535	itergenic_varia	1.00E-06	New
20	57164852	20	A	G	57164855	3688	liovascular disease risk fa	20q13.32	58589799	APCDD1L-AS1	rs127430	intron_variant	1.00E-06	Near_to_perfect
22	46632595	14	G	A	46632589	171	saturated fatty acid levels	22q13.31	46236692	PPARA	rs9615264	rime_UTR_var	1.00E-06	Bigger
2	2483627	12	T	G	2483626	1939	nin B levels in ischemic st	2p25.3	2479854	MYT1L - LOC105373389	rs12611820	itergenic_varia	1.00E-06	Near_to_perfect
2	63777852	40	T	C	63777858	29	taneous malignant melano	2p15	63550724	WDPCP	rs186133190	intron_variant	1.00E-06	Bigger
2	162867610	0	G	A	162867613	1904	Educational attainment	2q24.2	162011103	DPP4	rs10166311	intron_variant	1.00E-06	New
2	227092798	24	A	G	227092802	3759	Body mass index	2q36.3	226228086	OC646736 - LOC10537391	rs2176040	itergenic_varia	1.00E-06	Perfect_to_near
2	235302801	0	C	T	235302810	503	rement (high sodium and p	2q37.1	234394166	C105373933 - LOC105373	rs11887188	itergenic_varia	1.00E-06	New
2	237509202	0	G	A	237509207	1761	hol dependence (age at o	2q37.3	236600564	ACKR3 - LOC105373946	rs896543	itergenic_varia	1.00E-06	New
3	12923196	16	C	A	12923197	3257	Periodontitis (DPAL)	3p25.2	12881698	LOC105376956	rs2569991	intron_variant	1.00E-06	Near_to_perfect
4	27254624	14	T	A,C	27254628	84,386	r frontal gyrus grey matter	4p15.2	27253006	LOC101929199	rs1906528	intron_variant	1.00E-06	Near_to_perfect
4	72604298	10	C	G	72604299	1460	lic fatty liver disease histo	4q13.3	71738582	LOC105377271 - GC	rs222054	stream_gene_v	1.00E-06	Near_to_perfect
5	13798559	18	A	C	13798559	3959	Corneal astigmatism	5p15.2	13798450	DNAH5	rs795544	intron_variant	1.00E-06	Perfect_to_near
5	87756709	8	G	A	87756708	1472	Cognitive function	5q14.3	88460891	.OC102546226 - LINC0046	rs6452790	intron_variant	1.00E-06	Perfect_to_near

5	87965019	18	A	G	87965021	1504	Body mass index	5q14.3	88669203	LINC00461	rs6893807	intron_variant	1.00E-06	Near_to_perfect
5	95273412	22	T	A	95273410	1970	IgG glycosylation	5q15	95937706	ELL2	rs7700895	intron_variant	1.00E-06	Perfect_to_near
5	169074053	12	C	T	169074056	2098	Protein quantitative trait loc	5q35.1	169647052	DOCK2	rs169082	intron_variant	1.00E-06	Perfect_to_near
6	14598822	8	T	C	14598820	1654	otic treatment in schizoph	6p23	14598589	LOC101928354	rs7770731	intron_variant	1.00E-06	Near_to_perfect
6	22087074	16	T	A	22087074	1815	Refractive astigmatism	6p22.3	22086845	CASC15	rs12212674	intron_variant	1.00E-06	Perfect_to_near
6	22087074	16	T	A	22087074	1815	Refractive astigmatism	6p22.3	22086845	CASC15	rs12212674	intron_variant	1.00E-06	Perfect_to_near
6	29387000	8	G	T	29387003	120	iisocyanate-induced asthn	6p22.1	29419226	OR5V1	rs16894878	intron_variant	1.00E-06	Perfect_to_near
7	25608409	18	G	A	25608409	1589	d atherosclerosis in HIV in	7p15.2	25568789	OC105375196 - LOC64658	rs17151904	ream_gene_va	1.00E-06	Perfect_to_near
7	28911809	14	C	T	28911807	287	Vascular brain injury	7p14.3	28872190	CREB5 - TRIL	rs11769293	ntergenic_variar	1.00E-06	Perfect_to_near
7	82082439	20	T	C	82082438	188	iisocyanate-induced asthn	7q21.11	82453122	CACNA2D1 - MTHFD2P5	rs10268774	ntergenic_variar	1.00E-06	Perfect_to_near
8	6202971	26	G	A	6202964	22	iisocyanate-induced asthn	8p23.1	6345443	C105377797 - LOC100287(	rs114252942	ntergenic_variar	1.00E-06	Near_to_perfect
8	81308147	16	T	A	81308150	3098	Atopic dermatitis	8q21.13	80395915	C100216346 - LOC10537!	rs7000782	ntergenic_variar	1.00E-06	Perfect_to_near
8	92360395	8	T	C	92360396	898	Fat distribution (HIV)	8q21.3	91348168	SLC26A7	rs921231	intron_variant	1.00E-06	Near_to_perfect
9	73784263	22	C	T	73784264	2148	Longevity	9q21.12	71169348	TRPM3	rs4745062	intron_variant	1.00E-06	Near_to_perfect
9	121125744	12	T	C	121125746	2937	Osteoarthritis biomarkers	9q33.1	118363468	C105376247 - LOC105376	rs10429475	ntergenic_variar	1.00E-06	Near_to_perfect
9	138029699	18	T	G	138029701	3546	ponse to hepatitis C treatr	9q34.3	135137855	OLFM1 - LOC102723948	rs10776934	atory_region_v	1.00E-06	Perfect_to_near
X	37999648	20	A	T	37999652	1421	Anorexia nervosa	Xp11.4	38140399	SYTL5 - SRPX	rs56156506	intron_variant	1.00E-06	Perfect_to_near
X	133322600	18	C	T	133322604	229	F alpha therapy in inflamn	Xq26.2	134188574	MIR106A - CCDC160	rs765132	ntergenic_variar	1.00E-06	Near_to_perfect
12	121363715	20	T	C	121363724	3894	Longevity	12q24.31	120925921	RPL12P33 - HNF1A-AS1	rs6489785	atory_region_v	1.00E-06	Non_identical
14	72432152	8	G	A	72432149	538	political preferences (env	14q24.2	71965432	RGS6	rs4902960	nissense_variar	1.00E-06	Bigger
1	169100085	22	C	T	169100095	1341	Electrocardiographic traits	1q24.2	169130857	ATP1B1	rs11809180	intron_variant	1.00E-06	Non_identical
22	37551613	22	A	G	37551607	1386	Rheumatoid arthritis	22q12.3	37155567	IL2RB - C1QTNF6	rs743777	intron_variant	1.00E-06	Smaller
2	123291032	24	C	T	123291022	3298	function and prostate canc	2q14.3	122533446	C105373593 - LOC10537!	rs1527243	ntergenic_variar	1.00E-06	Smaller
2	157868861	18	A	G	157868868	136	iisocyanate-induced asthn	2q24.1	157012356	LOC105373710	rs16841200	stream_gene_v	1.00E-06	Non_identical
2	164630962	28	T	C	164630952	707	iisocyanate-induced asthn	2q24.3	163774442	FIGN - GRB14	rs7576072	intron_variant	1.00E-06	Non_identical
2	197920160	20	C	T	197920169	3602	a levels in systemic lupus	2q33.1	197055445	ANKRD44	rs4850410	intron_variant	1.00E-06	Smaller
3	175103607	22	G	T	175103614	1983	Metabolite levels (MHPG)	3q26.31	175385825	NAALADL2	rs62287976	intron_variant	1.00E-06	Bigger
4	40159620	20	T	C	40159617	104	Platelet thrombus formatio	4p14	40157997	N4BP2	rs7656730	rime_UTR_var	1.00E-06	Smaller
4	138134584	20	A	G	138134581	1206	se response to radiation tl	4q28.3	137213427	LOC729307 - STMN1P2	rs10519410	ream_gene_va	1.00E-06	Non_identical
5	77348647	30	A	C	77348640	171	iisocyanate-induced asthn	5q14.1	78052816	AP3B1	rs73132886	intron_variant	1.00E-06	Non_identical
5	175244040	22	C	T	175244034	107	yperactivity disorder (inatt	5q35.2	175817031	CPLX2	rs7448069	intron_variant	1.00E-06	Smaller
6	31802458	18	C	G	31802465	616	IgG glycosylation	6p21.33	31834688	HSPA1B - C6orf48	rs4711279	rime_UTR_var	1.00E-06	Non_identical
6	34582267	22	T	G	34582274	182	yopathy in Tripanosoma cr	6p21.31	34614497	C6orf106	rs182503338	intron_variant	1.00E-06	Smaller
6	72139563	20	G	A	72139572	2663	erebrospinal AB1-42 leve	6q13	71429869	LOC102724000	rs1727638	ntergenic_variar	1.00E-06	Smaller
7	31136890	26	C	G	31136897	1413	min B levels in ischemic st	7p14.3	31097283	ADCYAP1R1	rs2267739	intron_variant	1.00E-06	Non_identical
10	6791032	8	C	G	6791034	121	rence (psychosocial stres:	10p14	6749072	LOC105376385	rs7094310	atory_region_v	2.00E-06	Near_to_perfect
10	32634970	20	G	A	32634972	942	Sexual dysfunction (female	10p11.22	32346044	EPC1	rs2370759	intron_variant	2.00E-06	Near_to_perfect
10	44753866	0	T	C	44753867	1666	Coronary artery disease	10q11.21	44258419	LINC00841 - LOC1019294!	rs501120	stream_gene_v	2.00E-06	New
10	60668563	18	G	A	60668571	133	al in Tripanosoma cruzi ser	10q21.1	58908811	BICC1 - LINC00844	rs61458523	ntergenic_variar	2.00E-06	Perfect_to_near
10	101365311	20	G	A	101365313	647	coprotein I (β2-GPI) p	10q24.2	99605556	NKX2-3 - SLC25A28	rs11190179	stream_gene_v	2.00E-06	Near_to_perfect
10	123338980	12	A	G	123338975	1549	Breast cancer	10q26.13	121579461	FGFR2	rs1078806	intron_variant	2.00E-06	Near_to_perfect
10	128267637	0	T	A	128267640	1393	Metabolite levels (MHPG)	10q26.2	126579071	C10orf90	rs11245052	intron_variant	2.00E-06	New
11	45947470	20	G	C	45947464	815	Axial length	11p11.2	45925913	GYLTL1B	rs10838532	intron_variant	2.00E-06	Non_identical
11	94667965	8	G	A	94667964	3957	hyperactivity disorder and	11q21	94934799	AMOTL1 - CWC15	rs10831284	atory_region_v	2.00E-06	Near_to_perfect
12	28815789	18	G	A	28815792	3655	abolite levels (Pyroglutam	12p11.22	28662859	C101928705 - LOC105369	rs1606355	ntergenic_variar	2.00E-06	Near_to_perfect
12	122781895	10	G	A	122781897	164	Body mass index	12q24.31	122297350	CLIP1	rs11057405	intron_variant	2.00E-06	Perfect_to_near
12	123457618	22	C	T	123457619	3275	Platelet count	12q24.31	122973072	ABCB9	rs7296418	rime_UTR_var	2.00E-06	Perfect_to_near
12	124375208	0	A	G	124375210	2101	Osteoarthritis (hip)	12q24.31	123890663	DNAH10	rs10773046	intron_variant	2.00E-06	New
13	103928004	22	T	A	103928007	434	Prostate cancer	13q33.1	103275657	SLC10A2 - LOC105370338	rs1529276	ntergenic_variar	2.00E-06	Near_to_perfect
14	29132878	0	G	A	29132877	2624	Ulcerative colitis	14q12	28663671	LOC105370422 - BTF3P2	rs1956388	ntergenic_variar	2.00E-06	Near_to_perfect
14	68973541	14	A	G	68973546	2600	Breast cancer	14q24.1	68508657	RAD51B	42347, rs75736	intron_variant	2.00E-06	Perfect_to_near
15	33050423	16	C	T	33050423	2302	Orofacial clefts	15q13.3	32758222	GREM1 - FMN1	rs1258763	ntergenic_variar	2.00E-06	Perfect_to_near
15	45357404	0	T	G	45357410	3384	er's disease in APOE e4+	15q21.1	45065212	SORD	rs2854437	intron_variant	2.00E-06	New
15	72169466	28	G	A	72169466	2184	bolite levels (HVA/MHPG	15q23	71877125	MYO9A	rs12050794	intron_variant	2.00E-06	Near_to_perfect
15	86067305	12	G	A	86067306	3109	Interstitial lung disease	15q25.3	85524075	AKAP13	rs6496044	intron_variant	2.00E-06	Perfect_to_near
16	68383046	8	G	A,C	68383047	57,917	Magnesium levels	16q22.1	68349144	PRMT7	rs7197653	intron_variant	2.00E-06	Perfect_to_near
17	35898341	18	G	T	35898342	1983	angiotensin II receptor blo	17q12	37538240	SYNRG	rs2074409	intron_variant	2.00E-06	Near_to_perfect
17	77273509	16	G	A	77273513	490	rate levels (BMI interactio	17q25.3	79277431	RBFOX3	rs898534	intron_variant	2.00E-06	Near_to_perfect
18	68153616	18	T	C	68153620	3638	onse to antipsychotic trea	18q22.2	70486384	LOC105376872	rs11663206	ream_gene_va	2.00E-06	Perfect_to_near
18	71267053	14	C	T	71267054	1491	n disease and lewy body p	18q22.3	73599819	C100505817 - LOC10537	rs12959200	ntergenic_variar	2.00E-06	Perfect_to_near
19	1205889	8	C	T	1205889	88	al in Tripanosoma cruzi ser	19p13.3	1205890	STK11	rs147615524	rime_UTR_var	2.00E-06	Perfect_to_near
19	1811603	20	C	T	1811603	1984	Bipolar disorder	19p13.3	1811604	ATP8B3	rs7250872	nissense_variar	2.00E-06	Perfect_to_near
19	42521108	8	T	G	42521108	1640	Bipolar disorder	19q13.2	42016956	GRIK5	rs8099939	intron_variant	2.00E-06	Perfect_to_near
1	17613804	24	A	G	17613809	2134	IgA nephropathy	1p36.13	17287314	PADI3 - PADI4	rs12568771	stream_gene_v	2.00E-06	Near_to_perfect
1	59762463	20	A	G	59762468	2481	Obesity-related traits	1p32.1	59296796	FGGY	rs835367	rime_UTR_var	2.00E-06	Near_to_perfect
1	76477209	20	A	C	76477207	695	Pubertal anthropometrics	1p31.1	76011522	LOC101927342	rs12122440	intron_variant	2.00E-06	Near_to_perfect
1	150066383	10	C	T	150066384	294	Uric acid levels	1q21.2	150094287	VPS45	rs12058524	intron_variant	2.00E-06	Near_to_perfect
1	169543132	12	G	A	169543131	1359	Optic disc area	1q24.2	169573893	F5	rs12406092	intron_variant	2.00E-06	Near_to_perfect
1	209727254	20	T	A	209727257	1715	Obesity (extreme)	1q32.2	209553912	C105372898 - LOC10537	rs12130212	intron_variant	2.00E-06	Near_to_perfect
1	209989279	0	A	G	209989270	4121	Orofacial clefts	1q32.2	209815925	IRF6 - DIEXF	rs642961	atory_region_v	2.00E-06	New
1	233294775	0	C	A	233294777	3412	Body mass index	1q42.2	233159031	PCNXL2	rs7550169	intron_variant	2.00E-06	New
1	240904625	8	A	G	240904628	54	ongevity (90 years and olde	1q43	240741328	LOC100506929	rs4611001	ream_gene_va	2.00E-06	Perfect_to_near
20	5949534	22	G	A	5949534	297	Preeclampsia	20p12.3	5968888	MCM8	rs4815879	intron_variant	2.00E-06	Near_to_perfect
20	8836779	0	G	A	8836771	2271	Cognitive performance	20p12.3	8856124	PLCB1	rs6056209	intron_variant	2.00E-06	New
20	20848966	26	A	G	20848968	2665	Cognitive function	20p11.23	20868325	C105372555 - LOC10537	rs6047116	ntergenic_variar	2.00E-06	Near_to_perfect
20	48632602	18	T	C	48632610	1754	Psoriasis	20q13.13	50016073	SNAI1 - LOC105372654	rs7352944	atory_region_v	2.00E-06	New
22	22047971	8	C	T	22047969	49	al in Tripanosoma cruzi ser	22q11.21	21693680	PPIL2	rs79384503	intron_variant	2.00E-06	Near_to_perfect
22	26403596	18	G	A	26403599	505	bipolar disorder and depres	22q12.1	26007633	MYO18B	rs1001021	intron_variant	2.00E-06	Near_to_perfect
2	79632344	14	A	G	79632347	1916	Alcohol consumption	2p12	79405221	C105374823 - LOC10192	rs2100290	intron_variant	2.00E-06	Perfect_to_near
2	113890371	10	C	T	113890373	71	Obesity-related traits	2q14.1	113132796	IL1RN	rs4252023	nonymous_vari	2.00E-06	Near_to_perfect

2	116241185	12	G	A	116241186	2345	Bipolar disorder	2q14.1	115483610	DPP10	rs1375144	intron_variant	2.00E-06	Near_to_perfect
2	155070564	8	A	T	155070567	4349	Aging traits	2q24.1	154214054	GALNT13	rs958672	intron_variant	2.00E-06	Near_to_perfect
2	179641980	20	C	T	179641975	377	QT interval	2q31.2	178777248	LOC101927055, TTN	rs12476289	nissense_variar	2.00E-06	Near_to_perfect
2	185293912	22	G	A	185293916	1394	ıl in Tripanosoma cruzi ser	2q32.1	184429189	C105373776 - LOC105373	rs11691711	ntergenic_varia	2.00E-06	Perfect_to_near
2	202060827	18	C	T	202060820	870	Trans fatty acid levels	2q33.1	201196097	CASP10	rs3731714	intron_variant	2.00E-06	Smaller
2	228397575	20	T	G	228397566	2319	irway hyperresponsivenes	2q36.3	227532850	AGFG1	rs6731443	intron_variant	2.00E-06	Perfect_to_near
3	60763258	26	G	T	60763256	1294	Breast size	3p14.2	60777523	FHIT	rs11919041	intron_variant	2.00E-06	Near_to_perfect
3	122003759	16	G	T	122003757	472	Calcium levels	3q21.1	122284910	CASR	rs1801725	nissense_variar	2.00E-06	Perfect_to_near
3	131574483	14	G	A	131574485	800	Body mass index	3q22.1	131855641	LOC105374113, CPNE4	rs9856151	intron_variant	2.00E-06	Near_to_perfect
3	168252299	14	C	T	168252304	114	ıl in Tripanosoma cruzi ser	3q26.2	168534516	EGFEM1P	rs6782264	intron_variant	2.00E-06	Perfect_to_near
3	171558342	18	G	A	171558344	157	Body mass index	3q26.31	171840554	PLD1 - TMEM212	rs6794092	ream_gene_va	2.00E-06	Near_to_perfect
4	27066038	24	G	A	27066038	1114	Hirschsprung disease	4p15.2	27064416	STIM2 - LOC101929199	rs11725593	ntergenic_varia	2.00E-06	Near_to_perfect
4	35410633	16	T	C	35410631	2126	QT interval	4p15.1	35409009	C105374395 - LOC105374	rs1533317	ntergenic_varia	2.00E-06	Near_to_perfect
4	56298192	14	G	A	56298194	3601	Personality dimensions	4q12	55432027	TMEM165, CLOCK	rs6832769	rime_UTR_var	2.00E-06	Near_to_perfect
4	92039004	20	A	G	92039007	2685	l cancer (calcium intake ir	4q22.1	91117856	CCSER1	rs6855885	intron_variant	2.00E-06	Near_to_perfect
4	186612674	18	G	A	186612675	4640	e tissue/subcutaneous adi	4q35.1	185691521	SORBS2	rs4376189	intron_variant	2.00E-06	Near_to_perfect
5	71410357	18	G	C	71410356	3601	ion deficit hyperactivity di	5q13.2	72114529	MAP1B	rs2199161	intron_variant	2.00E-06	Perfect_to_near
5	84583771	18	G	T	84583769	437	od trace element (Cu leve	5q14.3	85287951	C101060076 - LOC101929	rs12153606	ntergenic_varia	2.00E-06	Near_to_perfect
5	122685129	8	A	G	122685128	2120	Weight	5q23.2	123349434	CEP120	rs2115172	intron_variant	2.00E-06	Perfect_to_near
6	24544967	8	C	T	24544967	851	Radiation response	6p22.3	24544739	KIAA0319	rs16889440	rime_UTR_var	2.00E-06	Near_to_perfect
6	37278928	22	T	C	37278933	751	Acne (severe)	6p21.2	37311157	TBC1D22B	rs149709	intron_variant	2.00E-06	Perfect_to_near
6	44739948	12	C	T	44739949	303	Obesity-related traits	6p21.1	44772212	LOC101929770	rs1342371	atory_region_v	2.00E-06	Perfect_to_near
6	66598194	0	G	A	66598194	1234	HIV-1 viral setpoint	6q12	65888301	ADH5P4 - LOC105377838	rs10455590	ntergenic_varia	2.00E-06	New
6	80953256	0	C	A	80953257	3012	Sitting height ratio	6q14.1	80243540	BCKDHB	rs9341808	intron_variant	2.00E-06	New
6	88032385	14	G	C	88032390	73	ıl in Tripanosoma cruzi ser	6q15	87322672	GJB7, SMIM8	rs7341237	ce_region_vari	2.00E-06	Near_to_perfect
6	99782876	8	A	C	99782879	1142	Illicit drug use	6q16.2	99335003	FAXC	rs17059400	intron_variant	2.00E-06	Near_to_perfect
6	127391852	20	C	G	127391844	2209	Iron status biomarkers	6q22.33	127070699	LOC105377989	rs972275	ntergenic_varia	2.00E-06	Near_to_perfect
7	28271686	18	G	A	28271690	94	iisocyanate-induced asthn	7p15.1	28232071	JAZF1-AS1	rs73302615	intron_variant	2.00E-06	Near_to_perfect
7	70587144	20	T	C	70587145	794	e-related hearing impairm	7q11.22	71122159	AUTS2 - WBSCR17	rs62459614	ntergenic_varia	2.00E-06	Near_to_perfect
7	95455840	12	C	T	95455845	39	Alzheimer's disease neurc	7q21.3	95826533	DYNC1I1	rs3779483	intron_variant	2.00E-06	Near_to_perfect
7	95455840	12	C	T	95455845	39	Alzheimer's disease neurc	7q21.3	95826533	DYNC1I1	rs3779483	intron_variant	2.00E-06	Near_to_perfect
7	113741102	8	T	G	113741105	1787	IgG glycosylation	7q31.1	114101050	FOXP2	rs6466479	intron_variant	2.00E-06	Perfect_to_near
7	113741102	8	T	G	113741105	1787	IgG glycosylation	7q31.1	114101050	FOXP2	rs6466479	intron_variant	2.00E-06	Perfect_to_near
7	129356206	8	G	A	129356206	2841	Obesity-related traits	7q32.2	129716366	NRF1	rs9641855	intron_variant	2.00E-06	Near_to_perfect
7	132189687	18	G	A	132189689	1740	Pulmonary function decline	7q32.3	132504930	PLXNA4	rs10808265	intron_variant	2.00E-06	Perfect_to_near
7	154509318	20	T	C	154509324	1980	QT interval	7q36.2	154717614	DPP6	rs12666280	intron_variant	2.00E-06	Non_identical
8	516487	18	A	C	516479	3741	lated macular degeneratic	8p23.3	566479	TDRP - ERICH1	rs722782	ntergenic_varia	2.00E-06	Perfect_to_near
8	12772757	20	G	T	12772755	165	orectal cancer (diet interac	8p22	12915246	LOC105379289 - KIAA1456	rs6989010	ntergenic_varia	2.00E-06	Near_to_perfect
8	54410209	16	G	A	54410208	1621	ponse to antineoplastic ag	8q11.23	53497648	C102724359 - LOC100507	rs10958369	ntergenic_varia	2.00E-06	Near_to_perfect
8	60178720	16	C	A	60178721	1558	e tissue/subcutaneous adi	8q12.1	59266162	.OC100505501 - NUDT15P	rs10089517	ntergenic_varia	2.00E-06	Near_to_perfect
8	92360395	8	T	C	92360396	898	Fat distribution (HIV)	8q21.3	91348168	SLC26A7	rs921231	intron_variant	2.00E-06	Near_to_perfect
8	115578306	8	T	G	115578307	1622	stress disorder (asjusted f	8q23.3	114566078	LOC105375710 - TRPS1	rs7014900	ntergenic_varia	2.00E-06	Near_to_perfect
9	7081673	8	A	G	7081674	378	HIV-1 susceptibility	9p24.1	7081674	KDM4C	rs16925298	intron_variant	2.00E-06	Near_to_perfect
9	10192283	0	A	C	10192290	2954	y and major depressive di	9p23	10192290	PTPRD	rs294856	intron_variant	2.00E-06	New
9	11930370	18	A	G	11930364	53	Hippocampal volume	9p23	11930364	C101929446 - LOC105375	rs35419961	ntergenic_varia	2.00E-06	Near_to_perfect
9	11964867	18	A	C	11964872	1477	Inflammatory skin disease	9p23	11964872	C101929446 - LOC105375	rs12001137	ntergenic_varia	2.00E-06	Perfect_to_near
9	82163310	8	C	T	82163313	433	ıl in Tripanosoma cruzi ser	9q21.31	79548398	LOC102723932	rs11138290	ntergenic_varia	2.00E-06	Near_to_perfect
X	107413928	8	G	A	107413930	293	Electrodermal activity	Xq22.3	108170700	COL4A6	rs2295912	nonymous_vari	2.00E-06	Perfect_to_near
10	29336854	20	G	A	29336849	4652	oleic acid (16:1n-7) plasma	10p12.1	29047920	C101929236 - LOC105376	rs788076	ntergenic_varia	2.00E-06	Non_identical
16	84046720	18	A	G	84046715	923	lic fatty liver disease histo	16q23.3	84013110	SLC38A8	rs11864146	intron_variant	2.00E-06	Non_identical
18	41480532	20	C	T	41480527	3510	Obesity-related traits	18q12.3	43900562	LOC105372088	rs1380836	ntergenic_varia	2.00E-06	Non_identical
22	17597467	14	G	A	17597462	832	Heschl's gyrus morphology	22q11.1	17116572	CECR6	rs971768	rime_UTR_var	2.00E-06	Smaller
22	37551613	22	A	G	37551607	1386	pe 1 diabetes autoantibod	22q12.3	37155567	IL2RB - C1QTNF6	rs743777	intron_variant	2.00E-06	Smaller
2	37940546	18	T	C	37940542	998	r volume (Alzheimer's dise	2p22.2	37713399	CDC42EP3 - LOC10537446	rs4670766	intron_variant	2.00E-06	Non_identical
2	174504931	16	G	A	174504924	556	ole myeloma (IgH transloc	2q31.1	173640196	OC643997 - LOC1053737	rs13028485	ntergenic_varia	2.00E-06	Bigger
3	172481	20	A	G	172477	319	oressant treatment in majo	3p26.3	130794	LOC101927174	rs6795349	ntergenic_varia	2.00E-06	Non_identical
3	99167097	22	A	C	99167107	2297	RR interval (heart rate)	3q12.1	99448263	C105374004 - LOC105374	rs2670321	ntergenic_varia	2.00E-06	Smaller
3	103307646	12	T	G	103307642	3018	political preferences (env	3q13.11	103588798	MIR548AB - RAP1BP2	rs7612581	ntergenic_varia	2.00E-06	Smaller
4	68056604	20	T	C	68056613	3665	Glucose homeostasis traits	4q13.2	67190895	C105377262 - LOC100996	rs7690543	ntergenic_varia	2.00E-06	Non_identical
5	134240228	18	T	C	134240235	3150	Type 2 diabetes	5q31.1	134904545	TXNDC15 - PCBD2	rs319598	ream_gene_va	2.00E-06	Non_identical
6	20109584	24	G	A	20109592	4019	Obesity-related traits	6p22.3	20109361	MBOAT1	rs2457335	intron_variant	2.00E-06	Non_identical
6	30666675	22	C	G	30666669	317	IgG glycosylation	6p21.33	30698892	RPL7P4 - MDC1	rs9468811	ream_gene_va	2.00E-06	Non_identical
7	7848906	22	T	C	7848912	718	Obesity-related traits	7p21.3	7809281	UMAD1	rs10259199	intron_variant	2.00E-06	Smaller
8	99022001	24	C	A	99022009	3581	hemotherapy (neutropenia	8q22.2	98009781	MATN2	rs2444896	intron_variant	2.00E-06	Non_identical
9	6068070	18	A	T	6068077	739	Asthma and hay fever	9p24.1	6068077	RANBP6 - IL33	rs343496	stream_gene_v	2.00E-06	Non_identical
9	84308954	18	G	A	84308948	1989	Type 2 diabetes	9q21.32	81694033	LOC101927502	rs2796441	intron_variant	2.00E-06	Non_identical
10	88162239	14	A	G	88162238	4383	rophic lateral sclerosis (sp	10q23.2	86402481	GRID1 - WAPAL	rs10458771	ntergenic_varia	3.00E-06	Perfect_to_near
10	94168794	32	G	A	94168797	1593	Airflow obstruction	10q23.33	92409040	MARCH5 - MARK2P9	rs2263638	ntergenic_varia	3.00E-06	Near_to_perfect
10	94462877	14	C	T	94462882	2285	Type 2 diabetes	10q23.33	92703125	HHEX - EXOC6	rs1111875	atory_region_v	3.00E-06	Near_to_perfect
10	104207796	22	C	A	104207799	4372	Social autistic-like traits	10q24.32	102448042	MIR146B - RPARP-AS1	rs927821	ream_gene_va	3.00E-06	Near_to_perfect
10	114758351	14	C	T	114758349	1141	ulin-related traits (interactio	10q25.2	112998590	TCF7L2	rs7903146	intron_variant	3.00E-06	Near_to_perfect
10	135225665	8	T	C	135225666	1438	ystemic lupus erythematos	10q26.3	133412162	MTG1	rs10857712	intron_variant	3.00E-06	Near_to_perfect
11	5959757	26	A	C	5959757	1456	DNA methylation (variation	11p15.4	5938527	OR52E4 - OR56A3	rs3858526	rime_UTR_var	3.00E-06	Near_to_perfect
11	32364187	18	G	A	32364187	898	Tuberculosis	11p13	32342641	RCN1 - WT1	rs2057178	intron_variant	3.00E-06	Perfect_to_near
11	60906450	12	A	G	60906450	4063	Rheumatoid arthritis	11q12.2	61138978	VPS37C	rs508970	intron_variant	3.00E-06	Near_to_perfect
11	121497886	20	T	C	121497884	1781	neous preterm birth (preter	11q24.1	121627175	SORL1	rs10892761	g_transcript_ex	3.00E-06	Near_to_perfect
11	128147447	16	C	G	128147449	1032	Psoriasis	11q24.3	128277554	C105369564 - LOC105369	rs55974252	ntergenic_varia	3.00E-06	Near_to_perfect
13	34903089	18	G	A	34903092	4594	Obesity-related traits	13q13.2	34328955	C105370156 - LOC105370	rs571411	ntergenic_varia	3.00E-06	Perfect_to_near
13	79410575	16	T	C	79410574	116	Conduct disorder	13q31.1	78836439	LINC00331	rs11838918	ntergenic_varia	3.00E-06	Near_to_perfect
13	113659105	8	G	A	113659108	1214	Bladder cancer	13q34	113004794	MCF2L	rs4907479	intron_variant	3.00E-06	Perfect_to_near

14	90034974	8	G	A	90034972	1518	ose-related traits (interacti	14q32.11	89568628	FOXN3	rs8004664	intron_variant	3.00E-06	Near_to_perfect
15	60910559	22	C	T	60910550	572	r volume (Alzheimer's dise	15q22.2	60618351	RORA, RORA-AS1	rs3784609	intron_variant	3.00E-06	Smaller
15	95165585	14	T	G	95165580	999	Sudden cardiac arrest	15q26.2	94622351	C105370985 - LOC105370	rs1014922	itergenic_varia	3.00E-06	Smaller
15	96844724	18	C	T	96844727	1279	ncephalographic traits in al	15q26.2	96301498	NR2F2-AS1	rs7181753	intron_variant	3.00E-06	Perfect_to_near
16	24621347	16	G	A	24621348	1078	Electrodermal activity	16p12.1	24610027	RBBP6 - LINC01567	rs17831015	ream_gene_va	3.00E-06	Near_to_perfect
16	35088128	14	T	C	35088131	1114	oleic acid (16:1n-7) plasma	16p11.1	35853760	IMGN2P41 - LOC10192992	rs12599426	itergenic_varia	3.00E-06	Near_to_perfect
16	49750792	14	T	C	49750790	1047	Inattentive symptoms	16q12.1	49716879	ZNF423	rs17281813	intron_variant	3.00E-06	Near_to_perfect
16	56995229	30	C	A	56995236	2737	Triglycerides	16q13	56961324	HERPUD1 - CETP	rs1800775	ream_gene_va	3.00E-06	New
16	82326815	24	G	A	82326805	338	Obesity-related traits	16q23.3	82293200	C105371365 - LOC100419	rs11863065	itergenic_varia	3.00E-06	Smaller
17	64517313	16	T	A	64517313	1971	Coronary artery calcificatio	17q24.2	66521195	PRKCA	rs11651708	intron_variant	3.00E-06	Perfect_to_near
18	322520	18	C	T	322522	1059	Obesity-related traits	18p11.32	322522	COLEC12	rs621636	intron_variant	3.00E-06	Near_to_perfect
18	27956443	10	C	T	27956443	356	Obesity-related traits	18q12.1	30376477	LOC105372047 - DSC3	rs6508673	atory_region_v	3.00E-06	Near_to_perfect
18	46227444	20	C	G	46227444	574	IgG glycosylation	18q21.1	48701073	CTIF	rs16949825	intron_variant	3.00E-06	Perfect_to_near
18	52863108	22	C	G	52863108	1614	Diabetic retinopathy	18q21.2	55195877	LOC101927229 - TCF4	rs1970671	itergenic_varia	3.00E-06	Near_to_perfect
19	3435543	20	G	A	3435545	127	on in Tripanosoma cruzi s	19p13.3	3435547	NFIC	rs72974768	intron_variant	3.00E-06	Near_to_perfect
19	49228268	24	T	C	49228272	1082	Bipolar disorder	19q13.33	48725015	RASIP1	rs2287921	g_transcript_ex	3.00E-06	Perfect_to_near
1	101540997	14	T	C	101540999	990	QT interval (interaction)	1p21.2	101075443	LOC102606465	rs17450029	g_transcript_ex	3.00E-06	Perfect_to_near
1	113313558	14	C	A	113313563	991	peractive-impulsive sympto	1p13.2	112770941	NUTF2P4 - LINC01356	rs11590090	itergenic_varia	3.00E-06	Perfect_to_near
1	114715583	16	G	A	114715584	306	lar disorder and schizoph	1p13.2	114172962	SYT6 - TRIM33	rs2774292	itergenic_varia	3.00E-06	Perfect_to_near
1	157669277	12	C	T	157669278	2315	Multiple sclerosis	1q23.1	157699488	FCRL3	rs3761959	intron_variant	3.00E-06	Perfect_to_near
1	159012639	16	C	T	159012646	2786	utrophil count in HIV-infect	1q23.1	159042856	IFI16	rs2570916	intron_variant	3.00E-06	Non_identical
1	239601967	14	A	C	239601969	291	Platelet count	1q43	239438669	CHRM3	rs6677208	intron_variant	3.00E-06	Near_to_perfect
20	20634105	18	T	C	20634106	383	IgG glycosylation	20p11.23	20653462	RALGAPA2	rs6132333	intron_variant	3.00E-06	Perfect_to_near
20	46779241	0	C	T	46779235	1811	ephrotic syndrome (acquire	20q13.13	48150492	C105372640 - LOC105372	rs11086243	itergenic_varia	3.00E-06	New
20	47089548	26	C	T	47089537	2002	hyperactivity disorder (com	20q13.13	48461291	C105372644 - LOC105372	rs4458264	atory_region_v	3.00E-06	Smaller
20	47089548	26	C	T	47089537	2002	tivity disorder (hyperactivit	20q13.13	48461291	C105372644 - LOC105372	rs4458264	atory_region_v	3.00E-06	Smaller
22	32783903	12	T	C	32783904	278	IgG glycosylation	22q12.3	32387917	RTCB	rs12530	rime_UTR_var	3.00E-06	Near_to_perfect
2	9291226	22	T	C	9291234	425	iisocyanate-induced asthm	2p25.1	9151105	C105373417 - LOC100506	rs73912949	stream_gene_v	3.00E-06	Bigger
2	20335701	20	G	A	20335709	2817	hemotherapy (neutropenia	2p24.1	20135948	LOC105373463 - RPS16P2	rs4666360	atory_region_v	3.00E-06	Bigger
2	26112516	0	T	A	26112518	2691	Multiple myeloma	2p23.3	25889649	ASXL2 - KIF3C	rs12986445	ream_gene_va	3.00E-06	New
2	69033583	8	C	T	69033583	4	arginine levels (asymmetri	2p13.3	68806451	ARHGAP25	rs115522963	intron_variant	3.00E-06	Near_to_perfect
2	99465491	30	A	G	99465502	2931	Bipolar disorder	2q11.2	98849039	KIAA1211L	rs6733011	intron_variant	3.00E-06	Smaller
2	211608378	18	T	G	211608379	2352	te levels in obese individu	2q34	210743655	CPS1 - RPS27P10	rs4673553	itergenic_varia	3.00E-06	Near_to_perfect
2	229969804	0	A	G	229969798	1696	ychotic treatment in schizo	2q36.3	229105082	PID1	rs6436839	intron_variant	3.00E-06	New
2	234183359	0	A	G	234183368	1983	Crohn's disease	2q37.1	233274722	ATG16L1	rs2241880	nissense_varia	3.00E-06	New
2	237521847	0	C	A	237521842	1660	Airflow obstruction	2q37.3	236613199	C105373947 - LOC105373	rs7607316	itergenic_varia	3.00E-06	New
3	3650241	12	A	C	3650239	616	Response to amphetamine	3p26.2	3608555	C105376928 - LOC100130	rs9837561	intron_variant	3.00E-06	Near_to_perfect
3	42894425	14	C	G	42894427	1591	apine-induced agranulocy	3p22.1	42852935	ACKR2	rs10865924	intron_variant	3.00E-06	Perfect_to_near
3	55313399	0	A	C	55313400	4237	Type 2 diabetes	3p14.3	55279372	C105377097 - LOC105377	rs358806	itergenic_varia	3.00E-06	New
3	109204409	14	C	T	109204413	108	Cardiac hypertrophy	3q13.13	109485566	LINC01205	rs769554	intron_variant	3.00E-06	Perfect_to_near
3	122356449	16	G	A	122356451	182	Periodontitis (CDC/AAP)	3q21.1	122637604	PARP15	rs78411303	rime_UTR_var	3.00E-06	Perfect_to_near
4	7423753	26	C	A	7423751	2046	Obesity-related traits	4p16.1	7422024	SORCS2	rs7694661	intron_variant	3.00E-06	Perfect_to_near
5	8543192	18	C	T	8543190	1565	Verbal declarative memory	5p15.31	8543078	LOC105374647	rs1633735	itergenic_varia	3.00E-06	Perfect_to_near
5	8543192	18	C	T	8543190	1565	Verbal declarative memory	5p15.31	8543078	LOC105374647	rs1633735	itergenic_varia	3.00E-06	Perfect_to_near
5	26139138	22	A	T	26139138	489	Visceral fat	5p14.1	26139029	MSNP1 - CDH9	rs4701523	itergenic_varia	3.00E-06	Near_to_perfect
5	34266579	18	C	A	34266577	3910	Inflammatory skin disease	5p13.2	34266472	LOC105374719	rs11743355	itergenic_varia	3.00E-06	Perfect_to_near
5	107419544	20	C	T	107419548	1205	Body mass index	5q21.3	108083847	FBXL17	rs288232	intron_variant	3.00E-06	Perfect_to_near
5	146320814	22	G	T	146320823	1271	Alcohol dependence	5q32	146941260	PPP2R2B	rs1864982	intron_variant	3.00E-06	Non_identical
5	156945149	22	T	C	156945148	249	sorder (body mass index ir	5q33.3	157518140	ADAM19	rs58873874	intron_variant	3.00E-06	Perfect_to_near
6	8001114	18	A	G	8001117	846	Coronary artery calcificatio	6p24.3	8000884	BLOC1S5-TXNDC5	rs9328448	intron_variant	3.00E-06	Perfect_to_near
6	13216060	8	A	G	13216058	2387	Hip geometry	6p24.1	13215826	PHACTR1	rs4715166	intron_variant	3.00E-06	Perfect_to_near
6	32076497	22	G	A	32076499	1584	Eosinophil counts	6p21.32	32108722	TNXB	rs2269426	intron_variant	3.00E-06	Near_to_perfect
6	32411648	16	T	G	32411646	3303	on-obstructive azoosperm	6p21.32	32443869	HLA-DRA	rs7192	nissense_varia	3.00E-06	Perfect_to_near
6	139831178	12	G	A	139831180	2642	ood trace element (Se leve	6q24.1	139510043	OC645434 - LOC10537802	rs679582	itergenic_varia	3.00E-06	Near_to_perfect
6	149435108	8	T	C	149435111	1937	Diabetic retinopathy	6q25.1	149113975	LOC729200 - TAB2	rs7772697	ream_gene_va	3.00E-06	Near_to_perfect
7	68326659	20	G	A	68326660	3392	es per day in chronic obstr	7q11.22	68861673	C100419458 - LOC105375	rs10237067	itergenic_varia	3.00E-06	Perfect_to_near
7	112159177	20	G	A	112159178	1608	Osteoarthritis (hip)	7q31.1	112519123	LSMEM1 - NPM1P14	rs5009270	ream_gene_va	3.00E-06	Near_to_perfect
7	113741102	8	T	G	113741105	1787	IgG glycosylation	7q31.1	114101050	FOXP2	rs6466479	intron_variant	3.00E-06	Perfect_to_near
8	80985032	0	T	C	80985035	398	bolite levels (HVA/MHPG	8q21.13	80072800	TPD52	rs181166265	intron_variant	3.00E-06	New
8	86108146	22	C	A	86108149	2923	id beta peptide concentrat	8q21.2	85195914	E2F5	rs2403083	intron_variant	3.00E-06	Near_to_perfect
8	108281428	12	C	G	108281427	2374	IgG glycosylation	8q23.1	107269199	ANGPT1	rs6993449	intron_variant	3.00E-06	Near_to_perfect
8	118184780	8	C	T	118184783	1278	Type 2 diabetes	8q24.11	117172544	LOC105375716, SLC30A8	rs13266634	nissense_varia	3.00E-06	Near_to_perfect
8	142359551	8	A	G	142359550	1901	lar disorder and schizoph	8q24.3	141349451	LINC01300 - GPR20	rs7386474	stream_gene_v	3.00E-06	Perfect_to_near
9	92887226	22	C	T	92887231	888	Protein quantitative trait lo	9q22.2	90124949	OC286370 - LOC10537614	rs2081670	atory_region_v	3.00E-06	Near_to_perfect
9	100591458	18	C	T	100591463	3285	Thyroid hormone levels	9q22.33	97829181	PTCSC2	rs7045138	itergenic_varia	3.00E-06	Near_to_perfect
9	107531956	12	C	T	107531956	3164	Obesity-related traits	9q31.1	104769675	NIPSNAP3B	rs2472476	intron_variant	3.00E-06	Perfect_to_near
9	117778357	12	A	G	117778355	3820	Glucose homeostasis trait	9q33.1	115016076	TNFSF8 - TNC	rs1888221	stream_gene_v	3.00E-06	Near_to_perfect
9	126926105	8	T	C	126926107	1030	Cognitive performance	9q33.3	124163828	LHX2 - NEK6	rs2807580	itergenic_varia	3.00E-06	Perfect_to_near
9	136676014	18	T	C	136676014	1308	IgG glycosylation	9q34.2	133810892	VAV2	rs7021663	intron_variant	3.00E-06	Perfect_to_near
X	4596131	20	G	A	4596138	444	myotrophic lateral scleros	Xp22.32	4678097	OC101928201 - LOC34738	rs5916687	itergenic_varia	3.00E-06	Non_identical
10	9193893	26	C	A	9193901	1120	ozapine-induced cytotoxic	10p14	9151938	C105376400 - LOC101928	rs1149933	itergenic_varia	3.00E-06	Smaller
11	5625608	20	A	G	5625603	2695	IgG glycosylation	11p15.4	5604373	RIM6, TRIM5, TRIM6-TRIM	rs7108470	intron_variant	3.00E-06	Non_identical
12	26636378	18	A	G	26636386	4061	myotrophic lateral scleros	12p11.23	26483453	ITPR2	rs2306677	intron_variant	3.00E-06	Non_identical
12	101687053	22	C	T	101687043	1608	structure (hippocampal vo	12q23.2	101293265	UTP20	rs2290720	intron_variant	3.00E-06	Smaller
12	132701188	16	G	A	132701184	928	Body mass index	12q24.33	132216639	GALNT9 - GALNT9	rs11247009	intron_variant	3.00E-06	Bigger
12	132701188	16	G	A	132701184	928	Body mass index	12q24.33	132216639	GALNT9 - GALNT9	rs11247009	intron_variant	3.00E-06	Bigger
13	71595313	14	C	T	71595310	49	Type 2 diabetes	13q21.33	71021178	LINC00348	rs78319313	intron_variant	3.00E-06	Non_identical
13	92015982	14	G	A	92015977	2613	Height	13q31.3	91363723	MIR17HG - GPC5	rs8002779	itergenic_varia	3.00E-06	Smaller
14	62518352	18	C	G	62518348	911	Self-rated health	14q23.2	62051630	SYT16	rs6573416	intron_variant	3.00E-06	Non_identical
14	101690038	16	G	T	101690045	1080	ody mass index (interactio	14q32.31	101223708	C101929422 - LOC105370	rs8008758	itergenic_varia	3.00E-06	Non_identical

1	18244710	22	C	T	18244707	2638	Obesity-related traits	1p36.13	17918213	ACTL8 - LOC105376809	rs6686929	ntergenic_varia	3.00E-06	Non_identical
20	7752374	22	C	T	7752366	914	hemotherapy (neutropenia	20p12.3	7771719	.OC105372518 - SRSF10P2	rs6077251	ntergenic_varia	3.00E-06	Non_identical
22	26207904	16	C	T	26207910	210	Trans fatty acid levels	22q12.1	25811943	MYO18B	rs5752223	intron_variant	3.00E-06	Bigger
2	156579665	14	T	G	156579660	4262	l in Tripanosoma cruzi se	2q24.1	155723148	C105373700 - LOC105373	rs4664774	ntergenic_varia	3.00E-06	Smaller
2	205761824	20	A	C	205761832	737	Hippocampal volume	2q33.3	204897109	PARD3B	rs13023239	intron_variant	3.00E-06	Smaller
2	216860557	18	A	T	216860550	1808	ood trace element (Se leve	2q35	215995827	MREG	rs3770549	intron_variant	3.00E-06	Smaller
2	222801688	26	C	T	222801699	2681	Body mass index	2q36.1	221936980	LOC105373900	rs824931	intron_variant	3.00E-06	Non_identical
3	1839687	20	G	A	1839693	538	Total ventricular volume	3p26.3	1798009	RPL23AP38 - RPL21P17	rs10510217	ntergenic_varia	3.00E-06	Smaller
3	76261826	16	T	C	76261820	4709	lisorders (purging via subs	3p12.3	76212669	ROBO2	rs1516459	intron_variant	3.00E-06	Smaller
5	94148544	18	G	A	94148538	4542	Anorexia nervosa	5q15	94812833	MCTP1	rs469339	intron_variant	3.00E-06	Non_identical
6	30666675	22	C	G	30666669	317	IgG glycosylation	6p21.33	30698892	RPL7P4 - MDC1	rs9468811	ream_gene_va	3.00E-06	Non_identical
6	30666675	22	C	G	30666669	317	IgG glycosylation	6p21.33	30698892	RPL7P4 - MDC1	rs9468811	ream_gene_va	3.00E-06	Non_identical
6	161333941	10	G	C	161333937	886	Aging	6q26	160912905	LOC105378093	rs1247318	intron_variant	3.00E-06	Bigger
7	38128333	20	T	C	38128326	3221	Height	7p14.1	38088724	.OC105375236 - STARD3N	rs6959212	ntergenic_varia	3.00E-06	Non_identical
8	18258320	18	G	A	18258316	3391	nsulin resistance/respons	8p22	18400806	NAT2	rs1208	nissense_varia	3.00E-06	Non_identical
8	80103435	22	T	C	80103432	1869	Suicide in bipolar disorder	8q21.13	79191197	C105375912 - LOC105375	rs10448044	ntergenic_varia	3.00E-06	Smaller
10	12438785	18	G	A	12438783	1638	Obesity-related traits	10p13	12396784	CAMK1D	rs10906142	intron_variant	4.00E-06	Near_to_perfect
10	29487372	18	G	A	29487365	820	macokinetics (acute lymph	10p12.1	29198436	C105376471 - LOC105376	rs4387258	ntergenic_varia	4.00E-06	Smaller
10	71749674	20	T	C	71749675	69	l in Tripanosoma cruzi se	10q22.1	69989919	COL13A1 - LOC102723350	rs113808744	stream_gene_v	4.00E-06	Near_to_perfect
10	76808757	0	C	T	76808760	1295	Weight	10q22.2	75049002	DUPD1	rs7919006	intron_variant	4.00E-06	New
10	119193152	0	C	A	119193153	3131	Pit-and-Fissure caries	10q26.11	117433642	C105378501 - LOC105378	rs758569	intron_variant	4.00E-06	New
11	15355245	18	T	C	15355246	827	e cancer (gene x gene inte	11p15.2	15333700	INSC - LOC105376566	rs11605083	ntergenic_varia	4.00E-06	Near_to_perfect
11	21620949	18	A	T	21620948	2617	Cannabis use (initiation)	11p15.1	21599402	NELL1 - LOC102723370	rs1573535	ntergenic_varia	4.00E-06	Perfect_to_near
11	65260647	20	A	G	65260646	2512	Bone mineral density	11q13.1	65493175	LOC105369346 - MALAT1	rs600231	ream_gene_va	4.00E-06	Perfect_to_near
11	73477038	16	C	T	73477045	368	Obesity-related traits	11q13.4	73766000	RAB6A - MRPL48	rs1723838	ream_gene_va	4.00E-06	Near_to_perfect
11	93166731	20	T	G	93166731	4343	Pulmonary function decline	11q21	93433565	CCDC67	rs2658782	intron_variant	4.00E-06	Near_to_perfect
12	2783970	0	T	C	2783972	3378	Sleep quality	12p13.33	2674806	CACNA1C	rs2302729	intron_variant	4.00E-06	New
12	78772438	24	T	A	78772439	13	Hippocampal volume	12q21.2	78378659	C105369860 - LOC105369	rs148621641	intron_variant	4.00E-06	Near_to_perfect
13	44808177	18	C	A	44808174	1423	myotrophic lateral scleros	13q14.11	44234038	MIR8079 - LOC105370297	rs9533799	intron_variant	4.00E-06	Near_to_perfect
13	53605236	8	G	A	53605235	4052	Trans fatty acid levels	13q14.3	53031100	OLFM4	rs8000124	intron_variant	4.00E-06	Near_to_perfect
14	65747752	0	A	G	65747759	1476	duct disorder (symptom co	14q23.3	65281041	LOC105370536, PTBP1P	rs1256531	g_transcript_ex	4.00E-06	New
14	90301030	0	A	G	90301035	3253	Hepatocellular carcinoma	14q32.11	89834691	EFCAB11	rs12100561	intron_variant	4.00E-06	New
14	95107978	22	C	T	95107973	860	cial communication proble	14q32.13	94641636	SERPINA13P	rs4905226	g_transcript_ex	4.00E-06	Near_to_perfect
15	61702782	8	A	G	61702779	3565	Breast cancer (prognosis)	15q22.2	61410580	LOC105370847	rs3884558	intron_variant	4.00E-06	Perfect_to_near
15	75132317	8	A	G	75132319	3331	emotherapy (neutropenia/l	15q24.1	74839978	ULK3	rs936229	intron_variant	4.00E-06	Perfect_to_near
15	93564972	14	T	C	93564972	2786	IgG glycosylation	15q26.1	93021742	CHD2	rs7179432	rime_UTR_var	4.00E-06	Perfect_to_near
15	100297668	20	A	G	100297663	1933	Coronary artery calcificatio	15q26.3	99757458	LOC105371020	rs1993293	ntergenic_varia	4.00E-06	Near_to_perfect
16	3962620	20	A	G	3962620	2853	Neurofibrillary tangles	16p13.3	3912619	CREBBP - LOC102724927	rs12446940	ntergenic_varia	4.00E-06	Perfect_to_near
16	23888838	22	T	C	23888840	1317	Rheumatoid arthritis	16p12.2	23877519	PRKCB	rs7404928	intron_variant	4.00E-06	Perfect_to_near
16	50437529	20	C	T	50437528	448	zyme levels (alanine trans	16q12.1	50403617	C105371248 - LOC105371	rs9941219	ream_gene_va	4.00E-06	Near_to_perfect
16	82519227	8	C	T	82519228	3926	D-dimer levels	16q23.3	82485623	C100419639 - LOC101928	rs1991867	ntergenic_varia	4.00E-06	Perfect_to_near
18	46579969	12	C	T	46579970	2503	Dental caries	18q21.1	49053600	DYM	rs357894	intron_variant	4.00E-06	Perfect_to_near
19	22135272	8	A	C	22135270	3732	yopathy in Tripanosoma cr	19p12	21952468	LOC105372325 - ZNF208	rs2262909	intron_variant	4.00E-06	Absent
1	50937846	20	T	C	50937848	1880	ession and alcohol depenc	1p32.3	50472176	FAF1, LOC105378715	rs3827730	intron_variant	4.00E-06	Perfect_to_near
1	80734581	8	C	T	80734581	2569	Aging (time to event)	1p31.1	80268896	.OC105378810 - COX6A1P	rs11162963	ntergenic_varia	4.00E-06	Near_to_perfect
1	92194322	0	C	A	92194322	2757	Type 2 diabetes	1p22.1	91728765	TGFBR3	rs11165354	intron_variant	4.00E-06	New
1	109417678	0	C	A	109417679	828	Fat distribution (HIV)	1p13.3	108875057	SPATA42 - GPSM2	rs7523050	intron_variant	4.00E-06	New
1	163142553	20	G	A	163142555	1616	zyme levels (alanine trans	1q23.3	163172765	RGS5, LOC101928404	rs12035879	intron_variant	4.00E-06	Near_to_perfect
1	173301515	12	G	A	173301516	2394	Diabetic retinopathy	1q25.1	173332377	LOC100506023	rs1342038	atory_region_v	4.00E-06	Perfect_to_near
20	33775202	0	G	A	33775200	446	rophic lateral sclerosis (sp	20q11.22	35187397	PROCR	rs11167260	intron_variant	4.00E-06	New
21	32060492	0	A	G	32060490	2398	HDL cholesterol	21q22.11	30688172	(RTAP20-3 - LOC10537277	rs13046373	ntergenic_varia	4.00E-06	New
22	40820158	18	T	C	40820151	1048	Height	22q13.1	40424147	MKL1	rs5757949	intron_variant	4.00E-06	Smaller
2	26526427	18	T	G	26526419	506	Non-small cell lung cancer	2p23.3	26303551	LOC105374334	rs6753473	ream_gene_va	4.00E-06	Perfect_to_near
2	31590779	14	C	G	31590785	1368	Obesity-related traits	2p23.1	31367919	XDH	rs761926	intron_variant	4.00E-06	Non_identical
2	38277828	18	T	G	38277832	1391	function and prostate canc	2p22.2	38050689	RMDN2	rs6741148	intron_variant	4.00E-06	Near_to_perfect
2	104578533	8	T	C	104578533	878	tivity disorder (hyperactivi	2q12.1	103962075	C105373520 - LOC105373	rs1036736	intron_variant	4.00E-06	Perfect_to_near
2	105877872	12	C	T	105877870	54	Preeclampsia	2q12.1	105261413	TGFBRAP1	rs17636747	stream_gene_v	4.00E-06	Perfect_to_near
2	166943275	8	T	G	166943277	1045	Epilepsy (generalized)	2q24.3	166086767	LOC101929680, SCN1A	rs11890028	intron_variant	4.00E-06	Perfect_to_near
2	190771946	10	T	C	190771945	465	hol dependence (age at or	2q32.2	189907219	C2orf88, LOC105373795	rs62184315	intron_variant	4.00E-06	Perfect_to_near
2	232192306	0	C	T	232192305	1684	verse metabolic effects in	2q37.1	231327593	ARMC9	rs1669070	intron_variant	4.00E-06	New
2	234584324	0	T	C	234584324	2214	ion deficit hyperactivity dis	2q37.1	233675678	JGT1A9, UGT1A10, UGT1A	rs2602381	intron_variant	4.00E-06	New
2	238672418	22	G	C	238672425	170	Adiposity	2q37.3	237763782	LRRFIP1	rs11680012	nissense_varia	4.00E-06	Near_to_perfect
3	3588043	16	A	C	3588042	500	rophic lateral sclerosis (sp	3p26.2	3546358	C105376928 - LOC100130	rs17684824	intron_variant	4.00E-06	Near_to_perfect
3	54632551	20	T	A	54632547	991	lar disorder and schizophr	3p14.3	54598520	CACNA2D3	rs13064588	intron_variant	4.00E-06	Near_to_perfect
3	61413814	14	C	G	61413814	445	Major depressive disorder	3p14.2	61428140	LOC105377114	rs10514718	ntergenic_varia	4.00E-06	Near_to_perfect
3	72023695	16	G	A	72023700	563	Bilirubin levels	3p13	71974549	C105377156 - LOC105377	rs2135319	ntergenic_varia	4.00E-06	Perfect_to_near
3	142328916	20	C	G	142328919	1100	on in Tripanosoma cruzi s	3q23	142610077	PLS1, LOC105374136	rs9826463	intron_variant	4.00E-06	Perfect_to_near
4	37651153	8	A	G	37651155	1493	IgG glycosylation	4p14	37649533	RELL1	rs2292298	intron_variant	4.00E-06	Perfect_to_near
4	188617874	20	G	A	188617869	1007	Trans fatty acid levels	4q35.2	187696715	LOC105377603	rs9714717	ntergenic_varia	4.00E-06	Smaller
5	1320719	8	C	T	1320722	1682	Lung cancer	5p15.33	1320607	CLPTM1L	rs402710	g_transcript_ex	4.00E-06	Near_to_perfect
5	39902374	22	A	T	39902365	759	Urate levels	5p13.1	39902263	.OC101926940 - LINC0060	rs11954519	ntergenic_varia	4.00E-06	Smaller
5	107419544	20	C	T	107419548	1205	Body mass index	5q21.3	108083847	FBXL17	rs288232	intron_variant	4.00E-06	Perfect_to_near
5	134563163	22	C	T	134563157	3128	ne response to anthrax va	5q31.1	135227467	C5orf66	rs634308	intron_variant	4.00E-06	Smaller
5	153811488	8	A	G	153811490	1051	l disinhibition (generation	5q33.2	154431930	SAP30L-AS1	rs10037670	intron_variant	4.00E-06	Near_to_perfect
6	7106314	0	G	C	7106316	1126	l adipose tissue adjusted	6p24.3	7106083	LOC105374904 - RREB1	rs2842895	ream_gene_va	4.00E-06	New
6	7106314	0	G	C	7106316	1126	l adipose tissue adjusted	6p24.3	7106083	LOC105374904 - RREB1	rs2842895	ream_gene_va	4.00E-06	New
6	13268217	18	A	G	13268211	3370	HIV-1 viral setpoint	6p24.1	13267979	PHACTR1	rs202072	intron_variant	4.00E-06	Bigger
6	32381736	24	T	A	32381736	3250	p ratio adjusted for body r	6p21.32	32413959	BTNL2 - HLA-DRA	rs7759742	atory_region_v	4.00E-06	Near_to_perfect
6	142822216	16	T	C	142822217	244	Neutrophil count	6q24.2	142501080	ADGRG6 - LOC153910	rs9496398	ntergenic_varia	4.00E-06	Near_to_perfect
6	166571441	14	C	T	166571443	3583	e tissue/subcutaneous adi	6q27	166157955	T	rs1056053	rime_UTR_var	4.00E-06	Near_to_perfect

7	7268430	8	C	T	7268431	2859	Multiple sclerosis (severity	7p21.3	7228800	C1GALT1	rs10259085	intron_variant	4.00E-06	Perfect_to_near
7	8142921	14	T	C	8142927	701	Cognitive function	7p21.3	8103297	LOC105375142 - ICA1	rs7791362	ntergenic_variar	4.00E-06	Bigger
7	22027207	0	C	T	22027212	1233	ate levels in obese individu	7p15.3	21987594	CDCA7L - LOC102724143	rs7783529	ntergenic_variar	4.00E-06	New
7	37746561	20	C	T	37746569	2641	Periodontitis	7p14.1	37706967	GPR141	rs2392510	intron_variant	4.00E-06	Non_identical
7	80799832	28	T	G	80799834	4939	Political ideology	7q21.11	81170518	SEMA3C - LOC105369146	rs500454	ream_gene_va	4.00E-06	Perfect_to_near
7	113741102	8	T	G	113741105	1787	IgG glycosylation	7q31.1	114101050	FOXP2	rs6466479	intron_variant	4.00E-06	Perfect_to_near
7	114629284	8	G	A	114629285	1128	Obesity	7q31.1	114989231	MDFIC	rs7784447	intron_variant	4.00E-06	Perfect_to_near
7	152387869	0	T	G	152387869	3850	nitic acid (16:0) plasma le	7q36.1	152690784	XRCC2 - ACTR3B	rs10234749	ntergenic_variar	4.00E-06	New
8	60130297	14	C	T	60130296	2553	RR interval (heart rate)	8q12.1	59217737	.OC100505501 - NUDT15P	rs3110127	ntergenic_variar	4.00E-06	Perfect_to_near
8	115578306	8	T	G	115578307	1622	stress disorder (asjusted f	8q23.3	114566078	LOC105375710 - TRPS1	rs7014900	ntergenic_variar	4.00E-06	Near_to_perfect
10	115814402	24	C	T	115814392	2238	Adiponectin levels	10q25.3	114054633	ADRB1 - CCDC186	rs10885531	ntergenic_variar	4.00E-06	Smaller
11	123956472	16	A	G	123956466	951	Obesity-related traits	11q24.2	124085759	LOC105369545	rs1893767	atory_region_v	4.00E-06	Smaller
12	43160817	22	C	T	43160825	908	Panic disorder	12q12	42767023	C105369739 - LOC105369	rs2731006	ntergenic_variar	4.00E-06	Non_identical
12	53102190	18	C	A	53102196	761	Electrodermal activity	12q13.13	52708412	KRT77 - LOC400036	rs678069	ream_gene_va	4.00E-06	Non_identical
12	132701188	16	G	A	132701184	928	Body mass index	12q24.33	132216639	GALNT9 - GALNT9	rs11247009	intron_variant	4.00E-06	Bigger
13	70658398	28	T	C	70658404	2008	Verbal declarative memory	13q21.33	70084272	KLHL1	rs1550546	intron_variant	4.00E-06	Smaller
14	57822220	18	G	A	57822216	2679	Alcohol consumption	14q22.3	57355498	LOC105370516 - NAA30	rs7144649	g_transcript_ex	4.00E-06	Non_identical
14	81523131	26	T	C	81523128	496	nerve measurement (rim	14q31.1	81056784	TSHR, LOC101928462	rs17111394	intron_variant	4.00E-06	Non_identical
15	37449625	14	G	A	37449619	325	Response to amphetamine	15q14	37157418	MEIS2 - LOC105370772	rs17439560	ntergenic_variar	4.00E-06	Bigger
18	66214689	22	C	T	66214696	936	Suicide in bipolar disorder	18q22.1	68547459	LOC105372175 - TMX3	rs7244261	ntergenic_variar	4.00E-06	Non_identical
1	209334226	22	G	A	209334217	2986	Response to amphetamine	1q32.2	209160872	C100509303 - LOC105372	rs4382726	ntergenic_variar	4.00E-06	Smaller
20	45497717	20	G	A	45497723	863	e tissue/subcutaneous adi	20q13.12	46869084	SLC2A10 - EYA2	rs6124878	ream_gene_va	4.00E-06	Non_identical
2	27587727	20	G	A	27587724	37	IgG glycosylation	2p23.3	27364857	LOC105374363, EIF2B4	rs1058065	nonymous_vari	4.00E-06	Non_identical
2	224274625	22	G	A	224274622	224	eoporosis-related phenoty	2q36.1	223409904	C105373906 - LOC105373	rs16864755	ntergenic_variar	4.00E-06	Smaller
3	60289848	16	G	A	60289842	456	Asperger disorder	3p14.2	60304113	FHIT	rs10510837	intron_variant	4.00E-06	Smaller
5	92567545	18	A	G	92567539	479	Natriuretic peptide levels	5q15	93231833	LOC105379083 - POLD2P1	rs4869419	ntergenic_variar	4.00E-06	Non_identical
5	127139622	14	G	A	127139616	570	tivity disorder (hyperactivit	5q23.2	127803924	LINC01183	rs1515641	intron_variant	4.00E-06	Non_identical
6	31802458	18	C	G	31802465	616	IgG glycosylation	6p21.33	31834688	HSPA1B - C6orf48	rs4711279	rime_UTR_var	4.00E-06	Non_identical
6	95967418	18	T	C	95967412	4337	l disinhibition (generation	6q16.1	95519536	LOC105377903	rs2380220	atory_region_v	4.00E-06	Non_identical
7	12193435	12	G	A	12193432	1263	Anorexia nervosa	7p21.3	12153806	THSD7A - TMEM106B	rs114945094	ntergenic_variar	4.00E-06	Smaller
7	21861106	16	C	T	21861112	4306	ognitive decline (age-relate	7p15.3	21821494	DNAH11	rs2390593	intron_variant	4.00E-06	Non_identical
8	10022943	20	G	T	10022938	2083	Schizophrenia	8p23.1	10165428	MSRA	rs7017212	intron_variant	4.00E-06	Non_identical
9	94395864	14	A	T	94395860	692	Type 2 diabetes	9q22.31	91633578	.OC101927916 - MIR3910-1	rs1873747	intron_variant	4.00E-06	Non_identical
X	33784068	20	A	T	33784063	435	Tuberculosis	Xp21.1	33765946	LOC105373153	rs5928363	intron_variant	4.00E-06	Non_identical
10	27693307	22	G	A	27693305	4799	ulin-related traits (interactio	10p12.1	27404376	PTCHD3	rs1334893	intron_variant	5.00E-06	Perfect_to_near
10	50500457	8	C	G	50500459	190	c acid (18:1n-9) plasma le	10q11.23	49292414	C10orf128 - C10orf71-AS1	rs17774576	stream_gene_v	5.00E-06	Near_to_perfect
10	97295578	24	A	G	97295589	310	IgG glycosylation	10q24.1	95535832	SORBS1	rs12772243	intron_variant	5.00E-06	Smaller
11	30223574	8	G	A	30223574	3695	Body mass index	11p14.1	30202027	LOC105376607	rs607987	ntergenic_variar	5.00E-06	Near_to_perfect
11	80854373	8	C	A	80854375	491	HIV-1 viral setpoint	11q14.1	81143332	LOC101928964	rs1357339	ntergenic_variar	5.00E-06	Near_to_perfect
12	43434284	0	C	A	43434282	4662	Obesity-related traits	12q12	43040479	C105369739 - LOC105369	rs1167125	ntergenic_variar	5.00E-06	New
13	44702869	16	T	A	44702869	1579	assessment of insulin res	13q14.11	44128733	.OC105370183, SMIM2-AS1	rs9525916	intron_variant	5.00E-06	Perfect_to_near
13	51724661	24	T	C	51724664	3250	IgG glycosylation	13q14.3	51150528	LINC00371	rs7325564	intron_variant	5.00E-06	Perfect_to_near
13	92501662	34	C	T	92501664	606	Obesity-related traits	13q31.3	91849410	GPC5	rs7328464	intron_variant	5.00E-06	Near_to_perfect
14	51323740	16	G	A	51323742	2155	Dietary macronutrient intak	14q22.1	50857024	LOC105370491	rs8019546	intron_variant	5.00E-06	Perfect_to_near
15	40673462	8	T	C	40673465	315	Heschl's gyrus morphology	15q15.1	40381264	DISP2 - KNSTRN	rs78153629	ream_gene_va	5.00E-06	Absent
15	61702782	8	A	G	61702779	3565	Response to amphetamine	15q22.2	61410580	LOC105370847	rs3884558	intron_variant	5.00E-06	Perfect_to_near
16	10663629	14	T	G	10663627	3864	Eating disorders	16p13.13	10569770	EMP2	rs2221433	intron_variant	5.00E-06	Perfect_to_near
16	24810684	16	C	T	24810681	112	es (dietary heme iron intal	16p12.1	24799360	TNRC6A	rs17177078	intron_variant	5.00E-06	Near_to_perfect
16	82326815	24	G	A	82326805	338	Obesity-related traits	16q23.3	82293200	C105371365 - LOC100419	rs11863065	ntergenic_variar	5.00E-06	Smaller
17	43824359	24	T	C	43824360	1317	ner's disease in APOE e4-	17q21.31	45746994	MGC57346-CRHR1	rs7207400	intron_variant	5.00E-06	Perfect_to_near
18	5857090	20	C	T	5857091	599	Immunoglobulin A	18p11.31	5857092	MIR3976 - TMEM200C	rs11662763	intron_variant	5.00E-06	Perfect_to_near
18	63031226	22	G	T	63031236	3603	Atrioventricular conduction	18q22.1	65364000	C105372168 - LOC105372	rs470490	ntergenic_variar	5.00E-06	Non_identical
19	19829102	18	T	G	19829101	938	Toenail selenium levels	19p13.11	19718292	ZNF14	rs2163813	intron_variant	5.00E-06	Perfect_to_near
19	46974001	16	C	T	46974003	337	orectal cancer (diet interac	19q13.32	46470746	PNMAL1	rs7248888	nissense_variar	5.00E-06	Near_to_perfect
1	8395556	26	G	T	8395560	773	Breast cancer	1p36.23	8335500	SLC45A1	, rs12711517,	nissense_variar	5.00E-06	Near_to_perfect
1	8395556	26	G	T	8395560	773	Breast cancer	1p36.23	8335500	SLC45A1	528987, rs7535	nissense_variar	5.00E-06	Near_to_perfect
1	25402623	8	C	T	25402624	1761	IgG glycosylation	1p36.11	25076133	OC105376879 - LOC39102	rs10903027	intron_variant	5.00E-06	Near_to_perfect
1	109417678	0	C	A	109417679	828	Fat distribution (HIV)	1p13.3	108875057	SPATA42 - GPSM2	rs7523050	intron_variant	5.00E-06	New
1	162319528	18	A	G	162319524	413	Word reading	1q23.3	162349734	NOS1AP	rs11577628	intron_variant	5.00E-06	Non_identical
1	192995211	24	C	T	192995213	80	Cannabis dependence	1q31.2	193026083	UCHL5	rs9427573	intron_variant	5.00E-06	Perfect_to_near
1	197306099	8	C	T	197306099	3708	ritis in systemic lupus ery	1q31.3	197336969	CRB1	rs2786111	intron_variant	5.00E-06	Perfect_to_near
20	20634105	18	T	C	20634106	383	IgG glycosylation	20p11.23	20653462	RALGAPA2	rs6132333	intron_variant	5.00E-06	Perfect_to_near
20	25240186	16	G	T	25240189	1442	Calcium levels	20p11.21	25259553	PYGB	rs2281558	intron_variant	5.00E-06	Near_to_perfect
22	16504402	8	C	T	16504399	3949	Celiac disease	22q11.1	15473564	C105379428 - LOC105372	rs4911642	ntergenic_variar	5.00E-06	Near_to_perfect
2	26112516	0	T	A	26112518	2691	yeloma and monoclonal ga	2p23.3	25889649	ASXL2 - KIF3C	rs12986445	ream_gene_va	5.00E-06	New
2	38916967	8	A	T	38916970	919	brain serotonin transporte	2p22.1	38689828	GALM	rs6741892	nissense_variar	5.00E-06	Near_to_perfect
2	52713804	8	T	G	52713807	665	Cardiac Troponin-T levels	2p16.3	52486669	OC730100 - LOC10537459	rs1526687	ntergenic_variar	5.00E-06	Perfect_to_near
2	61772254	8	A	G	61772257	2908	Tuberculosis	2p15	61545122	XPO1 - FAM161A	rs6545883	ntergenic_variar	5.00E-06	Perfect_to_near
2	65027619	18	T	C	65027624	1105	Visceral fat	2p14	64800490	C105374776 - LOC101927	rs11683197	atory_region_v	5.00E-06	Perfect_to_near
2	70869471	8	A	G	70869473	949	Mammographic density	2p13.3	70642341	TGFA - ADD2	rs6733938	intron_variant	5.00E-06	Near_to_perfect
2	141226317	18	T	A	141226309	539	Periodontitis (CDC/AAP)	2q22.1	140468740	LRP1B	rs72899866	intron_variant	5.00E-06	Near_to_perfect
2	231230169	0	C	T	231230171	2015	Nicotine use	2q37.1	230365456	SP140L	rs6712333	intron_variant	5.00E-06	New
2	232176540	12	G	A	232176541	1090	Multiple myeloma	2q37.1	231311828	ARMC9	rs7580717	intron_variant	5.00E-06	Near_to_perfect
3	21706370	24	G	A	21706369	447	Bipolar disorder	3p24.3	21664877	ZNF385D	rs3821396	intron_variant	5.00E-06	Near_to_perfect
3	21719248	12	C	T	21719246	3381	Partial epilepsies	3p24.3	21677754	ZNF385D	rs1490157	intron_variant	5.00E-06	Near_to_perfect
3	62112137	16	G	A	62112141	560	bolite levels (HVA/5-HIAA	3p14.2	62126467	PTPRG, LOC105377118	rs35593266	intron_variant	5.00E-06	Near_to_perfect
3	80368459	20	T	C	80368463	288	Migraine	3p12.2	80319313	ROBO1 - LOC105377177	rs10511112	ntergenic_variar	5.00E-06	Near_to_perfect
4	13794412	22	G	A	13794416	885	factors and hematological	4p15.33	13792792	.OC101929048, LINC01182	rs10489087	intron_variant	5.00E-06	Near_to_perfect
4	23588463	0	G	A	23588462	664	verse metabolic effects in	4p15.2	23586839	C105374525 - LOC105374	rs1511453	intron_variant	5.00E-06	New
4	88919103	18	G	A	88919106	2745	Caffeine consumption	4q22.1	87997954	SPP1 - LOC105377325	rs2725236	ream_gene_va	5.00E-06	Near_to_perfect

4	111734131	12	T	C	111734136	1774	Coronary heart disease	4q25	110812980	PITX2 - MIR297	rs3853444	itergenic_variai	5.00E-06	Near_to_perfect
4	128466695	12	A	G	128466700	2492	IgG glycosylation	4q28.1	127545545	C101060158 - LOC105377	rs2090104	itergenic_variai	5.00E-06	Near_to_perfect
4	154703587	20	T	C	154703596	945	ardiographic conduction m	4q31.3	153782444	SFRP2	rs17030434	intron_variant	5.00E-06	Non_identical
5	127875729	30	A	G	127875736	138	l in Tripanosoma cruzi sei	5q23.3	128540043	LOC105379168	rs186263310	intron_variant	5.00E-06	Non_identical
5	170720735	18	C	G	170720733	268	Visceral fat	5q35.1	171293729	RANBP17	rs2278255	intron_variant	5.00E-06	Perfect_to_near
6	16343056	16	A	G	16343056	1131	Gambling	6p22.3	16342825	ATXN1	rs9383153	intron_variant	5.00E-06	Perfect_to_near
6	32381736	24	T	A	32381736	3250	ip ratio adjusted for body r	6p21.32	32413959	BTNL2 - HLA-DRA	rs7759742	atory_region_v	5.00E-06	Near_to_perfect
6	44928955	8	T	C	44928953	106	Number of pregnancies	6p21.1	44961216	SUPT3H	rs16872971	intron_variant	5.00E-06	Near_to_perfect
6	90657783	0	T	C	90657783	2861	IgG glycosylation	6q15	89948064	BACH2	rs404256	intron_variant	5.00E-06	New
6	97922186	18	T	C	97922184	2016	Body mass index	6q16.1	97474308	LOC101927314	rs200810	intron_variant	5.00E-06	Perfect_to_near
7	28812494	16	A	G	28812493	3503	ety in major depressive dis	7p15.1	28772876	CREB5	rs10238623	intron_variant	5.00E-06	Near_to_perfect
7	49469002	18	C	T	49469003	1067	hyroid-stimulating hormon	7p12.2	49429407	CDC14C - VWC2	rs740083	itergenic_variai	5.00E-06	Near_to_perfect
7	107443382	22	C	T	107443386	924	IgG glycosylation	7q31.1	107802941	SLC26A3	rs4730268	intron_variant	5.00E-06	Near_to_perfect
7	107479515	20	C	A	107479519	1313	Ulcerative colitis	7q31.1	107839074	SLC26A3 - LOC105375444	rs4730273	itergenic_variai	5.00E-06	Near_to_perfect
7	111698107	12	T	C	111698108	3710	mass index (change over	7q31.1	112058053	DOCK4	rs634010	intron_variant	5.00E-06	Perfect_to_near
7	154072011	24	T	C	154072020	2891	Tardive dyskinesia	7q36.2	154374935	DPP6	rs6977820	intron_variant	5.00E-06	Absent
8	5764945	0	G	A	5764942	1739	Dietary macronutrient intak	8p23.2	5907420	LOC105377795	rs2840445	stream_gene_v	5.00E-06	New
8	31567004	16	A	T	31567006	3794	Cardiac Troponin-T levels	8p12	31709490	NRG1	rs4733271	intron_variant	5.00E-06	Perfect_to_near
8	57179018	8	C	T	57179020	781	Height	8q12.1	56266461	CHCHD7 - SDR16C5	rs7815788	itergenic_variai	5.00E-06	Near_to_perfect
8	61105199	0	T	C	61105199	2536	Obesity-related traits	8q12.1	60192640	CA8	rs10098647	intron_variant	5.00E-06	New
9	27533990	0	C	A	27533984	2296	Heart failure	9p21.2	27533986	MOB3B - C9orf72	rs10812610	ream_gene_va	5.00E-06	New
9	30751217	0	T	C	30751220	767	hol dependence (age at oi	9p21.1	30751222	RBMXP2 - KRT18P66	rs10969853	itergenic_variai	5.00E-06	New
9	37916407	8	T	C	37916408	796	evels (Dihydroxy docosatr	9p13.1	37916411	SHB	rs3747547	intron_variant	5.00E-06	Perfect_to_near
9	105573035	18	G	A	105573037	1804	drome or obsessive-comp	9q31.1	102810755	LINC00587 - CYLC2	rs7848024	itergenic_variai	5.00E-06	Perfect_to_near
9	139265593	18	G	A	139265596	6	Obesity-related traits	9q34.3	136371144	CARD9	rs34971035	ce_region_vari	5.00E-06	Perfect_to_near
X	139166681	12	C	T	139166683	957	lar disorder and schizophi	Xq27.1	140084524	LOC728660 - LOC389895	rs5907577	ream_gene_va	5.00E-06	Near_to_perfect
10	80953141	24	A	C	80953136	3190	disorder, bipolar disorder, i	10q22.3	79193379	ZMIZ1	rs703970	intron_variant	5.00E-06	Smaller
11	63666948	18	C	T	63666944	414	nent in attention-deficit/hy	11q13.1	63899472	MARK2	rs12099085	intron_variant	5.00E-06	Non_identical
12	90609021	14	G	T	90609017	3255	vWF and FVIII levels	12q21.33	90215240	C105369890 - LOC105369	rs10745527	itergenic_variai	5.00E-06	Non_identical
14	38641713	18	T	C	38641706	2010	Lewy body disease	14q21.1	38172501	LOC105370456 - SSTR1	rs2803904	intron_variant	5.00E-06	Smaller
15	96708284	20	T	C	96708291	1565	hormone-binding globulin l	15q26.2	96165062	NR2F2-AS1	rs8023580	intron_variant	5.00E-06	Bigger
16	4257319	22	C	T	4257313	103	Obesity-related traits	16p13.3	4207312	SRL	rs75825892	nissense_variai	5.00E-06	Smaller
1	155098911	16	G	A	155098905	47	Obesity-related traits	1q22	155126429	EFNA3 - EFNA1	rs11264330	ream_gene_va	5.00E-06	Smaller
20	36977975	16	G	A	36977970	717	IgG glycosylation	20q11.23	38349567	LBP	rs1739654	nonymous_vari	5.00E-06	Non_identical
2	75790497	22	T	C	75790489	303	ce of antiphospholipid ant	2p12	75563363	EVA1A	rs17011455	intron_variant	5.00E-06	Bigger
2	198143996	16	A	G	198144002	1719	a levels in systemic lupus	2q33.1	197279278	ANKRD44-IT1, ANKRD44	rs1429411	intron_variant	5.00E-06	Bigger
3	150935365	20	A	G	150935371	275	Heschl's gyrus morphology	3q25.1	151217583	P2RY14, MED12L	rs1554120	intron_variant	5.00E-06	Smaller
4	76207573	18	A	C	76207570	1376	Classic bladder exstrophy	4q13.3	75282360	OC100506253 - LOC44102	rs7689350	intron_variant	5.00E-06	Non_identical
4	88707075	20	C	T	88707081	1530	Sitting height ratio	4q22.1	87785929	CHCHD2P7	rs13136331	g_transcript_ex	5.00E-06	Non_identical
5	167500453	20	T	A	167500460	542	Uric acid levels	5q34	168073455	TENM2	rs13358864	intron_variant	5.00E-06	Non_identical
6	156345134	28	T	C	156345146	2978	Trans fatty acid levels	6q25.3	156024012	LOC101928923	rs1449672	itergenic_variai	5.00E-06	Smaller
7	105222444	20	C	A	105222451	521	sorder (body mass index ir	7q22.3	105582004	YBX1P2	rs116979167	intron_variant	5.00E-06	Non_identical
8	77611617	18	A	G	77611625	1967	Menarche (age at onset)	8q21.13	76699390	ZFHX4	rs4735738	intron_variant	5.00E-06	Non_identical
8	87721353	20	A	G	87721349	3703	statin treatment (PCSK9 p	8q21.3	86709121	CNGB3	rs7814749	intron_variant	5.00E-06	Non_identical
9	28414346	18	A	G	28414339	1020	Body mass index	9p21.1	28414341	LINGO2	rs10968576	intron_variant	5.00E-06	Smaller
10	1630823	24	C	T	1630821	3612	Emphysema-related traits	10p15.3	1588626	ADARB2	rs2999399	intron_variant	6.00E-06	Perfect_to_near
10	18759628	10	T	C	18759629	3317	Protein quantitative trait loc	10p12.31	18470700	CACNB2	rs7076247	intron_variant	6.00E-06	Near_to_perfect
10	21436221	0	C	A	21436222	303	o radiotherapy in cancer (l	10p12.31	21147293	NEBL	rs12243039	intron_variant	6.00E-06	New
10	127699926	22	T	C	127699936	1663	Visceral fat	10q26.2	126011367	FANK1 - ADAM12	rs10901513	stream_gene_v	6.00E-06	Smaller
11	7701499	14	G	C	7701503	327	Blood pressure	11p15.4	7680272	OVCH2, LOC105376534	rs11041530	ream_gene_va	6.00E-06	Near_to_perfect
11	13345589	24	G	A	13345593	2346	Body mass index	11p15.3	13324046	ARNTL	rs9633835	intron_variant	6.00E-06	Near_to_perfect
11	15620587	8	A	G	15620586	506	Obesity-related traits	11p15.2	15599040	OC105376569, LOC105376	rs17439299	intron_variant	6.00E-06	Near_to_perfect
11	85838803	14	G	A	85838808	2810	chosis and Alzheimer's dis	11q14.2	86127766	PICALM - FNTAP1	rs10792830	atory_region_v	6.00E-06	Near_to_perfect
12	60735169	0	C	T	60735173	4869	Obesity-related traits	12q14.1	60341392	LOC100996696 - PGBD3P1	rs7974425	stream_gene_v	6.00E-06	New
12	60735169	0	C	T	60735173	4869	Obesity-related traits	12q14.1	60341392	LOC100996696 - PGBD3P1	rs7974425	stream_gene_v	6.00E-06	New
12	114359264	20	G	T	114359265	400	rophic lateral sclerosis (sp	12q24.21	113921460	RBM19	rs3782455	intron_variant	6.00E-06	Near_to_perfect
13	32972625	8	A	T	32972626	22	Breast cancer	13q13.1	32398489	BRCA2	rs11571833	stop_gained	6.00E-06	Near_to_perfect
13	38260398	0	C	T	38260408	2720	drome or obsessive-comp	13q13.3	37686271	TRPC4	rs6563569	intron_variant	6.00E-06	New
13	71249530	26	T	A	71249529	1	tus in Tripanosoma cruzi s	13q21.33	70675397	ATXN8OS - LOC105370255	rs186967887	itergenic_variai	6.00E-06	Near_to_perfect
14	48054161	12	A	G	48054159	2819	IgG glycosylation	14q21.3	47584956	MDGA2	rs4143912	intron_variant	6.00E-06	Perfect_to_near
14	80587140	14	T	C	80587141	3367	(forced expiratory volume	14q31.1	80120798	C105370590 - LOC105370	rs10220309	itergenic_variai	6.00E-06	Perfect_to_near
14	90396568	18	T	A	90396574	1352	verse metabolic effects in l	14q32.11	89930230	EFCAB11	rs7147996	intron_variant	6.00E-06	Bigger
15	80219590	16	G	A	80219593	64	Periodontitis (Mean PAL)	15q25.1	79927251	ST20-AS1 - FDPSP9	rs75598935	ream_gene_va	6.00E-06	Near_to_perfect
15	88723714	18	T	G	88723712	1215	disorder, bipolar disorder, i	15q25.3	88180481	NTRK3	rs1104918	intron_variant	6.00E-06	Perfect_to_near
16	10630772	24	C	A	10630777	4101	Metabolite levels (MHPG)	16p13.13	10536920	EMP2	rs6498068	intron_variant	6.00E-06	Near_to_perfect
16	63955449	8	G	A	63955447	1987	hyperactivity disorder and	16q21	63921543	OC105371309 - LOC72921	rs1381102	itergenic_variai	6.00E-06	Perfect_to_near
17	30877662	10	C	T	30877658	3513	Pancreatic cancer	17q11.2	32550640	MYO1D	rs225190	intron_variant	6.00E-06	Near_to_perfect
18	44736332	20	C	T	44736336	1889	Educational attainment	18q21.1	47209965	SKOR2	rs2635047	stream_gene_v	6.00E-06	Perfect_to_near
18	65483807	18	C	G	65483812	97	sorder (body mass index ir	18q22.1	67816575	LOC643542	rs11661646	intron_variant	6.00E-06	Perfect_to_near
18	71476134	8	T	A	71476133	454	Coronary artery calcificatio	18q22.3	73808898	LOC105372191 - FBXO15	rs17088339	itergenic_variai	6.00E-06	Near_to_perfect
19	51726610	24	G	A	51726613	3176	Alzheimer's disease	19q13.41	51223357	CD33	rs3826656	ream_gene_va	6.00E-06	Near_to_perfect
1	26884865	18	G	A	26884864	2478	Glucose homeostasis traits	1p36.11	26558373	RPS6KA1	rs3790645	intron_variant	6.00E-06	Perfect_to_near
1	71073918	0	A	G	71073921	964	constitution type in type 2 c	1p31.1	70608238	OC391048 - LOC10537879	rs1932064	itergenic_variai	6.00E-06	New
1	95029579	18	G	T	95029581	2181	on in Tripanosoma cruzi se	1p21.3	94564025	F3 - LOC105378861	rs1146509	itergenic_variai	6.00E-06	Near_to_perfect
1	184677464	18	G	A	184677464	3265	Obesity-related traits	1q25.3	184708330	EDEM3	rs3736757	nonymous_vari	6.00E-06	Near_to_perfect
20	7552506	16	C	T	7552504	1219	factors and hematological	20p12.3	7571857	MIR8062 - LOC105372518	rs6108011	atory_region_v	6.00E-06	Near_to_perfect
20	17566063	0	A	T	17566069	187	ce of antiphospholipid ant	20p12.1	17585424	DSTN	rs17791782	intron_variant	6.00E-06	New
21	44448711	22	C	T	44448718	802	formation processing spee	21q22.3	43028608	PKNOX1	rs2839627	ce_region_vari	6.00E-06	Near_to_perfect
22	37258507	18	T	C	37258503	1767	Atopic dermatitis	22q12.3	36862461	NCF4	rs4821544	intron_variant	6.00E-06	Non_identical
2	6134935	20	T	C	6134940	1079	HIV-1 control	2p25.2	5994808	OC400940 - LOC10537340	rs1405262	intron_variant	6.00E-06	Near_to_perfect

2	25887555	16	A	G	25887558	1180	LDL cholesterol	2p23.3	25664689	DTNB	rs11684202	intron_variant	6.00E-06	Perfect_to_near
2	77223937	22	C	T	77223938	2793	IgG glycosylation	2p12	76996812	LOC101927907, LRRTM4	rs1470506	intron_variant	6.00E-06	Near_to_perfect
2	103512292	16	T	A	103512292	983	verse metabolic effects in	2q12.1	102895834	C105373518 - LOC105373	rs13402330	ream_gene_va	6.00E-06	Perfect_to_near
2	155002847	18	T	C	155002847	742	Obesity-related traits	2q24.1	154146334	GALNT13	rs16836124	intron_variant	6.00E-06	Near_to_perfect
2	170624218	8	C	T	170624221	1268	Obesity-related traits	2q31.1	169767711	PHOSPHO2-KLHL23 - SSB	rs2114646	ice_donor_vari	6.00E-06	Perfect_to_near
2	174326852	18	C	T	174326845	544	rement (high sodium and	2q31.1	173462117	OC105373744 - LOC64399	rs10930597	ntergenic_varia	6.00E-06	Smaller
2	227092798	24	A	G	227092802	3759	Body mass index	2q36.3	226228086	OC646736 - LOC10537391	rs2176040	ntergenic_varia	6.00E-06	Perfect_to_near
2	236795336	0	A	C	236795343	2176	Schizophrenia	2q37.2	235886699	AGAP1	rs13025591	intron_variant	6.00E-06	New
2	239306263	20	A	C	239306268	214	Iris characteristics	2q37.3	238397627	TRAF3IP1	rs3739070	nissense_varia	6.00E-06	Near_to_perfect
3	3199636	12	A	C	3199635	3623	White matter integrity	3p26.2	3157951	CRBN	rs1669338	intron_variant	6.00E-06	Near_to_perfect
3	71414042	8	A	G	71414045	2363	IgG glycosylation	3p13	71364894	FOXP1	rs13072512	intron_variant	6.00E-06	Perfect_to_near
3	106734133	20	T	C	106734131	2614	coustic startle blink respon	3q13.12	107015284	OC101929534 - LOC34459	rs2399126	intron_variant	6.00E-06	Perfect_to_near
3	112681581	12	A	G	112681585	4418	nomic and political prefere	3q13.2	112962738	CD200R1	rs1488193	intron_variant	6.00E-06	Near_to_perfect
3	142982896	26	A	G	142982899	1421	ion deficit hyperactivity dis	3q24	143264057	PBX2P1 - SLC9A9	rs9810857	stream_gene_v	6.00E-06	Near_to_perfect
3	165125158	28	T	C	165125159	1299	Response to amphetamine	3q26.1	165407371	LINC01322	rs9878522	intron_variant	6.00E-06	Near_to_perfect
4	735149	8	G	A	735150	972	factors and hematological	4p16.3	741362	PCGF3	rs4234853	intron_variant	6.00E-06	Near_to_perfect
4	14144207	20	A	G	14144208	1901	Obesity-related traits	4p15.33	14142584	LINC01085 - LINC00504	rs6844339	intron_variant	6.00E-06	Near_to_perfect
4	183137395	18	T	C	183137398	2566	Schizophrenia	4q34.3	182216245	LOC105377572	rs2726807	intron_variant	6.00E-06	Perfect_to_near
5	62384698	14	C	T	62384699	413	ronchopulmonary dysplas	5q12.1	63088872	ISCA1P1 - LOC105378999	rs71627250	ntergenic_varia	6.00E-06	Near_to_perfect
5	79099456	20	A	C	79099464	990	lar disorder and schizoph	5q14.1	79803641	LOC102724557	rs7735699	intron_variant	6.00E-06	Non_identical
5	98781100	16	C	T	98781103	1883	factors and hematological	5q21.1	99445399	C102724810 - LOC100652	rs1829883	ntergenic_varia	6.00E-06	Near_to_perfect
5	159849581	14	A	G	159849586	807	Insulin-related traits	5q33.3	160422579	PTTG1	rs1895320	rime_UTR_var	6.00E-06	Perfect_to_near
6	542416	22	T	A	542416	1742	Psoriasis	6p25.3	542416	EXOC2	rs3799296	intron_variant	6.00E-06	Perfect_to_near
6	53307694	24	C	T	53307694	2633	ncephalographic traits in a	6p12.1	53442896	NANOGP3 - LOC10537509	rs9395865	ntergenic_varia	6.00E-06	Near_to_perfect
6	130216511	26	A	G	130216510	2699	Renal sinus fat	6q22.33	129895365	LOC105377999	rs9375674	atory_region_v	6.00E-06	Near_to_perfect
6	130358427	10	G	A	130358428	2677	Height	6q23.1	130037283	L3MBTL3	rs6899976	intron_variant	6.00E-06	Near_to_perfect
6	157216038	16	T	C	157216039	3485	nosis in coronary artery by	6q25.3	156894905	ARID1B	rs184074	intron_variant	6.00E-06	Near_to_perfect
7	12391315	12	G	T	12391319	3139	se to taxane treatment (pla	7p21.3	12351693	VWDE	rs6967385	nissense_varia	6.00E-06	Near_to_perfect
7	13560215	16	C	A	13560221	560	Word reading	7p21.3	13520596	C105375159 - LOC10537	rs1357978	intron_variant	6.00E-06	Non_identical
7	56139501	0	A	T	56139505	596	l in Tripanosoma cruzi se	7p11.2	56071812	SUMF2	rs7806994	intron_variant	6.00E-06	New
7	70067681	8	G	A	70067679	1703	Body mass index	7q11.22	70602693	AUTS2	rs38313	intron_variant	6.00E-06	Near_to_perfect
7	113741102	8	T	G	113741105	1787	IgG glycosylation	7q31.1	114101050	FOXP2	rs6466479	intron_variant	6.00E-06	Perfect_to_near
8	6821624	0	T	C	6821617	2572	Periodontitis (PAL4Q3)	8p23.1	6964095	DEFA9P - DEFA10P	rs2738058	ream_gene_va	6.00E-06	New
8	24734686	22	C	T	24734677	677	Seasonality	8p21.2	24877164	C101929294 - LOC105379	rs196889	intron_variant	6.00E-06	Near_to_perfect
8	119766193	30	C	T	119766194	349	Pancreatitis	8q24.12	118753955	SAMD12-AS1 - RPS26P35	rs11988997	intron_variant	6.00E-06	Near_to_perfect
8	137768894	8	C	T	137768892	65	Bilirubin levels	8q24.23	136756649	C105375775 - LOC101927	rs16906293	intron_variant	6.00E-06	Near_to_perfect
9	100742254	18	C	T	100742253	3069	hyroid-stimulating hormon	9q22.33	97979971	HEMGN - ANP32B	rs7855088	ream_gene_va	6.00E-06	Perfect_to_near
10	37559574	22	A	G	37559580	2852	factors and hematological	10p11.21	37270652	C102724509 - LOC101929	rs1200821	intron_variant	6.00E-06	Smaller
14	56886680	22	A	C	56886686	797	tive pulmonary disease-rel	14q22.3	56419968	LOC105370514	rs17832777	ntergenic_varia	6.00E-06	Smaller
16	20674486	20	G	C	20674492	504	Schizophrenia	16p12.3	20663170	ACSM1	rs151222	intron_variant	6.00E-06	Non_identical
19	50909770	24	C	T	50909765	388	Obesity-related traits	19q13.33	50406508	POLD1	rs2230245	nonymous_vari	6.00E-06	Non_identical
1	70404547	24	A	T	70404540	2822	Obesity-related traits	1p31.1	69938857	LRRC7	rs1023008	intron_variant	6.00E-06	Non_identical
1	217718122	22	C	G	217718132	60	l adipose tissue adjusted	1q41	217544790	GPATCH2	rs2059397	intron_variant	6.00E-06	Smaller
21	15845055	12	A	G	15845052	805	one mineral density (spine	21q11.2	14472731	C105369304 - LOC105369	rs1006899	ntergenic_varia	6.00E-06	Non_identical
2	12553844	18	C	T	12553838	979	Electroencephalogram trait	2p24.3	12413712	LOC100506457	rs11677203	intron_variant	6.00E-06	Non_identical
10	133330019	26	G	A	133330026	1049	Temperament	10q26.3	131531763	LOC101927461 - LINC0116	rs11018023	ntergenic_varia	7.00E-06	Smaller
11	3722302	14	T	C	3722304	961	evels (Dihydroxy docosat	11p15.4	3701074	NUP98	rs685782	intron_variant	7.00E-06	Perfect_to_near
11	7464370	20	T	C	7464372	2015	IgG glycosylation	11p15.4	7443141	SYT9, LOC100506258	rs4076555	intron_variant	7.00E-06	Perfect_to_near
11	132584121	8	C	T	132584119	454	Alcohol dependence	11q25	132714224	OPCML	rs1793257	intron_variant	7.00E-06	Near_to_perfect
11	134139778	0	A	G	134139780	3582	Obesity-related traits	11q25	134269886	ACAD8 - GLB1L3	rs478881	ream_gene_va	7.00E-06	New
11	134139778	0	A	G	134139780	3582	Obesity-related traits	11q25	134269886	ACAD8 - GLB1L3	rs478881	ream_gene_va	7.00E-06	New
12	19927801	18	T	C	19927800	3346	t cancer hypertensive cas	12p12.3	19774866	C101928387 - LOC105369	rs2731620	ream_gene_va	7.00E-06	Perfect_to_near
12	32261234	16	A	G	32261239	2998	Obesity-related traits	12p11.21	32108305	BICD1	rs1144713	rime_UTR_var	7.00E-06	Perfect_to_near
12	124316872	12	A	G	124316876	1921	IgG glycosylation	12q24.31	123832329	DNAH10	rs7978454	intron_variant	7.00E-06	Near_to_perfect
13	31314676	14	T	C	31314680	4754	Non-word repetition	13q12.3	30740543	ALOX5AP	rs3922435	intron_variant	7.00E-06	Near_to_perfect
13	44702869	16	T	A	44702869	1579	Fasting insulin (interaction	13q14.11	44128733	LOC105370183, SMIM2-AS	rs9525916	intron_variant	7.00E-06	Perfect_to_near
13	95815532	20	A	G	95815535	1327	ess in chronic obstructive	13q32.1	95163281	ABCC4	rs3765535	intron_variant	7.00E-06	Near_to_perfect
14	48054161	12	A	G	48054159	2819	IgG glycosylation	14q21.3	47584956	MDGA2	rs4143912	intron_variant	7.00E-06	Perfect_to_near
15	78530937	16	G	A	78530940	3267	Personality dimensions	15q25.1	78238598	ACSBG1 - LOC105370911	rs1533665	intron_variant	7.00E-06	Perfect_to_near
16	24621347	16	G	A	24621348	1078	Electrodermal activity	16p12.1	24610027	RBBP6 - LINC01567	rs17831015	ream_gene_va	7.00E-06	Perfect_to_near
16	85906615	20	A	G	85906616	278	verse metabolic effects in	16q24.1	85873010	RPL10AP12 - IRF8	rs11648716	ntergenic_varia	7.00E-06	Perfect_to_near
17	77410858	14	T	C	77410859	1339	Self-reported allergy	17q25.3	79414777	RBFOX3	rs1316453	intron_variant	7.00E-06	Near_to_perfect
18	46227444	20	C	G	46227444	574	IgG glycosylation	18q21.1	48701073	CTIF	rs16949825	intron_variant	7.00E-06	Perfect_to_near
19	10798904	8	G	A	10798904	2129	Obesity-related traits	19p13.2	10688228	ILF3	rs2569507	intron_variant	7.00E-06	Near_to_perfect
19	18487665	12	C	T	18487667	80	Periodontitis (Mean PAL)	19p13.11	18376857	PGPEP1 - GDF15	rs78015699	intron_variant	7.00E-06	Near_to_perfect
1	82765051	8	T	C	82765052	449	Obesity-related traits	1p31.1	82299369	ADGRL2 - LOC105378814	rs1770678	ntergenic_varia	7.00E-06	Near_to_perfect
1	100335972	18	A	G	100335977	242	Pulmonary function decline	1p21.2	99870421	AGL	rs17121403	nissense_varia	7.00E-06	Perfect_to_near
1	154754195	16	C	T	154754194	3502	Obesity-related traits	1q21.3	154781718	KCNN3	rs6691316	intron_variant	7.00E-06	Near_to_perfect
1	161953394	0	G	A	161953400	556	a in Tripanosoma cruzi se	1q23.3	161983610	OLFML2B	rs12039519	rime_UTR_var	7.00E-06	New
1	174552804	16	C	T	174552811	442	Migraine - clinic-based	1q25.1	174583673	RABGAP1L	rs17301853	intron_variant	7.00E-06	Non_identical
20	14309953	16	A	G	14309952	731	ood disorder in prion disea	20p12.1	14329306	FLRT3, MACROD2	rs761998	rime_UTR_var	7.00E-06	Near_to_perfect
20	60514222	0	G	A	60514224	1329	Obesity-related traits	20q13.33	61939168	CDH4	rs4925325	rime_UTR_var	7.00E-06	New
2	2843307	8	G	A	2843308	1698	Smoking initiation	2p25.3	2839536	C105373390 - LOC101927	rs13403149	ream_gene_va	7.00E-06	Near_to_perfect
2	37957474	14	T	C	37957480	1406	bolite levels (HVA/MHPG	2p22.2	37730337	LOC105374465	rs13012266	intron_variant	7.00E-06	Near_to_perfect
2	64364631	0	T	C	64364632	3797	Allergic dermatitis (nickel)	2p14	64137498	PELI1	rs6733160	intron_variant	7.00E-06	New
2	174326852	18	C	T	174326845	544	e measurement (low sodiu	2q31.1	173462117	OC105373744 - LOC64399	rs10930597	ntergenic_varia	7.00E-06	Smaller
2	232159214	0	T	C	232159212	4225	Hormone measurements	2q37.1	231294499	ARMC9	rs745962	g_transcript_ex	7.00E-06	New
2	233998475	0	C	A	233998481	2400	Coronary heart disease	2q37.1	233133771	INPP5D	rs10933436	intron_variant	7.00E-06	New
2	236128947	0	A	G	236128951	4342	Obesity-related traits	2q37.2	235220307	OC642692 - LOC10537394	rs1991705	ntergenic_varia	7.00E-06	New
2	241515592	20	G	C	241515596	4116	Bipolar disorder	2q37.3	240576179	RNPEPL1	rs2953145	g_transcript_ex	7.00E-06	Perfect_to_near

3	113680949	8	A	G	113680951	1128	rohn's disease and psorias	3q13.31	113962104	ZDHHC23	rs1386478	rime_UTR_var	7.00E-06	Near_to_perfect
3	138916395	10	C	T	138916393	927	IgG glycosylation	3q23	139197551	BPESC1 - PISRT1	rs11706018	intron_variant	7.00E-06	Near_to_perfect
3	152672771	20	G	T	152672779	2701	ponse to antineoplastic ag	3q25.2	152954990	P2RY1 - HMGN2P13	rs6785504	itergenic_varia	7.00E-06	Smaller
3	169518456	16	T	C	169518455	2110	yeloma and monoclonal ga	3q26.2	169800667	LRRC34	rs6793295	nissense_varia	7.00E-06	Perfect_to_near
3	192772297	8	G	A	192772295	727	Sleep time	3q29	193054506	MB21D2 - VEZF1P1	rs13068101	itergenic_varia	7.00E-06	Near_to_perfect
5	173359332	8	A	G	173359331	90	Prion diseases	5q35.2	173932328	CPEB4	rs17763373	intron_variant	7.00E-06	Perfect_to_near
6	97922186	18	T	C	97922184	2016	Body mass index	6q16.1	97474308	LOC101927314	rs200810	intron_variant	7.00E-06	Perfect_to_near
7	9189540	18	A	C	9189541	2355	Obesity-related traits	7p21.3	9149911	RPL9P19 - GAPDHP68	rs1371737	intron_variant	7.00E-06	Perfect_to_near
7	67426323	20	T	C	67426323	574	formation processing spee	7q11.22	67961336	C105375340 - LOC102723	rs6961611	itergenic_varia	7.00E-06	Perfect_to_near
7	113741102	8	T	G	113741105	1787	IgG glycosylation	7q31.1	114101050	FOXP2	rs6466479	intron_variant	7.00E-06	Perfect_to_near
8	6821624	0	T	C	6821617	2572	Periodontitis (PAL4Q3)	8p23.1	6964095	DEFA9P - DEFA10P	rs2738058	ream_gene_va	7.00E-06	New
8	22088429	16	A	G	22088432	1231	Hypertriglyceridemia	8p21.3	22230919	PHYHIP	rs12541335	intron_variant	7.00E-06	Perfect_to_near
8	26718877	18	A	C	26718880	3229	Response to amphetamine	8p21.2	26861363	ADRA1A	rs4732957	intron_variant	7.00E-06	Near_to_perfect
8	118184780	8	C	T	118184783	1278	Type 2 diabetes	8q24.11	117172544	LOC105375716, SLC30A8	rs13266634	nissense_varia	7.00E-06	Near_to_perfect
9	38645055	26	C	A	38645055	899	drome or obsessive-comp	9p13.1	38645058	FAM201A - LOC101927024	rs10973956	stream_gene_v	7.00E-06	Near_to_perfect
9	74887701	16	A	C	74887703	1175	Suicidal ideation	9q21.13	72272787	GDA - LOC100289320	rs11143230	ream_gene_va	7.00E-06	Near_to_perfect
9	131084630	18	C	A,T	131084628	39,10	Obesity-related traits	9q34.11	128322349	TRUB2	rs11539570	nonymous_vari	7.00E-06	Perfect_to_near
10	60555581	8	A	G	60555578	300	Obesity-related traits	10q21.1	58795818	BICC1	rs11006263	intron_variant	7.00E-06	Bigger
11	120311150	20	A	G	120311157	93	Intraocular pressure	11q23.3	120440448	ARHGEF12	rs77216358	intron_variant	7.00E-06	Smaller
12	99334536	18	C	T	99334529	2591	Obesity-related traits	12q23.1	98940751	ANKS1B	rs483610	intron_variant	7.00E-06	Smaller
1	77945627	20	G	A	77945635	477	Body mass index	1p31.1	77479950	AK5	rs6695572	intron_variant	7.00E-06	Non_identical
1	77945627	20	G	A	77945635	477	Body mass index	1p31.1	77479950	AK5	rs6695572	intron_variant	7.00E-06	Non_identical
1	116761698	22	T	C	116761694	22	iisocyanate-induced asthn	1p13.1	116219072	MAB21L3 - LOC105378919	rs74346392	itergenic_varia	7.00E-06	Non_identical
1	159700042	20	A	G	159700039	433	C-reactive protein	1q23.2	159730249	CRP - RPL27P2	rs11265260	itergenic_varia	7.00E-06	Non_identical
20	24717090	12	C	A	24717085	2019	lar disorder and schizoph	20p11.21	24736449	SYNDIG1 - LOC105372578	rs2424635	itergenic_varia	7.00E-06	Bigger
2	219279087	24	A	G	219279097	2514	myotrophic lateral scleros	2q35	218414374	CTDSP1 - VIL1	rs7607369	ream_gene_va	7.00E-06	Smaller
2	220668731	16	C	T	220668738	2737	Orofacial clefts	2q35	219804017	C105373888 - LOC10192	rs3815854	itergenic_varia	7.00E-06	Non_identical
6	31074024	20	A	C	31074030	3418	Follicular lymphoma	6p21.33	31106253	HCG22 - C6orf15	rs6457327	stream_gene_v	7.00E-06	Non_identical
6	32140477	22	T	C	32140487	779	ersensitivity in acute lymp	6p21.32	32172710	AGPAT1	rs3130284	intron_variant	7.00E-06	Non_identical
6	71660104	18	A	G	71660111	3434	Obesity-related traits	6q13	70950408	B3GAT2	rs6922893	intron_variant	7.00E-06	Non_identical
6	80156252	20	C	T	80156259	1005	ce of antiphospholipid ant	6q14.1	79446542	OC102724109, LOC105377	rs12204683	stream_gene_v	7.00E-06	Non_identical
6	141123718	16	G	A	141123711	538	iisocyanate-induced asthn	6q24.1	140802574	C105378027 - LOC102723	rs61613191	itergenic_varia	7.00E-06	Smaller
7	114322118	32	C	T	114322124	1041	ite levels in obese individu	7q31.1	114682069	FOXP2	rs13226763	intron_variant	7.00E-06	Bigger
8	6152913	26	T	A	6152919	688	Metabolite levels (5-HIAA)	8p23.2	6295398	C105377797 - LOC10028	rs6559140	itergenic_varia	7.00E-06	Smaller
8	141150177	26	T	C	141150169	1168	Response to amphetamine	8q24.3	140140070	TRAPPC9	rs6992848	intron_variant	7.00E-06	Non_identical
9	31479012	18	C	T	31479005	337	Classic bladder exstrophy	9p21.1	31479007	LINC01243 - LOC10537601	rs12345625	itergenic_varia	7.00E-06	Smaller
10	21436221	0	C	A	21436222	303	o radiotherapy in cancer (l	10p12.31	21147293	NEBL	rs12243039	intron_variant	8.00E-06	New
11	13345589	24	G	A	13345593	2346	Body mass index	11p15.3	13324046	ARNTL	rs9633835	intron_variant	8.00E-06	Near_to_perfect
11	20623019	20	C	T	20623023	1637	Obesity-related traits	11p15.1	20601477	SLC6A5	rs2241941	nissense_varia	8.00E-06	Near_to_perfect
11	27336706	18	C	T	27336704	2931	mineral density (paediatric	11p14.1	27315157	LOC105376601 - CCDC34	rs10160456	itergenic_varia	8.00E-06	Near_to_perfect
11	48382469	8	A	G	48382471	4088	D-dimer levels	11p11.2	48360919	OR4C4P - OR4C10P	rs1351696	stream_gene_v	8.00E-06	Near_to_perfect
11	69999254	14	G	A	69999252	3433	Survival in rectal cancer	11q13.3	70153146	ANO1	rs3781663	intron_variant	8.00E-06	Near_to_perfect
11	82821380	20	T	G	82821382	867	-related macular degenera	11q14.1	83110340	LOC105369414	rs4293143	ream_gene_va	8.00E-06	Perfect_to_near
11	90956211	0	T	C	90956214	2496	angiotensin II receptor blo	11q14.3	91223046	LOC105369427	rs4491175	itergenic_varia	8.00E-06	New
11	120288367	20	T	C	120288372	2190	Intraocular pressure	11q23.3	120417663	ARHGEF12	rs10790382	intron_variant	8.00E-06	Absent
12	13641705	8	C	A	13641706	3674	Major depressive disorder	12p13.1	13488772	LINC01559 - GRIN2B	rs1457614	itergenic_varia	8.00E-06	Near_to_perfect
12	19052187	18	A	G	19052180	1916	Personality dimensions	12p12.3	18899246	LOC102724227 - RPL7P6	rs1873386	itergenic_varia	8.00E-06	Smaller
12	61549074	0	C	T	61549081	333	IgG glycosylation	12q14.1	61155300	LOC100996696 - PGBD3P1	rs17124610	itergenic_varia	8.00E-06	New
14	75327438	14	T	C	75327443	2075	IgG glycosylation	14q24.3	74860740	PROX2	rs722599	intron_variant	8.00E-06	Perfect_to_near
16	3124922	0	A	G	3124920	3365	HIV-1 susceptibility	16p13.3	3074919	IL32 - LOC105371058	rs4349147	intron_variant	8.00E-06	New
16	12718852	18	T	A	12718853	18	Type 2 diabetes	16p13.12	12624996	SNX29 - LOC105371088	rs189178161	itergenic_varia	8.00E-06	Near_to_perfect
16	20089751	8	G	A	20089754	1221	yperactivity disorder (inatt	16p12.3	20078432	GPR139 - SALL4P3	rs10521114	ream_gene_va	8.00E-06	Near_to_perfect
16	79252325	10	C	T	79252324	2788	hyroid-stimulating hormon	16q23.2	79218427	MAF	rs270421	intron_variant	8.00E-06	Perfect_to_near
17	45195852	8	G	A	45195850	989	odosum in inflammatory bo	17q21.32	47118484	CDC27	rs11079757	rime_UTR_var	8.00E-06	Near_to_perfect
18	10766432	16	A	G	10766425	4091	olite levels (5-HIAA/ MHPG	18p11.22	10766427	PIEZO2	rs2865126	intron_variant	8.00E-06	Near_to_perfect
18	38220101	22	C	T	38220099	544	Metabolite levels (X-11787	18q12.3	40640135	C105372082 - LOC105372	rs10502739	itergenic_varia	8.00E-06	Perfect_to_near
19	6475613	16	T	C	6475613	2353	therapy (neutropenia/leuc	19p13.3	6475602	DENND1C	rs3745571	rime_UTR_var	8.00E-06	Near_to_perfect
19	6992233	0	C	T	6992244	856	Body mass index	19p13.2	6992233	ADGRE4P - FLJ25758	rs183353539	ream_gene_va	8.00E-06	New
19	29489049	0	T	A	29489042	2420	Anger	19q12	28998135	LOC102724958 - LINC0153	rs8102754	ream_gene_va	8.00E-06	New
19	41673841	20	G	T	41673848	960	Prion diseases	19q13.2	41167943	LOC105376908	rs8105815	ream_gene_va	8.00E-06	Near_to_perfect
19	45392254	24	C	T	45392254	553	Frontotemporal dementia	19q13.32	44888997	PVRL2	rs6857	rime_UTR_var	8.00E-06	Near_to_perfect
1	59036624	20	C	T	59036625	161	Word reading	1p32.1	58570953	OMA1 - TACSTD2	rs638065	stream_gene_v	8.00E-06	Near_to_perfect
20	5840537	10	A	G	5840539	520	formation processing spee	20p12.3	5859893	C20orf196	rs4815868	intron_variant	8.00E-06	Near_to_perfect
20	20634105	18	T	C	20634106	383	IgG glycosylation	20p11.23	20653462	RALGAPA2	rs6132333	intron_variant	8.00E-06	Perfect_to_near
21	30594527	14	G	A	30594532	687	se to mTOR inhibitor (eve	21q21.3	29222211	LINC00189	rs2832270	intron_variant	8.00E-06	Near_to_perfect
22	22506570	20	A	C	22506569	2374	Periodontitis (PAL4Q3)	22q11.22	22152176	LOC102724653 - IGLV4-60	rs11089937	ream_gene_va	8.00E-06	Perfect_to_near
22	23644793	20	C	T	23644794	485	esponse to smallpox vacc	22q11.23	23302607	BCR	rs9608102	g_transcript_ex	8.00E-06	Perfect_to_near
22	39748849	22	A	G	39748854	3074	IgG glycosylation	22q13.1	39352849	SYNGR1	rs137699	intron_variant	8.00E-06	Perfect_to_near
2	21294978	0	G	A	21294975	3754	Response to statin therapy	2p24.1	21072103	LOC105374316	rs541041	itergenic_varia	8.00E-06	New
2	125291838	20	C	T	125291837	242	IgG glycosylation	2q14.3	124534260	CNTNAP5	rs13021885	intron_variant	8.00E-06	Near_to_perfect
2	185533573	24	T	C	185533580	2496	lisorder, bipolar disorder, r	2q32.1	184668853	ZNF804A	rs7597593	intron_variant	8.00E-06	Non_identical
2	230349981	0	T	C	230349989	334	IgG glycosylation	2q36.3	229485273	DNER	rs11890081	intron_variant	8.00E-06	New
2	231420400	0	A	C	231420401	374	Cognitive performance	2q37.1	230555686	SP100 - TPM3P8	rs17275498	intron_variant	8.00E-06	New
2	235601062	0	T	G	235601064	4028	angiotensin II receptor blo	2q37.1	234692420	C105373936 - LOC101927	rs4663476	intron_variant	8.00E-06	New
3	49453831	14	C	T	49453834	1091	Cognitive function	3p21.31	49416401	TCTA	rs6997	rime_UTR_var	8.00E-06	Near_to_perfect
3	116761662	14	G	C	116761660	176	Cognitive function	3q13.31	117042813	LINC00901 - LOC10537405	rs1521418	intron_variant	8.00E-06	Near_to_perfect
3	117027511	18	A	G	117027512	1550	a in Tripanosoma cruzi se	3q13.31	117308665	C105374054 - LOC105374	rs7632070	itergenic_varia	8.00E-06	Near_to_perfect
3	175927451	12	G	A	175927451	992	Height	3q26.32	176209663	ACTG1P23 - LOC10537422	rs11923600	itergenic_varia	8.00E-06	Near_to_perfect
4	7459876	8	A	G	7459875	186	Hippocampal sclerosis	4p16.1	7458148	SORCS2	rs62277617	intron_variant	8.00E-06	Perfect_to_near
4	38465465	16	C	T	38465471	716	Obesity-related traits	4p14	38463850	LINC01258	rs4615179	intron_variant	8.00E-06	Near_to_perfect

4	63342265	16	G	C	63342270	2222	-related macular degenera	4q13.1	62476552	OC105377251 - HMGN1P1	rs6819266	ıtergenic_varia	8.00E-06	Perfect_to_near
4	95476714	12	C	A	95476719	1547	Lewy body disease	4q22.3	94555568	PDLIM5	rs2452591	intron_variant	8.00E-06	Near_to_perfect
4	100395414	18	A	C	100395414	55	Eating disorders	4q23	99474257	ADH7 - C4orf17	rs148915469	intron_variant	8.00E-06	Near_to_perfect
4	100444686	8	T	C	100444684	3518	Metabolite levels (MHPG)	4q23	99523527	C4orf17	rs13126513	intron_variant	8.00E-06	Absent
4	169338524	24	A	C	169338520	2643	Obesity-related traits	4q32.3	168417369	DDX60L	rs1963569	intron_variant	8.00E-06	Near_to_perfect
5	29368023	8	C	T	29368024	445	Alzheimer's disease neurc	5p13.3	29367917	C101929660 - LOC101929€	rs16898904	ıtergenic_varia	8.00E-06	Near_to_perfect
5	55904895	8	T	C	55904898	97	chosis in Alzheimer's dise	5q11.2	56609071	C101928448 - LOC10537	rs17716202	ream_gene_va	8.00E-06	Near_to_perfect
5	78272943	8	T	C	78272942	2438	Iron status biomarkers	5q14.1	78977119	ARSB	rs2052550	intron_variant	8.00E-06	Perfect_to_near
5	122253302	8	C	T	122253304	3307	Glucose homeostasis trait	5q23.2	122917609	SNX24	rs193541	intron_variant	8.00E-06	Near_to_perfect
5	124848904	18	A	G	124848906	373	Obesity-related traits	5q23.2	125513213	LOC101927460	rs17468244	intron_variant	8.00E-06	Near_to_perfect
5	134372684	10	T	C	134372685	1628	Height	5q31.1	135036995	C5orf66	rs31198	intron_variant	8.00E-06	Near_to_perfect
5	152940402	18	C	T	152940405	520	Obesity-related traits	5q33.2	153560845	GRIA1	rs10065813	intron_variant	8.00E-06	Near_to_perfect
6	23077306	18	G	A	23077311	959	Tourette syndrome	6p22.3	23077083	LOC105374974	rs9393366	ıtergenic_varia	8.00E-06	Near_to_perfect
6	65662620	18	A	G	65662621	883	levels (Dihydroxy docosat	6q12	64952728	EYS	rs7767572	intron_variant	8.00E-06	Near_to_perfect
6	68327027	0	C	T	68327030	3132	Response to amphetamine	6q12	67617137	C105377842 - LOC10537	rs875033	ıtergenic_varia	8.00E-06	New
6	77290794	26	A	G	77290799	333	Capecitabine sensitivity	6q14.1	76581082	LOC105377860	rs12198063	intron_variant	8.00E-06	Near_to_perfect
6	166128639	18	G	A	166128638	782	political preferences (imm	6q27	165715150	PDE10A - LOC105378114	rs9364813	intron_variant	8.00E-06	Near_to_perfect
7	28911809	14	C	T	28911807	287	Vascular brain injury	7p14.3	28872190	CREB5 - TRIL	rs11769293	ıtergenic_varia	8.00E-06	Perfect_to_near
7	31623744	18	T	C	31623746	2570	somal-dominant arteriopat	7p14.3	31584132	CCDC129	rs1014137	intron_variant	8.00E-06	Perfect_to_near
7	75975587	18	C	T	75975586	1566	Multiple sclerosis	7q11.23	76346269	YWHAG	rs7779014	intron_variant	8.00E-06	Perfect_to_near
7	116577126	24	G	A	116577124	2035	Cognitive performance	7q31.2	116937070	CAPZA2 - LOC105375465	rs7782376	ıtergenic_varia	8.00E-06	Perfect_to_near
7	147615639	20	C	T	147615640	3668	IgG glycosylation	7q35	147918548	CNTNAP2	rs2538958	intron_variant	8.00E-06	Near_to_perfect
8	9395530	14	G	A	9395532	2599	tiple myeloma (hyperdiplo	8p23.1	9538022	LOC105379229 - TNKS	rs6601327	ıtergenic_varia	8.00E-06	Near_to_perfect
8	16682745	0	A	T	16682746	346	apine-induced agranulocy	8p22	16825237	LOC105379297	rs73201717	intron_variant	8.00E-06	New
9	2978373	14	A	C	2978377	434	blood pressure in sickle ce	9p24.2	2978377	ATP5HP2 - LINC01231	rs7045640	intron_variant	8.00E-06	Perfect_to_near
9	38456360	20	G	A	38456365	1398	Hypertension	9p13.1	38456368	.OC105376041 - GAS2L1P	rs1110183	ream_gene_va	8.00E-06	Near_to_perfect
9	132339072	10	G	T	132339070	413	abolite levels (Pyroglutam	9q34.11	129576791	LOC105376292	rs10988428	intron_variant	8.00E-06	Near_to_perfect
10	11719001	22	T	G	11719010	884	Obesity-related traits	10p14	11677011	C105376412, LOC105376	rs12265836	ream_gene_va	8.00E-06	Smaller
11	18291740	14	G	A	18291736	1592	Lewy body disease	11p15.1	18270189	SAA1 - HPS5	rs11024598	stream_gene_v	8.00E-06	Non_identical
12	131022000	24	A	C	131022010	3612	chosis and Alzheimer's dis	12q24.33	130537465	RIMBP2	rs1464108	intron_variant	8.00E-06	Non_identical
13	74730396	24	C	T	74730392	956	nerve measurement (rim	13q22.1	74156255	KLF12	rs8000245	ıtergenic_varia	8.00E-06	Smaller
1	76484017	18	G	T	76484014	2328	Personality dimensions	1p31.1	76018329	LOC101927342	rs12753569	intron_variant	8.00E-06	Non_identical
1	161915505	18	G	A	161915501	1661	kinson's disease (age of o	1q23.3	161945711	ATF6	rs10918270	intron_variant	8.00E-06	Non_identical
22	50128276	20	G	A	50128268	642	rophic lateral sclerosis (sp	22q13.33	49734620	C22orf34 - RPL5P35	rs4824093	ream_gene_va	8.00E-06	Smaller
2	29743726	18	T	C	29743733	1450	Body mass index	2p23.2	29520867	ALK	rs7578465	intron_variant	8.00E-06	Non_identical
2	37977020	18	G	T	37977017	1930	onduct disorder (interactio	2p22.2	37749874	OC105374465 - LOC34438	rs604381	stream_gene_v	8.00E-06	Non_identical
2	46205042	16	T	G	46205039	1252	Metabolite levels (X-1178	2p21	45977900	PRKCE	rs13396424	intron_variant	8.00E-06	Non_identical
2	177012583	24	C	T	177012578	2984	IgG glycosylation	2q31.1	176147850	HOXD3	rs4972806	intron_variant	8.00E-06	Non_identical
2	188180402	22	T	C	188180409	164	Vascular brain injury	2q32.1	187315682	LOC105373787 - CALCRL	rs62174474	intron_variant	8.00E-06	Smaller
3	143772253	16	T	C	143772247	1564	political preferences (fem	3q24	144053405	C3orf58 - LOC105374139	rs13068298	stream_gene_v	8.00E-06	Smaller
4	46823623	24	A	C	46823634	813	Migraine - clinic-based	4p12	46821617	COX7B2	rs11726563	intron_variant	8.00E-06	Smaller
5	115568749	30	T	C	115568757	2109	lar disorder and schizoph	5q23.1	116233060	COMMD10	rs253959	intron_variant	8.00E-06	Smaller
6	10963853	28	G	A	10963847	89	ate levels in lean individu	6p24.2	10963614	SYCP2L	rs1150561	intron_variant	8.00E-06	Smaller
7	133335179	22	T	G	133335176	1274	Tonometry	7q33	133650423	EXOC4	rs10488172	intron_variant	8.00E-06	Non_identical
8	66624237	16	A	G	66624232	226	IgG glycosylation	8q13.1	65711997	MTFR1	rs10504390	intron_variant	8.00E-06	Non_identical
9	22488688	24	G	A	22488678	3352	Tuberculosis	9p21.3	22488679	DMRTA1 - LINC01239	rs586716	ıtergenic_varia	8.00E-06	Smaller
9	117083796	18	C	A,T	117083803	134,2	n generation potential phe	9q32	114321523	LOC105376224 - ORM1	rs150611042	ream_gene_va	8.00E-06	Non_identical
10	100583916	0	C	T	100583915	3811	Obesity-related traits	10q24.2	98824158	HPSE2	rs2801405	intron_variant	9.00E-06	New
10	124887130	18	T	C	124887125	51	Periodontitis (Mean PAL)	10q26.13	123127609	ACADSB - HMX3	rs61862032	ıtergenic_varia	9.00E-06	Near_to_perfect
11	47127151	8	A	G	47127153	292	D-dimer levels	11p11.2	47105602	C11orf49	rs7117404	intron_variant	9.00E-06	Perfect_to_near
11	84247118	12	A	G	84247123	452	Chronic periodontitis	11q14.1	84536080	DLG2	rs10501568	intron_variant	9.00E-06	Near_to_perfect
11	120288367	20	T	C	120288368	2127	Intraocular pressure	11q23.3	120417659	ARHGEF12	rs10750173	intron_variant	9.00E-06	Absent
12	6435563	20	A	G	6435571	1544	Obesity-related traits	12p13.31	6326405	PLEKHG6	rs10849441	intron_variant	9.00E-06	Bigger
12	24786135	16	A	C	24786136	1562	Calcium levels	12p12.1	24633202	KNOP1P1 - RPL21P102	rs12832485	ıtergenic_varia	9.00E-06	Perfect_to_near
12	60735169	0	C	T	60735173	4869	Obesity-related traits	12q14.1	60341392	LOC100996696 - PGBD3P	rs7974425	stream_gene_v	9.00E-06	New
12	74471337	22	C	T	74471340	2785	dial infarction in coronary	12q21.1	74077560	C105369841 - LOC10050	rs2303970	ıtergenic_varia	9.00E-06	Near_to_perfect
12	96027757	8	A	G	96027759	1324	Breast cancer	12q22	95633983	USP44 - PGAM1P5	rs17356907	intron_variant	9.00E-06	Perfect_to_near
12	129637349	16	C	T	129637348	1068	Heart rate	12q24.33	129152803	TMEM132D	rs12824981	intron_variant	9.00E-06	Near_to_perfect
13	51194407	12	C	T	51194405	1250	Osteosarcoma	13q14.3	50620269	DLEU1 - DLEU7	rs573666	intron_variant	9.00E-06	Near_to_perfect
13	72048093	24	A	G	72048096	1515	rophic lateral sclerosis (sp	13q21.33	71473964	DACH1	rs9599848	intron_variant	9.00E-06	Near_to_perfect
13	98200570	8	G	A	98200572	4539	levels (Dihydroxy docosat	13q32.2	97548318	C105370325, LOC105370	rs2770228	ıtergenic_varia	9.00E-06	Perfect_to_near
14	56231802	18	A	C	56231800	684	apy (neutropenia/leucoper	14q22.3	55765082	.OC105370510 - RPL13AP	rs7494275	intron_variant	9.00E-06	Perfect_to_near
15	59719156	28	A	G	59719169	1636	lar disorder and schizoph	15q22.2	59426970	FAM81A	rs28890483	intron_variant	9.00E-06	Near_to_perfect
15	67066561	8	C	T	67066563	530	ubcutaneous adipose tiss	15q22.31	66774225	SMAD6	rs11858577	intron_variant	9.00E-06	Near_to_perfect
16	3796154	0	T	C	3796147	1529	QT interval (interaction)	16p13.3	3746146	CREBBP	rs129963	intron_variant	9.00E-06	New
16	75167579	8	G	A	75167579	4438	hn's disease (time to surg	16q23.1	75133681	ZFP1	rs7195303	intron_variant	9.00E-06	Near_to_perfect
16	81774796	12	T	C	81774798	2499	emotherapy (neutropenia/l	16q23.3	81741193	LOC105369213	rs12446319	intron_variant	9.00E-06	Perfect_to_near
16	82326815	24	G	A	82326805	338	Obesity-related traits	16q23.3	82293200	C105371365 - LOC10041	rs11863065	ıtergenic_varia	9.00E-06	Smaller
17	20023098	24	G	A	20023099	907	Obesity-related traits	17p11.2	20119786	SPECC1	rs2013441	intron_variant	9.00E-06	Near_to_perfect
17	32422688	0	G	T	32422699	912	l in Tripanosoma cruzi se	17q12	34095680	ASIC2	rs116908816	intron_variant	9.00E-06	New
18	59838918	18	T	G	59838919	168	taining treatment in HIV-1	18q21.33	62171686	PIGN	rs62096106	intron_variant	9.00E-06	Near_to_perfect
18	59838918	18	T	G	59838919	168	ntaining treatment in HIV 1	18q21.33	62171686	PIGN	rs62096106	intron_variant	9.00E-06	Near_to_perfect
1	57805548	8	A	C	57805550	498	HIV-1 control	1p32.2	57339878	DAB1	rs10159302	intron_variant	9.00E-06	Near_to_perfect
1	99453285	20	T	C	99453287	985	Obesity-related traits	1p21.3	98987731	LPPR5	rs10443196	intron_variant	9.00E-06	Perfect_to_near
1	100268990	0	G	A	100268991	3551	te matter integrity (interac	1p21.2	99803435	FRRS1 - RPL39P9	rs6689305	ıtergenic_varia	9.00E-06	New
1	147226790	0	A	G	147226791	4494	Cognitive performance	1q21.2	147754660	LOC102723321	rs1891498	ream_gene_va	9.00E-06	New
1	154754195	16	C	T	154754194	3502	Obesity-related traits	1q21.3	154781718	KCNN3	rs6691316	intron_variant	9.00E-06	Near_to_perfect
1	159753179	18	A	G	159753183	3256	Interstitial lung disease	1q23.2	159783393	DUSP23 - LOC105371464	rs4233356	stream_gene_v	9.00E-06	Perfect_to_near
1	183852909	16	G	C	183852914	2270	hyperactivity disorder and	1q25.3	183883780	RGL1	rs10797919	ce_region_vari	9.00E-06	Near_to_perfect
1	206715850	8	A	G	206715851	2919	Neurofibrillary tangles	1q32.1	206542522	RASSF5	rs11118993	g_transcript_ex	9.00E-06	Perfect_to_near

1	231899699	20	T	G	231899704	483	ponse to antineoplastic ag	1q42.2	231763958	TSNAX-DISC1, DISC1	rs2793086	intron_variant	9.00E-06	Near_to_perfect
20	6980635	16	G	T	6980635	113	heimer's disease (late ons	20p12.3	6999988	BMP2 - LINC01428	rs6085820	itergenic_varia	9.00E-06	Near_to_perfect
20	31531545	16	C	T	31531544	654	ype 1 diabetes nephropath	20q11.21	32943738	EFCAB8	rs13045180	stop_gained	9.00E-06	Perfect_to_near
20	43721490	10	C	T	43721493	703	Bipolar disorder	20q13.12	45092852	KCNS1	rs6124684	rime_UTR_var	9.00E-06	Near_to_perfect
20	44761483	8	T	G	44761485	1414	Response to amphetamine	20q13.12	46132846	CD40 - CDH22	rs12480534	stream_gene_v	9.00E-06	Near_to_perfect
2	9823879	20	A	C	9823870	1501	Mammographic density	2p25.1	9683741	C101926982 - LOC10537373	rs4669418	intron_variant	9.00E-06	Smaller
2	46321095	8	G	A	46321094	3694	Suicide risk	2p21	46093955	PRKCE	rs12373805	intron_variant	9.00E-06	Near_to_perfect
2	124515039	18	G	A	124515041	594	yperactivity disorder (inatt	2q14.3	123757465	LOC105373596 - CNTNAP5	rs13006237	ream_gene_va	9.00E-06	Perfect_to_near
2	166370823	20	A	C	166370826	3782	ngevity (85 years and old	2q24.3	165514316	CSRNP3	rs6432832	intron_variant	9.00E-06	Near_to_perfect
2	207121780	12	A	G	207121782	974	lar disorder and schizophr	2q33.3	206257058	GPR1-AS	rs2058710	intron_variant	9.00E-06	Near_to_perfect
2	234814051	0	C	T	234814059	2698	Pulmonary function decline	2q37.1	233905414	MSL3P1 - TRPM8	rs10187654	itergenic_varia	9.00E-06	New
3	13857968	14	T	C	13857969	2093	tive pulmonary disease-rel	3p25.1	13816472	LINC00620 - WNT7A	rs1124480	rime_UTR_var	9.00E-06	Near_to_perfect
3	21447335	20	G	A	21447336	366	on in Tripanosoma cruzi s	3p24.3	21405844	VENTXP7	rs409974	g_transcript_ex	9.00E-06	Absent
3	34013202	20	C	T	34013205	1256	ance related behavioral di	3p22.3	33971713	C105377024 - LOC10192	rs7620363	itergenic_varia	9.00E-06	Near_to_perfect
3	36842629	18	T	A	36842623	1416	lisorder, bipolar disorder,	3p22.2	36801132	C105377030 - LOC105377	rs13072940	itergenic_varia	9.00E-06	Near_to_perfect
3	40008630	24	T	C	40008634	1338	formation processing spe	3p22.1	39967143	MYRIP	rs9985399	intron_variant	9.00E-06	Near_to_perfect
3	103450597	16	A	C	103450591	1682	IgG glycosylation	3q13.11	103731747	MIR548AB - RAP1BP2	rs2677247	itergenic_varia	9.00E-06	Near_to_perfect
3	138916395	10	C	T	138916393	927	IgG glycosylation	3q23	139197551	BPESC1 - PISRT1	rs11706018	intron_variant	9.00E-06	Near_to_perfect
3	193537999	22	C	T	193537991	4210	on in chronic obstructive p	3q29	193820202	C105374283 - LOC105374	rs1165640	itergenic_varia	9.00E-06	Non_identical
4	7423753	26	C	A	7423751	2046	Obesity-related traits	4p16.1	7422024	SORCS2	rs7694661	intron_variant	9.00E-06	Perfect_to_near
4	89421082	18	A	G	89421086	174	Pulmonary function decline	4q22.1	88499935	HERC5	rs10516809	nonymous_vari	9.00E-06	Perfect_to_near
4	101901407	8	A	C	101901410	154	tion in Tripanosoma cruzi	4q24	100980253	LINC01216 - PPP3CA	rs72927357	itergenic_varia	9.00E-06	Near_to_perfect
4	128320908	14	T	C	128320908	189	Obesity-related traits	4q28.1	127399753	C102724210 - LOC101060	rs1443170	intron_variant	9.00E-06	Perfect_to_near
4	131026480	18	T	C	131026488	98	bolite levels (HVA/MHPG	4q28.3	130105333	C105377418 - LOC102724	rs114646238	itergenic_varia	9.00E-06	Near_to_perfect
4	165878334	20	A	G	165878335	1395	Obesity-related traits	4q32.3	164957183	FAM218A, TRIM61	rs3733418	nissense_varia	9.00E-06	Perfect_to_near
5	98593452	8	G	A	98593453	1090	Periodontitis (Mean PAL)	5q21.1	99257749	C102724810 - LOC100652	rs1500251	itergenic_varia	9.00E-06	Near_to_perfect
5	128722698	18	G	A	128722696	2507	Obesity-related traits	5q23.3	129387003	LOC102723654	rs10077875	itergenic_varia	9.00E-06	Perfect_to_near
6	20156176	22	T	C	20156174	4465	peractive-impulsive sympt	6p22.3	20155943	MBOAT1	rs1202199	intron_variant	9.00E-06	Perfect_to_near
6	20652718	14	C	G	20652717	1736	Type 2 diabetes	6p22.3	20652486	CDKAL1	rs9295474	intron_variant	9.00E-06	Perfect_to_near
6	44928955	8	T	C	44928953	106	Number of children	6p21.1	44961216	SUPT3H	rs16872971	intron_variant	9.00E-06	Near_to_perfect
6	164900595	20	G	A	164900598	1242	Major depressive disorder	6q27	164479565	C105378107 - LOC105378	rs4709845	itergenic_varia	9.00E-06	Near_to_perfect
7	89628633	18	G	A	89628635	605	Heschl's gyrus morpholog	7q21.13	89999321	STEAP2-AS1	rs73215715	intron_variant	9.00E-06	Perfect_to_near
7	93199926	22	T	A	93199928	3108	Body mass index	7q21.3	93570616	CALCR	rs7786765	intron_variant	9.00E-06	Near_to_perfect
8	22400988	22	C	T	22400989	144	HIV-1 viral setpoint	8p21.3	22543476	PPP3CC - SORBS3	rs4872511	g_transcript_ex	9.00E-06	Near_to_perfect
8	40484241	24	T	C	40484239	3697	Fasting plasma glucose	8p11.21	40626720	ZMAT4	rs2722425	intron_variant	9.00E-06	Perfect_to_near
8	60623345	8	A	C	60623348	3889	Obesity-related traits	8q12.1	59710789	C105375859 - LOC105375	rs12541902	itergenic_varia	9.00E-06	Near_to_perfect
8	121635389	18	C	A	121635396	511	lar disorder and schizophr	8q24.12	120623156	SNTB1	rs6986718	intron_variant	9.00E-06	Near_to_perfect
8	136989203	18	C	T	136989204	582	nsomnia (caffeine-induce	8q24.23	135976961	KHDRBS3 - LOC10537577	rs16905439	itergenic_varia	9.00E-06	Perfect_to_near
8	143691837	18	T	A	143691838	1011	lar disorder and schizophr	8q24.3	142610477	ADGRB1 - ARC	rs7465272	stream_gene_v	9.00E-06	Near_to_perfect
9	119798884	14	C	T	119798885	4811	Glucose homeostasis trait	9q33.1	117036606	ASTN2	rs7036846	intron_variant	9.00E-06	Near_to_perfect
9	135344408	18	G	A	135344407	2943	Obesity-related traits	9q34.13	132469020	C9orf171	rs569434	intron_variant	9.00E-06	Perfect_to_near
10	70712055	20	A	G	70712062	211	epression (quantitative tra	10q22.1	68952306	DDX50 - DDX21	rs2017305	ream_gene_va	9.00E-06	Non_identical
10	99637584	20	G	A	99637578	2486	Metabolic syndrome	10q24.2	97877821	CRTAC1	rs531676	intron_variant	9.00E-06	Bigger
11	18291740	14	G	A	18291736	1592	Lewy body disease	11p15.1	18270189	SAA1 - HPS5	rs11024598	stream_gene_v	9.00E-06	Non_identical
11	123956472	16	A	G	123956466	951	Obesity-related traits	11q24.2	124085759	LOC105369545	rs1893767	atory_region_v	9.00E-06	Smaller
12	48403769	14	G	A	48403765	1087	lar disorder and schizophr	12q13.11	48009982	COL2A1	rs11168351	intron_variant	9.00E-06	Non_identical
12	79811433	18	T	C	79811429	3516	Classic bladder exstrophy	12q21.2	79417649	SYT1	rs7310309	intron_variant	9.00E-06	Non_identical
16	54027965	20	T	C	54027971	645	hormone-binding globulin l	16q12.2	53994059	FTO	rs12596210	g_transcript_ex	9.00E-06	Smaller
3	64173894	18	T	C	64173901	1520	IgG glycosylation	3p14.1	64188225	PRICKLE2-AS3, PRICKLE2	rs1035275	intron_variant	9.00E-06	Non_identical
3	71464338	10	G	A	71464335	110	Migraine - clinic-based	3p13	71415184	FOXP1	rs13063872	intron_variant	9.00E-06	Smaller
3	174029032	28	A	G	174029045	3401	Major depressive disorder	3q26.31	174311255	NLGN1 - RPL8P4	rs13074924	itergenic_varia	9.00E-06	Smaller
6	44065319	32	G	A	44065311	1404	Major depressive disorder	6p21.1	44097574	RPL29P16 - MRPL14	rs7742824	itergenic_varia	9.00E-06	Smaller
9	21035296	28	G	T	21035307	2870	Axial length	9p21.3	21035308	LOC105375989	rs7867456	ream_gene_va	9.00E-06	Smaller
18	10382882	24	C	T	10382883	2353	Serum protein levels (sST2	18p11.22	10382886	LOC105371989	rs206548	stream_gene_v	9.00E+07	Near_to_perfect
19	45411944	8	T	C	45411941	754	Brain imaging	19q13.32	44908684	APOE	rs429358	nissense_variant		Perfect_to_near
1	63586972	8	G	T	63586973	1040	Brain imaging	1p31.3	63121302	LOC105378769 - LINC0046	rs7526034	itergenic_variant		Near_to_perfect
3	69623185	18	G	A,T	69623188	442,562	Brain imaging	3p14.1	69574037	FRMD4B - MITF	rs7647307	ream_gene_variant		Near_to_perfect
