# Supplementary material for: A reference catalog of DNA palindromes in the human genome and their variations in 1000 Genomes
Source: Hum Genome Var. 2020 Nov 20;7:40. doi: 10.1038/s41439-020-00127-5 (PMC7680136; doi:10.1038/s41439-020-00127-5)
Supplement: Supplementary file 6 — Supplementary Table 6 [file 41439_2020_127_MOESM6_ESM.pdf]

Disease	No.of SNPs associated with Palindromes	Percent of SNPs associated with Palindromes
Obesity-related traits	62	7.42
Height	57	8.68
IgG glycosylation	43	10.89
Body mass index	36	8.55
Crohn's disease	30	15.46
Intelligence	28	50.91
Schizophrenia	21	8.97
F-cell distribution	20	64.52
Type 2 diabetes	20	8.77
Breast cancer	19	12.58
Rheumatoid arthritis	17	9.29
Blood metabolite levels	17	9.14
Prostate cancer	15	10.64
Bipolar disorder and schizophrenia	14	11.57
Bipolar disorder	14	11.02
PR interval in Tripanosoma cruzi seropositivity	13	12.75
Amyotrophic lateral sclerosis (sporadic)	13	7.26
Response to amphetamines	12	13.48
Diisocyanate-induced asthma	12	6.67
Trans fatty acid levels	11	8.40
Motion sickness	10	28.57
Bilirubin levels	10	26.32
HDL cholesterol	10	6.54
Hemostatic factors and hematological phenotypes	9	31.03
Bone mineral density	9	7.96
QT interval	9	6.21
Multiple sclerosis	9	5.56
ion deficit-hyperactivity disorder, bipolar disorder, major depressive disorde	8	10.81
Colorectal cancer	8	9.76
Metabolite levels	8	8.16
Coronary heart disease	8	8.00
Inflammatory bowel disease	8	6.96
Total bilirubin levels in HIV-1 infection	7	63.64
Alzheimer's disease (cognitive decline)	7	15.91
Pulmonary function (interaction)	7	15.91
Educational attainment	7	9.46
Obesity	7	8.97
Cognitive performance	7	5.93
Ulcerative colitis	7	5.56
Fasting glucose-related traits (interaction with BMI)	6	19.35
IgA nephropathy	6	18.18
Pulmonary function decline	6	15.38
Orofacial clefts	6	13.33
Metabolic syndrome	6	12.77
Thiazide-induced adverse metabolic effects in hypertensive patients	6	10.17
Amyotrophic lateral sclerosis	6	10.17
Cognitive function	6	9.84
Glucose homeostasis traits	6	8.57
Waist-to-hip ratio adjusted for body mass index	6	8.45
Inflammatory skin disease	6	6.82
Blood pressure	6	6.67
Menarche (age at onset)	6	3.49
Nodular sclerosis Hodgkin lymphoma	5	100.00
Metabolite levels (Dihydroxy docosatrienoic acid)	5	25.00
Information processing speed	5	17.24
Response to tocilizumab in rheumatoid arthritis	5	16.13

Migraine - clinic-based	5	13.51
QRS duration in Tripanosoma cruzi seropositivity	5	13.16
Tuberculosis	5	12.50
Sitting height ratio	5	11.36
Polychlorinated biphenyl levels	5	10.87
Coronary artery calcification	5	8.93
Response to antipsychotic treatment	5	8.77
Phospholipid levels (plasma)	5	8.06
Urate levels	5	7.35
Migraine	5	7.14
Age-related macular degeneration	5	7.14
Attention deficit hyperactivity disorder	5	6.67
Major depressive disorder	5	6.49
Psoriasis	5	5.62
QT interval in Tripanosoma cruzi seropositivity	4	28.57
Word reading	4	26.67
Sphingolipid levels	4	25.00
Interstitial lung disease	4	22.22
Glycerophospholipid levels	4	21.05
Lewy body disease	4	20.00
Metabolite levels (MHPG)	4	20.00
Neurofibrillary tangles	4	19.05
Personality dimensions	4	19.05
Response to anti-depressant treatment in major depressive disorder	4	18.18
Metabolite levels (Pyroglutamine)	4	17.39
Metabolite levels (HVA/MHPG ratio)	4	16.00
Liver enzyme levels (gamma-glutamyl transferase)	4	15.38
Alcohol dependence (age at onset)	4	15.38
Hippocampal volume	4	14.81
Bone mineral density (spine)	4	14.81
Leprosy	4	12.90
C-reactive protein	4	12.50
HIV-1 viral setpoint	4	12.12
Heschl's gyrus morphology	4	12.12
Iron status biomarkers	4	11.76
Menopause (age at onset)	4	11.11
Periodontitis (Mean PAL)	4	11.11
Primary biliary cirrhosis	4	11.11
Disc degeneration (lumbar)	4	11.11
Heart rate	4	10.81
Visceral fat	4	10.26
Visceral adipose tissue/subcutaneous adipose tissue ratio	4	10.00
Lipid metabolism phenotypes	4	9.76
HIV-1 control	4	8.70
Protein quantitative trait loci	4	8.33
Metabolic traits	4	8.33
Bipolar disorder (body mass index interaction)	4	7.84
Blood metabolite ratios	4	7.55
Asthma	4	7.41
Red blood cell traits	4	6.35
Celiac disease	4	6.15
Alzheimer's disease (late onset)	4	5.88
Intraocular pressure	4	5.80
Platelet count	4	5.13
Parkinson's disease	4	4.00
LDL cholesterol	4	3.39
Response to diuretic therapy	3	100.00
Osteoarthritis (hip)	3	60.00
Type 2 diabetes and other traits	3	50.00

Vitamin B levels in ischemic stroke	3	37.50
Multiple myeloma and monoclonal gammopathy	3	33.33
Adverse response to chemotherapy (neutropenia/leucopenia) (carboplatin)	3	33.33
Response to antineoplastic agents	3	33.33
Cannabis dependence	3	30.00
Blood trace element (Se levels)	3	30.00
Brain imaging	3	30.00
Nasopharyngeal carcinoma	3	27.27
Psychosis and Alzheimer's disease	3	27.27
Electrodermal activity	3	25.00
Local histogram emphysema pattern	3	25.00
Follicular lymphoma	3	23.08
Presence of antiphospholipid antibodies	3	23.08
Elevated serum carcinoembryonic antigen levels	3	23.08
Immune reponse to smallpox (secreted IL-1beta)	3	23.08
Serum protein levels (sST2)	3	21.43
D-dimer levels	3	20.00
Tourette's syndrome or obsessive-compulsive disorder	3	20.00
Multiple myeloma	3	17.65
White blood cell types	3	15.79
Response to angiotensin II receptor blocker therapy	3	15.00
Fasting insulin-related traits (interaction with BMI)	3	15.00
Axial length	3	14.29
HIV-1 susceptibility	3	14.29
Serum thyroid-stimulating hormone levels	3	13.64
Bladder cancer	3	13.64
Attention deficit hyperactivity disorder (inattention symptoms)	3	13.64
Erectile dysfunction and prostate cancer treatment	3	13.04
Graves' disease	3	13.04
Testicular germ cell tumor	3	13.04
Alzheimer's disease biomarkers	3	13.04
Parasitemia in Tripanosoma cruzi seropositivity	3	12.50
Dementia and core Alzheimer's disease neuropathologic changes	3	12.50
Attention deficit hyperactivity disorder (hyperactivity-impulsivity symptoms)	3	12.00
Classic bladder exstrophy	3	12.00
Periodontitis (CDC/AAP)	3	11.54
Anorexia nervosa	3	11.54
Attention deficit hyperactivity disorder and conduct disorder	3	11.54
Diabetic retinopathy	3	10.71
Glycated hemoglobin levels	3	10.71
Refractive error	3	10.34
Metabolite levels (X-11787)	3	10.34
Verbal declarative memory	3	9.68
Chronic obstructive pulmonary disease-related biomarkers	3	8.82
Urate levels in obese individuals	3	8.57
Vitiligo	3	8.11
Calcium levels	3	7.89
Atopic dermatitis	3	7.69
Chronic lymphocytic leukemia	3	7.14
Longevity	3	6.98
Immune response to smallpox vaccine (IL-6)	3	6.52
Mean platelet volume	3	5.66
Alzheimer's disease	3	5.26
Pulmonary function	3	4.84
Hypertension	3	4.84
Type 1 diabetes	3	3.85
Cholesterol, total	3	3.30
Triglycerides	3	2.91
Systemic lupus erythematosus	3	2.75

Rotator cuff tears	2	100.00
Sexual dysfunction (female)	2	66.67
Suicide in bipolar disorder	2	50.00
Mammographic density	2	40.00
Erythema nodosum in inflammatory bowel disease	2	40.00
Periodontitis	2	33.33
Optic nerve measurement (rim area)	2	33.33
Blood pressure measurement (high sodium and potassium intervention)	2	33.33
Myocardial infarction	2	33.33
Protein C levels	2	28.57
Cerebrospinal AB1-42 levels	2	28.57
Smoking cessation in chronic obstructive pulmonary disease	2	25.00
Electroencephalographic traits in alcoholism	2	25.00
HDL Cholesterol - Triglycerides (HDLC-TG)	2	25.00
Adverse response to chemotherapy (neutropenia/leucopenia) (paclitaxel)	2	25.00
Osteoporosis-related phenotypes	2	25.00
Interferon alpha levels in systemic lupus erythematosus	2	25.00
Myasthenia gravis	2	25.00
Bone mineral accretion in asthma (oral corticosteroid dose interaction)	2	22.22
Lung function (forced expiratory volume in 1 second)	2	22.22
Aging	2	22.22
Amino acid levels	2	22.22
β2-Glycoprotein I (β2-GPI) plasma levels	2	22.22
Dietary macronutrient intake	2	22.22
Body mass index in non-asthmatics	2	22.22
Chronic obstructive pulmonary disease (moderate to severe)	2	22.22
Lymphoma	2	22.22
Complement C3 and C4 levels	2	20.00
Epilepsy (generalized)	2	20.00
Aging traits	2	20.00
Multiple sclerosis (OCB status)	2	20.00
Febrile seizures (MMR vaccine-related)	2	20.00
Vascular brain injury	2	20.00
Self-rated health	2	20.00
Longevity (90 years and older)	2	20.00
Parkinson disease and lewy body pathology	2	20.00
Proinsulin levels	2	20.00
Eating disorders (purging via substances)	2	18.18
Response to antipsychotic treatment in schizophrenia (working memory)	2	18.18
Male-pattern baldness	2	18.18
Total ventricular volume (Alzheimer's disease interaction)	2	18.18
Prion diseases	2	18.18
Progressive supranuclear palsy	2	18.18
Bone mineral density (paediatric, skull)	2	18.18
Subcortical brain region volumes	2	16.67
Immunoglobulin A	2	16.67
Hyperactive-impulsive symptoms	2	15.38
Colorectal cancer (diet interaction)	2	15.38
Tonometry	2	15.38
Resting heart rate	2	15.38
Fat distribution (HIV)	2	14.29
Renal cell carcinoma	2	14.29
Vitamin D levels	2	14.29
Palmitoleic acid (16:1n-7) plasma levels	2	13.33
Liver enzyme levels (alanine transaminase)	2	13.33
Eating disorders	2	13.33
Conduct disorder (symptom count)	2	13.33
Cutaneous malignant melanoma	2	12.50
Polycystic ovary syndrome	2	12.50

Fractional exhaled nitric oxide (childhood)	2	12.50
to anti-retroviral therapy (ddI/d4T) in HIV-1 infection (Grade 3 peripheral ne	2	12.50
Periodontitis (PAL4Q3)	2	12.50
QT interval (interaction)	2	11.76
Survival in rectal cancer	2	11.76
Obesity (extreme)	2	11.76
Heart failure	2	11.76
Lupus nephritis in systemic lupus erythematosus	2	11.76
Economic and political preferences (environmentalism)	2	11.76
Preeclampsia	2	11.11
Chagas cardiomyopathy in Tripanosoma cruzi seropositivity	2	11.11
Response to citalopram treatment	2	11.11
Breast cancer (early onset)	2	11.11
Cardiac Troponin-T levels	2	11.11
Homocysteine levels	2	10.53
Cardiac hypertrophy	2	10.53
Lipoprotein-associated phospholipase A2 activity and mass	2	10.53
Primary tooth development (time to first tooth eruption)	2	10.53
Non-alcoholic fatty liver disease histology (other)	2	10.00
Behavioural disinhibition (generation interaction)	2	10.00
Primary tooth development (number of teeth)	2	10.00
Response to serotonin reuptake inhibitors in major depressive disorder	2	10.00
RR interval (heart rate)	2	10.00
Idiopathic membranous nephropathy	2	9.52
Very long-chain saturated fatty acid levels (fatty acid 20:0)	2	9.52
Urate levels (BMI interaction)	2	9.52
Methotrexate phramacokinetics (acute lymphoblastic leukemia)	2	9.52
Type 1 diabetes autoantibodies	2	8.70
Alcohol consumption	2	8.70
Eosinophilic esophagitis	2	8.70
Sex hormone-binding globulin levels	2	8.33
Magnesium levels	2	8.33
Fasting plasma glucose	2	8.00
Waist-hip ratio	2	8.00
Ejection fraction in Tripanosoma cruzi seropositivity	2	8.00
Venous thromboembolism	2	7.69
Coronary artery disease	2	7.69
Electrocardiographic traits	2	7.41
esponse to abacavir-containing treatment in HIV-1 infection (virologic failur	2	7.41
Lipid traits	2	7.41
Lung cancer	2	7.41
Clozapine-induced agranulocytosis	2	7.14
Blood pressure (smoking interaction)	2	7.14
Cardiovascular disease risk factors	2	7.14
Inflammatory biomarkers	2	6.90
Alcohol dependence	2	6.90
Alzheimer's disease in APOE e4- carriers	2	6.90
Airflow obstruction	2	6.67
Urate levels in lean individuals	2	6.67
Breast size	2	6.45
Migraine without aura	2	6.25
Immune reponse to smallpox (secreted IFN-alpha)	2	6.25
Weight	2	6.06
PR interval	2	5.88
Hippocampal atrophy	2	5.71
Self-reported allergy	2	5.71
Subcutaneous adipose tissue	2	5.41
Mean corpuscular volume	2	5.26
Urate levels in overweight individuals	2	5.26

Uric acid levels	2	5.26
Diastolic blood pressure	2	4.55
Visceral adipose tissue adjusted for BMI	2	4.55
Systolic blood pressure	2	4.55
Prostate cancer (gene x gene interaction)	2	3.92
Adiponectin levels	2	3.85
Dental caries	2	3.13
Myopia (pathological)	2	2.56
Mesial temporal lobe epilepsy with hippocampal sclerosis	1	100.00
Cholesterol	1	100.00
Cholelithiasis-related traits in sickle cell anemia	1	100.00
Recombination rate (females)	1	100.00
Airway hyperresponsiveness	1	100.00
YKL-40 levels	1	100.00
Odorant perception (isobutyraldehyde)	1	100.00
Enteric fever	1	100.00
Crohn's disease (time to surgery)	1	100.00
Tardive dyskinesia	1	100.00
Otosclerosis	1	100.00
Arterial stiffness	1	100.00
5-HTT brain serotonin transporter levels	1	100.00
Functional MRI	1	50.00
Allergic dermatitis (nickel)	1	50.00
Cerebrospinal AB1-42 levels in Alzheimer's disease dementia	1	50.00
Serum lipase activity	1	50.00
Orofacial clefts (interaction)	1	50.00
Angiotensin-converting enzyme activity	1	50.00
Ulcerative colitis or Crohn's disease	1	50.00
Protein biomarker	1	50.00
Mixed cryoglobulinemia vasculitis in chronic hepatitis C infection	1	50.00
Neutrophil count in HIV-infection	1	50.00
Helicobacter pylori serologic status	1	50.00
Chronic periodontitis	1	50.00
Lymphocyte counts	1	50.00
onse to inhaled glucocorticoid treatment in asthma (percentage change of F	1	50.00
Nephrotic syndrome (acquired)	1	50.00
Urinary albumin excretion	1	33.33
Anxiety and major depressive disorder	1	33.33
Plasma renin activity levels	1	33.33
Thrombin generation potential phenotypes	1	33.33
Adverse response to radiation therapy	1	33.33
Adverse response to chemotherapy in breast cancer (alopecia)	1	33.33
verse response to chemotherapy (neutropenia/leucopenia) (cyclophospham	1	33.33
Mood disorder in prion disease	1	33.33
Cortical thickness	1	33.33
Natriuretic peptide levels	1	33.33
Hoarding	1	33.33
Testicular cancer	1	33.33
Colorectal cancer (calcium intake interaction)	1	33.33
Age-related macular degeneration (smoking status interaction)	1	33.33
Suicidal ideation	1	33.33
Free thyroxine concentration	1	33.33
Beta thalassemia/hemoglobin E disease	1	33.33
Breast cancer (male)	1	33.33
Monocyte early outgrowth colony forming units	1	33.33
Blood pressure measurement (cold pressor test)	1	25.00
Brain structure (hippocampal volume)	1	25.00
HIV-associated dementia	1	25.00
Serum alkaline phosphatase levels	1	25.00

Sporadic pituitary adenoma	1	25.00
Insulin-related traits	1	25.00
Ileal carcinoids	1	25.00
Non-substance related behavioral disinhibition	1	25.00
Vogt-Koyanagi-Harada syndrome	1	25.00
Other erythrocyte phenotypes	1	25.00
Mucinous ovarian carcinoma	1	25.00
Parent of origin effect on language impairment (paternal)	1	25.00
Matrix metalloproteinase levels	1	25.00
Crohn's disease and psoriasis	1	25.00
volume in cerebral autosomal-dominant arteriopathy with subcortical infarc	1	25.00
Response to anti-TNF alpha therapy in inflammatory bowel disease	1	25.00
Acenocoumarol maintenance dosage	1	25.00
Osteoprotegerin levels	1	25.00
Longevity (85 years and older)	1	25.00
Immunoglobulin G index levels in multiple sclerosis	1	25.00
nferior lateral ventricle volume (Cerebrospinal fluid biomarker status interac	1	25.00
Type 2 diabetes (dietary heme iron intake interaction)	1	25.00
Suicide risk	1	25.00
Asperger disorder	1	20.00
Soluble levels of adhesion molecules	1	20.00
Hearing function	1	20.00
Non-word repetition	1	20.00
Sleep time	1	20.00
Hyperopia	1	20.00
Atrioventricular conduction	1	20.00
Urinary metabolites	1	20.00
Parkinson's disease (age of onset)	1	20.00
Retinal vascular caliber	1	20.00
Male infertility	1	20.00
Pancreatitis	1	20.00
Adverse response to chemotherapy (neutropenia/leucopenia) (gemcitabine)	1	20.00
Non-obstructive azoospermia	1	20.00
Schizophrenia, bipolar disorder and depression (combined)	1	20.00
Freckles	1	20.00
Sleep-related phenotypes	1	20.00
Hip circumference (psychosocial stress interaction)	1	20.00
Breast cancer (prognosis)	1	20.00
White matter microstructure (global fractional anisotropy)	1	20.00
Bladder cancer (smoking interaction)	1	20.00
Cervical artery dissection	1	20.00
Gambling	1	20.00
Plasma homocysteine levels (post-methionine load test)	1	20.00
Iris characteristics	1	20.00
Spontaneous preterm birth (preterm birth)	1	20.00
Osteoarthritis biomarkers	1	20.00
Resistin levels	1	20.00
Adverse response to chemotherapy in breast cancer (alopecia) (paclitaxel)	1	20.00
Cerebrospinal P-tau181p levels	1	20.00
Erythrocyte sedimentation rate	1	20.00
Monocyte chemoattractant protein-1	1	20.00
Partial epilepsies	1	16.67
Cerebral amyloid angiopathy	1	16.67
Non-alcoholic fatty liver disease histology (AST)	1	16.67
Social autistic-like traits	1	16.67
Non-small cell lung cancer (survival)	1	16.67
Bone mineral density (paediatric, upper limb)	1	16.67
Hormone measurements	1	16.67
Platelet thrombus formation	1	16.67

Menarche and menopause (age at onset)	1	16.67
Acoustic startle blink response	1	16.67
Arthritis (juvenile idiopathic)	1	16.67
Nephropathy	1	16.67
Type 2 diabetes nephropathy	1	16.67
Testicular germ cell cancer	1	16.67
White matter integrity (interaction)	1	16.67
Nicotine use	1	16.67
Nonsyndromic cleft lip with or without cleft palate	1	16.67
Erectile dysfunction in type 1 diabetes	1	16.67
Blood pressure measurement (low sodium intervention)	1	16.67
Drug-induced liver injury (flucloxacillin)	1	16.67
e response to chemotherapy (neutropenia/leucopenia) (all topoisomerase in	1	16.67
Acute lymphoblastic leukemia (B-cell precursor)	1	16.67
Birth length	1	16.67
response to chemotherapy (neutropenia/leucopenia) (all anthracycline-base	1	16.67
Hepatitis C induced liver fibrosis	1	16.67
Osteosarcoma	1	16.67
Infantile hypertrophic pyloric stenosis	1	16.67
Sleep quality	1	14.29
Food antigen IgG levels	1	14.29
Eosinophil counts	1	14.29
Exudative age-related macular degeneration	1	14.29
Forced expiratory volume in 1 second (environmental interaction)	1	14.29
Schizophrenia or bipolar disorder	1	14.29
Response to simvastatin treatment (PCSK9 protein level change)	1	14.29
DNA methylation (parent-of-origin)	1	14.29
Glycemic traits (pregnancy)	1	14.29
Emphysema-related traits	1	14.29
Insulin-like growth factors	1	14.29
Narcolepsy	1	14.29
Adverse response to chemotherapy (neutropenia/leucopenia) (cisplatin)	1	14.29
Carotid atherosclerosis in HIV infection	1	14.29
Waist Circumference - Triglycerides (WC-TG)	1	14.29
to methylphenidate treatment in attention-deficit/hyperactivity disorder (bloo	1	14.29
Vein graft stenosis in coronary artery bypass grafting	1	14.29
Tourette syndrome	1	14.29
Blood trace element (Cu levels)	1	14.29
Immune response to anthrax vaccine	1	12.50
Aging (time to event)	1	12.50
Number of children	1	12.50
Depression and alcohol dependence	1	12.50
Psychosis in Alzheimer's disease	1	12.50
Alopecia areata	1	12.50
Plasma amyloid beta peptide concentrations (ABx-42)	1	12.50
Osteoarthritis	1	12.50
Hirschsprung disease	1	12.50
Adiposity	1	12.50
Red blood cell count	1	12.50
Response to statin therapy (LDL-C)	1	12.50
sma omega-6 polyunsaturated fatty acid levels (dihomo-gamma-linolenic a	1	12.50
Seasonality	1	12.50
Renal sinus fat	1	12.50
Anxiety in major depressive disorder	1	12.50
Thyroid cancer	1	12.50
PR segment	1	12.50
Total ventricular volume	1	12.50
Response to TNF antagonist treatment	1	12.50
Treatment response for severe sepsis	1	12.50

Estradiol levels	1	12.50
Response to mTOR inhibitor (everolimus)	1	12.50
Asparaginase hypersensitivity in acute lymphoblastic leukemia	1	11.11
Metabolite levels (HVA/5-HIAA ratio)	1	11.11
Age-related macular degeneration (GA)	1	11.11
Coffee consumption	1	11.11
White matter integrity	1	11.11
Serum uric acid levels	1	11.11
Bone mineral density (paediatric, total body less head)	1	11.11
C-reactive protein and white blood cell count	1	11.11
Insomnia (caffeine-induced)	1	11.11
Body mass index (change over time)	1	11.11
Oleic acid (18:1n-9) plasma levels	1	11.11
Renal function-related traits (sCR)	1	11.11
Response to radiotherapy in cancer (late toxicity)	1	11.11
Type 1 diabetes nephropathy	1	10.00
Systolic blood pressure in sickle cell anemia	1	10.00
Caffeine consumption	1	10.00
Superior frontal gyrus grey matter volume	1	10.00
Hepatocellular carcinoma	1	10.00
Esophageal squamous cell carcinoma	1	10.00
Renal function-related traits (eGRFcrea)	1	10.00
Sex hormone levels	1	10.00
Illicit drug use	1	10.00
Central corneal thickness	1	10.00
Systolic blood pressure (alcohol consumption interaction)	1	10.00
Psychosis (atypical)	1	10.00
Plasma amyloid beta peptide concentrations (ABx-40)	1	10.00
Response to antipsychotic treatment in schizophrenia (reasoning)	1	10.00
Acylcarnitine levels	1	9.09
Type 1 diabetes and autoimmune thyroid diseases	1	9.09
Yu-Zhi constitution type in type 2 diabetes	1	9.09
Cognitive decline (age-related)	1	9.09
Glaucoma	1	9.09
Folate pathway vitamin levels	1	9.09
vWF and FVIII levels	1	9.09
Immune response to measles-mumps-rubella vaccine	1	9.09
.ifetime average cigarettes per day in chronic obstructive pulmonary diseas	1	9.09
Metabolite levels (5-HIAA/ MHPG Ratio)	1	9.09
Attention deficit hyperactivity disorder (time to onset)	1	9.09
Blood and toenail selenium levels	1	8.33
Electrocardiographic conduction measures	1	8.33
Conduct disorder	1	8.33
Hematological parameters	1	8.33
Multiple sclerosis (severity)	1	8.33
Number of pregnancies	1	8.33
Mean corpuscular hemoglobin concentration	1	8.33
Economic and political preferences (feminism/equality)	1	8.33
Hypertriglyceridemia	1	8.33
Radiation response	1	8.33
Body mass index (interaction)	1	8.33
Palmitic acid (16:0) plasma levels	1	8.33
Allergic sensitization	1	8.33
Insulin resistance/response	1	7.69
Aortic root size	1	7.69
o chemotherapy in breast cancer hypertensive cases (cumulative dose) (be	1	7.69
Non-alcoholic fatty liver disease histology (lobular)	1	7.69
Response to taxane treatment (placlitaxel)	1	7.69
Malaria	1	7.69

Esophageal cancer	1	7.69
Metabolite levels (5-HIAA)	1	7.69
Hippocampal sclerosis	1	7.69
Depression (quantitative trait)	1	7.69
Fasting insulin (interaction)	1	7.14
Multiple myeloma (hyperdiploidy)	1	7.14
Serum metabolite levels	1	7.14
Anger	1	7.14
Waist-to-hip circumference ratio (interaction)	1	7.14
Liver enzyme levels (alkaline phosphatase)	1	7.14
Periodontitis (DPAL)	1	7.14
Refractive astigmatism	1	6.67
Infant length	1	6.67
Lipoprotein (a) - cholesterol levels	1	6.67
Ovarian cancer	1	6.67
Behcet's disease	1	6.67
Metabolic syndrome (bivariate traits)	1	6.67
Economic and political preferences (immigration/crime)	1	6.67
Post-traumatic stress disorder (asjusted for relatedness)	1	6.67
Conduct disorder (interaction)	1	6.67
Lead levels in blood	1	6.67
Response to hepatitis C treatment	1	6.67
Inattentive symptoms	1	6.67
Renal function-related traits (BUN)	1	6.67
Chronic hepatitis B infection	1	6.67
Homeostasis model assessment of insulin resistance (interaction)	1	6.67
IgE levels	1	6.25
Temperament	1	6.25
Toenail selenium levels	1	6.25
Clozapine-induced cytotoxicity	1	6.25
Response to taxane treatment (docetaxel)	1	6.25
Hip geometry	1	6.25
Alzheimer's disease in APOE e4+ carriers	1	5.88
Economic and political preferences	1	5.88
Panic disorder	1	5.88
Non-small cell lung cancer	1	5.88
Neuritic plaque	1	5.88
Acne (severe)	1	5.88
Fasting glucose-related traits	1	5.88
Corneal curvature	1	5.56
Optic disc area	1	5.56
Response to antipsychotic therapy (extrapyramidal side effects)	1	5.56
Antineutrophil cytoplasmic antibody-associated vasculitis	1	5.56
Systemic sclerosis	1	5.56
Political ideology	1	5.26
DNA methylation (variation)	1	5.26
Perioperative myocardial infarction in coronary artery bypass surgery	1	5.26
Gout	1	5.26
Antibody status in Tripanosoma cruzi seropositivity	1	5.00
Sleep duration	1	5.00
Cannabis use (initiation)	1	5.00
Frontotemporal dementia	1	5.00
Atrial fibrillation	1	5.00
Asthma and hay fever	1	4.76
Airway responsiveness in chronic obstructive pulmonary disease	1	4.76
Social communication problems	1	4.76
Brain structure	1	4.76
Attention deficit hyperactivity disorder (combined symptoms)	1	4.76
Capecitabine sensitivity	1	4.76

Urinary metabolites (H-NMR features)	1	4.76
Pit-and-Fissure caries	1	4.55
Epithelial ovarian cancer	1	4.55
Multiple myeloma (IgH translocation)	1	4.35
Migraine with aura	1	4.35
End-stage coagulation	1	4.35
Serum dimethylarginine levels (asymmetric/symetric ratio)	1	4.17
Pubertal anthropometrics	1	4.17
C-reactive protein levels	1	4.00
Ankylosing spondylitis	1	4.00
Electroencephalogram traits	1	3.85
Bronchopulmonary dysplasia	1	3.85
Neutrophil count	1	3.85
Hematological and biochemical traits	1	3.85
Thyroid hormone levels	1	3.85
Endometriosis	1	3.70
Smoking initiation	1	3.70
QRS duration	1	3.70
Age-related hearing impairment	1	3.45
Corneal astigmatism	1	3.33
Telomere length	1	3.33
Asthma (childhood onset)	1	3.23
Corneal structure	1	3.23
Chronic kidney disease	1	3.23
Allergic rhinitis	1	3.23
esponse to efavirenz-containing treatment in HIV 1 infection (virologic failur	1	2.86
Fibrinogen	1	2.70
Response to statin therapy	1	2.63
Smoking behavior	1	2.44
Pancreatic cancer	1	2.08
Sudden cardiac arrest	1	2.00

SNP Id	ulomeDBScore
rs679087	0
rs2262909	0
rs13126513	0
rs472402	0
rs542340	0
rs650258	0
rs7147996	0
rs11590421	0
rs186133190	0
rs1544167	0
rs10508921	0
rs531676	0
rs315135	0
rs4902960	0
rs17439560	0
rs5752223	0
rs4538475	0
rs12527089	0
rs10795917	0
rs10904849	0
rs17774576	0
rs10994336	0
rs113808744	0
rs2071348	0
rs9633835	0
rs17556665	0
rs17439299	0
rs10160456	0
rs1351696	0
rs1391576	0
rs600550	0
rs112295236	0
rs1723838	0
rs1357339	0
rs10501568	0
rs2658782	0
rs2075290	0
rs2075290	0
rs2075290	0
rs55974252	0
rs2900333	0
rs2900333	0
rs148621641	0
rs991427	0
rs10861337	0
rs3782455	0
rs9533090	0
rs9533090	0
rs9533799	0
rs8000124	0
rs186967887	0
rs10492494	0
rs3765535	0
rs12891047	0

rs28890483	0
rs75598935	0
rs1993293	0
rs189178161	0
rs10521114	0
rs8045064	0
rs17177078	0
rs17281813	0
rs16967753	0
rs2013441	0
rs225190	0
rs8074751	0
rs11654749	0
rs206548	0
rs2865126	0
rs6508673	0
rs1187220	0
rs17512836	0
rs62096106	0
rs62096106	0
rs2980976	0
rs17073641	0
rs17088339	0
rs412658	0
rs8105815	0
rs6857	0
rs6857	0
rs6857	0
rs6857	0
rs6857	0
rs6857	0
rs6857	0
rs6857	0
rs503279	0
rs503279	0
rs10903027	0
rs7533254	0
rs114216682	0
rs10159302	0
rs1125777	0
rs12568010	0
rs41453448	0
rs1125777	0
rs12568010	0
rs7526034	0
rs12069782	0
rs12122440	0
rs903263	0
rs12058524	0
rs6691316	0
rs6684514	0
rs6684514	0
rs6659742	0
rs74133262	0
rs2793086	0

rs6677208	0
rs761998	0
rs6043979	0
rs6124684	0
rs6097169	0
rs12530	0
rs12611820	0
rs13403149	0
rs1405262	0
rs17043947	0
rs13012266	0
rs2540917	0
rs9309413	0
rs6733938	0
rs1375144	0
rs10496964	0
rs958672	0
rs2074955	0
rs13389219	0
rs6432832	0
rs7587026	0
rs12476289	0
rs59979824	0
rs7563911	0
rs7589014	0
rs2348114	0
rs4673553	0
rs7580717	0
rs9287638	0
rs17684824	0
rs9837561	0
rs2569991	0
rs4330281	0
rs3821396	0
rs9985399	0
rs75594032	0
rs7647307	0
rs10511112	0
rs13078807	0
rs13078807	0
rs17023900	0
rs2677247	0
rs1488193	0
rs1386478	0
rs7632070	0
rs9868873	0
rs9856151	0
rs11706018	0
rs9825379	0
rs16851720	0
rs11924705	0
rs6789378	0
rs9878522	0
rs11923600	0
rs13068101	0

rs6844339	0
rs1533317	0
rs222054	0
rs17484474	0
rs148915469	0
rs72927357	0
rs1908039	0
rs13113376	0
rs1908038	0
rs1800789	0
rs4376189	0
rs12109285	0
rs16898904	0
rs2229882	0
rs10940138	0
rs12153606	0
rs1500251	0
rs6887649	0
rs193541	0
rs17468244	0
rs146579248	0
rs2457174	0
rs6890562	0
rs10065813	0
rs29784	0
rs6459467	0
rs2647045	0
rs9469300	0
rs9381040	0
rs734597	0
rs2207139	0
rs2207139	0
rs9395865	0
rs7744813	0
rs13202860	0
rs9320598	0
rs17059400	0
rs458017	0
rs3812111	0
rs531930	0
rs9375674	0
rs679582	0
rs9496398	0
rs6930576	0
rs2063714	0
rs4709845	0
rs1056053	0
rs740083	0
rs142857449	0
rs10262915	0
rs6465411	0
rs13221576	0
rs4729127	0
rs3779483	0
rs4730268	0

rs4730273	0
rs9641855	0
rs2888674	0
rs10480300	0
rs10480300	0
rs4872511	0
rs196889	0
rs4732957	0
rs7830371	0
rs2033732	0
rs160451	0
rs11558471	0
rs11558471	0
rs11558471	0
rs6986718	0
rs16925298	0
rs1110183	0
rs11138290	0
rs1875620	0
rs2081670	0
rs7045138	0
rs7027110	0
rs4838255	0
rs765132	0
rs12243039	0
rs501120	0
rs501120	0
rs2801405	0
rs758569	0
rs7394570	0
rs1167125	0
rs7974425	0
rs2325244	0
rs3742207	0
rs12884468	0
rs12100561	0
rs4349147	0
rs12444268	0
rs2865531	0
rs2865531	0
rs10514604	0
rs3826782	0
rs183353539	0
rs1120900	0
rs2064689	0
rs1004819	0
rs2201841	0
rs11209026	0
rs1343151	0
rs10889677	0
rs12567232	0
rs1932064	0
rs11165354	0
rs1891498	0
rs3093059	0

rs6056209	0
rs816535	0
rs541041	0
rs4315565	0
rs7594321	0
rs11890081	0
rs6716753	0
rs10201872	0
rs7423615	0
rs17275498	0
rs1669070	0
rs12475512	0
rs3103267	0
rs10933436	0
rs2241880	0
rs3792109	0
rs6704644	0
rs2602381	0
rs11891311	0
rs887829	0
rs887829	0
rs887829	0
rs887829	0
rs887829	0
rs887829	0
rs887829	0
rs11563251	0
rs11563251	0
rs10187654	0
rs7577262	0
rs6741751	0
rs6741751	0
rs6741751	0
rs6741751	0
rs11887188	0
rs896543	0
rs75968099	0
rs1511453	0
rs13145041	0
rs875033	0
rs9341808	0
rs404256	0
rs12195230	0
rs271170	0
rs7783529	0
rs17169634	0
rs4947534	0
rs4947534	0
rs3735025	0
rs10234749	0
rs2738058	0
rs2738058	0
rs2738058	0
rs181166265	0
rs514716	0

rs294856	0
rs5904818	0
rs11864909	0
rs11864909	0
rs470490	0
rs2570916	0
rs761926	0
rs6781149	0
rs1165640	0
rs7735699	0
rs186263310	0
rs1357978	0
rs12666280	0
rs5916687	0
rs5943057	0
rs2306677	0
rs11168351	0
rs7310309	0
rs10745527	0
rs1021188	0
rs12429545	0
rs78319313	0
rs7144649	0
rs6573416	0
rs17101394	0
rs2453533	0
rs16951021	0
rs34517613	0
rs318095	0
rs7533254	0
rs2064689	0
rs1004819	0
rs2201841	0
rs11209026	0
rs1343151	0
rs10889677	0
rs12567232	0
rs1023008	0
rs6695572	0
rs12044963	0
rs8133843	0
rs7578465	0
rs604381	0
rs1206397	0
rs11677416	0
rs16841200	0
rs7576072	0
rs3815854	0
rs3815854	0
rs6795349	0
rs6782531	0
rs1035275	0
rs6439167	0
rs6444931	0
rs10513686	0

rs7690543	0
rs6828740	0
rs2710833	0
rs469339	0
rs1515641	0
rs7765379	0
rs7765379	0
rs7765379	0
rs12204683	0
rs6569474	0
rs2267739	0
rs6959212	0
rs6959212	0
rs116979167	0
rs10242100	0
rs73660619	0
rs4841132	0
rs4841132	0
rs4841132	0
rs4841132	0
rs7017212	0
rs12542677	0
rs10504390	0
rs113889867	0
rs343496	0
rs1873747	0
rs150611042	0
rs2519093	0
rs5980075	0
rs5928363	0
rs1334893	0
rs61458523	0
rs4076555	0
rs1573535	0
rs2057178	0
rs495366	0
rs17368659	0
rs1816537	0
rs2731620	0
rs11564258	0
rs11564258	0
rs11057405	0
rs7296418	0
rs6563210	0
rs4143912	0
rs2842346	0
rs711355	0
rs2306786	0
rs3884558	0
rs3884558	0
rs6496044	0
rs1104918	0
rs735951	0
rs1381102	0
rs1991867	0

rs1991867	0
rs7207400	0
rs11662763	0
rs10502739	0
rs17697518	0
rs2635047	0
rs16949825	0
rs12959200	0
rs10469074	0
rs9961113	0
rs147615524	0
rs2064689	0
rs1004819	0
rs2201841	0
rs11209026	0
rs1343151	0
rs10889677	0
rs12567232	0
rs17121403	0
rs602633	0
rs2774292	0
rs4076764	0
rs4611001	0
rs7274811	0
rs6088735	0
rs1371614	0
rs6545816	0
rs6545817	0
rs1896294	0
rs11886868	0
rs10195871	0
rs10172646	0
rs11683197	0
rs2298948	0
rs2100290	0
rs13402330	0
rs1036736	0
rs4848780	0
rs13006237	0
rs7568498	0
rs6753739	0
rs2176040	0
rs6731443	0
rs2953145	0
rs2399126	0
rs769554	0
rs78411303	0
rs9826463	0
rs6793295	0
rs6793295	0
rs6819266	0
rs115136538	0
rs10516809	0
rs9991328	0
rs1842896	0

rs4253311	0
rs4253311	0
rs11949289	0
rs1823068	0
rs2307121	0
rs2199161	0
rs261967	0
rs288232	0
rs11168048	0
rs11168048	0
rs10076782	0
rs1895320	0
rs2278255	0
rs3799296	0
rs4715166	0
rs1202199	0
rs16894878	0
rs6917603	0
rs1029295	0
rs4424056	0
rs200810	0
rs6938574	0
rs9373124	0
rs9373124	0
rs9389269	0
rs10945919	0
rs1524058	0
rs3757840	0
rs10268774	0
rs4733271	0
rs2722425	0
rs7000782	0
rs800586	0
rs16905439	0
rs12001137	0
rs10811771	0
rs7848024	0
rs1536895	0
rs2472476	0
rs13290997	0
rs2807580	0
rs56156506	0
rs2295912	0
rs4387258	0
rs1873386	0
rs3784609	0
rs4669418	0
rs6733011	0
rs10930597	0
rs10930597	0
rs6785504	0
rs6774494	0
rs9714717	0
rs301901	0
rs11954519	0

rs634308	0
rs7739264	0
rs10885531	0
rs610932	0
rs4408325	0
rs2290720	0
rs8000245	0
rs2803904	0
rs12902421	0
rs12940887	0
rs4824093	0
rs7569716	0
rs6756629	0
rs6756629	0
rs1527243	0
rs4664774	0
rs6732189	0
rs10510217	0
rs1809529	0
rs13063872	0
rs1554120	0
rs6764050	0
rs11726563	0
rs1399212	0
rs2456203	0
rs182503338	0
rs182503338	0
rs1727638	0
rs61613191	0
rs6974491	0
rs6974491	0
rs10448044	0
rs7867456	0
rs586716	0
rs10968576	0
rs10968576	0
rs12345625	0
rs7929679	4
rs4666360	4
rs7094310	4
rs11254363	4
rs1329650	4
rs11190179	4
rs2241941	4
rs3781663	4
rs12824981	4
rs11621145	4
rs11858577	4
rs9941219	4
rs312457	4
rs621636	4
rs4368253	4
rs72974768	4
rs1077667	4
rs2569507	4

rs737337	4
rs17131547	4
rs2274432	4
rs4815868	4
rs12480534	4
rs140522	4
rs140522	4
rs77569859	4
rs2540051	4
rs10937275	4
rs34311866	4
rs4689388	4
rs7720838	4
rs2073643	4
rs10037670	4
rs9393903	4
rs6899976	4
rs6967385	4
rs1534696	4
rs4948102	4
rs115744676	4
rs73229090	4
rs13266634	4
rs13266634	4
rs13266634	4
rs7919006	4
rs17152411	4
rs742071	4
rs642961	4
rs7352944	4
rs6436839	4
rs11677466	4
rs17863787	4
rs4663476	4
rs2842895	4
rs11043007	4
rs11577628	4
rs711830	4
rs17030434	4
rs1245541	4
rs12099085	4
rs678069	4
rs1532624	4
rs1532624	4
rs1532624	4
rs11209003	4
rs12753569	4
rs6693017	4
rs913678	4
rs2592394	4
rs2072590	4
rs2072590	4
rs1540771	4
rs806276	4
rs2380220	4

rs4735738	4
rs3123078	4
rs7090445	4
rs10458771	4
rs2237886	4
rs478607	4
rs3217901	4
rs10841496	4
rs2188380	4
rs10132579	4
rs1258767	4
rs7179432	4
rs6598541	4
rs8049607	4
rs7404928	4
rs11868441	4
rs820218	4
rs7250872	4
rs3093030	4
rs2292342	4
rs2287921	4
rs2287921	4
rs2287921	4
rs2287921	4
rs1976403	4
rs3827730	4
rs11209003	4
rs11590090	4
rs7606173	4
rs4849121	4
rs3733418	4
rs9328448	4
rs4959053	4
rs1371737	4
rs17151904	4
rs11769293	4
rs7779014	4
rs10776934	4
rs4458264	4
rs4458264	4
rs12479254	4
rs1893767	4
rs17797882	4
rs743777	4
rs743777	4
rs1550623	4
rs4850410	4
rs11958779	5
rs73912949	5
rs11006263	5
rs2007044	5
rs317689	5
rs11247009	5
rs2880058	5
rs2424635	5

rs3891585	5
rs9841144	5
rs10906142	5
rs1801239	5
rs266095	5
rs2291429	5
rs10857636	5
rs2263638	5
rs1111875	5
rs7903146	5
rs7903146	5
rs7903146	5
rs7903146	5
rs7903146	5
rs7903146	5
rs7903146	5
rs7903146	5
rs2981579	5
rs1078806	5
rs10857712	5
rs2237895	5
rs3858526	5
rs7940423	5
rs1007392	5
rs508970	5
rs2851682	5
rs606452	5
rs12576775	5
rs12576775	5
rs4938534	5
rs10892761	5
rs75059851	5
rs2272046	5
rs995030	5
rs77956314	5
rs11061269	5
rs954108	5
rs1886512	5
rs2526932	5
rs7158300	5
rs4905226	5
rs7173964	5
rs12719740	5
rs2573625	5
rs2573652	5
rs6498068	5
rs17831015	5
rs305061	5
rs1859962	5
rs7219585	5
rs898534	5
rs1316453	5
rs8089099	5
rs3745571	5
rs78015699	5

rs7248888	5
rs2289731	5
rs12711517	5
rs2305016	5
rs7535752	5
rs7535752	5
rs12568771	5
rs16835742	5
rs638065	5
rs12035879	5
rs3736757	5
rs11584383	5
rs11584383	5
rs7523273	5
rs1690789	5
rs6108011	5
rs932541	5
rs6044003	5
rs6127471	5
rs2023454	5
rs127430	5
rs2427308	5
rs2839627	5
rs229527	5
rs229527	5
rs17000918	5
rs5771069	5
rs6413458	5
rs17511102	5
rs6741892	5
rs7579899	5
rs115522963	5
rs3771381	5
rs4252023	5
rs4321325	5
rs72899866	5
rs2551802	5
rs2058710	5
rs11680012	5
rs3739070	5
rs3749171	5
rs7620363	5
rs7650267	5
rs2742417	5
rs35593266	5
rs1847202	5
rs9810857	5
rs6788895	5
rs9841616	5
rs4234853	5
rs10489087	5
rs4615179	5
rs1902859	5
rs1902859	5
rs1902859	5

rs3853444	5
rs13144478	5
rs2090104	5
rs17031671	5
rs1963569	5
rs402710	5
rs17716202	5
rs6893807	5
rs31198	5
rs9393366	5
rs16889440	5
rs7759742	5
rs972275	5
rs7772697	5
rs184074	5
rs926849	5
rs9364813	5
rs6968865	5
rs73302615	5
rs38313	5
rs314370	5
rs2687481	5
rs791595	5
rs2854536	5
rs11771145	5
rs1005390	5
rs1005390	5
rs2360806	5
rs10089517	5
rs10089517	5
rs40457	5
rs6993449	5
rs1036821	5
rs7465272	5
rs35419961	5
rs4745062	5
rs2006996	5
rs10988428	5
rs495828	5
rs495828	5
rs495828	5
rs495828	5
rs495828	5
rs4491175	5
rs478881	5
rs7979473	5
rs10773046	5
rs223116	5
rs129963	5
rs9924951	5
rs712046	5
rs8102754	5
rs11465802	5
rs9988642	5
rs6669582	5

rs10789230	5
rs905938	5
rs905938	5
rs12039519	5
rs11086243	5
rs4925325	5
rs2075876	5
rs8127571	5
rs10166311	5
rs745962	5
rs1656404	5
rs1881492	5
rs10210302	5
rs12994997	5
rs10173355	5
rs3755319	5
rs1991705	5
rs13401104	5
rs7607316	5
rs7599706	5
rs7642243	5
rs12198220	5
rs9314614	5
rs2980879	5
rs1782032	5
rs4132699	5
rs7916441	5
rs7916441	5
rs12772169	5
rs10838532	5
rs3889237	5
rs11675119	5
rs11713158	5
rs17584516	5
rs4629318	5
rs11713158	5
rs1173766	5
rs2392510	5
rs9406636	5
rs8176741	5
rs2393967	5
rs11024598	5
rs11263654	5
rs4938642	5
rs1464500	5
rs2731006	5
rs12868495	5
rs8008758	5
rs11864146	5
rs12447690	5
rs11880333	5
rs2230245	5
rs1172822	5
rs1172822	5
rs4648845	5

rs3920498	5
rs16835742	5
rs11465802	5
rs9988642	5
rs6669582	5
rs10789230	5
rs10918270	5
rs11809180	5
rs12134279	5
rs1739654	5
rs6124878	5
rs11703808	5
rs761746	5
rs9621305	5
rs1058065	5
rs10194115	5
rs4972806	5
rs3849570	5
rs13136331	5
rs898518	5
rs11741861	5
rs5985	5
rs2457335	5
rs6922893	5
rs9918668	5
rs4731742	5
rs10488172	5
rs7814749	5
rs6992848	5
rs2999399	5
rs71508903	5
rs685782	5
rs7112383	5
rs508487	5
rs4937362	5
rs4937362	5
rs12422552	5
rs1520832	5
rs80130819	5
rs10875943	5
rs17178006	5
rs17178006	5
rs9318086	5
rs9525638	5
rs9525638	5
rs2770228	5
rs4907479	5
rs4280164	5
rs8019546	5
rs7157785	5
rs7157785	5
rs7157785	5
rs7157785	5
rs1077989	5
rs1077989	5

rs737387	5
rs2842347	5
rs722599	5
rs10150332	5
rs1129038	5
rs936229	5
rs7181753	5
rs2018860	5
rs12446940	5
rs2221433	5
rs7197653	5
rs270421	5
rs30388	5
rs12446319	5
rs3844412	5
rs11648716	5
rs242557	5
rs11651708	5
rs71380849	5
rs357894	5
rs3745567	5
rs1051738	5
rs8099939	5
rs10119	5
rs429358	5
rs429358	5
rs429358	5
rs429358	5
rs61776290	5
rs3790645	5
rs11465802	5
rs9988642	5
rs6669582	5
rs10789230	5
rs115699453	5
rs6060278	5
rs2238732	5
rs9608102	5
rs137699	5
rs4038131	5
rs404005	5
rs10189857	5
rs1427407	5
rs7599488	5
rs766432	5
rs4671393	5
rs7584113	5
rs7557939	5
rs6706648	5
rs6738440	5
rs6729815	5
rs1896295	5
rs1896296	5
rs17636747	5
rs13419716	5

rs2114646	5
rs231770	5
rs972540	5
rs12151790	5
rs11679072	5
rs4675874	5
rs1372072	5
rs10865924	5
rs6784615	5
rs13072512	5
rs2135319	5
rs1801725	5
rs7694661	5
rs62277617	5
rs3114018	5
rs3114018	5
rs12500579	5
rs17049741	5
rs2726807	5
rs1912826	5
rs7725218	5
rs11743355	5
rs2052550	5
rs7700895	5
rs17763373	5
rs4713103	5
rs9383153	5
rs7756992	5
rs1799945	5
rs1799945	5
rs1799945	5
rs1799945	5
rs4418214	5
rs4418214	5
rs3094604	5
rs28421666	5
rs17299841	5
rs10259085	5
rs1014137	5
rs10237067	5
rs500454	5
rs6466479	5
rs7784447	5
rs7782376	5
rs12541335	5
rs28834970	5
rs11991744	5
rs4360309	5
rs61271866	5
rs3747547	5
rs7855088	5
rs10121150	5
rs7021663	5
rs34971035	5
rs34971035	5

rs17582416	5
rs11018023	5
rs1014922	5
rs5757949	5
rs1550546	5
rs9301951	5
rs17832777	5
rs6540223	5
rs2059397	5
rs4239702	5
rs597800	5
rs4618210	5
rs10510837	5
rs2670321	5
rs13068298	5
rs6869841	5
rs7448069	5
rs2621416	5
rs6901250	5
rs42648	5
rs10822136	6
rs10750173	6
rs10790382	6
rs78153629	6
rs6977820	6
rs644234	6
rs9615264	6
rs202072	6
rs7775861	6
rs7791362	6
rs11049611	6
rs7297610	6
rs11104877	6
rs11071200	6
rs8023580	6
rs9956738	6
rs17011455	6
rs13028485	6
rs1429411	6
rs62287976	6
rs1501357	6
rs1247318	6
rs17513961	6
rs13226763	6
rs11777116	6
rs10817758	6
rs11594111	6
rs7076247	6
rs7922491	6
rs56322409	6
rs927821	6
rs61862032	6
rs12788102	6
rs11041530	6
rs607987	6

rs9300039	6
rs174555	6
rs174555	6
rs174555	6
rs10792830	6
rs10831284	6
rs1793257	6
rs1457614	6
rs4363657	6
rs4363657	6
rs1606355	6
rs7980799	6
rs11172134	6
rs2303970	6
rs4964469	6
rs1896312	6
rs7978454	6
rs2798269	6
rs11571833	6
rs11571833	6
rs573666	6
rs1340319	6
rs9599848	6
rs11838918	6
rs11838918	6
rs7328464	6
rs1529276	6
rs1956388	6
rs8004664	6
rs1258763	6
rs16964211	6
rs1528473	6
rs937254	6
rs1077835	6
rs3743266	6
rs12591650	6
rs12050794	6
rs2764743	6
rs274068	6
rs12599426	6
rs2080454	6
rs7195303	6
rs2074409	6
rs7216389	6
rs11079757	6
rs7237848	6
rs10775480	6
rs1970671	6
rs10469266	6
rs73963343	6
rs10514168	6
rs632111	6
rs3826656	6
rs9628987	6
rs9628987	6

rs74225573	6
rs4654925	6
rs72647484	6
rs528059	6
rs544991	6
rs6686842	6
rs657452	6
rs117633859	6
rs4650608	6
rs4650608	6
rs4650608	6
rs11162963	6
rs1770678	6
rs1146509	6
rs857684	6
rs12406092	6
rs2488389	6
rs12130212	6
rs12757165	6
rs6687758	6
rs6687758	6
rs7529251	6
rs6045676	6
rs4815879	6
rs6085820	6
rs6040399	6
rs680379	6
rs680379	6
rs680379	6
rs2208059	6
rs6044001	6
rs6047116	6
rs1160312	6
rs2281558	6
rs2832270	6
rs4911642	6
rs2330809	6
rs1001021	6
rs9607782	6
rs6741148	6
rs12373805	6
rs4852324	6
rs1470506	6
rs13021885	6
rs34311235	6
rs3820706	6
rs16836124	6
rs12693043	6
rs13424612	6
rs1669338	6
rs1124480	6
rs76942353	6
rs1490157	6
rs13072940	6
rs538867	6

rs13064588	6
rs10514718	6
rs1521418	6
rs1830084	6
rs6794092	6
rs78647349	6
rs11725593	6
rs1906528	6
rs6832769	6
rs7685921	6
rs340630	6
rs2725236	6
rs6855885	6
rs2452591	6
rs2087160	6
rs7655841	6
rs114646238	6
rs11099040	6
rs1492820	6
rs1378552	6
rs11748327	6
rs4701523	6
rs6888304	6
rs71627250	6
rs921943	6
rs921943	6
rs1829883	6
rs11241936	6
rs12661281	6
rs204999	6
rs9268542	6
rs6903608	6
rs10484561	6
rs7767572	6
rs9447700	6
rs12198063	6
rs10155981	6
rs10238623	6
rs12532960	6
rs62459614	6
rs7786765	6
rs12533251	6
rs4646450	6
rs17477177	6
rs5009270	6
rs2538958	6
rs114252942	6
rs6601327	6
rs6989010	6
rs1594829	6
rs10958369	6
rs7815788	6
rs12541902	6
rs2403083	6
rs921231	6

rs7014900	6
rs11988997	6
rs16906293	6
rs10973956	6
rs11143230	6
rs1888221	6
rs7036846	6
rs10429475	6
rs11796357	6
rs11796357	6
rs5907577	6
rs10508503	6
rs11245052	6
rs2509843	6
rs17124610	6
rs6563569	6
rs4886238	6
rs2282032	6
rs2854437	6
rs11634397	6
rs7477	6
rs116908816	6
rs12140275	6
rs6663731	6
rs11209002	6
rs2902440	6
rs11465804	6
rs10889676	6
rs6689305	6
rs7523050	6
rs7550169	6
rs17791782	6
rs13046373	6
rs12986445	6
rs12986445	6
rs1465618	6
rs6733160	6
rs6712333	6
rs6750795	6
rs6728302	6
rs1656402	6
rs2741012	6
rs1875263	6
rs929596	6
rs10166942	6
rs13025591	6
rs11687170	6
rs358806	6
rs6448771	6
rs546829	6
rs11951515	6
rs647161	6
rs12663356	6
rs9267911	6
rs12196860	6

rs10455590	6
rs7806994	6
rs2840445	6
rs12716641	6
rs73201717	6
rs10098647	6
rs10812610	6
rs10969853	6
rs8015016	6
rs17301853	6
rs17301853	6
rs13428823	6
rs7597593	6
rs1864982	6
rs788076	6
rs10763170	6
rs2017305	6
rs7923837	6
rs7108470	6
rs4474514	6
rs1464108	6
rs3736995	6
rs17111394	6
rs151222	6
rs7206971	6
rs7206971	6
rs1380836	6
rs1787200	6
rs7244261	6
rs6686929	6
rs3091242	6
rs528059	6
rs544991	6
rs11206801	6
rs10889569	6
rs11209002	6
rs2902440	6
rs11465804	6
rs10889676	6
rs4656784	6
rs11265260	6
rs6077251	6
rs151361	6
rs1006899	6
rs9609565	6
rs13021737	6
rs11677203	6
rs13396424	6
rs12328675	6
rs824931	6
rs9815195	6
rs11129078	6
rs3773643	6
rs7629490	6
rs16850864	6

rs7689350	6
rs10519410	6
rs73132886	6
rs4869419	6
rs4713039	6
rs9468811	6
rs3130284	6
rs2390593	6
rs6601299	6
rs1208	6
rs13702	6
rs17786744	6
rs2444896	6
rs4256922	6
rs4363506	6
rs1353649	6
rs652722	6
rs7951105	6
rs2176598	6
rs4293143	6
rs74380195	6
rs4936518	6
rs12832485	6
rs7137869	6
rs830124	6
rs9542236	6
rs571411	6
rs17536328	6
rs9567307	6
rs7325564	6
rs9600079	6
rs10132280	6
rs7494275	6
rs765899	6
rs999737	6
rs7141420	6
rs7141420	6
rs10220309	6
rs10131728	6
rs2093746	6
rs8042543	6
rs4620914	6
rs1533665	6
rs2307449	6
rs11648796	6
rs5743289	6
rs5743289	6
rs4784223	6
rs9932186	6
rs1728785	6
rs12942547	6
rs1431318	6
rs1019238	6
rs8079702	6
rs8079702	6

rs10502575	6
rs12967135	6
rs11661646	6
rs11663206	6
rs2163813	6
rs8102476	6
rs2746347	6
rs12402265	6
rs11209002	6
rs2902440	6
rs11465804	6
rs10889676	6
rs10443196	6
rs17450029	6
rs11205006	6
rs3761959	6
rs3761959	6
rs4233356	6
rs9427573	6
rs2786111	6
rs11118993	6
rs6044112	6
rs6132333	6
rs4810479	6
rs6094710	6
rs11089937	6
rs6733301	6
rs6753473	6
rs1526687	6
rs7565301	6
rs6545883	6
rs6738962	6
rs12999542	6
rs7567288	6
rs11890028	6
rs13010713	6
rs11691711	6
rs62184315	6
rs1024161	6
rs1024161	6
rs12470505	6
rs12472274	6
rs6799705	6
rs73071352	6
rs73071352	6
rs6782264	6
rs1670533	6
rs2292298	6
rs4389526	6
rs788867	6
rs994014	6
rs1463104	6
rs2609255	6
rs114452217	6
rs1443170	6

rs4547811	6
rs1633735	6
rs795544	6
rs73069924	6
rs6452790	6
rs2115172	6
rs10077875	6
rs2405522	6
rs11740474	6
rs58873874	6
rs10040979	6
rs12212674	6
rs2894207	6
rs3134792	6
rs3134792	6
rs6910071	6
rs9268402	6
rs6457620	6
rs6457620	6
rs9276606	6
rs3747767	6
rs2530544	6
rs6961611	6
rs73215715	6
rs17153527	6
rs4730250	6
rs634010	6
rs4609139	6
rs722782	6
rs75609241	6
rs7815909	6
rs3110127	6
rs10957961	6
rs7004633	6
rs7004633	6
rs17359493	6
rs6469804	6
rs7837688	6
rs1478110	6
rs7045640	6
rs2479106	6
rs569434	6
rs12772243	6
rs10901513	6
rs11820589	6
rs11863065	6
rs10494270	6
rs3914132	6
rs13240464	6
rs1149933	6
rs12265836	6
rs1200821	6
rs703970	6
rs77216358	6
rs11065706	6

rs4942242	6
rs8002779	6
rs9788721	6
rs12596210	6
rs4839516	6
rs4382726	6
rs60490158	6
rs62174474	6
rs13023239	6
rs3770549	6
rs7607369	6
rs16864755	6
rs1516459	6
rs7612581	6
rs9810233	6
rs13074924	6
rs7656730	6
rs13135284	6
rs253959	6
rs1150561	6
rs9357155	6
rs7742824	6
rs1449672	6
rs10259199	6
rs114945094	6
rs6559140	6
rs1331623	6
rs4821544	1a
rs3218255	1b
rs2121875	1b
rs2535629	1b
rs2370759	1f
rs16928809	1f
rs1692120	1f
rs3825932	1f
rs12915845	1f
rs11649653	1f
rs3785574	1f
rs6997	1f
rs10004195	1f
rs2269426	1f
rs9268528	1f
rs2858870	1f
rs798497	1f
rs471364	1f
rs11789898	1f
rs4950928	1f
rs11167260	1f
rs1883832	1f
rs2318131	1f
rs6489785	1f
rs154657	1f
rs6088792	1f
rs12627933	1f
rs6863411	1f

rs6457327	1f
rs3130618	1f
rs3130618	1f
rs3129882	1f
rs3129882	1f
rs7748270	1f
rs7117404	1f
rs2074518	1f
rs3894194	1f
rs3894194	1f
rs867186	1f
rs12489828	1f
rs7192	1f
rs660895	1f
rs660895	1f
rs7386474	1f
rs7025162	1f
rs3731714	1f
rs971768	1f
rs58370486	2a
rs1256531	2a
rs10849441	2b
rs1227756	2b
rs963167	2b
rs3922435	2b
rs835367	2b
rs12032643	2b
rs79384503	2b
rs10938494	2b
rs7770731	2b
rs7341237	2b
rs4410871	2b
rs4410871	2b
rs2302729	2b
rs11150589	2b
rs2476601	2b
rs2476601	2b
rs2476601	2b
rs2476601	2b
rs2476601	2b
rs2476601	2b
rs2476601	2b
rs2073398	2b
rs7571816	2b
rs422421	2b
rs3824999	2b
rs4846922	2b
rs319598	2b
rs3805663	2b
rs4711279	2b
rs10456100	2b
rs2796441	2b
rs290475	2b
rs600231	2b
rs1144713	2b

rs17356907	2b
rs17356907	2b
rs9525916	2b
rs9525916	2b
rs704	2b
rs2287838	2b
rs8109578	2b
rs1342038	2b
rs17310467	2b
rs11684202	2b
rs2302612	2b
rs2667011	2b
rs169082	2b
rs67250450	2b
rs10781380	2b
rs11539570	2b
rs11264330	2b
rs643381	2b
rs55874825	2c
rs74346392	2c
rs9295474	2c
rs409974	3a
rs11605083	3a
rs116909374	3a
rs7229639	3a
rs307896	3a
rs10797919	3a
rs669408	3a
rs11919041	3a
rs789852	3a
rs9461776	3a
rs16872971	3a
rs16872971	3a
rs1889321	3a
rs946053	3a
rs1800775	3a
rs1800775	3a
rs1800775	3a
rs1800775	3a
rs1800775	3a
rs9896933	3a
rs13394720	3a
rs11892031	3a
rs7164883	3a
rs4670766	3a
rs13358864	3a
rs757369	3a
rs1873147	3a
rs11205303	3a
rs11205303	3a
rs13045180	3a
rs496300	3a
rs1982346	3a
rs17026425	3a
rs149709	3a

rs1342371	3a
rs10808265	3a
rs483610	3a
rs75825892	3a
